# scQCEA: a framework for annotation and quality control report of single-cell RNA-sequencing data

**DOI:** 10.1186/s12864-023-09447-6

**Published:** 2023-07-06

**Authors:** Isar Nassiri, Benjamin Fairfax, Angela Lee, Yanxia Wu, David Buck, Paolo Piazza

**Affiliations:** 1 Oxford Genomics Centre, Nuffield Department of Medicine, Wellcome Centre for Human Genetics, University of Oxford, Oxford, UK; 2 MRC–Weatherall Institute of Molecular Medicine, University of Oxford, Oxford, UK; 3 Department of Oncology, University of Oxford & Oxford Cancer Centre, Churchill Hospital, Oxford University Hospitals NHS Foundation Trust, Oxford, UK

**Keywords:** Single cell RNA sequencing, Transcriptomics, Genomics, Cell type annotation

## Abstract

**Background:**

Systematic description of library quality and sequencing performance of single-cell RNA sequencing (scRNA-seq) data is imperative for subsequent downstream modules, including re-pooling libraries. While several packages have been developed to visualise quality control (QC) metrics for scRNA-seq data, they do not include expression-based QC to discriminate between true variation and background noise.

**Results:**

We present scQCEA (acronym of the single-cell RNA sequencing Quality Control and Enrichment Analysis), an R package to generate reports of process optimisation metrics for comparing sets of samples and visual evaluation of quality scores. scQCEA can import data from 10X or other single-cell platforms and includes functions for generating an interactive report of QC metrics for multi-omics data. In addition, scQCEA provides automated cell type annotation on scRNA-seq data using differential gene expression patterns for expression-based quality control. We provide a repository of reference gene sets, including 2348 marker genes, which are exclusively expressed in 95 human and mouse cell types.

Using scRNA-seq data from 56 gene expressions and V(D)J T cell replicates, we show how scQCEA can be applied for the visual evaluation of quality scores for sets of samples. In addition, we use the summary of QC measures from 342 human and mouse shallow-sequenced gene expression profiles to specify optimal sequencing requirements to run a cell-type enrichment analysis function.

**Conclusions:**

The open-source R tool will allow examining biases and outliers over biological and technical measures, and objective selection of optimal cluster numbers before downstream analysis. scQCEA is available at https://isarnassiri.github.io/scQCEA/ as an R package. Full documentation, including an example, is provided on the package website.

**Supplementary Information:**

The online version contains supplementary material available at 10.1186/s12864-023-09447-6.

## Background

Quality control is a critical step to identify biases during sequencing or alignment for single-cell RNA sequencing data (scRNA-seq) and can be a tedious task to evaluate multiple samples separately [1]. We present the scQCEA tool for generating interactive reports of process optimisation metrics and visual evaluation of quality scores for sets of samples. In addition, expression-based quality control using cell-type enrichment analysis is integrated into the scQCEA workflow. These are done in a single R software environment and easy to use for those who possess little or no programming language skills. A step-by-step workflow vignette demonstrates the detailed use of scQCEA (https://isarnassiri.github.io/scQCEA/).

**Fig. 1:**
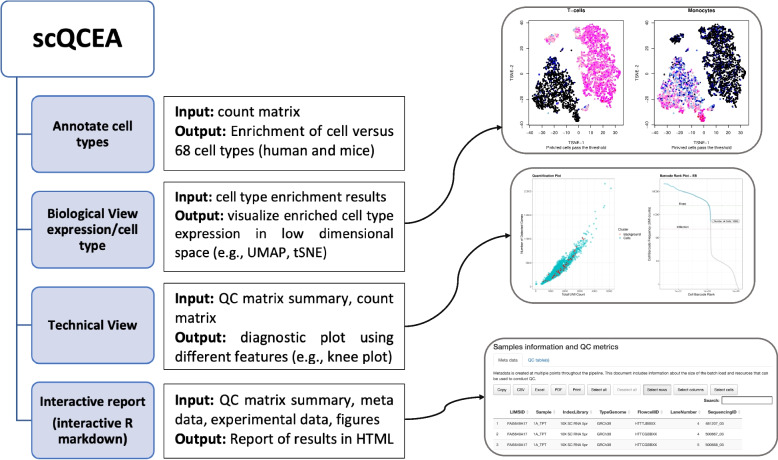
The outline of scQCEA. The scQCEA workflow is developed in R and can import datasets generated from pre-processing tools. The workflow incorporates various packages to perform cell-type enrichment analysis from gene sets and generates an interactive QC report to compare multiple sample sets over QC metrics. See text for details

The scQCEA workflow assumes that raw count data, a summary of the alignment and assignment of reads to cells and genes have been created by a tool for data pre-processing, such as CellRanger [2]. The steps in the workflow include (1) generating a description of the computational experiment per application (CITE, GEX, VDJ, HTO-CMO, mxATAC, ATAC), (2) visualisation of metadata document including information about the batches for data loads, (3) visualisation of quality control measures, separated by samples, (4) visualisation of cell type annotation on scRNA-seq profiles for expression-based quality control evaluation (Fig. 1). To demonstrate the utility of scQCEA, we apply the workflow to the sixteen gene expression profiles of eight patients with metastatic melanoma, prepared from pre- and post-treatment experimental batches [3].

### Implementation

scQCEA (acronym of the single-cell RNA sequencing Quality Control and Enrichment Analysis) is written in R, combining Shiny and Markdown. scQCEA creates an interactive QC report in one HTML file, which includes four sections: experimental workflow, data processing workflow, samples information and QC metrics, data analysis and quality control (Fig. 1). “Experimental workflow” describes the scRNA-seq transcriptome processing and sequencing platform. “Data processing workflow” presents an analysis pipeline to process data, including aligning reads, generating feature-barcode matrices and other secondary analyses. The content of the “Data processing workflow” section is automatically adjusted based on the type of application(s) and the “Library Type” column in the metadata. The “Samples information and QC metrics” section provides tables of various process optimisation metrics for comparing sets of samples per application. The “Data analysis and quality control” section presents a projection of transcriptionally and functionally distinct clusters, highlighted by cell type group, including UMAP and t-SNE plots. Diagnostic plots provide technical features, such as inflection or knee points in the distribution of non-duplicate read counts inside the cell barcodes. Cells under the threshold provided by the Cell Ranger selection algorithm [4] are flagged and filtered out as empty droplets, and cells above the threshold do not enrich with any reference gene set to introduce as background noise. In addition, the cells introduced as noise by expression-based quality control (cell-type enrichment analysis) are projected onto quantification and UMAP plots.

**Fig. 2:**
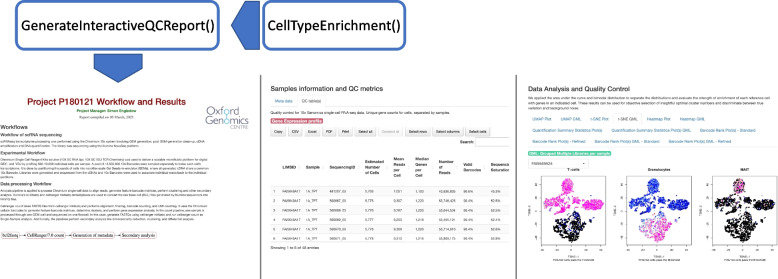
Generation of interactive HTML report for visual evaluation and comparison of multiple sample sets, over comprehensive technical and biological QC metrics using functions available in the scQCEA package. The InteractiveQCReport() function applies generated data by the CellTypeEnrichment() and other QC tools to create an interactive HTML report. The report contains figures visualising tSNE, UMAP, heatmap, quantification summary statistics, knee plots, and tables for sample sets. Examples of different sections of the report are shown

The easiest way to generate an interactive summary QC report is to run a function from the RStudio called GenerateInteractiveQCReport(), which utilises application-specific templates to generate an HTML report with the visualisations of QC metrics (Fig. 2). The required inputs are a gene-cell count matrix, feature-barcode matrices, and tSNE and UMAP projections from 10X CellRanger count. By running the function, all dependency packages automatically will be downloaded from CRAN-like repositories and installed. An interactive QC report automatically will be generated in one HTML file. Full documentation, including an example, is provided on the package website (https://isarnassiri.github.io/scQCEA/).

### Cell type enrichment analysis from gene sets

Cell type annotation on scRNA-seq data is a pre-step for generating an interactive QC report with scQCEA. This step requires some bioinformatics efforts, but scQCEA provides a function called CellTypeEnrichment() function, for automatic cell type identification and visualisation on the gene-by-cell count matrix.

We use the AUCell algorithm to enrich expressed genes for each cell individually, and gene sets exclusively expressed in each cell type [5]. It applies the area under the curve (AUC) and bimodal distribution to separate the distributions and evaluate the strength of enrichment of each reference cell with genes in an indicated cell. The AUC scores across all the cells represent the relative expression of the signature, therefore, we do not normalise the data before enrichment analysis. We refer the reader to [5] for evaluation of performance.

**Fig. 3:**
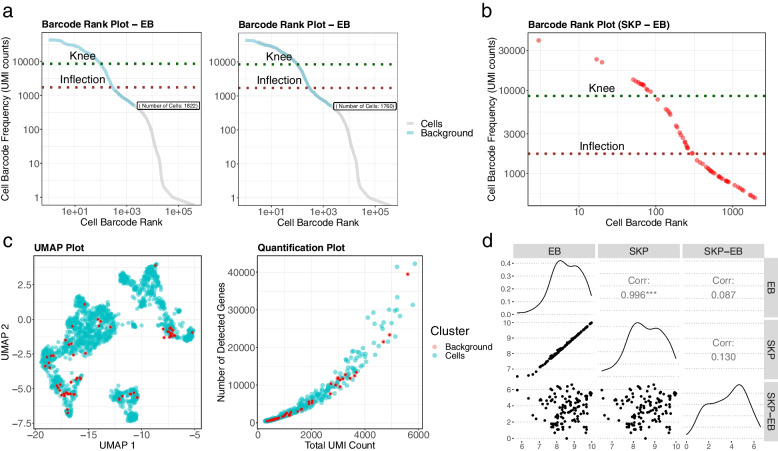
Discriminate between cells and background noise using cell-type enrichment analysis. **a** The knee plot is used for thresholding and selecting high-quality cells located on the left-hand side of the plot. In the barcode rank standard knee plot (SKP), the y-axis shows the value used to call cells, and the x-axis is the number of barcodes below that value. In the barcode rank expression-based plot (EB), cell-type specific enrichment analysis was applied to discriminate between true variation and background noise. As a result, we found 1760 cells enriched with different cell types. **b**, **c** Knee, quantification, and UMAP plots show the location of cells in relative complement (SKP-EB) of cells in SKP with respect to EB. To ensure that background red-coloured cells appear on top, we separate the points into different layers and plot the red points after the blue points. **d** Correlation analysis of the number of cells per sample and number of detected cells as background noise (SKP-EB) using cell-type enrichment analysis

The outputs of the CellTypeEnrichment() function include the visualisation of transcriptionally and functionally distinct clusters, highlighted by cell type group using Uniform Manifold Approximation and Projection (UMAP) and t-stochastic neighbour embedding (t-SNE) plots. UMAP, compared to t-SNE, constructs a high-dimensional graph representation of the data, including clusters that are as structurally similar as possible. In addition, the CellTypeEnrichment() function generates Heatmap, Quantification Summary Statistics, and Barcode Rank plots. Heatmap plot visualises cells showing enriched expressed genes in each cell type group. The Quantification Summary Statistics plot presents the distribution of total UMI versus the total number of detected genes. The Barcode Rank plot shows the distribution of non-duplicate reads, with a mapping quality of at least 30 per barcode associated with cells. The refined barcode rank plots are also created to visualise the selected cells using cell-type specific enrichment analysis to discriminate between true variation and background noise (Fig. 3).

The required inputs are a gene-cell count matrix, feature-barcode matrices, tSNE and UMAP projections in the format provided by 10X CellRanger count, and a repository of reference gene sets. We used the Human Protein Atlas database (version 22.0) to generate a repository of reference gene sets, which are exclusively expressed in each cell type [6]. The repository includes 95 pre-defined reference gene sets, and 2348 marker genes, and is available at https://github.com/isarnassiri/scQCEA/tree/Repository-of-Cell-Type-Specific-Gene-Sets. The repository of reference genes covers human and mouse genes, with the possibility of expansion to other species. Alternatively, users can apply gene sets associated with a biological process of interest (e.g., poorly differentiated cancer cells and stem cells) and perform enrichment analysis. The existing methods for the preparation of reference gene sets for rare or undergoing development cell types can be broadly categorised into two groups:

1. Knowledge-based method, that marker gene set is obtained from manual literature search or databases [7]. For instance, induced pluripotent stem cells (iPSCs) has four main sub-cell types (HIC2 + , ATF2 + , BRF2 + and CEBPG +). The transcription factors with high relative activities specific to iPSCs subtypes (e.g., *HMGB2*, *NR3C1*, *ATF2* for ATF2 + subtype) can be used as a reference gene set for the CellTypeEnrichment() function.
2. The second method involves three steps. First, we extract modules of co-expressed genes and transcription factors (TFs). Next, a transcription factor motif enrichment analysis is applied to prune each module and retain genes that contain sequence motifs related to TFs in the modules [5]. Lastly, the set of respective TFs and related co-expressed genes are used as back-end data (reference gene set) for a cell type enrichment tool (e.g., CellTypeEnrichment function).

## Results

We used the scRNA-seq profile of eight patients with metastatic melanoma, which includes a total of 48,768 cells in 48 replicates from the same set of cells, prepared from treatment with immune checkpoint blockade experimental batches [3]. Each batch had 24 replicates and aggregated into sixteen single feature-barcode matrices (8 pre- and 8 post-treatment). Supplementary File S1 is the output generated by scQCEA. In total, 33,325 cells passed quality control (Supplementary File S2). The interactive report of quality control metrics and image QC of profiles allowed visual evaluation and comparison of comprehensive QC metrics. The performance of the cell type annotation function was evaluated with existing labels for T cells and monocytes [3]. In the original paper, sub-setting was performed to select T cells expressing *CD8A*, *CD8B*, and *CD3D*, and monocytes expressing *CD14*. Further sub-setting excluded *CD14* cells expressing *CD3D* and *CD3E*, *CD3G*, *CD8E* and *CD19*, and T cells expressing *CD14* and *CD19* [3]. The results suggest that the cell type enrichment analysis captures the main clusters across cells, and samples share similar cellular compositions in agreement with existing labels.

In the next step, we used a sample whose knee plot was smooth with no cliff or knee, likely due to a large amount of ambient RNA in the background and poor wetting failure (Fig. 3a). The cell-type specific enrichment analysis was applied to discriminate between true variation and background noise. As a result, we found that 62 cells did not enrich with any cell type (Fig. 3b). The location of detected non-relevant cells on the knee plot showed aggregation after inflection point. It means filtering out non-relevant cells can improve the accuracy of cell calling, especially for samples with wetting failure. We observed ambient RNA profiles detected by enrichment analysis, mainly enriched in the bottom-left corner of the quantification plot (Fig. 3c). UMAP projection plot did not show an accumulation of ambient RNA profiles for specific clusters or regions (Fig. 3c). We compared the expression profile of these 62 cells versus the ambient RNA profile estimated using small barcodes by *the EmptyDrops* algorithm which did not flag them as empty droplets [8]. *Scran* package was applied to detect doublets/multiplets, and 29 cells scored as potential doublets (score > 1.5 marked as doublets) [9]. Therefore, the 62 cells cannot be some novel cell types, and by removing them, we do not lose potential novel biological findings. Further exploration using 286 single-cell gene expression profiles from humans and mice similarly did not show a significant association between the number of cells per sample and the number of detected cells as background noise using cell-type enrichment analysis (Supplementary File S1) (Fig. 3d). These findings confirm that confounding variables, such as cell detection rate, do not affect the application of cell-type specific enrichment analysis to discriminate between cells and expression activity associated with ambient RNA.

**Fig. 4:**
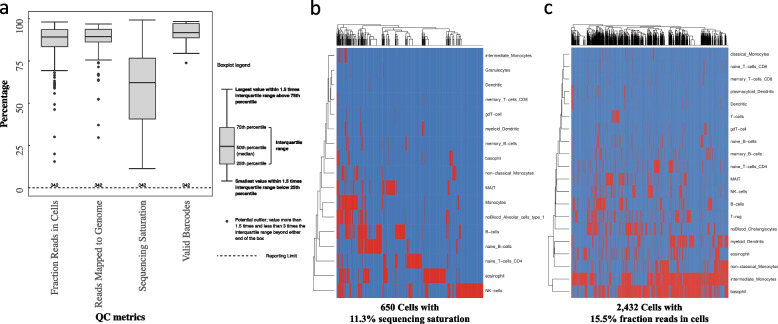
**a** QC metrics generated by the Cell Ranger tool for 342 human and mouse gene expression profiles. The CellTypeEnrichment() function could successfully generate results for samples with 11.3% sequencing saturation (**b**) and 15.5% fraction reads in cells (**c**)

We use the summary of QC measures from 342 shallow sequenced gene expression profiles from humans and mice to specify the optimal sequencing saturation required to run the CellTypeEnrichment() function (Fig. 4a). The results showed that sequencing saturation may influence the ability of cell type enrichment analysis using scQCEA, and the estimated minimum sequencing saturation and a fraction of reads in cells required for this tool to function are 11.3% and 15.5%, respectively (Fig. 4b-c). The recommended number of cells per sample and reads per cell for standard pooled scRNA-seq workflows would provide optimal input for the CellTypeEnrichment() function (50,000 reads per cell to have about 1,200 to 4,000 genes per cell).

### Comparison to other tools

Several other tools that can perform quality control have been introduced. While many formats are available to store the results of scRNA-seq analysis (e.g., h5ad), we use CSV as a generic data transfer format for required inputs to run scQCEA. Although other packages can generate interactive reports, including general QC metrics, scQCEA includes visualisation of different approaches (e.g., CITE-SEQ, VDJ, mxATAC). Furthermore, no other toolkits currently use cell-type enrichment analysis to allow expression-based quality control of sets of samples (Table 1). scQCEA can be applied to analyse and report both single cell 3' and 5' gene expression libraries, as the two assays are similar but capture different ends of transcripts. scQCEA generates a section in the interactive report, called GML (Grouped Multiple Libraries per sample), to present the aggregated QC measures for several sequencing runs of the same library (e.g., increase read depth by resequencing the same library).scQCEA visualises QC metrics in standardised HTML reports and stores result in zip format, which facilitates archiving the reports and keeping track of experimental and computational experiments. For a large data set including 56 gene expressions and 16 VDJ profiles, the run time of generating an interactive QC report using the GenerateInteractiveQCReport() function is 21.03 s.

**Table 1:** Comparison of features in the scQCEA workflow with other similar tools. Multiple applications: visualising the QC measures for scRNA-seq approaches in addition to gene expression (e.g., CITE-Seq and VDJ)

	**scQCEA**	**SCTK ** [10]	**PIVOT ** [11]	**Seurat ** [12]	**Ascend ** [13]	**scRNABatchQC ** [14]	**Adobo ** [15]	**SCONE ** [16]	**SCHNAPPs ** [17]	**iS-CellR ** [18]	**Ganatum ** [19]	**ASAP ** [20]
**Input Format**												
Input format CSV	✓	✓	✓	✓	✓	✓	✓		✓	✓	✓	✓
**General QC Metrics**												
Mean Reads per cell	✓	✓	✓	✓	✓	✓	✓	✓	✓	✓	✓	✓
Total genes	✓	✓	✓	✓	✓	✓	✓	✓	✓	✓	✓	✓
**HTML Report**												
Interactive report	✓	✓			✓	✓				✓		✓
Compare multiple samples	✓	✓				✓						
Multiple applications	✓											
**Additional Functionalities**												
Expression-based QC	✓											
Ambient droplets detection		✓										

We compare the run time of the CellTypeEnrichment() function with the ScType tool, as currently the fastest unsupervised method for cell-type enrichment analysis [7]. For this comparison, we used a dataset of 2,700 single cells from Peripheral Blood Mononuclear Cells (PBMCs) available from the ScType package website [7]. The results of the run test showed 27.57 and 27.47 s for scQCEA and ScType, respectively (2.6 GHz 6-Core Intel Core i7, 16 GB RAM, MacOS).

## Discussion

scRNA-seq is increasingly used to study transcriptomics at high resolution, and the evaluation of QC metrics for large-scale projects is not a straightforward process. Here, we present an R package, scQCEA, that provides a convenient workflow to present QC measures in the form of interactive tables and graphical plots for different scRNA-seq experiments. scQCEA facilitates visual evaluation of base quality, capture-efficiency, and expression-based quality metrics. In addition, the interactive QC report is particularly useful to share or store the documented history of experiments and a summary of QC, which will improve the reproducibility of analysis.

Although other packages like scRNABatchQC [14] and SCTK-QC [10] have been introduced to simplify the process of generating and visualising varieties of QC metrics, scQCEA provides a convenient workflow to generate an interactive report for different scRNA-seq experiments and visual evaluation of sets of samples. In addition, scQCEA facilitates the objective optimal cluster numbers selection using the enrichment of highly expressed gene sets in each cell. A general approach for cluster annotation consists of a gene set enrichment analysis by using the marker genes defining each cluster [21]. For example, SCSA [22] uses reference lists of markers from multiple sources to annotate and interpret scRNA-seq data. In contrast, reference-based tools such as ScType [7] annotate cells by comparing new data with existing training/reference collection of cell types. These methods successfully project cells onto cell types and compare multiple QC metrics, which gives valuable hints about technical and biological features. However, these cell annotation methods aggregate whole transcriptome gene expression data and introduce technical variability, which decreases the power to find cell–cell differences [21]. On the other hand, estimating the optimal and biologically meaningful number of clusters from the data depends on the user’s subjective choice [23]. Problems such as inconsistency among reference or training data sets still exist in classification methods for cell-type identification [22]. To address these issues, scQCEA by evaluating cells individually to score active cell type signature (set of genes with exclusive elevated expression in an indicated cell type) increases the power to find enrichments, independent of the clustering method and training dataset [5]. In addition, we applied cell-type specific enrichment analysis to discriminate between true variation and background noise. This approach is especially useful for experiments in which the knee plot works poorly due to a lack of clear knee points for thresholding high-quality cells (e.g., wetting failure) [24, 25].

## Conclusions

In summary, the scQCEA package, with two functions for cell-type enrichment analysis and generating an interactive QC report, provides infrastructure for different applications, including the delivery of high-quality genomic services.

## Supplementary Information

**Additional file 1: Supplementary file 1.** Supplementary Methods including data pre-processing and presentation.

**Additional file 2: Supplementary file 2. **To demonstrate the utility of scQCEA, we apply the workflow to the sixteen gene expression profiles of eight patients with metastatic melanoma, prepared from pre- and post-treatment experimental batches. You can find the QC interactive report at: https://github.com/isarnassiri/scQCEA/tree/Example-of-Application. Download and unzip the OGC_Interactive_QC_Report_P180121.zip file. You can open CLICK_ME.html file without using rStudio/R.

## Data Availability

The human and mouse reference dataset, GRCh38 and mm10 and all required reference files used in this article are publicly available through the Resource bundle of 10xgenomics at: https://cf.10xgenomics.com/supp/cell-exp/refdata-gex-GRCh38-2020-A.tar.gz, https://cf.10xgenomics.com/supp/cell-exp/refdata-gex-mm10-2020-A.tar.gz. The interactive QC reports for sixteen gene expression profiles of eight patients with metastatic melanoma are available at https://github.com/isarnassiri/scQCEA/tree/Example-of-Application. Detailed documentation to run and use scQCEA is available at https://isarnassiri.github.io/scQCEA/.

## Abbreviations

ATAC-seq: Assay for transposase-accessible chromatin using sequencing
CITE-seq: Cellular indexing of transcriptomes and epitopes by sequencing
EB: Barcode rank expression-based plot
GEX: Single-Cell Gene Expression
HTO-CMO: Hash-tag oligos or cell multiplex oligos
mxATAC: Single Cell Multiome ATAC
QC: Quality control
scRNA-seq: Single-cell RNA sequencing data
scQCEA: Single-cell RNA sequencing Quality Control and Enrichment Analysis
SKP: Barcode rank standard knee plot
VDJ: Single Cell 5′ V(D)J libraries
t-SNE: T-stochastic neighbour embedding
UMAP: Uniform Manifold Approximation and Projection
