# Supplementary material for: scQCEA: a framework for annotation and quality control report of single-cell RNA-sequencing data
Source: BMC Genomics. 2023 Jul 6;24:381. doi: 10.1186/s12864-023-09447-6 (PMC10327311; doi:10.1186/s12864-023-09447-6)
Supplement: Supplementary file 1 — Additional file 1: Supplementary file 1. Supplementary Methods including data pre-processing and presentation. [file 12864_2023_9447_MOESM1_ESM.docx]

Supplementary information for the paper:

**scQCEA: A Framework for Annotation and Quality Control Report of Single-Cell RNA-Sequencing Data**

Isar Nassiri^1*^, Benjamin Fairfax^2,3^, Angela Lee^1^, Yanxia Wu^1^, David Buck^1^, Paolo Piazza^1*^

1 Oxford Genomics Centre, Wellcome Centre for Human Genetics, Nuffield Department of Medicine, University of Oxford, Oxford, UK, 2 MRC–Weatherall Institute of Molecular Medicine, University of Oxford, Oxford, UK, 3 Department of Oncology, University of Oxford & Oxford Cancer Centre, Churchill Hospital, Oxford University Hospitals NHS Foundation Trust, Oxford, UK

**Supplementary Methods**

**Data pre-processing**

The Cell Ranger pipelines were used to process sequencing data. Cellranger (v7) mkfastq was applied to the Illumina BCL output to produce FASTQ files. Cellranger count (v7) was then applied to each FASTQ file to produce a Feature Barcoding and Gene expression library. We converted an HDF5 Feature-Barcode Matrix to a gene-cell count matrix using the cellranger mat2csv command provided by 10X genomics as the input for downstream analysis [1].

**Data presentation**

Plots were generated using ggpubr (v0.2) and customizing ggplot2 [2]. We leveraged HTML document from R Markdown to develop an interactive report for programming-free graphical and interactive evaluation of QC metrics, visualization of cell-type annotation for each cell, and objective selection of insightful optimal cluster numbers.

**Reference:**

1. Fairfax BP, Taylor CA, Watson RA, Nassiri I, Danielli S, Fang H, Mahé EA, Cooper R, Woodcock V, Traill Z *et al*: **Peripheral CD8+ T cell characteristics associated with durable responses to immune checkpoint blockade in patients with metastatic melanoma**. *Nature Medicine* 2020, **26**(2):193-199.

2. Almeida A, Loy A, Hofmann H: **ggplot2 Compatible Quantile-Quantile Plots in R**. *R J* 2018, **10**(2):248-261.
