# Supplementary material for: scQCEA: a framework for annotation and quality control report of single-cell RNA-sequencing data
Source: BMC Genomics. 2023 Jul 6;24:381. doi: 10.1186/s12864-023-09447-6 (PMC10327311; doi:10.1186/s12864-023-09447-6)
Supplement: Supplementary file 2 — Additional file 2: Supplementary file 2. To demonstrate the utility of scQCEA, we apply the workflow to the sixteen gene expression profiles of eight patients with metastatic melanoma, prepared from pre- and post-treatment experimental batches. You can find the QC interactive report at: https://github.com/isarnassiri/scQCEA/tree/Example-of-Application. Download and unzip the OGC_Interactive_QC_Report_P180121.zip file. You can open CLICK_ME.html file without using rStudio/R. [file 12864_2023_9447_MOESM2_ESM.zip › CLICK_ME.html]

Project P180121 Workflow and Results


# Project P180121 Workflow and Results

#### Project Manager: Simon Engledow

#### Report compiled on 03 March, 2023


### Workflows

#### Workflow of scRNA sequencing

scRNAseq transcriptome processing was performed using the Chromium
10x system involving GEM generation, post GEM-generation clean-up, cDNA
amplification and DNA quantification. The library was sequencing using
the Illumina NovaSeq platform.

#### Experimental Workflow

Chromium Single Cell Reagent Kits solution (10X SC RNA 5pr, 10X SC
VDJ TCR Chemistry) was used to deliver a scalable microfluidic platform
for digital GEX’, and ’VDJ by profiling 500-10,000 individual cells per
sample. A pool of ~3,500,000 10x Barcodes were sampled separately to
index each cell’s transcriptome. It is done by partitioning thousands of
cells into nanoliter-scale Gel Beads-in-emulsion (GEMs), where all
generated cDNA share a common 10x Barcode. Libraries were generated and
sequenced from the cDNAs and 10x Barcodes were used to associate
individual reads back to the individual partitions.

#### Data processing Workflow

Analysis pipeline is applied to process Chromium single-cell data to
align reads, generate feature-barcode matrices, perform clustering and
other secondary analysis. Illumina’s bcl2fastq and cellranger mkfastq
demultiplexes are used to convert the raw base call (BCL) files
generated by Illumina sequencers into FASTQ files.

Cellranger count takes FASTQ files from cellranger mkfastq and
performs alignment, filtering, barcode counting, and UMI counting. It
uses the Chromium cellular barcodes to generate feature-barcode
matrices, determine clusters, and perform gene expression analysis. In
the count pipeline, one sample is processed through one GEM well and
sequenced on one flowcell. In this case, generate FASTQs using
cellranger mkfastq and run cellranger count as Single-Sample analysis.
Additionally, the pipelines perform secondary analysis like
dimensionality reduction, clustering, and differential analysis.

In the grouped count pipeline, one sample is processed through one
GEM well, resulting in one library which is sequenced across multiple
flowcells. This workflow is commonly performed to increase sequencing
depth. In this case, all reads can be combined in a single instance of
the cellranger count pipeline. Finally, the pipelines perform secondary
analysis like dimensionality reduction, clustering, and differential
analysis.

To generate single-cell V(D)J sequences and annotations for a single
library, run cellranger vdj with the following parameters.

### Samples information and QC metrics

#### Meta data

Metadata is created at multiple points throughout the pipeline. This
document includes information about the size of the batch load and
resources that can be used to conduct QC.

#### QC table(s)

Quality control for 10x Genomics single-cell RNA-seq data, Unique
gene counts for cells, separated by samples.

Gene
Expression profile

Gene
Expression profiles - grouped

Single
Cell Immune Profiling

### Data Analysis and Quality Control

We applied the area under the curve and bimodal distribution to
separate the distributions and evaluate the strength of enrichment of
each reference cell with genes in an indicated cell. These results can
be used for objective selection of insightful optimal cluster numbers
and discriminate between true variation and background noise.

#### UMAP Plot

Samples

481207\_03
481207\_15
481207\_28
481207\_40
481207\_52
481207\_64
481207\_76
481207\_88
500667\_03
500667\_15
500667\_28
500667\_40
500667\_52
500667\_64
500667\_76
500667\_88
500668\_03
500668\_15
500668\_28
500668\_40
500668\_52
500668\_64
500668\_76
500668\_88
500669\_03
500669\_15
500669\_28
500669\_40
500669\_52
500669\_64
500669\_76
500669\_88
500670\_03
500670\_15
500670\_28
500670\_40
500670\_52
500670\_64
500670\_76
500670\_88
500671\_03
500671\_15
500671\_28
500671\_40
500671\_52
500671\_64
500671\_76
500671\_88

Uniform Manifold Approximation and Projection (UMAP) projection of
transcriptionally and functionally distinct clusters, highlighted by
cell type group. UMAP constructs a high-dimensional graph representation
of the data, then builds a low-dimensional graph that is as structurally
similar as possible.

#### UMAP GML

GML:
Grouped Multiple Libraries per sample

FAI5649A17
FAI5649A18
FAI5649A19
FAI5649A20
FAI5649A21
FAI5649A22
FAI5649A23
FAI5649A24

Uniform Manifold Approximation and Projection (UMAP) projection of
transcriptionally and functionally distinct clusters, highlighted by
cell type group. UMAP constructs a high-dimensional graph representation
of the data, then builds a low-dimensional graph that is as structurally
similar as possible.

#### t-SNE Plot

Samples

481207\_03
481207\_15
481207\_28
481207\_40
481207\_52
481207\_64
481207\_76
481207\_88
500667\_03
500667\_15
500667\_28
500667\_40
500667\_52
500667\_64
500667\_76
500667\_88
500668\_03
500668\_15
500668\_28
500668\_40
500668\_52
500668\_64
500668\_76
500668\_88
500669\_03
500669\_15
500669\_28
500669\_40
500669\_52
500669\_64
500669\_76
500669\_88
500670\_03
500670\_15
500670\_28
500670\_40
500670\_52
500670\_64
500670\_76
500670\_88
500671\_03
500671\_15
500671\_28
500671\_40
500671\_52
500671\_64
500671\_76
500671\_88

t-stochastic neighbor embedding (t-SNE) projection of transcriptionally
and functionally distinct clusters, highlighted by cell type group.

#### t-SNE GML

GML:
Grouped Multiple Libraries per sample

FAI5649A17
FAI5649A18
FAI5649A19
FAI5649A20
FAI5649A21
FAI5649A22
FAI5649A23
FAI5649A24

t-stochastic neighbor embedding (t-SNE) projection of transcriptionally
and functionally distinct clusters, highlighted by cell type group.

#### Heatmap Plot

Samples

481207\_03
481207\_15
481207\_28
481207\_40
481207\_52
481207\_64
481207\_76
481207\_88
500667\_03
500667\_15
500667\_28
500667\_40
500667\_52
500667\_64
500667\_76
500667\_88
500668\_03
500668\_15
500668\_28
500668\_40
500668\_52
500668\_64
500668\_76
500668\_88
500669\_03
500669\_15
500669\_28
500669\_40
500669\_52
500669\_64
500669\_76
500669\_88
500670\_03
500670\_15
500670\_28
500670\_40
500670\_52
500670\_64
500670\_76
500670\_88
500671\_03
500671\_15
500671\_28
500671\_40
500671\_52
500671\_64
500671\_76
500671\_88

Heatmap based on cells showing the most enriched expressed genes in each
cell type group. Red cells passed the threshold.

#### Heatmap GML

GML:
Grouped Multiple Libraries per sample

FAI5649A17
FAI5649A18
FAI5649A19
FAI5649A20
FAI5649A21
FAI5649A22
FAI5649A23
FAI5649A24

Heatmap based on cells showing the most enriched expressed genes in each
cell type group. Red cells passed the threshold.

#### Quantification Summary Statistics Plot(s)

Samples

481207\_03
481207\_15
481207\_28
481207\_40
481207\_52
481207\_64
481207\_76
481207\_88
500667\_03
500667\_15
500667\_28
500667\_40
500667\_52
500667\_64
500667\_76
500667\_88
500668\_03
500668\_15
500668\_28
500668\_40
500668\_52
500668\_64
500668\_76
500668\_88
500669\_03
500669\_15
500669\_28
500669\_40
500669\_52
500669\_64
500669\_76
500669\_88
500670\_03
500670\_15
500670\_28
500670\_40
500670\_52
500670\_64
500670\_76
500670\_88
500671\_03
500671\_15
500671\_28
500671\_40
500671\_52
500671\_64
500671\_76
500671\_88

The total UMI vs detected genes plot shows the distribution of total UMI
versus total numer of detected genes. The cell-type specific enrichment
analysis was applied to discriminate between true variation (Cells, gray
colour) and background noise (Background, purple colour), and filter out
the cells (Background) which did not enrich with any cell type.

#### Quantification Summary Statistics Plot(s) GML

Samples

FAI5649A17
FAI5649A18
FAI5649A19
FAI5649A20
FAI5649A21
FAI5649A22
FAI5649A23
FAI5649A24

The total UMI vs detected genes plot shows the distribution of total UMI
versus total numer of detected genes. The cell-type specific enrichment
analysis was applied to discriminate between true variation (Cells, gray
colour) and background noise (Background, purple colour), and filter out
the cells (Background) which did not enrich with any cell type.

#### Barcode Rank Plot(s) - Standard

Samples

481207\_03
481207\_15
481207\_28
481207\_40
481207\_52
481207\_64
481207\_76
481207\_88
500667\_03
500667\_15
500667\_28
500667\_40
500667\_52
500667\_64
500667\_76
500667\_88
500668\_03
500668\_15
500668\_28
500668\_40
500668\_52
500668\_64
500668\_76
500668\_88
500669\_03
500669\_15
500669\_28
500669\_40
500669\_52
500669\_64
500669\_76
500669\_88
500670\_03
500670\_15
500670\_28
500670\_40
500670\_52
500670\_64
500670\_76
500670\_88
500671\_03
500671\_15
500671\_28
500671\_40
500671\_52
500671\_64
500671\_76
500671\_88

The Barcode Rank Plot shows the distribution of non-duplicate reads with
mapping quality at least 30 per barcode and which barcodes were inferred
to be associated with cells. The y-axis shows the value that
cellranger-dna uses to call cells and the x-axis is the number of
barcodes below that value.

#### Barcode Rank Plot(s) - Refined

Samples

481207\_03
481207\_15
481207\_28
481207\_40
481207\_52
481207\_64
481207\_76
481207\_88
500667\_03
500667\_15
500667\_28
500667\_40
500667\_52
500667\_64
500667\_76
500667\_88
500668\_03
500668\_15
500668\_28
500668\_40
500668\_52
500668\_64
500668\_76
500668\_88
500669\_03
500669\_15
500669\_28
500669\_40
500669\_52
500669\_64
500669\_76
500669\_88
500670\_03
500670\_15
500670\_28
500670\_40
500670\_52
500670\_64
500670\_76
500670\_88
500671\_03
500671\_15
500671\_28
500671\_40
500671\_52
500671\_64
500671\_76
500671\_88

The Barcode Rank Plot shows the distribution of non-duplicate reads with
mapping quality at least 30 per barcode and which barcodes were inferred
to be associated with cells. The cell-type specific enrichment analysis
was applied to discriminate between true variation and background noise,
and refine the results. The y-axis shows the value that cellranger-dna
uses to call cells and the x-axis is the number of barcodes below that
value.

#### Barcode Rank Plot(s) GML - Standard

GML:
Grouped Multiple Libraries per sample

FAI5649A17
FAI5649A18
FAI5649A19
FAI5649A20
FAI5649A21
FAI5649A22
FAI5649A23
FAI5649A24

The Barcode Rank Plot shows the distribution of non-duplicate reads with
mapping quality at least 30 per barcode and which barcodes were inferred
to be associated with cells. The y-axis shows the value that
cellranger-dna uses to call cells and the x-axis is the number of
barcodes below that value.

#### Barcode Rank Plot(s) GML - Refined

GML:
Grouped Multiple Libraries per sample

FAI5649A17
FAI5649A18
FAI5649A19
FAI5649A20
FAI5649A21
FAI5649A22
FAI5649A23
FAI5649A24

The Barcode Rank Plot shows the distribution of non-duplicate reads with
mapping quality at least 30 per barcode and which barcodes were inferred
to be associated with cells. The cell-type specific enrichment analysis
was applied to discriminate between true variation and background noise,
and refine the results. The y-axis shows the value that cellranger-dna
uses to call cells and the x-axis is the number of barcodes below that
value.

### Link for Further Information

The analysis, gene count matrix, and reports have been copied to the
“additional\_analysis” folder on the FTP server (LINK).
