# Supplementary figures and images for: scQCEA: a framework for annotation and quality control report of single-cell RNA-sequencing data (part 2 of 2)
Source: BMC Genomics. 2023 Jul 6;24:381. doi: 10.1186/s12864-023-09447-6 (PMC10327311; doi:10.1186/s12864-023-09447-6)

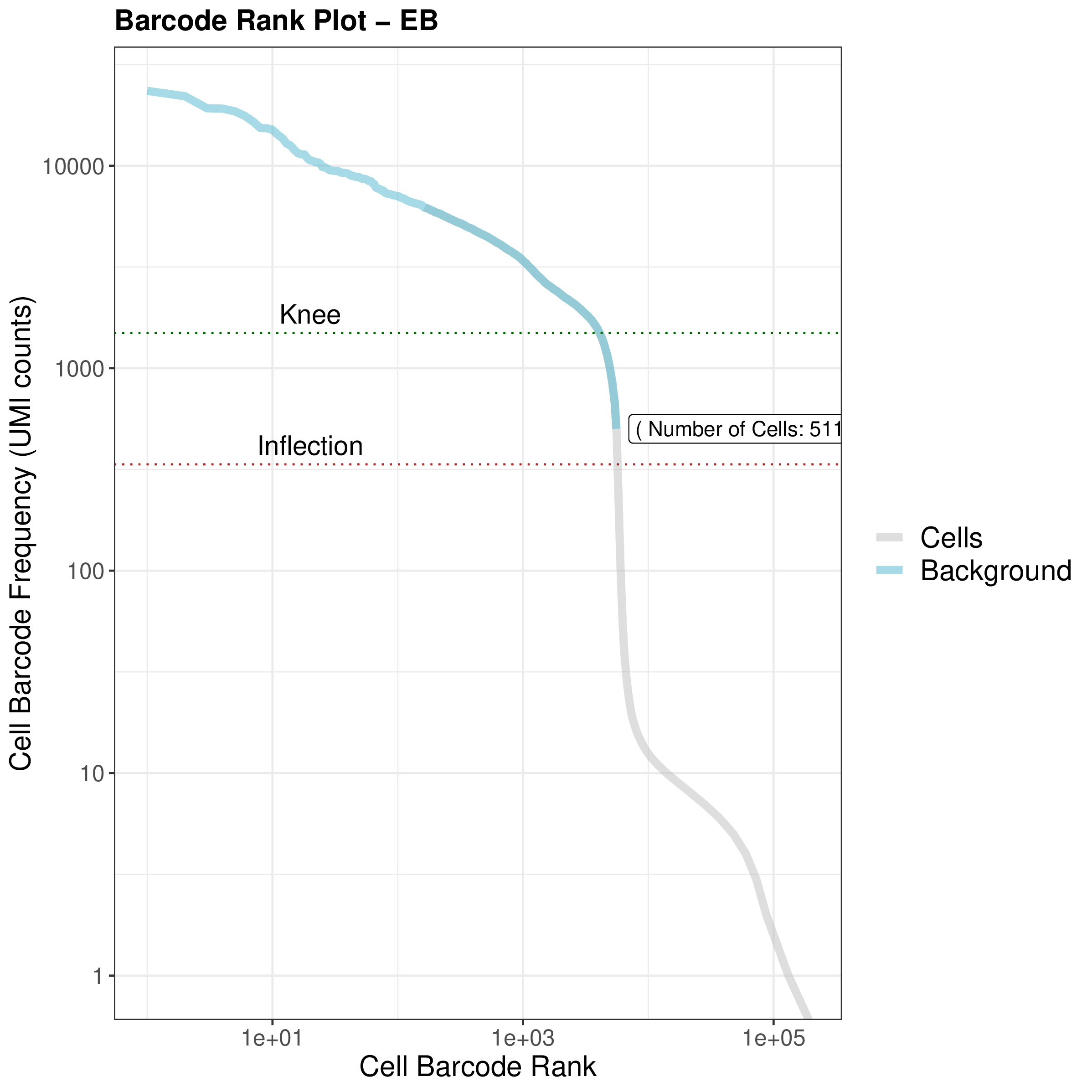

Supplement: Supplementary file 2 — Additional file 2: Supplementary file 2. To demonstrate the utility of scQCEA, we apply the workflow to the sixteen gene expression profiles of eight patients with metastatic melanoma, prepared from pre- and post-treatment experimental batches. You can find the QC interactive report at: https://github.com/isarnassiri/scQCEA/tree/Example-of-Application. Download and unzip the OGC_Interactive_QC_Report_P180121.zip file. You can open CLICK_ME.html file without using rStudio/R. [file 12864_2023_9447_MOESM2_ESM.zip › Inputs/10X-gex/481207_76/P180121-keep_481207_76_BarcodeRankPlot_EB.png]

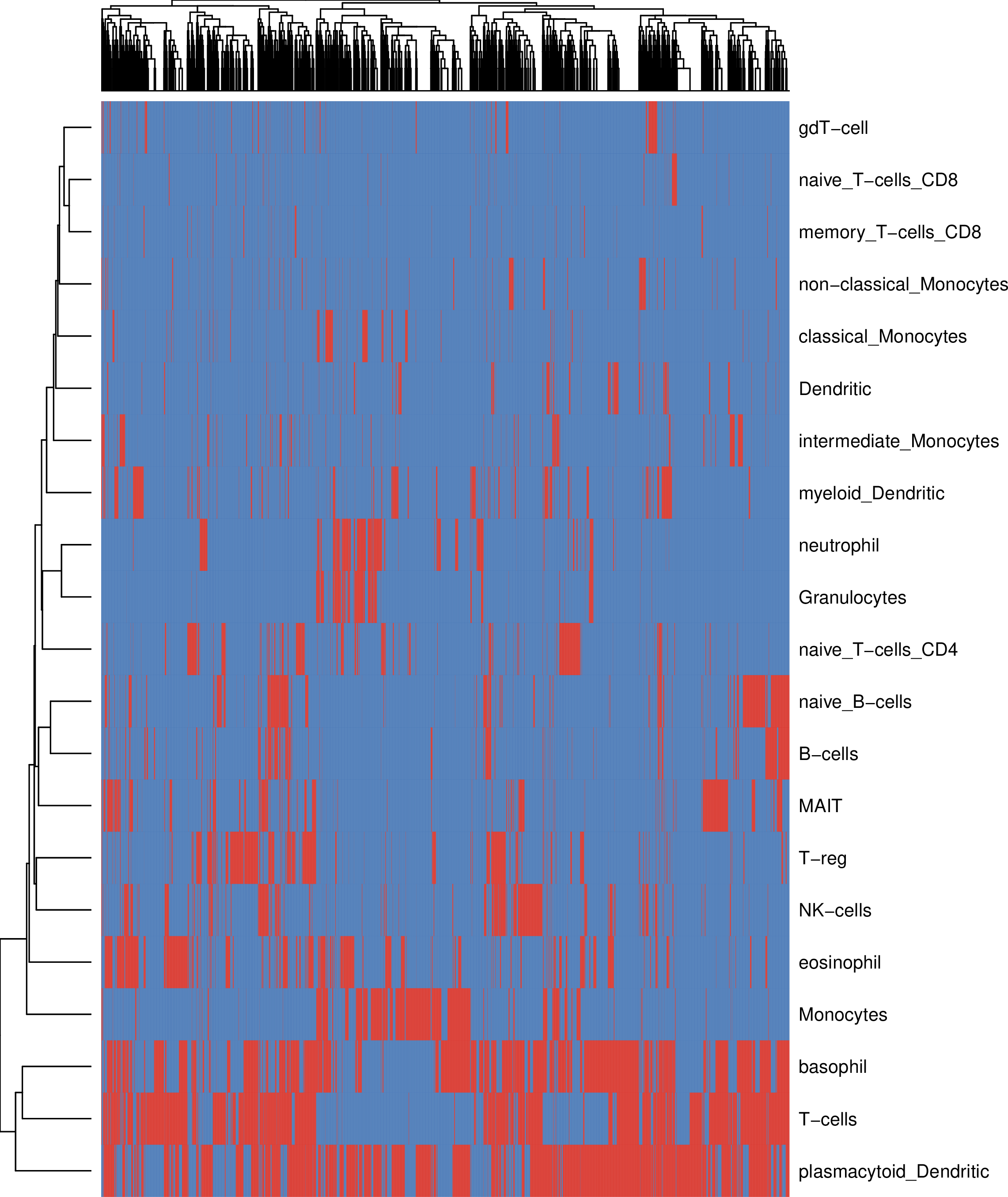

Supplement: Supplementary file 2 — Additional file 2: Supplementary file 2. To demonstrate the utility of scQCEA, we apply the workflow to the sixteen gene expression profiles of eight patients with metastatic melanoma, prepared from pre- and post-treatment experimental batches. You can find the QC interactive report at: https://github.com/isarnassiri/scQCEA/tree/Example-of-Application. Download and unzip the OGC_Interactive_QC_Report_P180121.zip file. You can open CLICK_ME.html file without using rStudio/R. [file 12864_2023_9447_MOESM2_ESM.zip › Inputs/10X-gex/481207_76/P180121-keep_481207_76_Celltype_assignment_HeatMap.png]

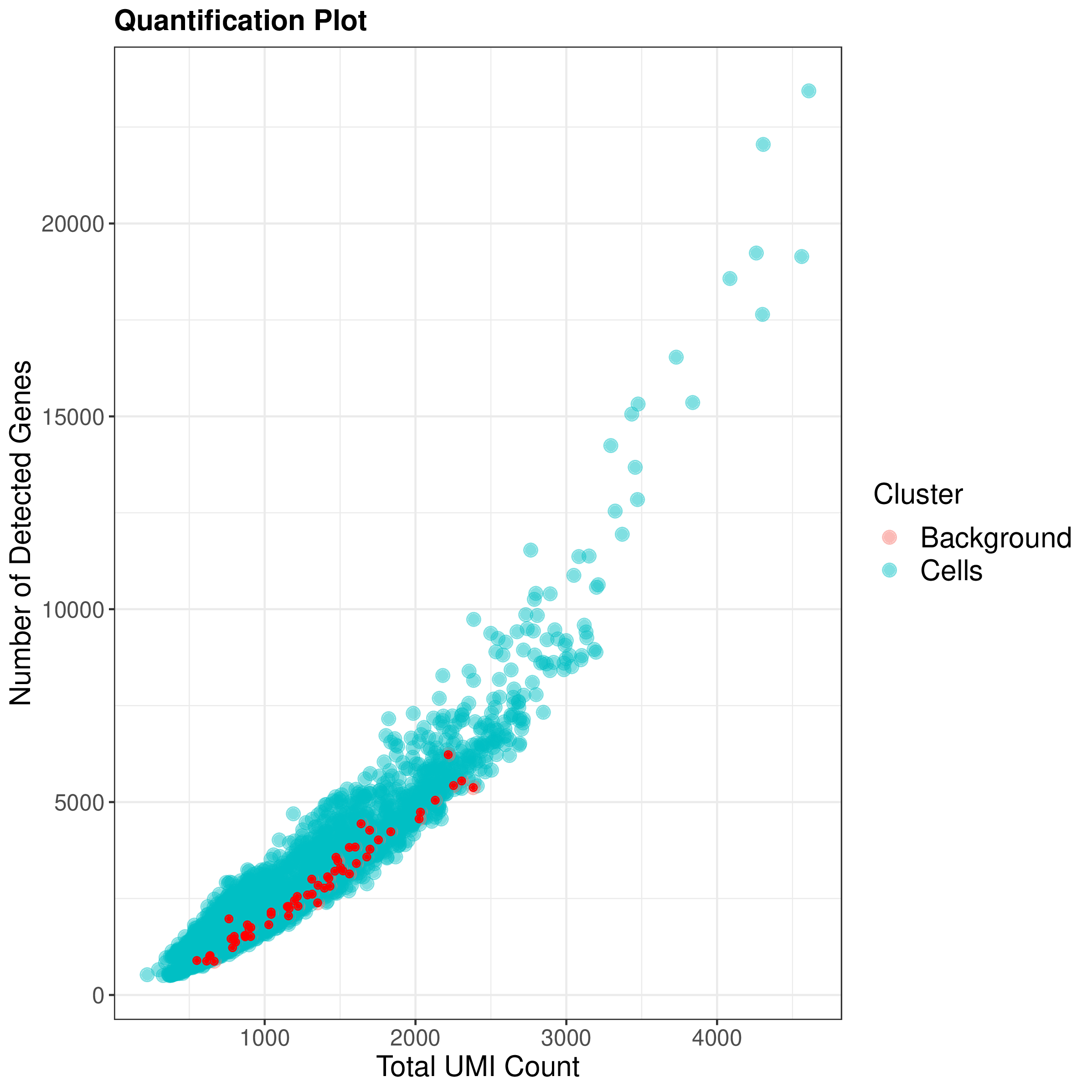

Supplement: Supplementary file 2 — Additional file 2: Supplementary file 2. To demonstrate the utility of scQCEA, we apply the workflow to the sixteen gene expression profiles of eight patients with metastatic melanoma, prepared from pre- and post-treatment experimental batches. You can find the QC interactive report at: https://github.com/isarnassiri/scQCEA/tree/Example-of-Application. Download and unzip the OGC_Interactive_QC_Report_P180121.zip file. You can open CLICK_ME.html file without using rStudio/R. [file 12864_2023_9447_MOESM2_ESM.zip › Inputs/10X-gex/481207_76/P180121-keep_481207_76_TotalUMIvsDetectedGenes.png]

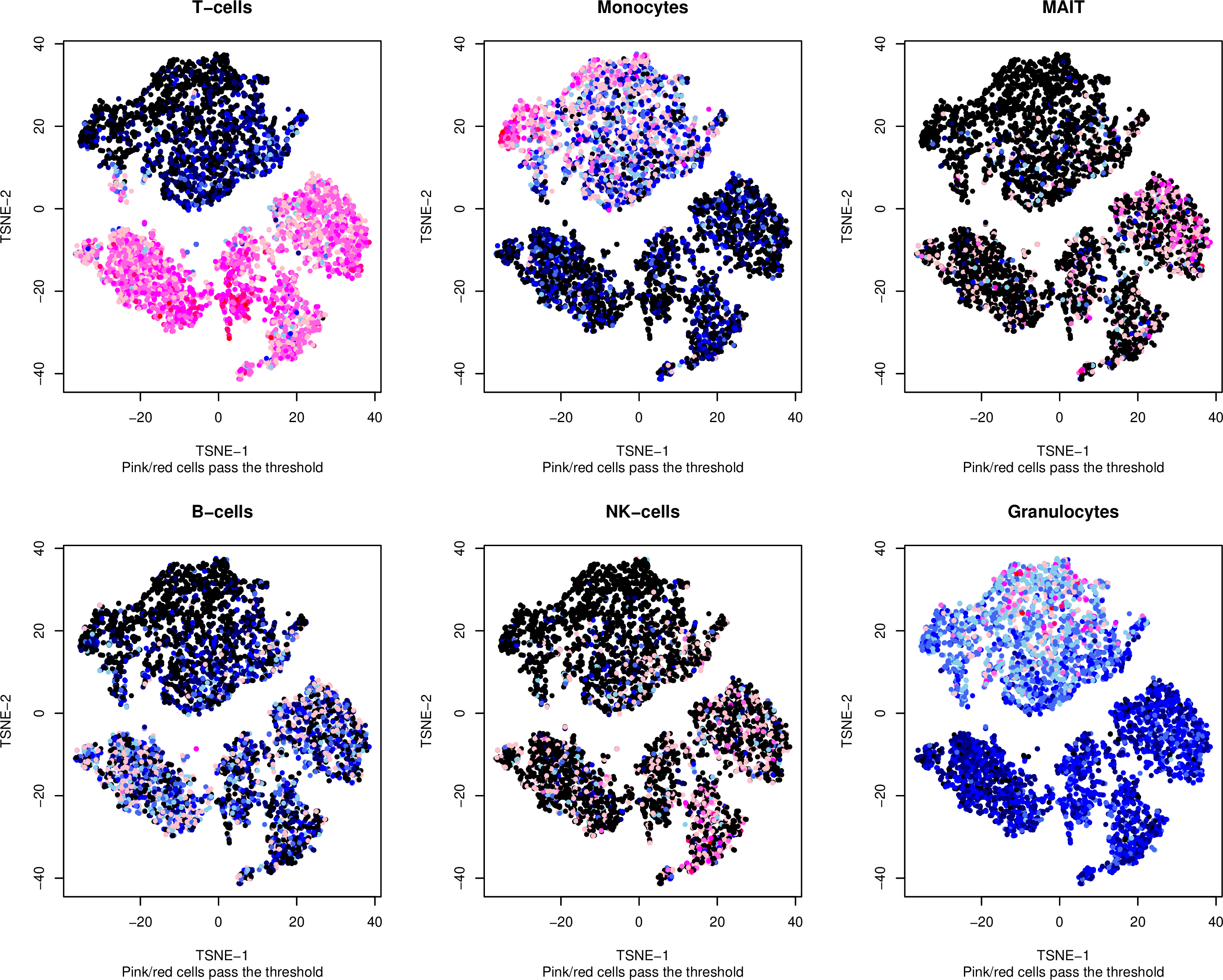

Supplement: Supplementary file 2 — Additional file 2: Supplementary file 2. To demonstrate the utility of scQCEA, we apply the workflow to the sixteen gene expression profiles of eight patients with metastatic melanoma, prepared from pre- and post-treatment experimental batches. You can find the QC interactive report at: https://github.com/isarnassiri/scQCEA/tree/Example-of-Application. Download and unzip the OGC_Interactive_QC_Report_P180121.zip file. You can open CLICK_ME.html file without using rStudio/R. [file 12864_2023_9447_MOESM2_ESM.zip › Inputs/10X-gex/481207_76/P180121-keep_481207_76_tSNE_Plot.png]

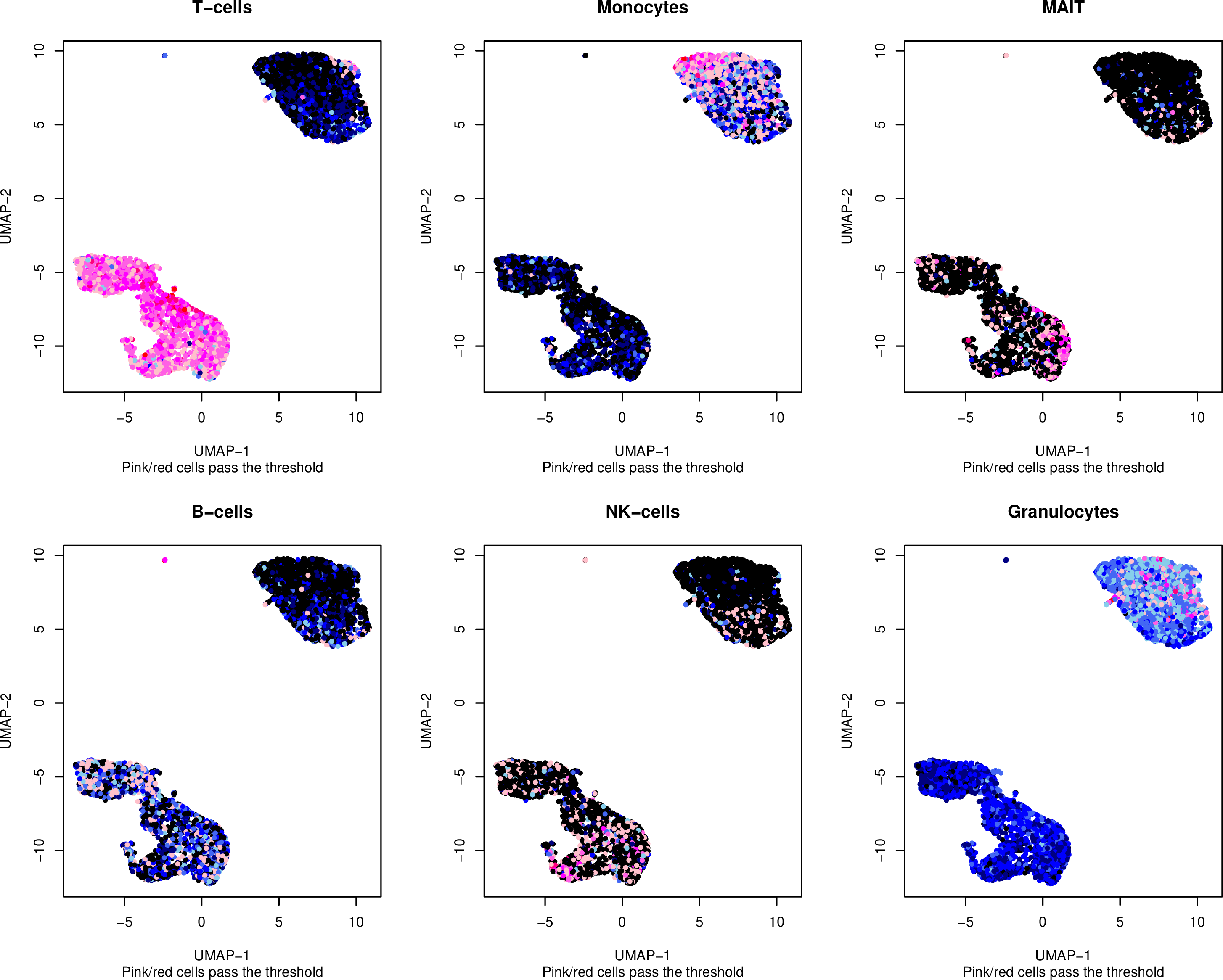

Supplement: Supplementary file 2 — Additional file 2: Supplementary file 2. To demonstrate the utility of scQCEA, we apply the workflow to the sixteen gene expression profiles of eight patients with metastatic melanoma, prepared from pre- and post-treatment experimental batches. You can find the QC interactive report at: https://github.com/isarnassiri/scQCEA/tree/Example-of-Application. Download and unzip the OGC_Interactive_QC_Report_P180121.zip file. You can open CLICK_ME.html file without using rStudio/R. [file 12864_2023_9447_MOESM2_ESM.zip › Inputs/10X-gex/481207_76/P180121-keep_481207_76_UMAP_Plot.png]

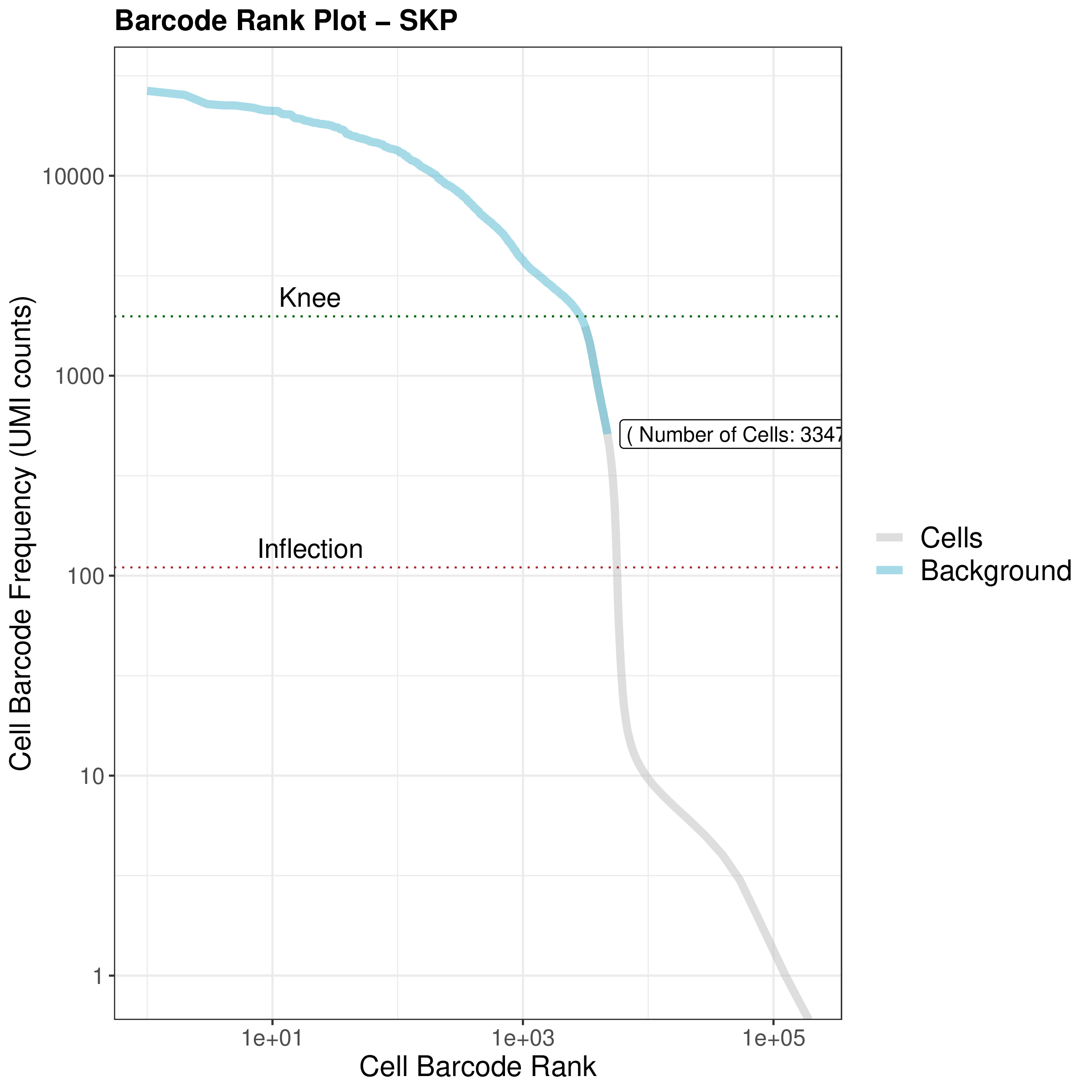

Supplement: Supplementary file 2 — Additional file 2: Supplementary file 2. To demonstrate the utility of scQCEA, we apply the workflow to the sixteen gene expression profiles of eight patients with metastatic melanoma, prepared from pre- and post-treatment experimental batches. You can find the QC interactive report at: https://github.com/isarnassiri/scQCEA/tree/Example-of-Application. Download and unzip the OGC_Interactive_QC_Report_P180121.zip file. You can open CLICK_ME.html file without using rStudio/R. [file 12864_2023_9447_MOESM2_ESM.zip › Inputs/10X-gex/481207_88/P180121-keep_481207_88_BarcodeRankPlot_10X.png]

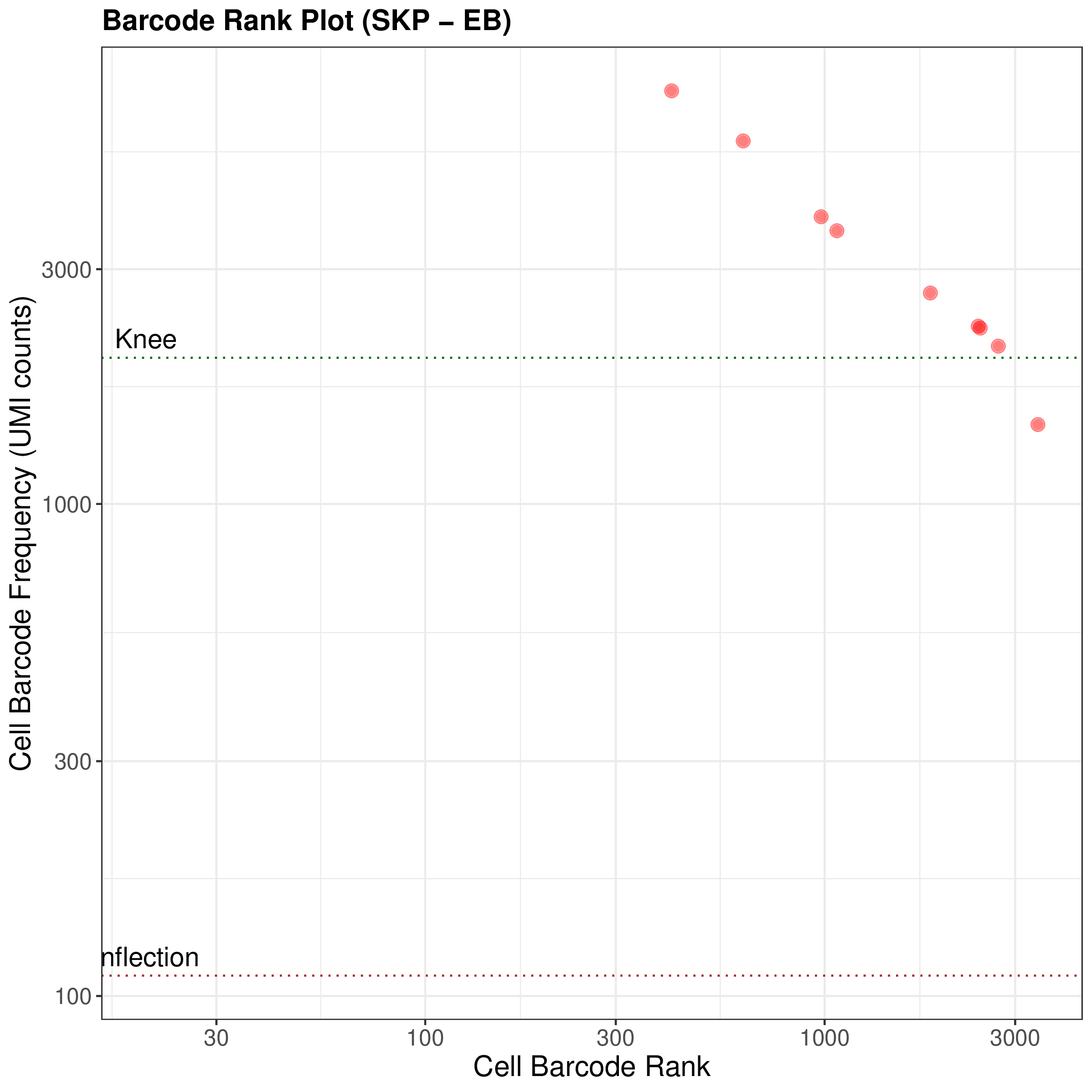

Supplement: Supplementary file 2 — Additional file 2: Supplementary file 2. To demonstrate the utility of scQCEA, we apply the workflow to the sixteen gene expression profiles of eight patients with metastatic melanoma, prepared from pre- and post-treatment experimental batches. You can find the QC interactive report at: https://github.com/isarnassiri/scQCEA/tree/Example-of-Application. Download and unzip the OGC_Interactive_QC_Report_P180121.zip file. You can open CLICK_ME.html file without using rStudio/R. [file 12864_2023_9447_MOESM2_ESM.zip › Inputs/10X-gex/481207_88/P180121-keep_481207_88_BarcodeRankPlot_EB_FilterOut.png]

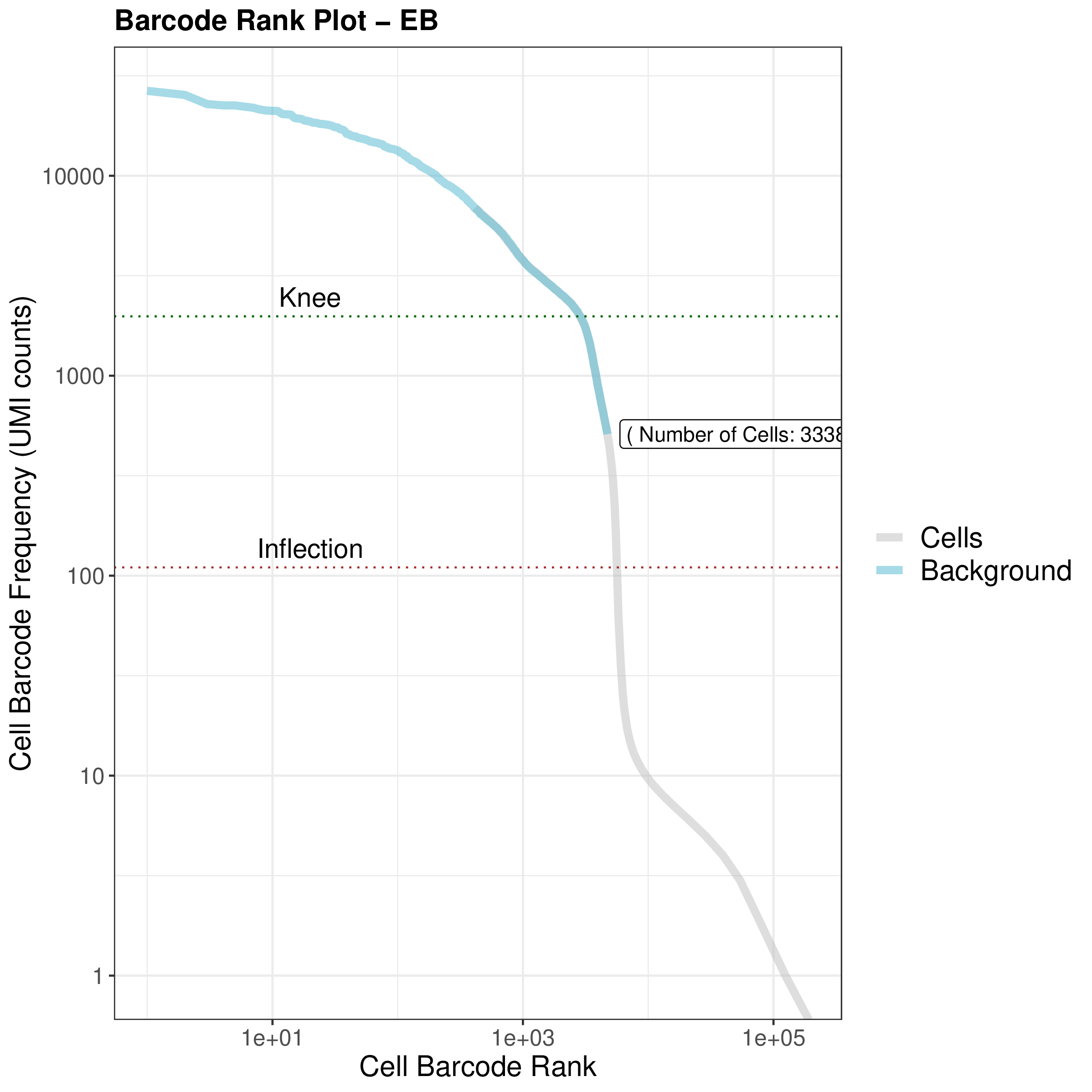

Supplement: Supplementary file 2 — Additional file 2: Supplementary file 2. To demonstrate the utility of scQCEA, we apply the workflow to the sixteen gene expression profiles of eight patients with metastatic melanoma, prepared from pre- and post-treatment experimental batches. You can find the QC interactive report at: https://github.com/isarnassiri/scQCEA/tree/Example-of-Application. Download and unzip the OGC_Interactive_QC_Report_P180121.zip file. You can open CLICK_ME.html file without using rStudio/R. [file 12864_2023_9447_MOESM2_ESM.zip › Inputs/10X-gex/481207_88/P180121-keep_481207_88_BarcodeRankPlot_EB.png]

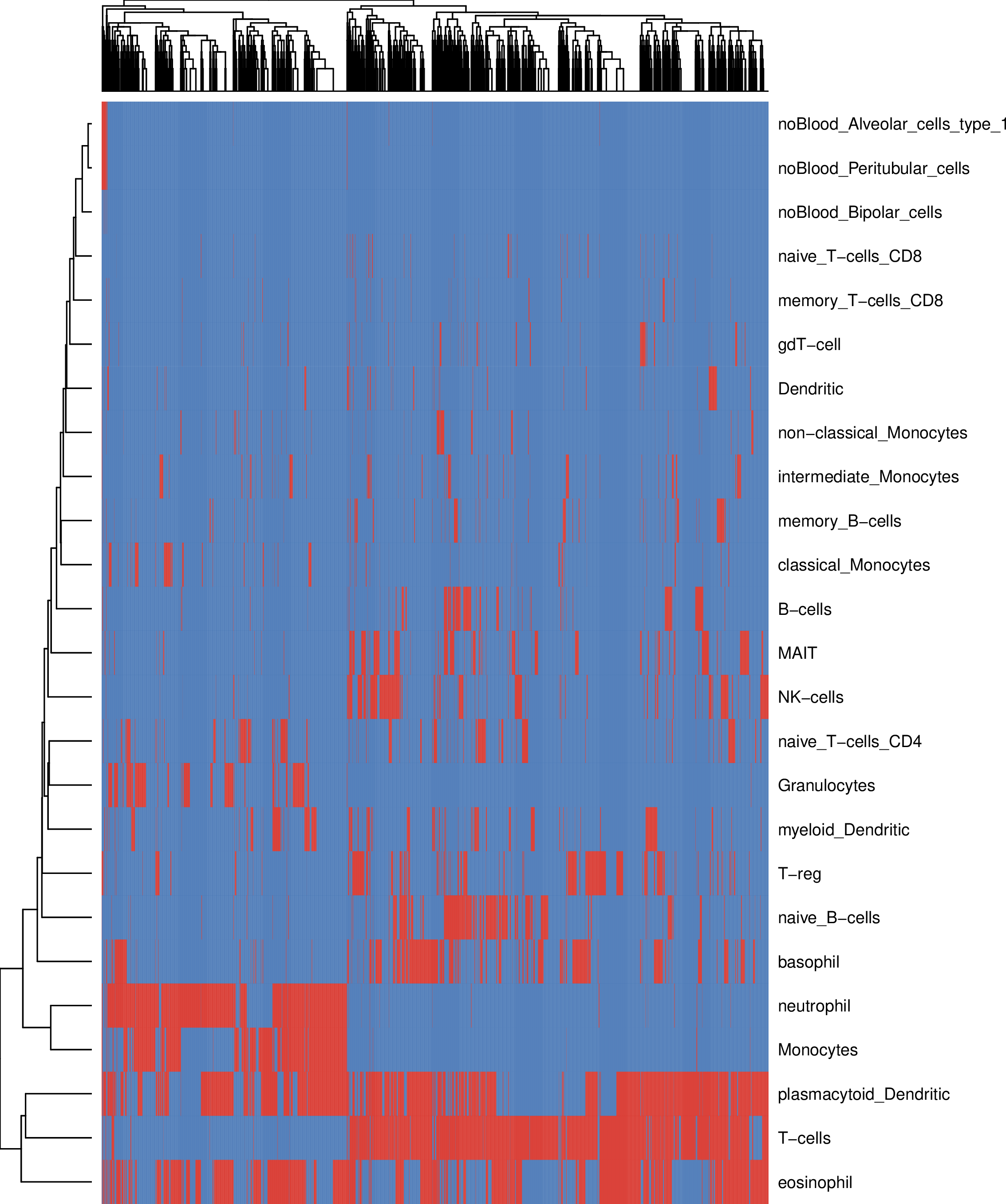

Supplement: Supplementary file 2 — Additional file 2: Supplementary file 2. To demonstrate the utility of scQCEA, we apply the workflow to the sixteen gene expression profiles of eight patients with metastatic melanoma, prepared from pre- and post-treatment experimental batches. You can find the QC interactive report at: https://github.com/isarnassiri/scQCEA/tree/Example-of-Application. Download and unzip the OGC_Interactive_QC_Report_P180121.zip file. You can open CLICK_ME.html file without using rStudio/R. [file 12864_2023_9447_MOESM2_ESM.zip › Inputs/10X-gex/481207_88/P180121-keep_481207_88_Celltype_assignment_HeatMap.png]

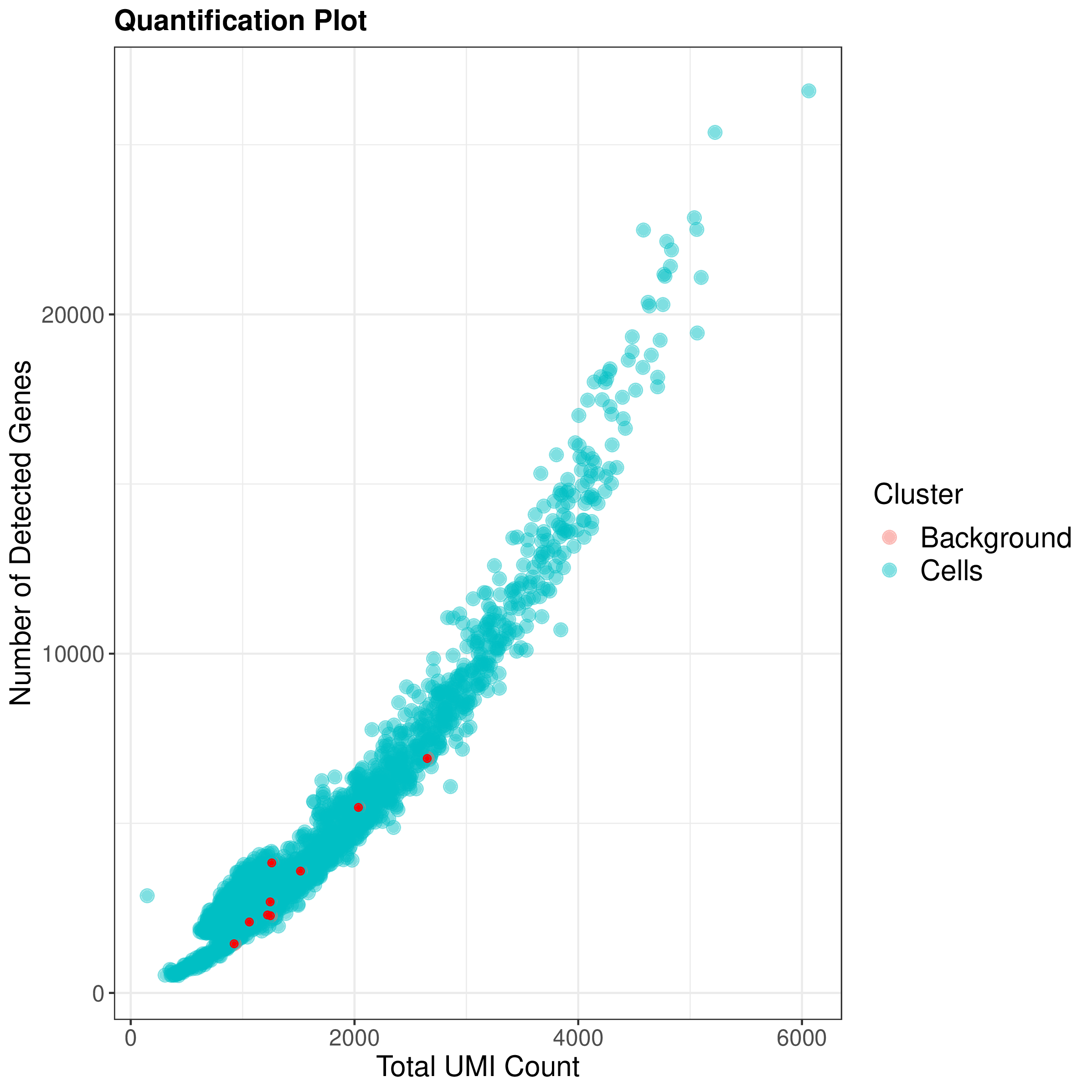

Supplement: Supplementary file 2 — Additional file 2: Supplementary file 2. To demonstrate the utility of scQCEA, we apply the workflow to the sixteen gene expression profiles of eight patients with metastatic melanoma, prepared from pre- and post-treatment experimental batches. You can find the QC interactive report at: https://github.com/isarnassiri/scQCEA/tree/Example-of-Application. Download and unzip the OGC_Interactive_QC_Report_P180121.zip file. You can open CLICK_ME.html file without using rStudio/R. [file 12864_2023_9447_MOESM2_ESM.zip › Inputs/10X-gex/481207_88/P180121-keep_481207_88_TotalUMIvsDetectedGenes.png]

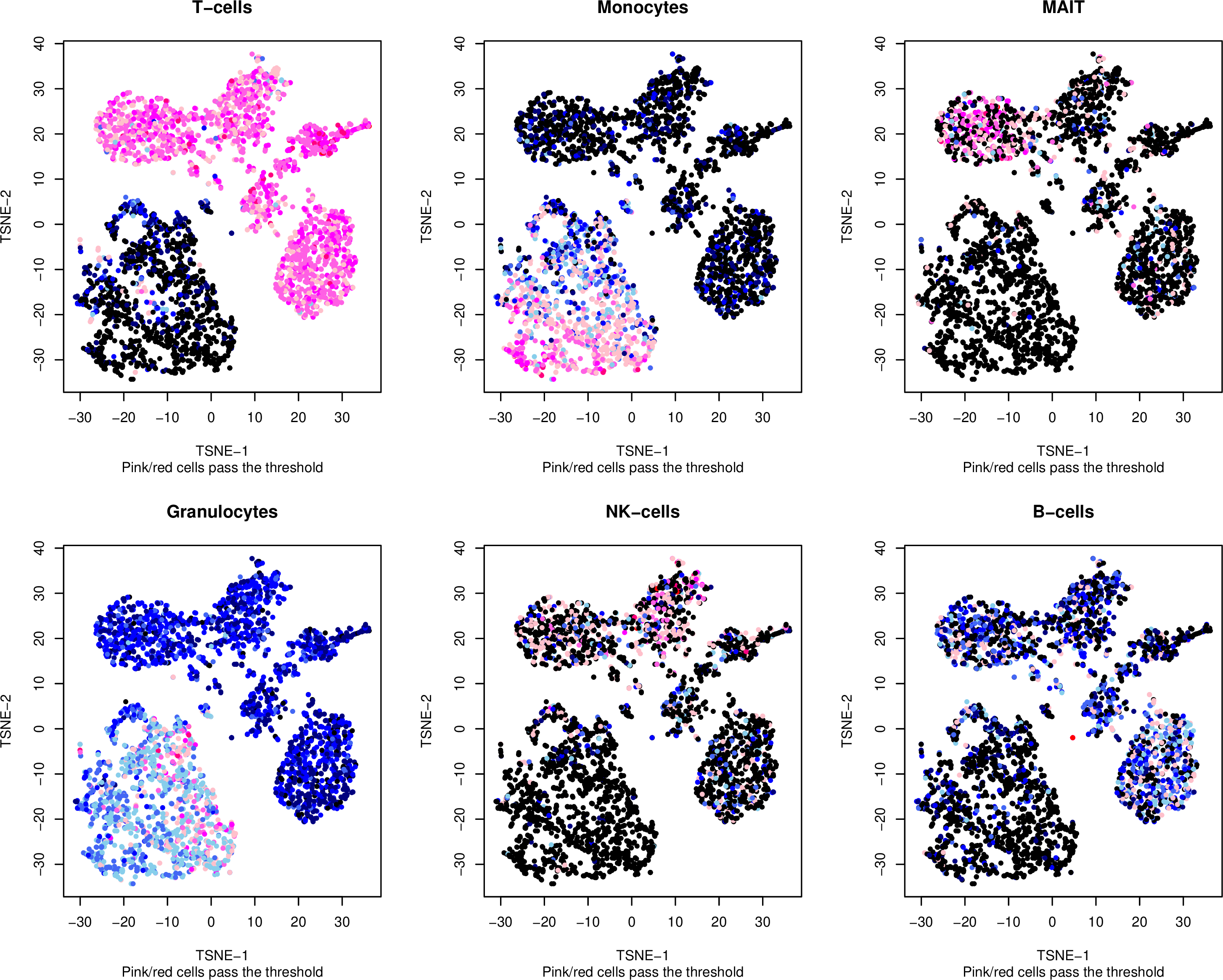

Supplement: Supplementary file 2 — Additional file 2: Supplementary file 2. To demonstrate the utility of scQCEA, we apply the workflow to the sixteen gene expression profiles of eight patients with metastatic melanoma, prepared from pre- and post-treatment experimental batches. You can find the QC interactive report at: https://github.com/isarnassiri/scQCEA/tree/Example-of-Application. Download and unzip the OGC_Interactive_QC_Report_P180121.zip file. You can open CLICK_ME.html file without using rStudio/R. [file 12864_2023_9447_MOESM2_ESM.zip › Inputs/10X-gex/481207_88/P180121-keep_481207_88_tSNE_Plot.png]

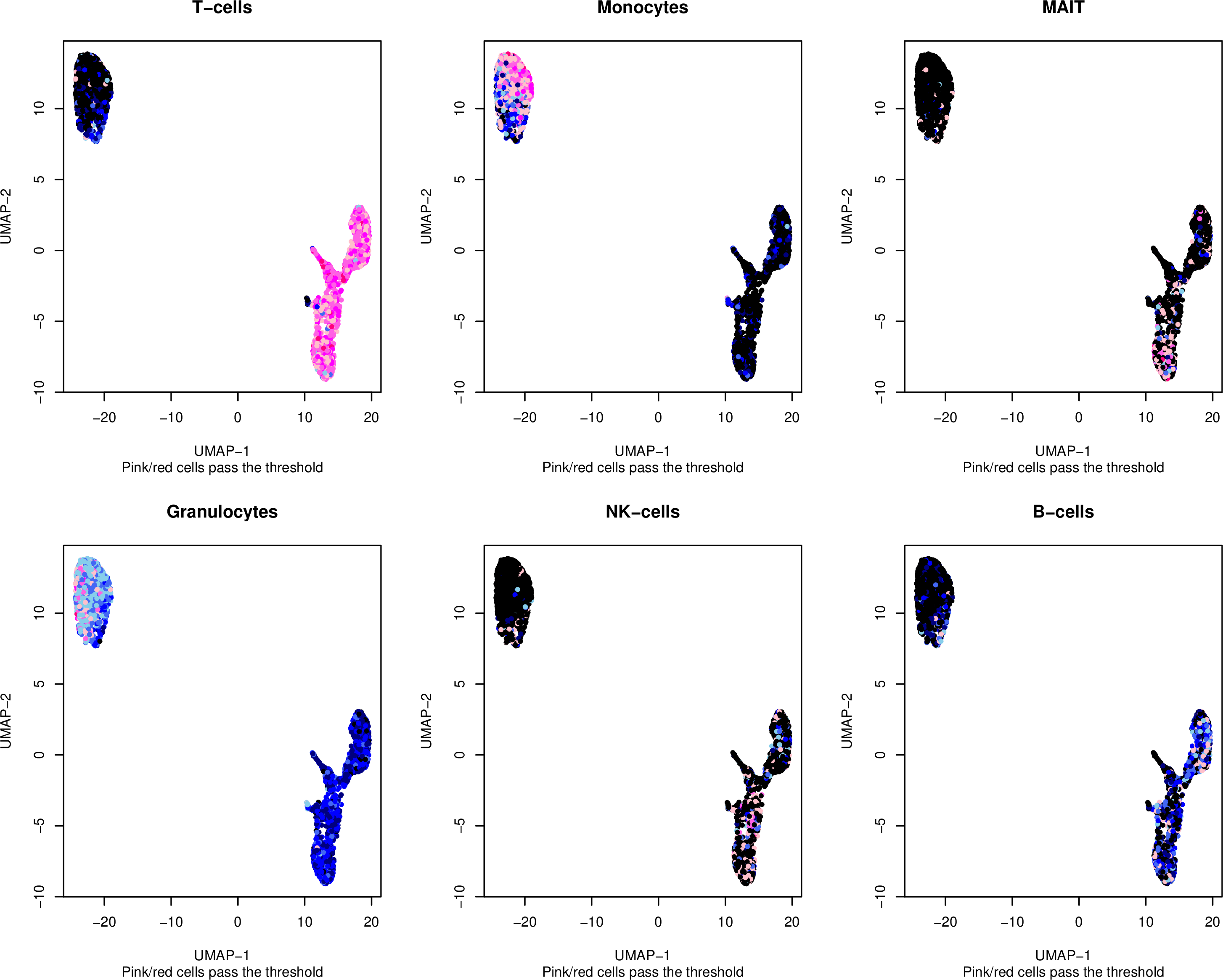

Supplement: Supplementary file 2 — Additional file 2: Supplementary file 2. To demonstrate the utility of scQCEA, we apply the workflow to the sixteen gene expression profiles of eight patients with metastatic melanoma, prepared from pre- and post-treatment experimental batches. You can find the QC interactive report at: https://github.com/isarnassiri/scQCEA/tree/Example-of-Application. Download and unzip the OGC_Interactive_QC_Report_P180121.zip file. You can open CLICK_ME.html file without using rStudio/R. [file 12864_2023_9447_MOESM2_ESM.zip › Inputs/10X-gex/481207_88/P180121-keep_481207_88_UMAP_Plot.png]

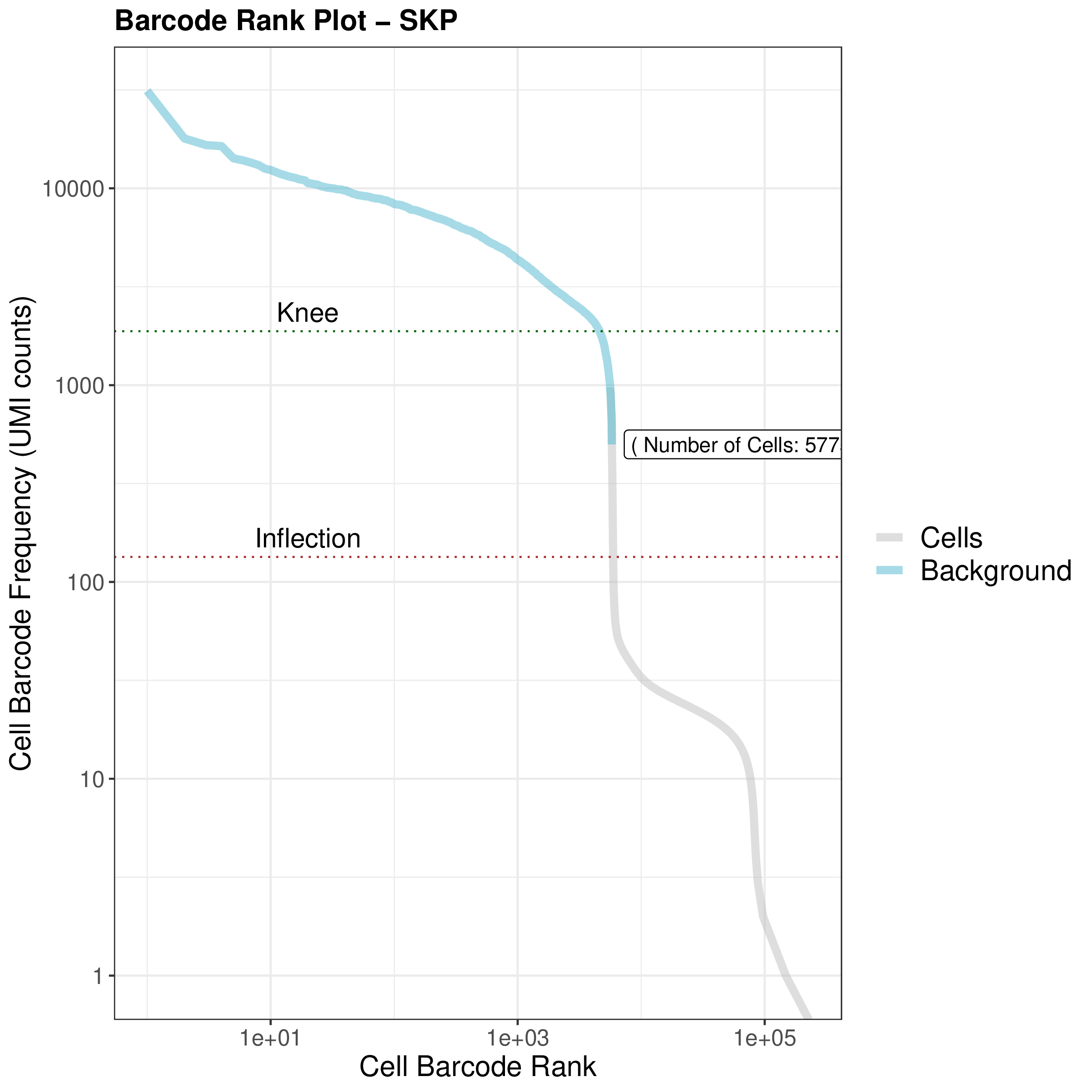

Supplement: Supplementary file 2 — Additional file 2: Supplementary file 2. To demonstrate the utility of scQCEA, we apply the workflow to the sixteen gene expression profiles of eight patients with metastatic melanoma, prepared from pre- and post-treatment experimental batches. You can find the QC interactive report at: https://github.com/isarnassiri/scQCEA/tree/Example-of-Application. Download and unzip the OGC_Interactive_QC_Report_P180121.zip file. You can open CLICK_ME.html file without using rStudio/R. [file 12864_2023_9447_MOESM2_ESM.zip › Inputs/10X-gex/500667_03/P180121-keep_500667_03_BarcodeRankPlot_10X.png]

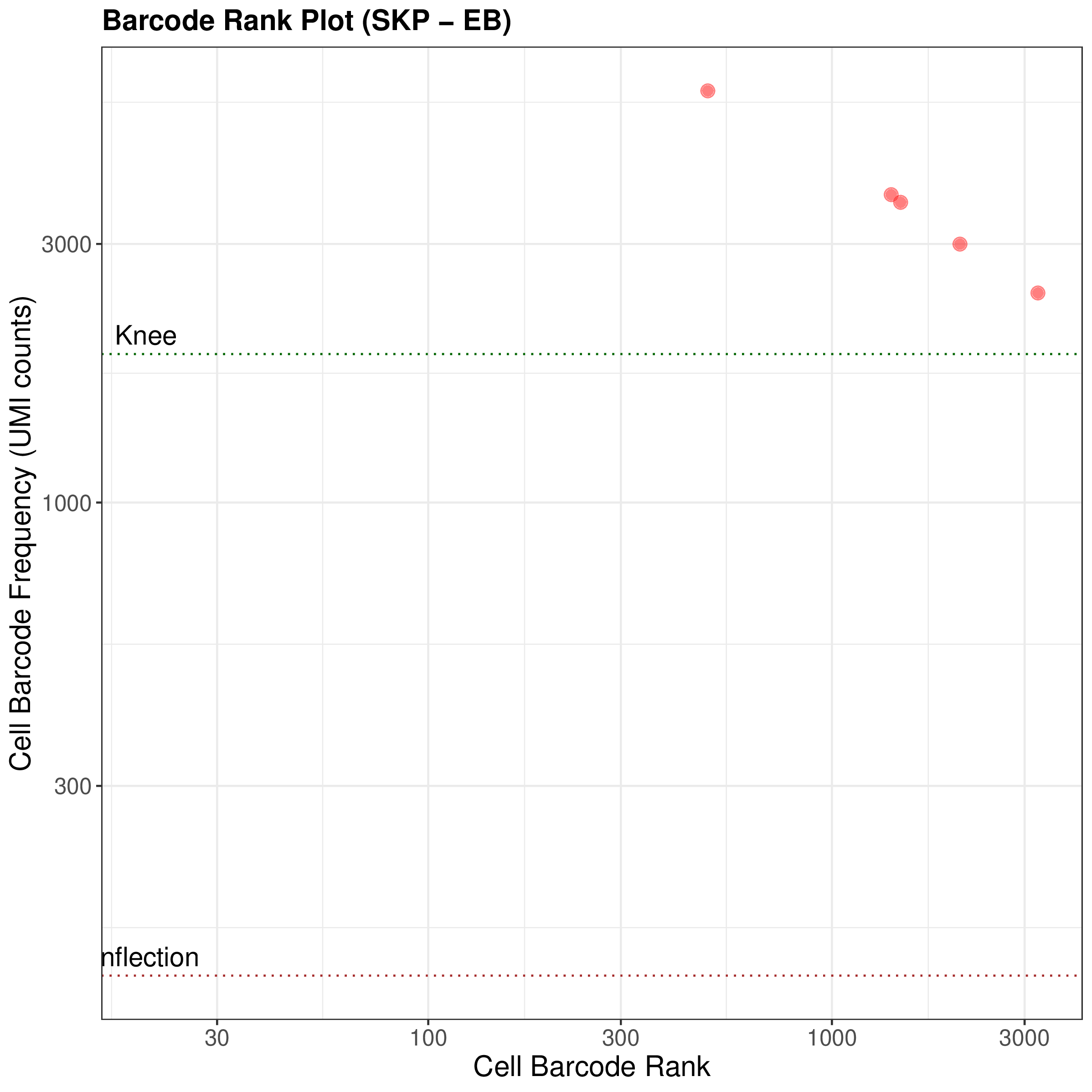

Supplement: Supplementary file 2 — Additional file 2: Supplementary file 2. To demonstrate the utility of scQCEA, we apply the workflow to the sixteen gene expression profiles of eight patients with metastatic melanoma, prepared from pre- and post-treatment experimental batches. You can find the QC interactive report at: https://github.com/isarnassiri/scQCEA/tree/Example-of-Application. Download and unzip the OGC_Interactive_QC_Report_P180121.zip file. You can open CLICK_ME.html file without using rStudio/R. [file 12864_2023_9447_MOESM2_ESM.zip › Inputs/10X-gex/500667_03/P180121-keep_500667_03_BarcodeRankPlot_EB_FilterOut.png]

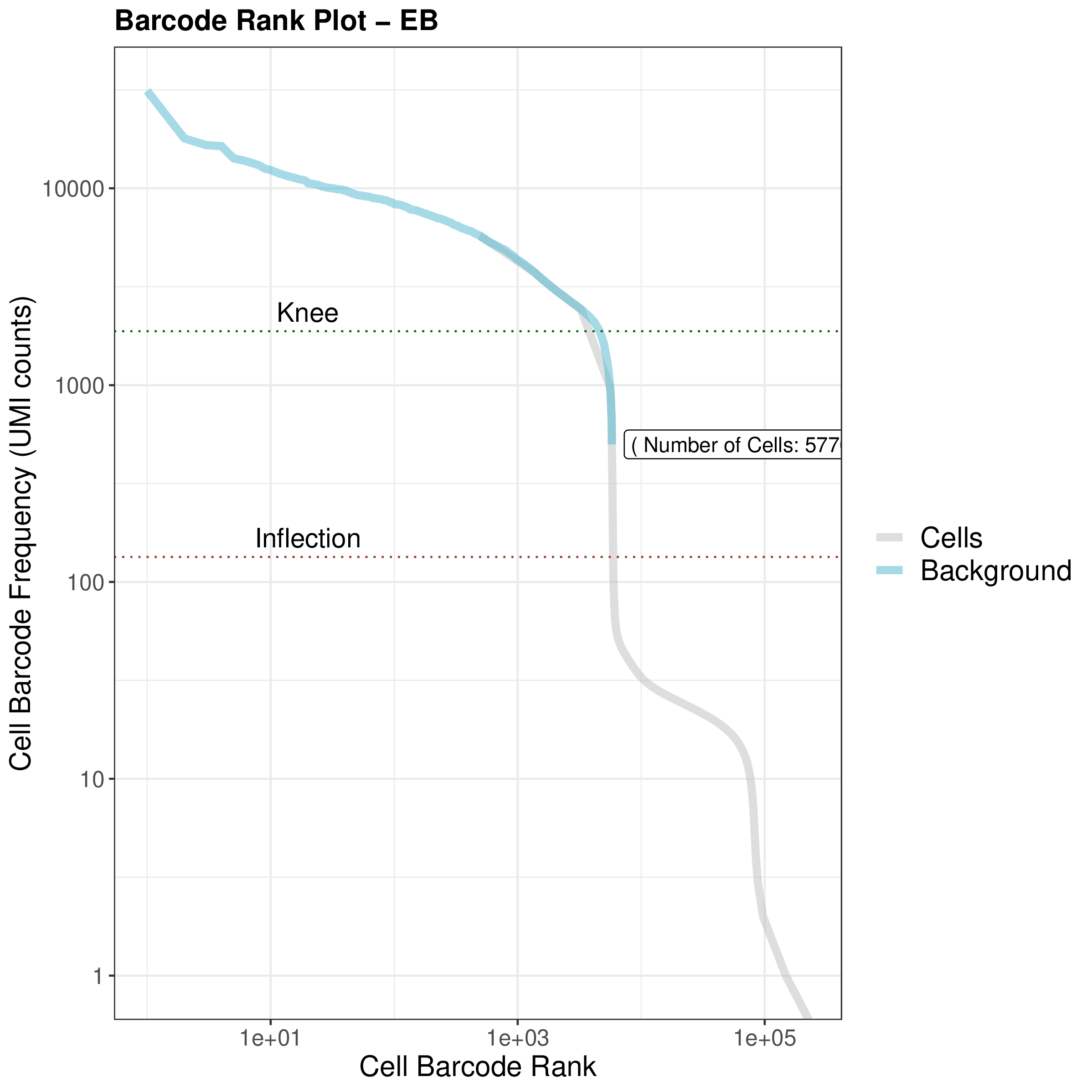

Supplement: Supplementary file 2 — Additional file 2: Supplementary file 2. To demonstrate the utility of scQCEA, we apply the workflow to the sixteen gene expression profiles of eight patients with metastatic melanoma, prepared from pre- and post-treatment experimental batches. You can find the QC interactive report at: https://github.com/isarnassiri/scQCEA/tree/Example-of-Application. Download and unzip the OGC_Interactive_QC_Report_P180121.zip file. You can open CLICK_ME.html file without using rStudio/R. [file 12864_2023_9447_MOESM2_ESM.zip › Inputs/10X-gex/500667_03/P180121-keep_500667_03_BarcodeRankPlot_EB.png]

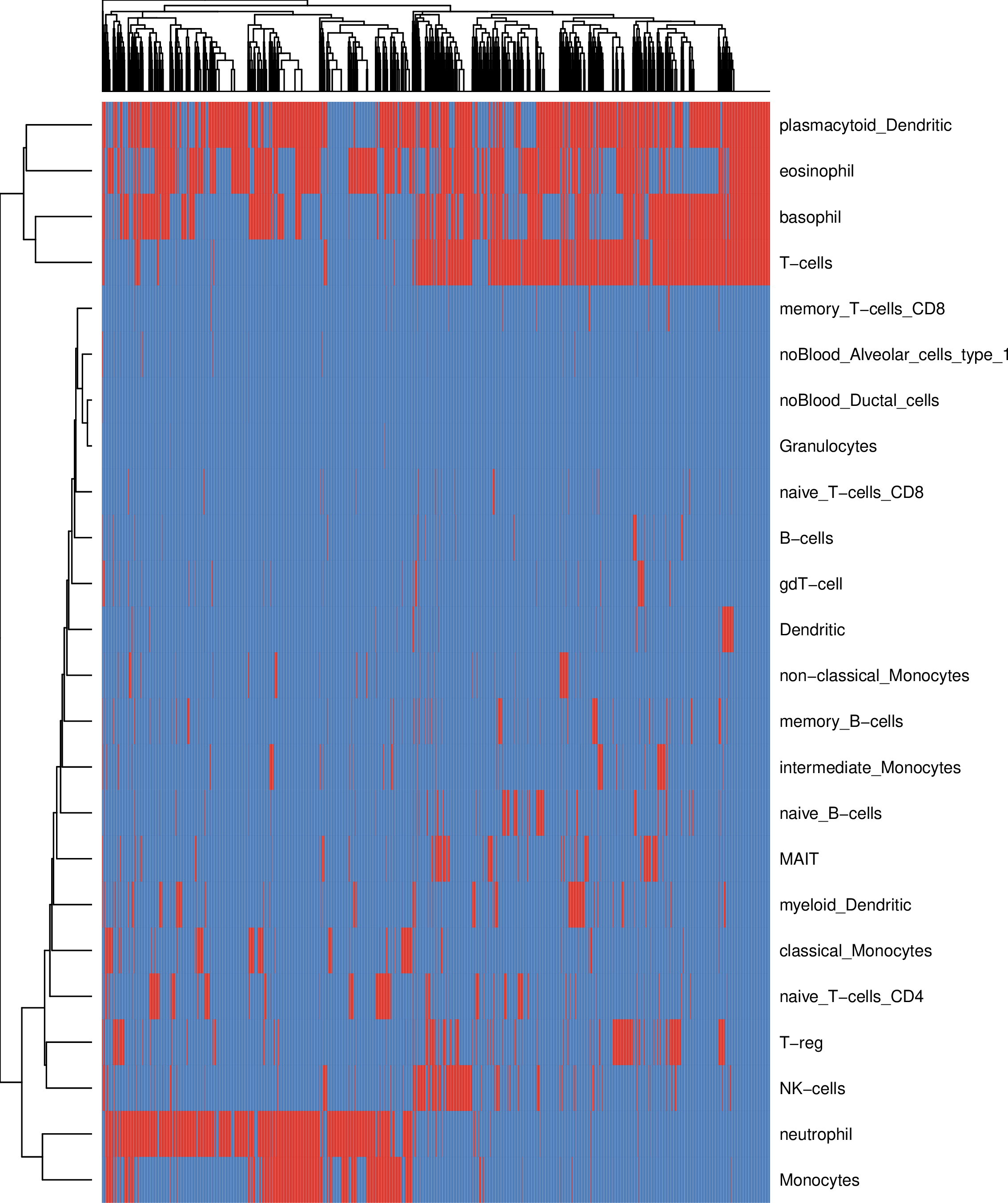

Supplement: Supplementary file 2 — Additional file 2: Supplementary file 2. To demonstrate the utility of scQCEA, we apply the workflow to the sixteen gene expression profiles of eight patients with metastatic melanoma, prepared from pre- and post-treatment experimental batches. You can find the QC interactive report at: https://github.com/isarnassiri/scQCEA/tree/Example-of-Application. Download and unzip the OGC_Interactive_QC_Report_P180121.zip file. You can open CLICK_ME.html file without using rStudio/R. [file 12864_2023_9447_MOESM2_ESM.zip › Inputs/10X-gex/500667_03/P180121-keep_500667_03_Celltype_assignment_HeatMap.png]

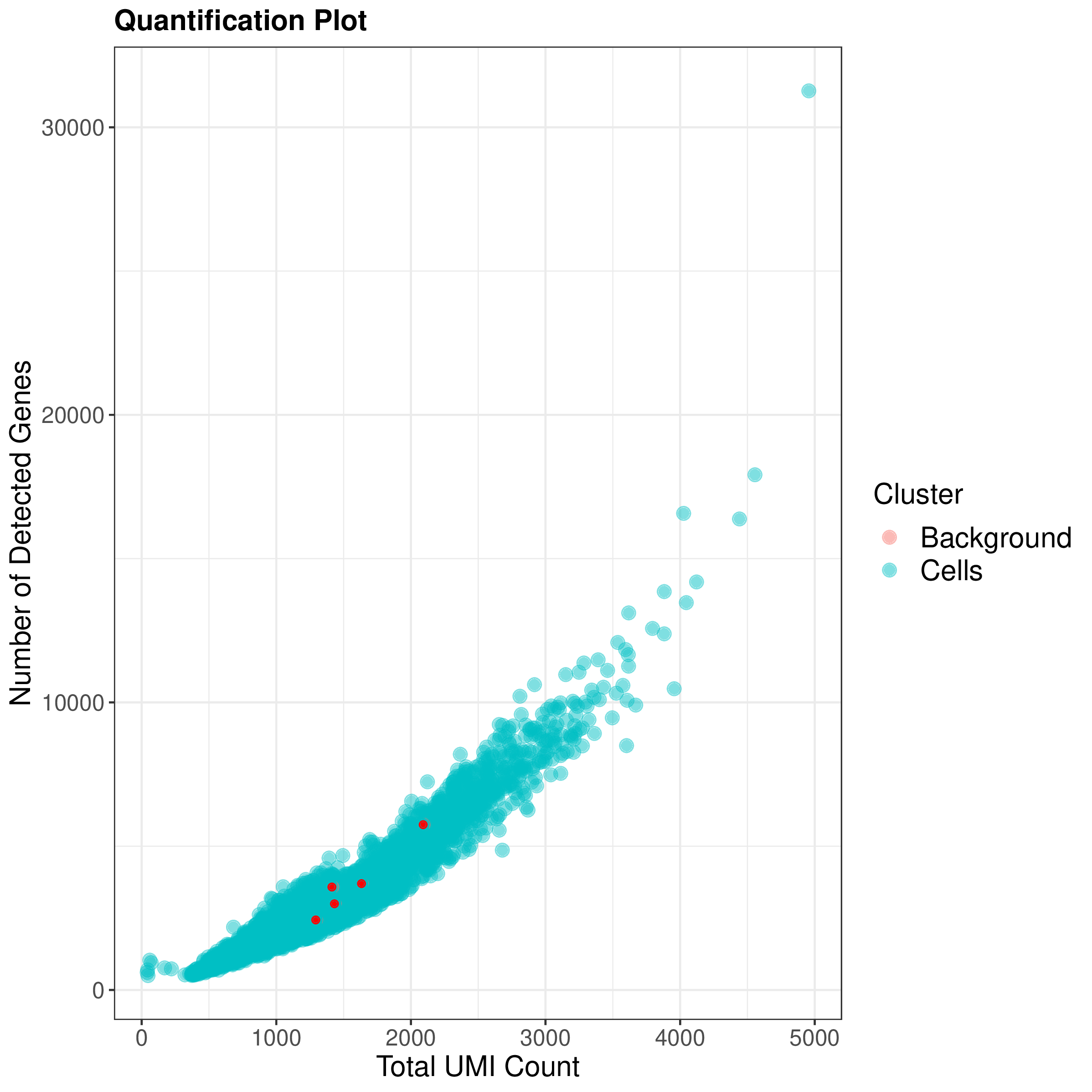

Supplement: Supplementary file 2 — Additional file 2: Supplementary file 2. To demonstrate the utility of scQCEA, we apply the workflow to the sixteen gene expression profiles of eight patients with metastatic melanoma, prepared from pre- and post-treatment experimental batches. You can find the QC interactive report at: https://github.com/isarnassiri/scQCEA/tree/Example-of-Application. Download and unzip the OGC_Interactive_QC_Report_P180121.zip file. You can open CLICK_ME.html file without using rStudio/R. [file 12864_2023_9447_MOESM2_ESM.zip › Inputs/10X-gex/500667_03/P180121-keep_500667_03_TotalUMIvsDetectedGenes.png]

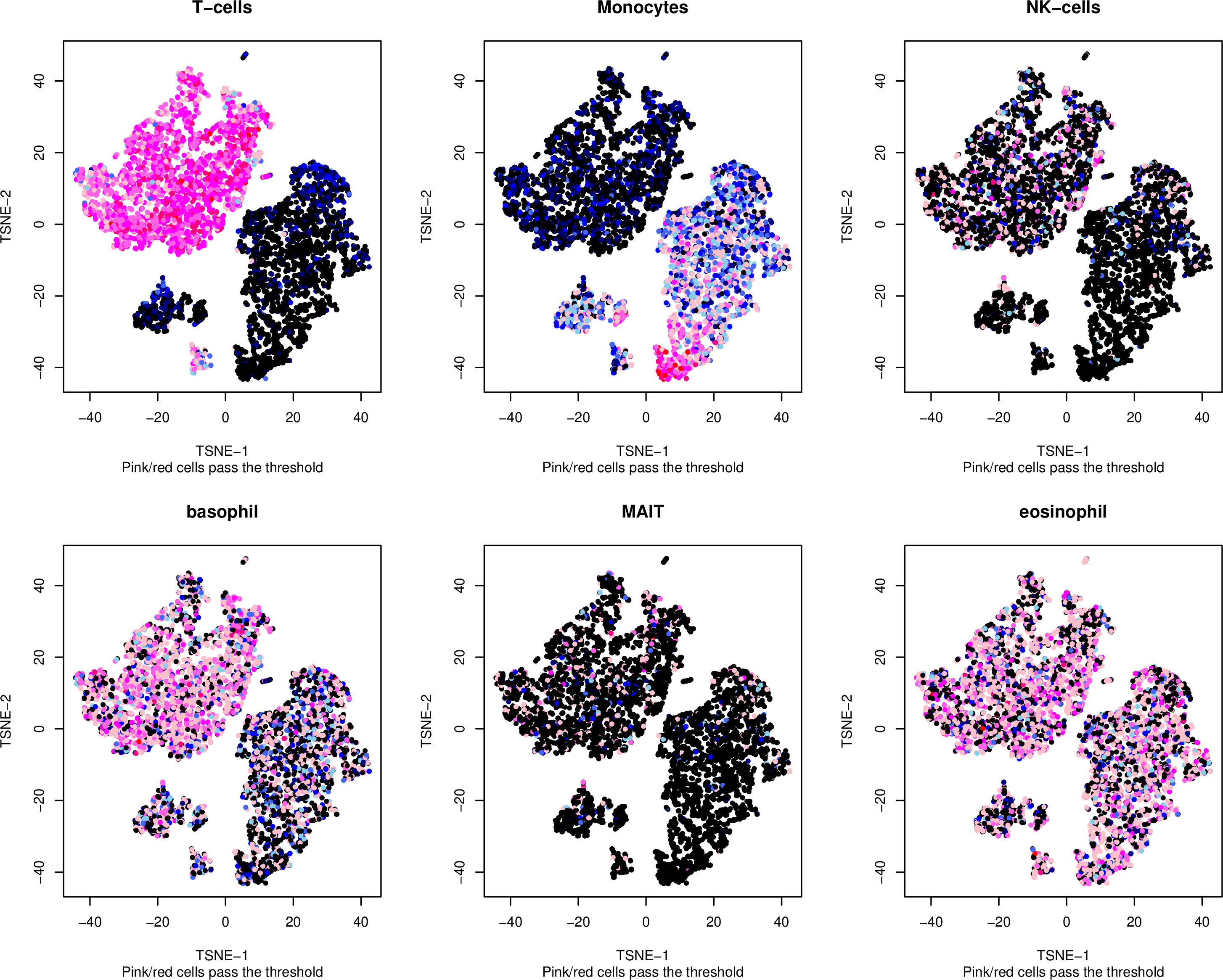

Supplement: Supplementary file 2 — Additional file 2: Supplementary file 2. To demonstrate the utility of scQCEA, we apply the workflow to the sixteen gene expression profiles of eight patients with metastatic melanoma, prepared from pre- and post-treatment experimental batches. You can find the QC interactive report at: https://github.com/isarnassiri/scQCEA/tree/Example-of-Application. Download and unzip the OGC_Interactive_QC_Report_P180121.zip file. You can open CLICK_ME.html file without using rStudio/R. [file 12864_2023_9447_MOESM2_ESM.zip › Inputs/10X-gex/500667_03/P180121-keep_500667_03_tSNE_Plot.png]

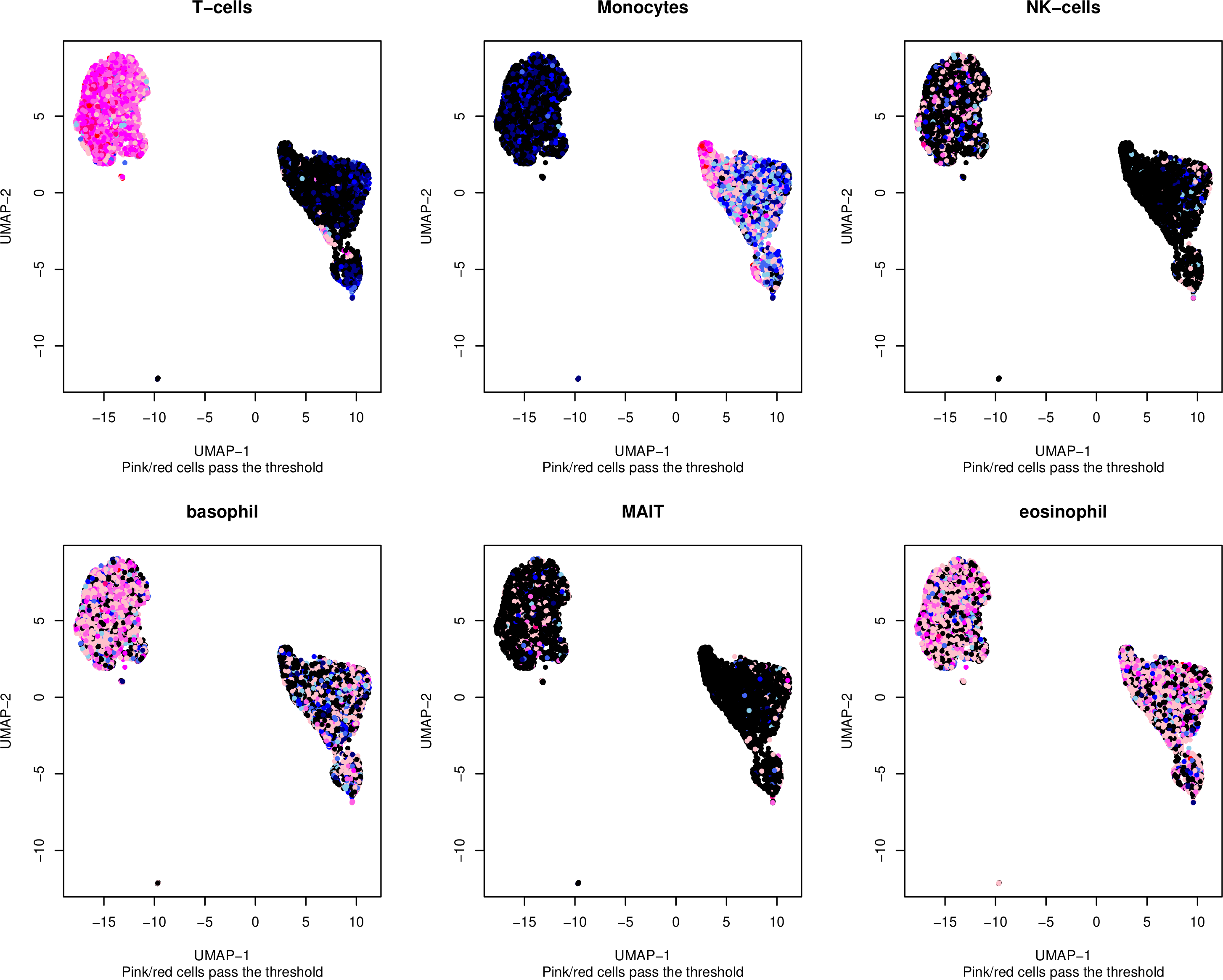

Supplement: Supplementary file 2 — Additional file 2: Supplementary file 2. To demonstrate the utility of scQCEA, we apply the workflow to the sixteen gene expression profiles of eight patients with metastatic melanoma, prepared from pre- and post-treatment experimental batches. You can find the QC interactive report at: https://github.com/isarnassiri/scQCEA/tree/Example-of-Application. Download and unzip the OGC_Interactive_QC_Report_P180121.zip file. You can open CLICK_ME.html file without using rStudio/R. [file 12864_2023_9447_MOESM2_ESM.zip › Inputs/10X-gex/500667_03/P180121-keep_500667_03_UMAP_Plot.png]

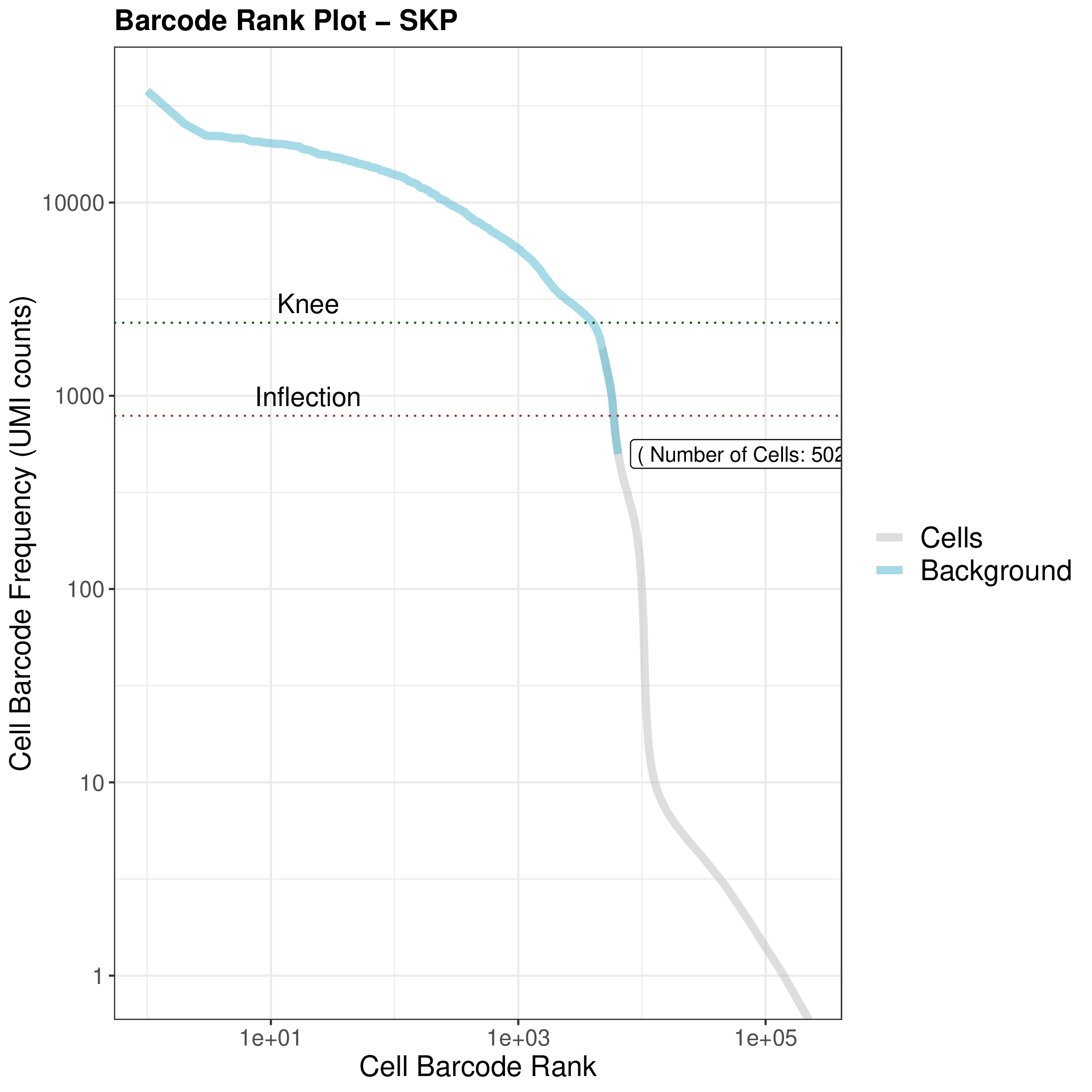

Supplement: Supplementary file 2 — Additional file 2: Supplementary file 2. To demonstrate the utility of scQCEA, we apply the workflow to the sixteen gene expression profiles of eight patients with metastatic melanoma, prepared from pre- and post-treatment experimental batches. You can find the QC interactive report at: https://github.com/isarnassiri/scQCEA/tree/Example-of-Application. Download and unzip the OGC_Interactive_QC_Report_P180121.zip file. You can open CLICK_ME.html file without using rStudio/R. [file 12864_2023_9447_MOESM2_ESM.zip › Inputs/10X-gex/500667_15/P180121-keep_500667_15_BarcodeRankPlot_10X.png]

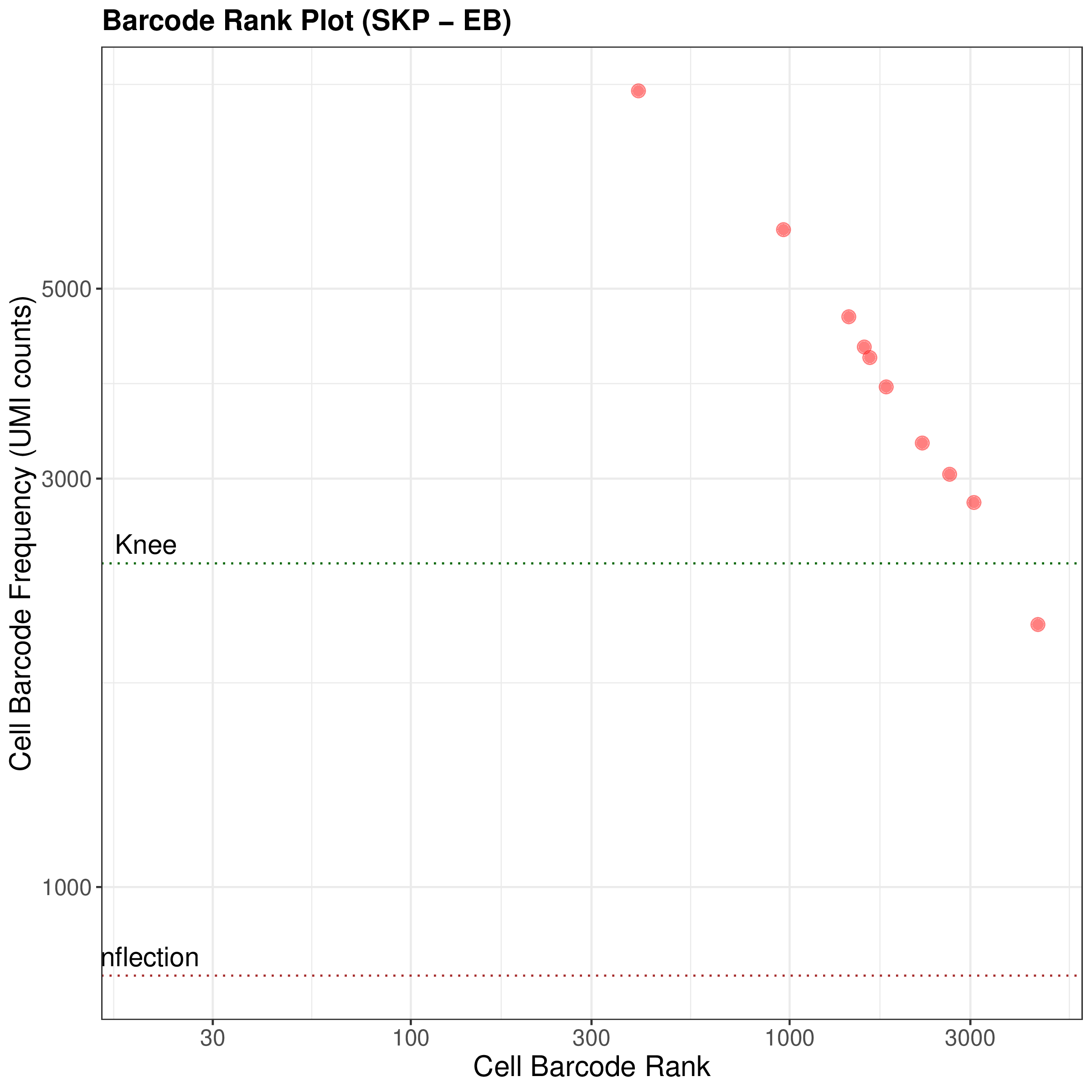

Supplement: Supplementary file 2 — Additional file 2: Supplementary file 2. To demonstrate the utility of scQCEA, we apply the workflow to the sixteen gene expression profiles of eight patients with metastatic melanoma, prepared from pre- and post-treatment experimental batches. You can find the QC interactive report at: https://github.com/isarnassiri/scQCEA/tree/Example-of-Application. Download and unzip the OGC_Interactive_QC_Report_P180121.zip file. You can open CLICK_ME.html file without using rStudio/R. [file 12864_2023_9447_MOESM2_ESM.zip › Inputs/10X-gex/500667_15/P180121-keep_500667_15_BarcodeRankPlot_EB_FilterOut.png]

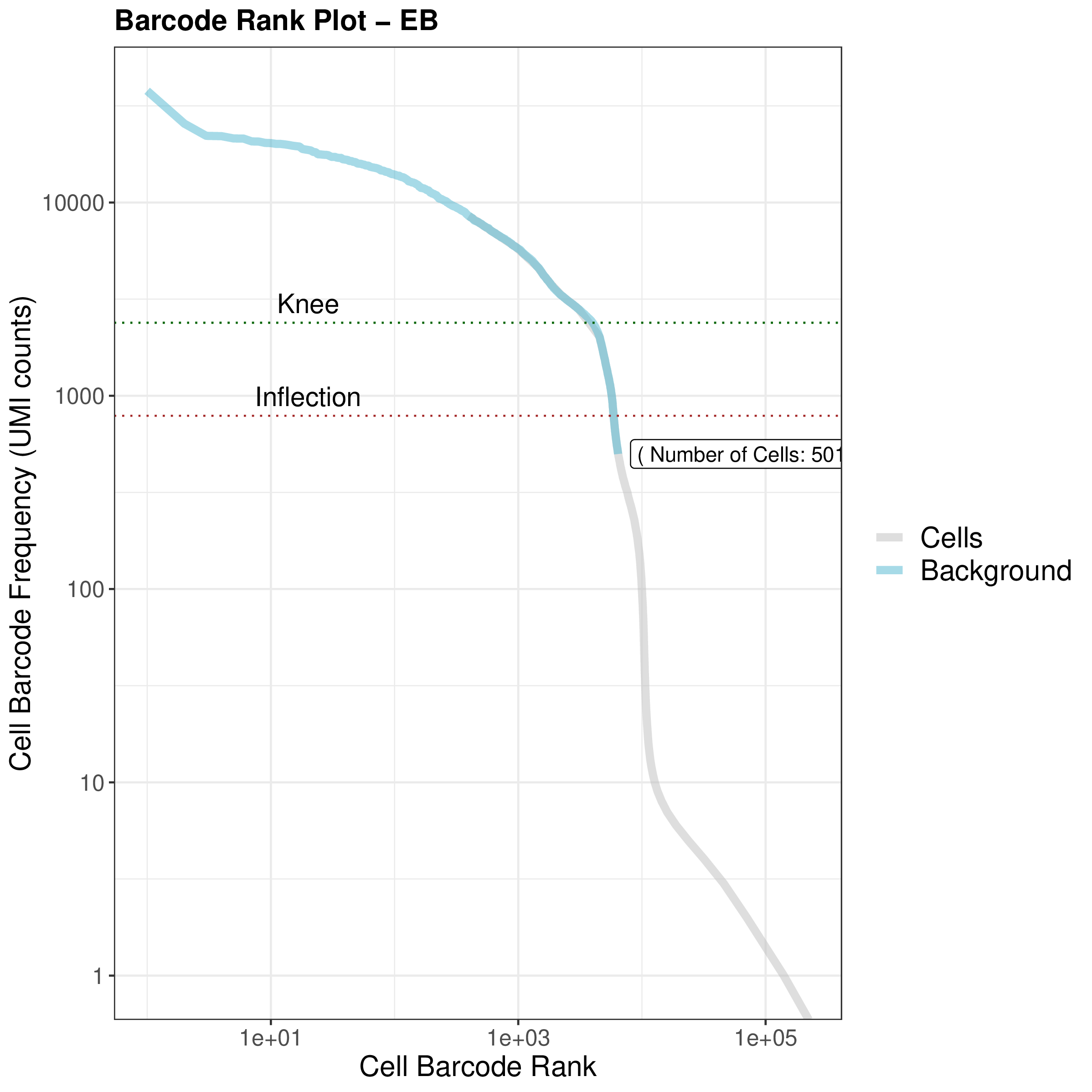

Supplement: Supplementary file 2 — Additional file 2: Supplementary file 2. To demonstrate the utility of scQCEA, we apply the workflow to the sixteen gene expression profiles of eight patients with metastatic melanoma, prepared from pre- and post-treatment experimental batches. You can find the QC interactive report at: https://github.com/isarnassiri/scQCEA/tree/Example-of-Application. Download and unzip the OGC_Interactive_QC_Report_P180121.zip file. You can open CLICK_ME.html file without using rStudio/R. [file 12864_2023_9447_MOESM2_ESM.zip › Inputs/10X-gex/500667_15/P180121-keep_500667_15_BarcodeRankPlot_EB.png]

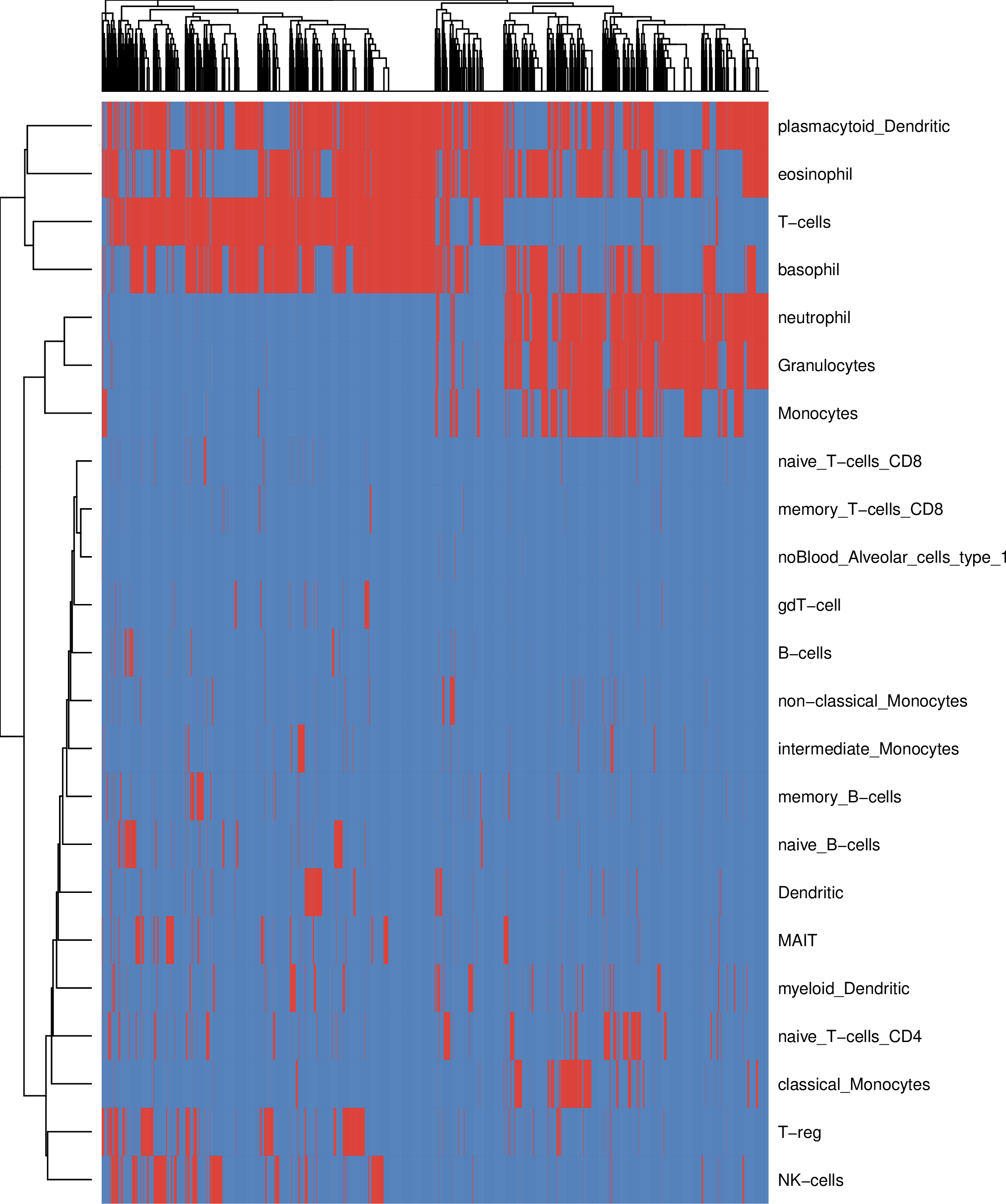

Supplement: Supplementary file 2 — Additional file 2: Supplementary file 2. To demonstrate the utility of scQCEA, we apply the workflow to the sixteen gene expression profiles of eight patients with metastatic melanoma, prepared from pre- and post-treatment experimental batches. You can find the QC interactive report at: https://github.com/isarnassiri/scQCEA/tree/Example-of-Application. Download and unzip the OGC_Interactive_QC_Report_P180121.zip file. You can open CLICK_ME.html file without using rStudio/R. [file 12864_2023_9447_MOESM2_ESM.zip › Inputs/10X-gex/500667_15/P180121-keep_500667_15_Celltype_assignment_HeatMap.png]

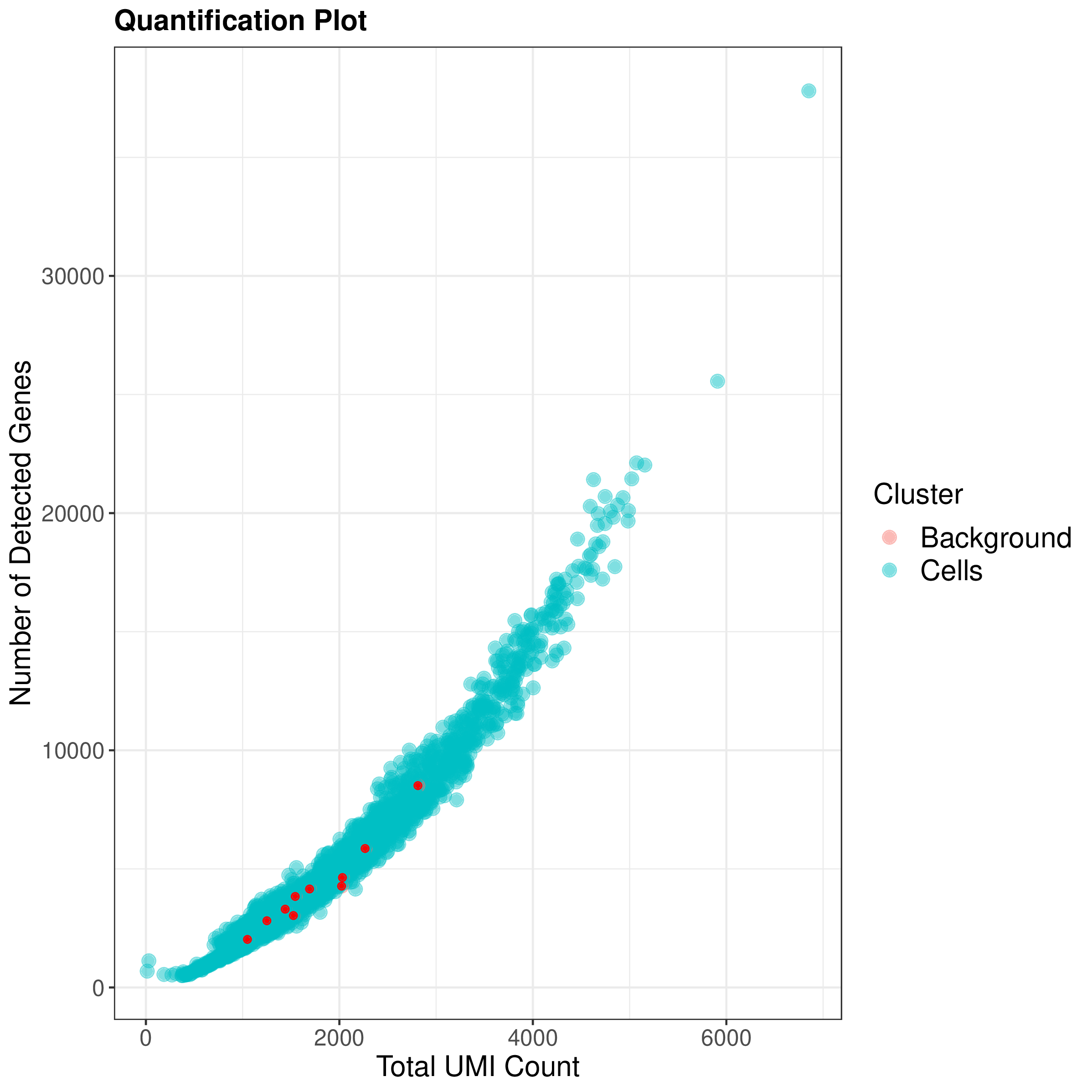

Supplement: Supplementary file 2 — Additional file 2: Supplementary file 2. To demonstrate the utility of scQCEA, we apply the workflow to the sixteen gene expression profiles of eight patients with metastatic melanoma, prepared from pre- and post-treatment experimental batches. You can find the QC interactive report at: https://github.com/isarnassiri/scQCEA/tree/Example-of-Application. Download and unzip the OGC_Interactive_QC_Report_P180121.zip file. You can open CLICK_ME.html file without using rStudio/R. [file 12864_2023_9447_MOESM2_ESM.zip › Inputs/10X-gex/500667_15/P180121-keep_500667_15_TotalUMIvsDetectedGenes.png]

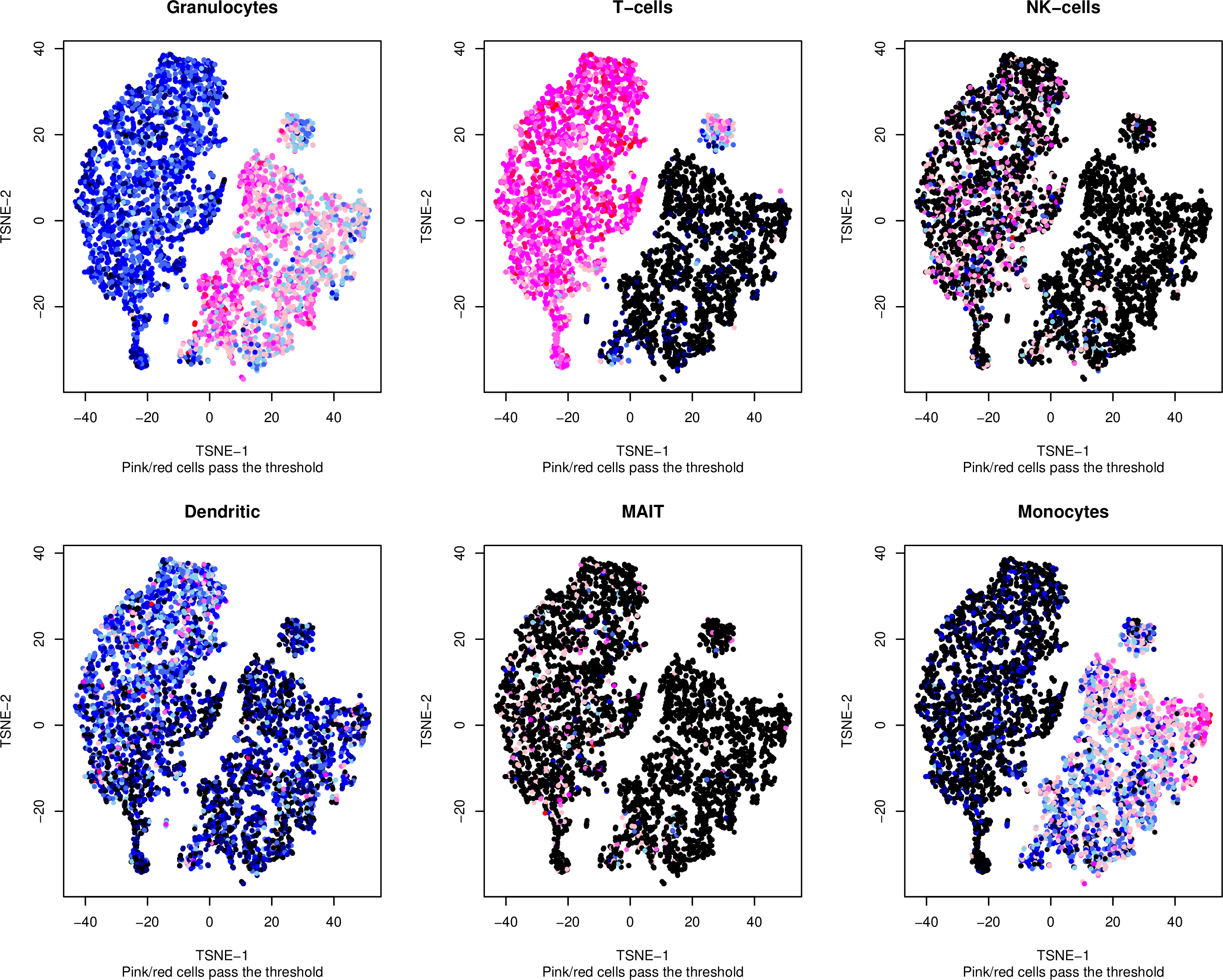

Supplement: Supplementary file 2 — Additional file 2: Supplementary file 2. To demonstrate the utility of scQCEA, we apply the workflow to the sixteen gene expression profiles of eight patients with metastatic melanoma, prepared from pre- and post-treatment experimental batches. You can find the QC interactive report at: https://github.com/isarnassiri/scQCEA/tree/Example-of-Application. Download and unzip the OGC_Interactive_QC_Report_P180121.zip file. You can open CLICK_ME.html file without using rStudio/R. [file 12864_2023_9447_MOESM2_ESM.zip › Inputs/10X-gex/500667_15/P180121-keep_500667_15_tSNE_Plot.png]

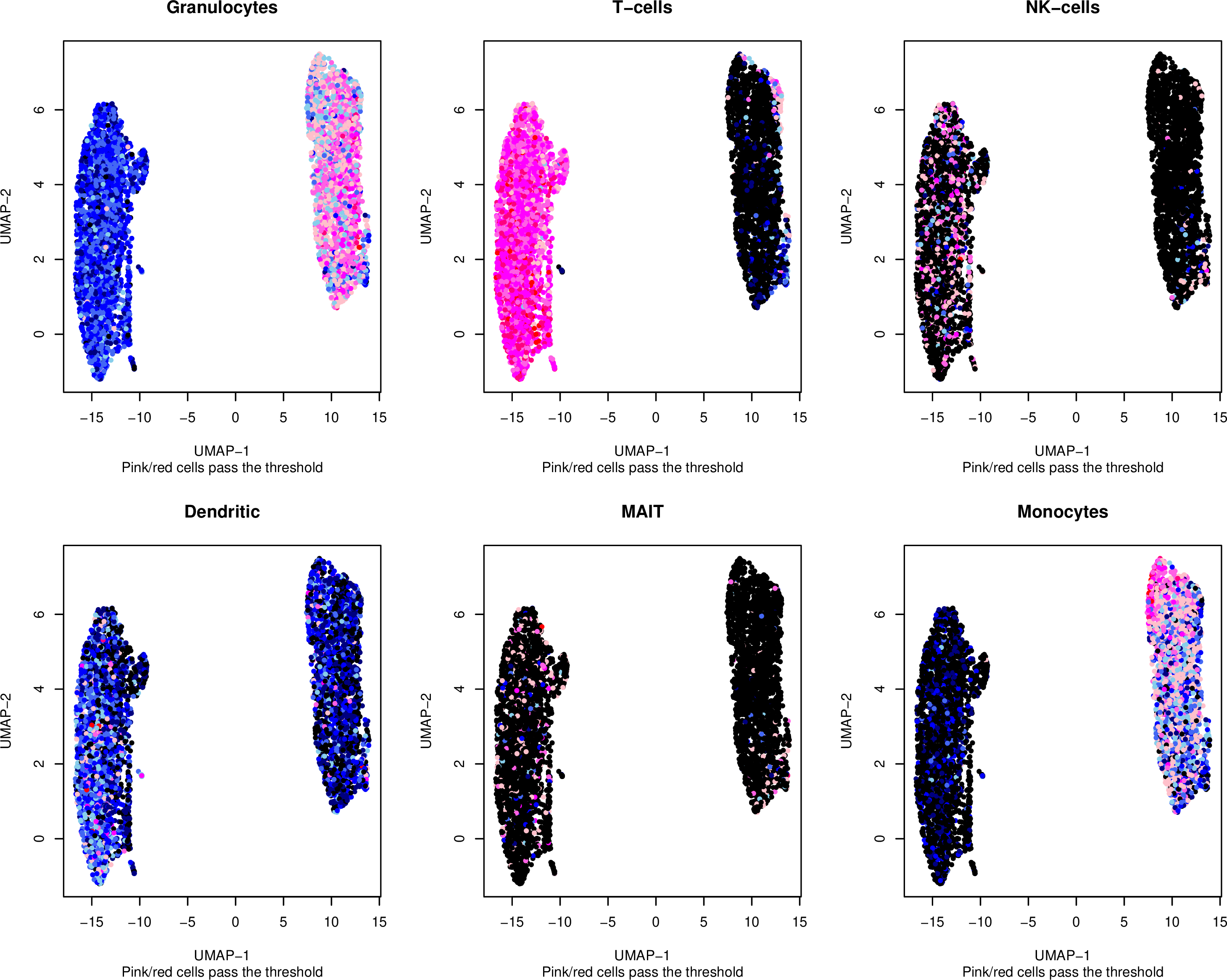

Supplement: Supplementary file 2 — Additional file 2: Supplementary file 2. To demonstrate the utility of scQCEA, we apply the workflow to the sixteen gene expression profiles of eight patients with metastatic melanoma, prepared from pre- and post-treatment experimental batches. You can find the QC interactive report at: https://github.com/isarnassiri/scQCEA/tree/Example-of-Application. Download and unzip the OGC_Interactive_QC_Report_P180121.zip file. You can open CLICK_ME.html file without using rStudio/R. [file 12864_2023_9447_MOESM2_ESM.zip › Inputs/10X-gex/500667_15/P180121-keep_500667_15_UMAP_Plot.png]

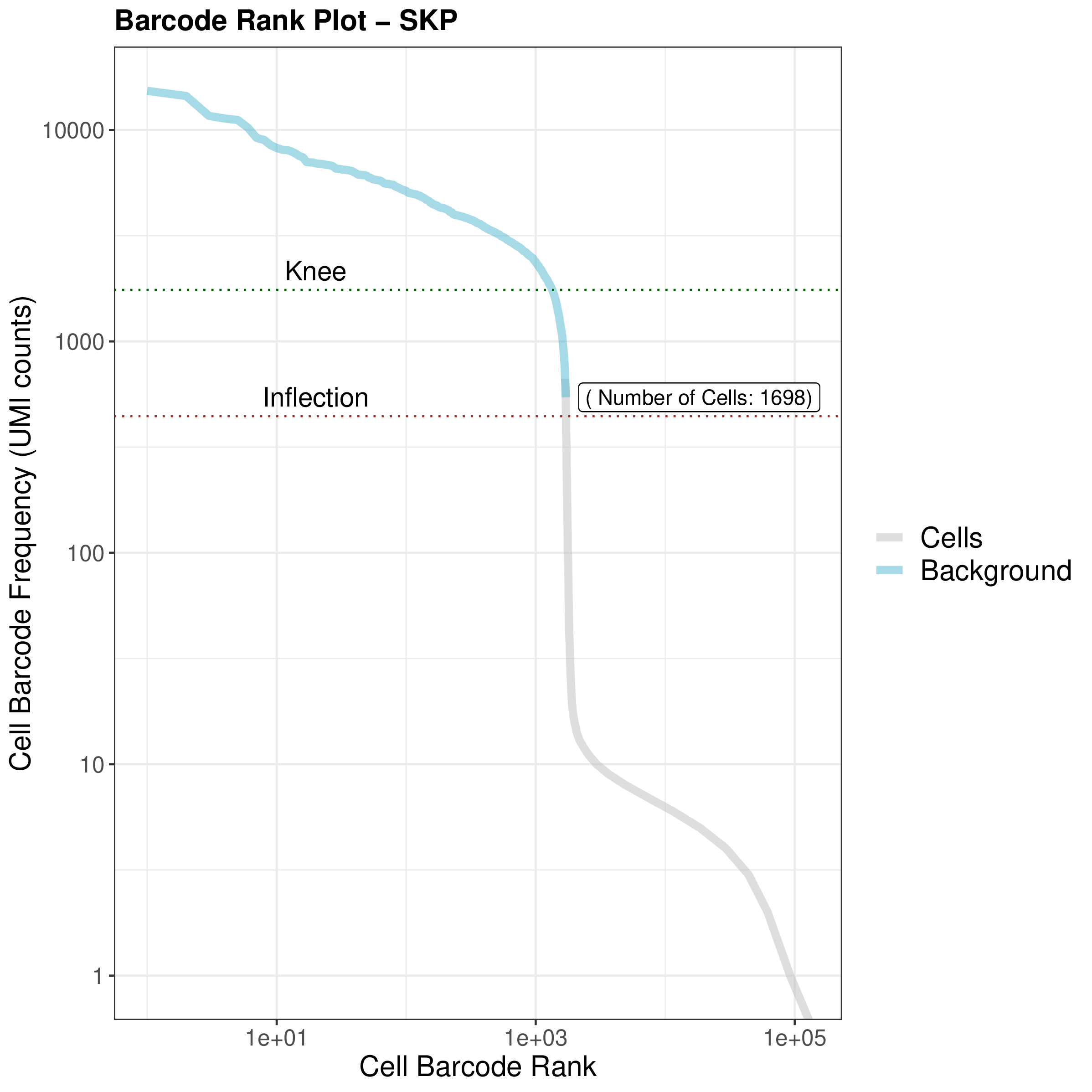

Supplement: Supplementary file 2 — Additional file 2: Supplementary file 2. To demonstrate the utility of scQCEA, we apply the workflow to the sixteen gene expression profiles of eight patients with metastatic melanoma, prepared from pre- and post-treatment experimental batches. You can find the QC interactive report at: https://github.com/isarnassiri/scQCEA/tree/Example-of-Application. Download and unzip the OGC_Interactive_QC_Report_P180121.zip file. You can open CLICK_ME.html file without using rStudio/R. [file 12864_2023_9447_MOESM2_ESM.zip › Inputs/10X-gex/500667_28/P180121-keep_500667_28_BarcodeRankPlot_10X.png]

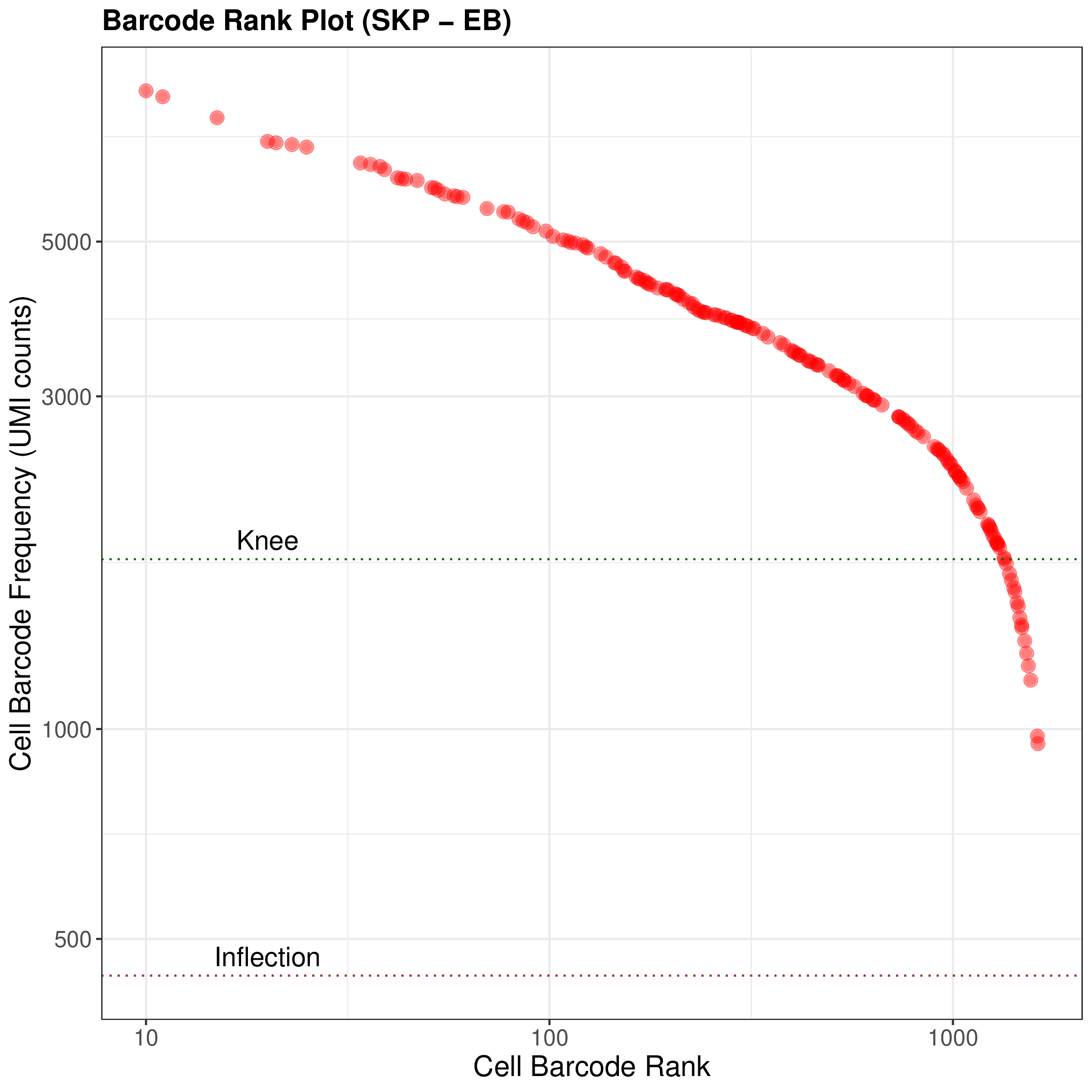

Supplement: Supplementary file 2 — Additional file 2: Supplementary file 2. To demonstrate the utility of scQCEA, we apply the workflow to the sixteen gene expression profiles of eight patients with metastatic melanoma, prepared from pre- and post-treatment experimental batches. You can find the QC interactive report at: https://github.com/isarnassiri/scQCEA/tree/Example-of-Application. Download and unzip the OGC_Interactive_QC_Report_P180121.zip file. You can open CLICK_ME.html file without using rStudio/R. [file 12864_2023_9447_MOESM2_ESM.zip › Inputs/10X-gex/500667_28/P180121-keep_500667_28_BarcodeRankPlot_EB_FilterOut.png]

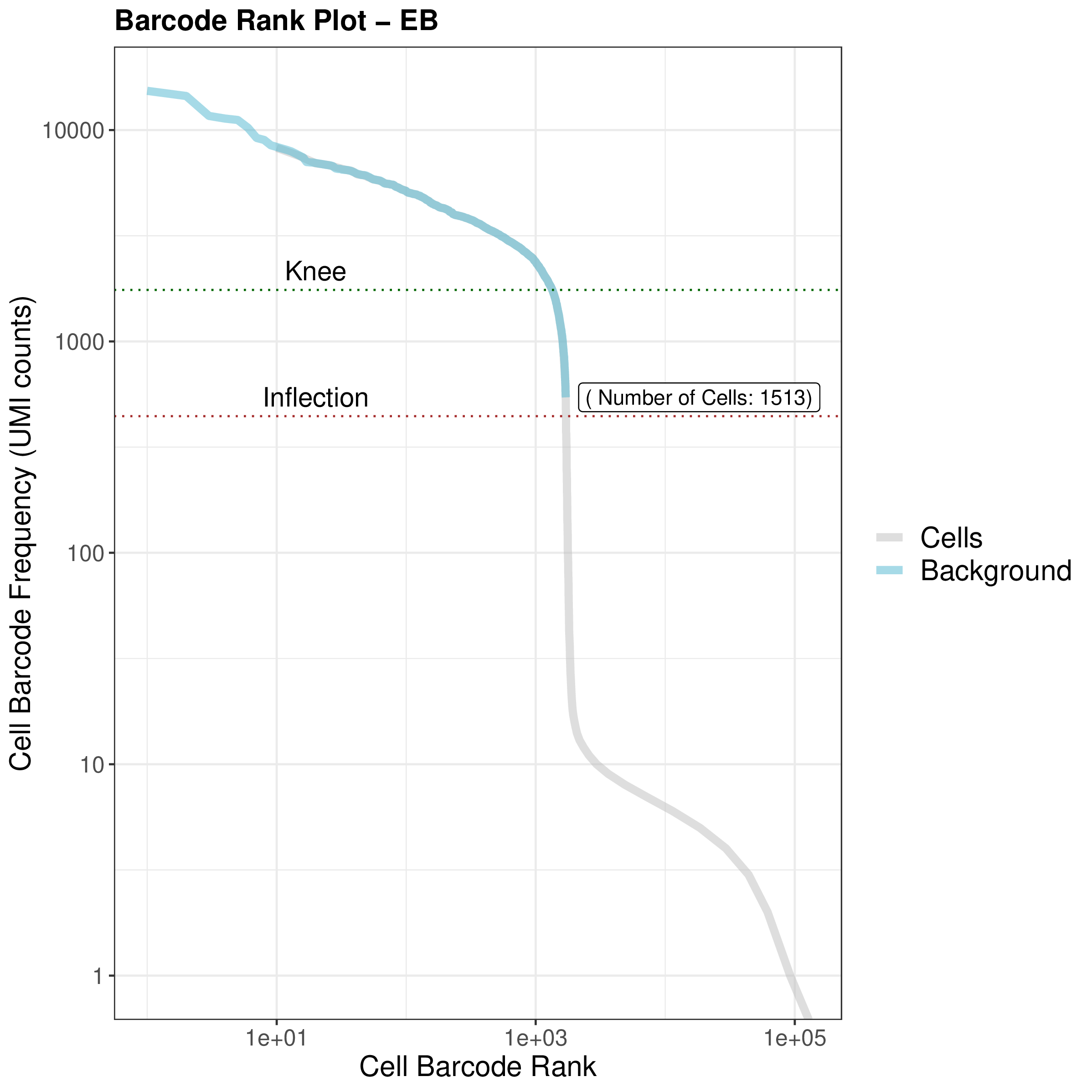

Supplement: Supplementary file 2 — Additional file 2: Supplementary file 2. To demonstrate the utility of scQCEA, we apply the workflow to the sixteen gene expression profiles of eight patients with metastatic melanoma, prepared from pre- and post-treatment experimental batches. You can find the QC interactive report at: https://github.com/isarnassiri/scQCEA/tree/Example-of-Application. Download and unzip the OGC_Interactive_QC_Report_P180121.zip file. You can open CLICK_ME.html file without using rStudio/R. [file 12864_2023_9447_MOESM2_ESM.zip › Inputs/10X-gex/500667_28/P180121-keep_500667_28_BarcodeRankPlot_EB.png]

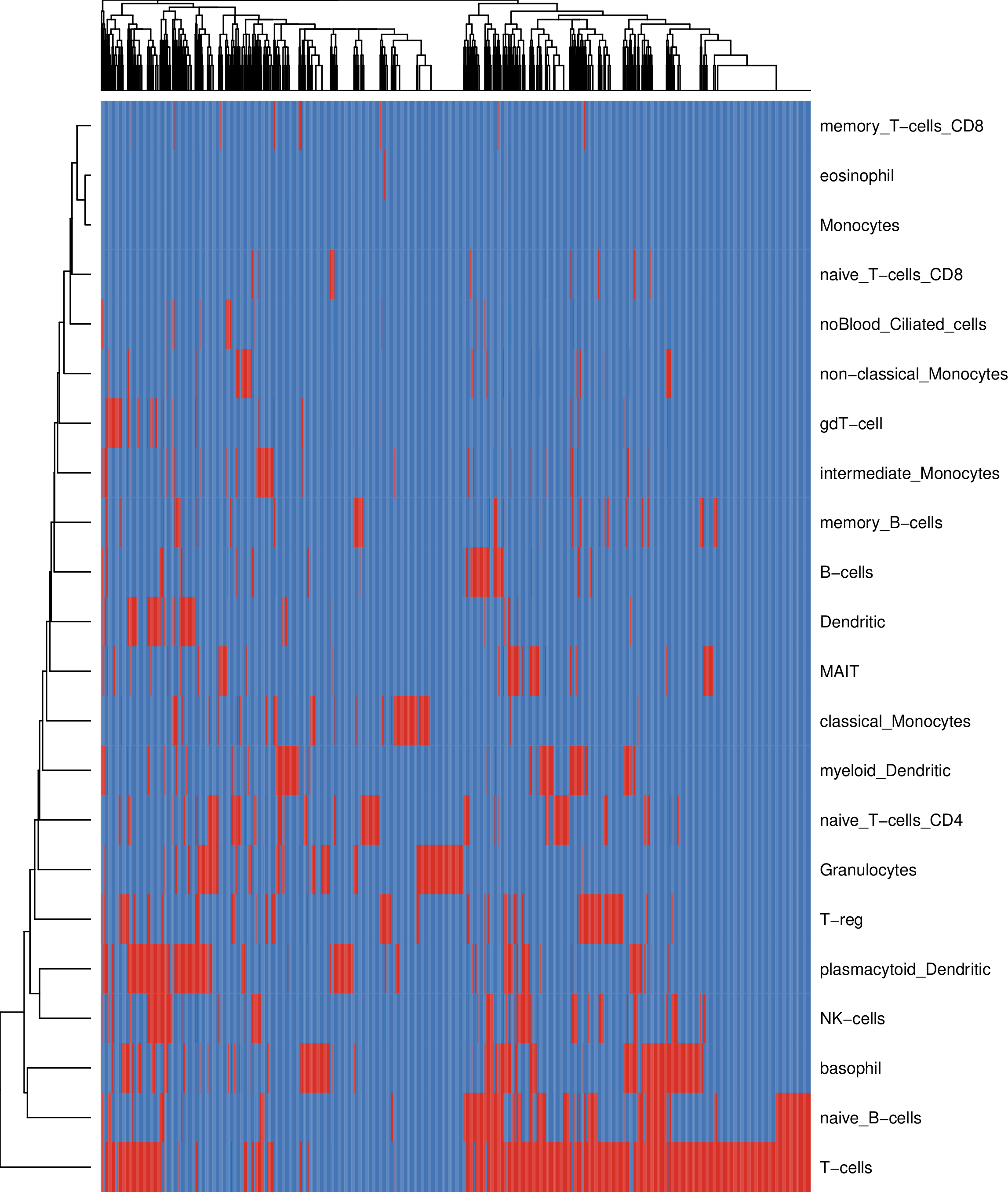

Supplement: Supplementary file 2 — Additional file 2: Supplementary file 2. To demonstrate the utility of scQCEA, we apply the workflow to the sixteen gene expression profiles of eight patients with metastatic melanoma, prepared from pre- and post-treatment experimental batches. You can find the QC interactive report at: https://github.com/isarnassiri/scQCEA/tree/Example-of-Application. Download and unzip the OGC_Interactive_QC_Report_P180121.zip file. You can open CLICK_ME.html file without using rStudio/R. [file 12864_2023_9447_MOESM2_ESM.zip › Inputs/10X-gex/500667_28/P180121-keep_500667_28_Celltype_assignment_HeatMap.png]

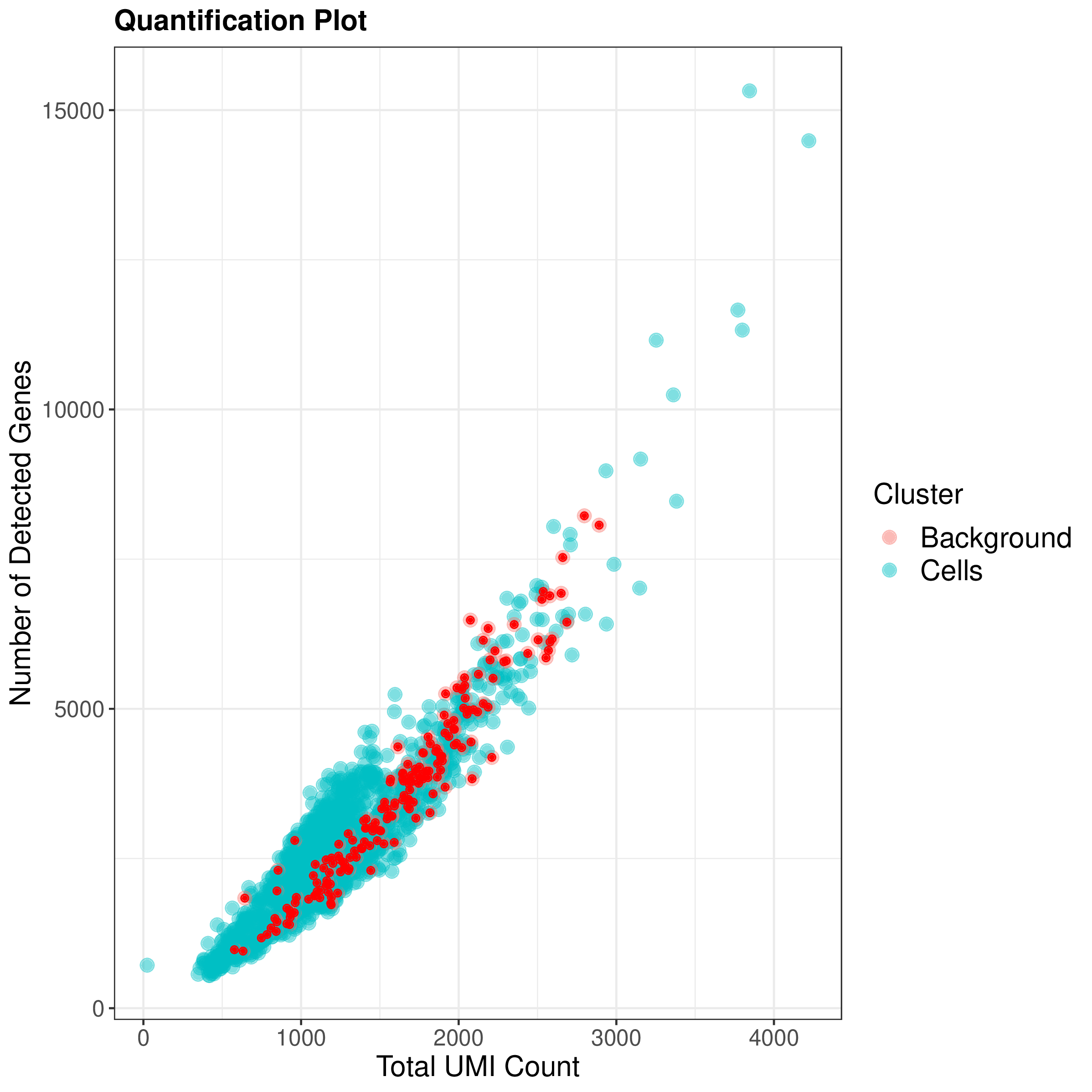

Supplement: Supplementary file 2 — Additional file 2: Supplementary file 2. To demonstrate the utility of scQCEA, we apply the workflow to the sixteen gene expression profiles of eight patients with metastatic melanoma, prepared from pre- and post-treatment experimental batches. You can find the QC interactive report at: https://github.com/isarnassiri/scQCEA/tree/Example-of-Application. Download and unzip the OGC_Interactive_QC_Report_P180121.zip file. You can open CLICK_ME.html file without using rStudio/R. [file 12864_2023_9447_MOESM2_ESM.zip › Inputs/10X-gex/500667_28/P180121-keep_500667_28_TotalUMIvsDetectedGenes.png]

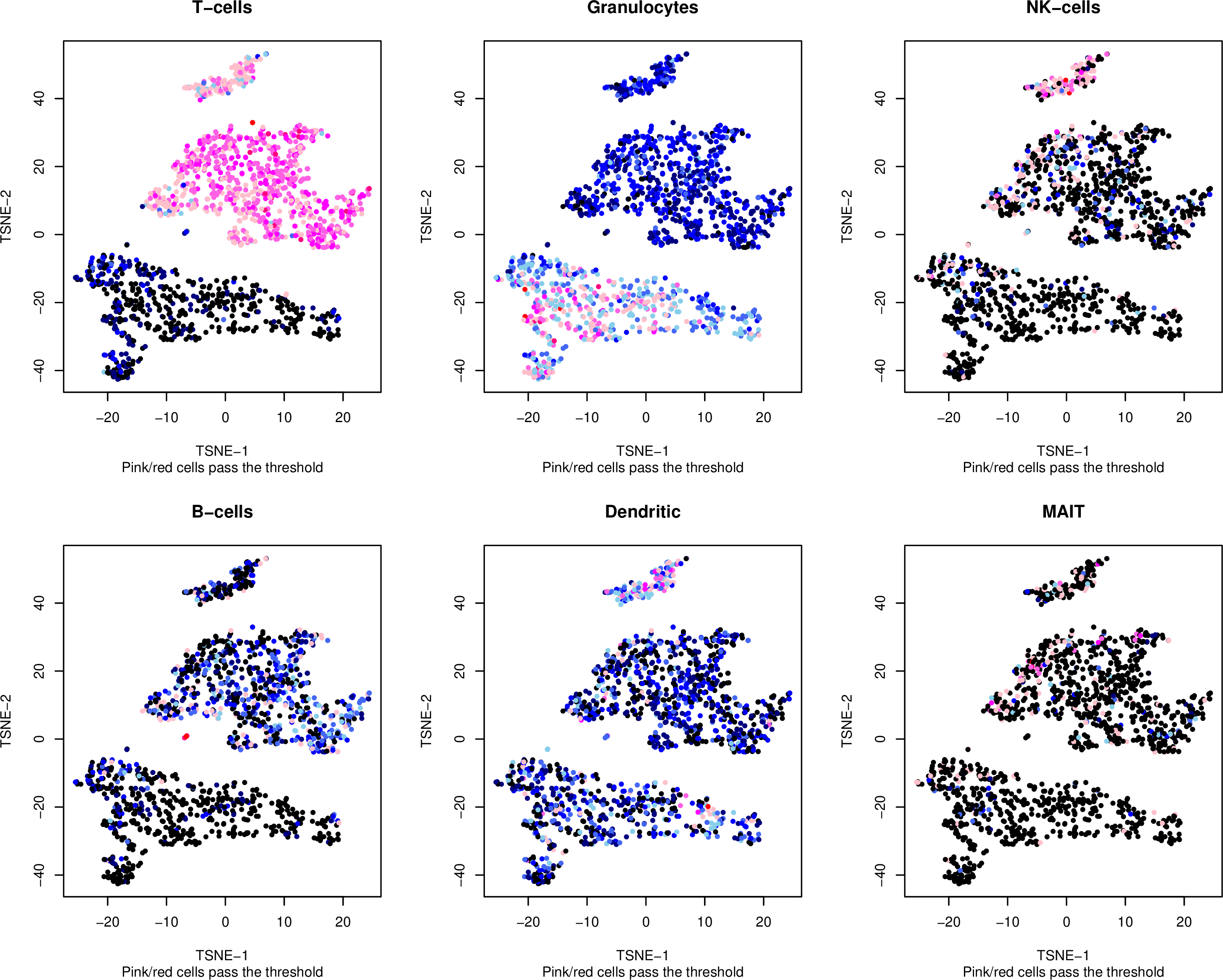

Supplement: Supplementary file 2 — Additional file 2: Supplementary file 2. To demonstrate the utility of scQCEA, we apply the workflow to the sixteen gene expression profiles of eight patients with metastatic melanoma, prepared from pre- and post-treatment experimental batches. You can find the QC interactive report at: https://github.com/isarnassiri/scQCEA/tree/Example-of-Application. Download and unzip the OGC_Interactive_QC_Report_P180121.zip file. You can open CLICK_ME.html file without using rStudio/R. [file 12864_2023_9447_MOESM2_ESM.zip › Inputs/10X-gex/500667_28/P180121-keep_500667_28_tSNE_Plot.png]

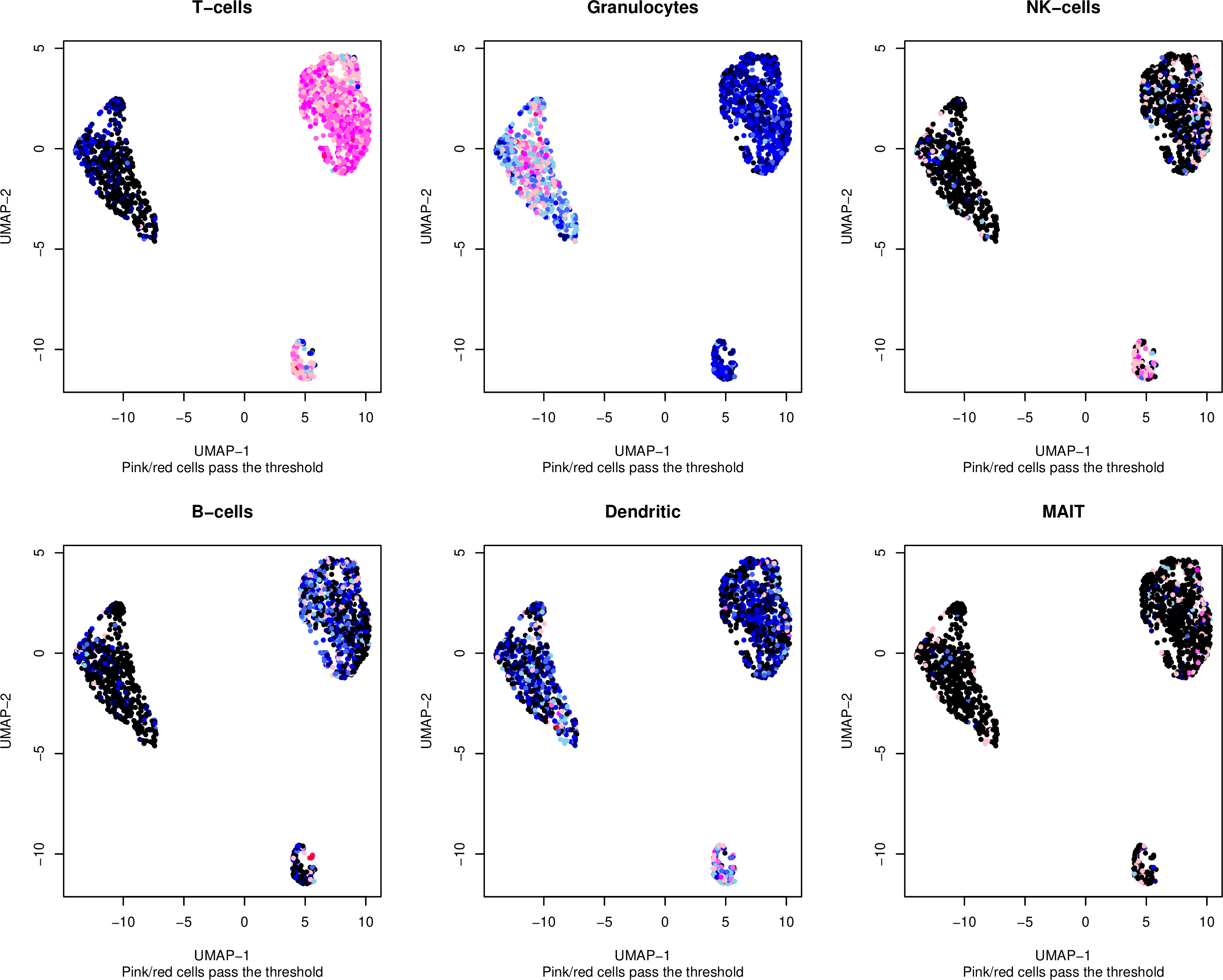

Supplement: Supplementary file 2 — Additional file 2: Supplementary file 2. To demonstrate the utility of scQCEA, we apply the workflow to the sixteen gene expression profiles of eight patients with metastatic melanoma, prepared from pre- and post-treatment experimental batches. You can find the QC interactive report at: https://github.com/isarnassiri/scQCEA/tree/Example-of-Application. Download and unzip the OGC_Interactive_QC_Report_P180121.zip file. You can open CLICK_ME.html file without using rStudio/R. [file 12864_2023_9447_MOESM2_ESM.zip › Inputs/10X-gex/500667_28/P180121-keep_500667_28_UMAP_Plot.png]

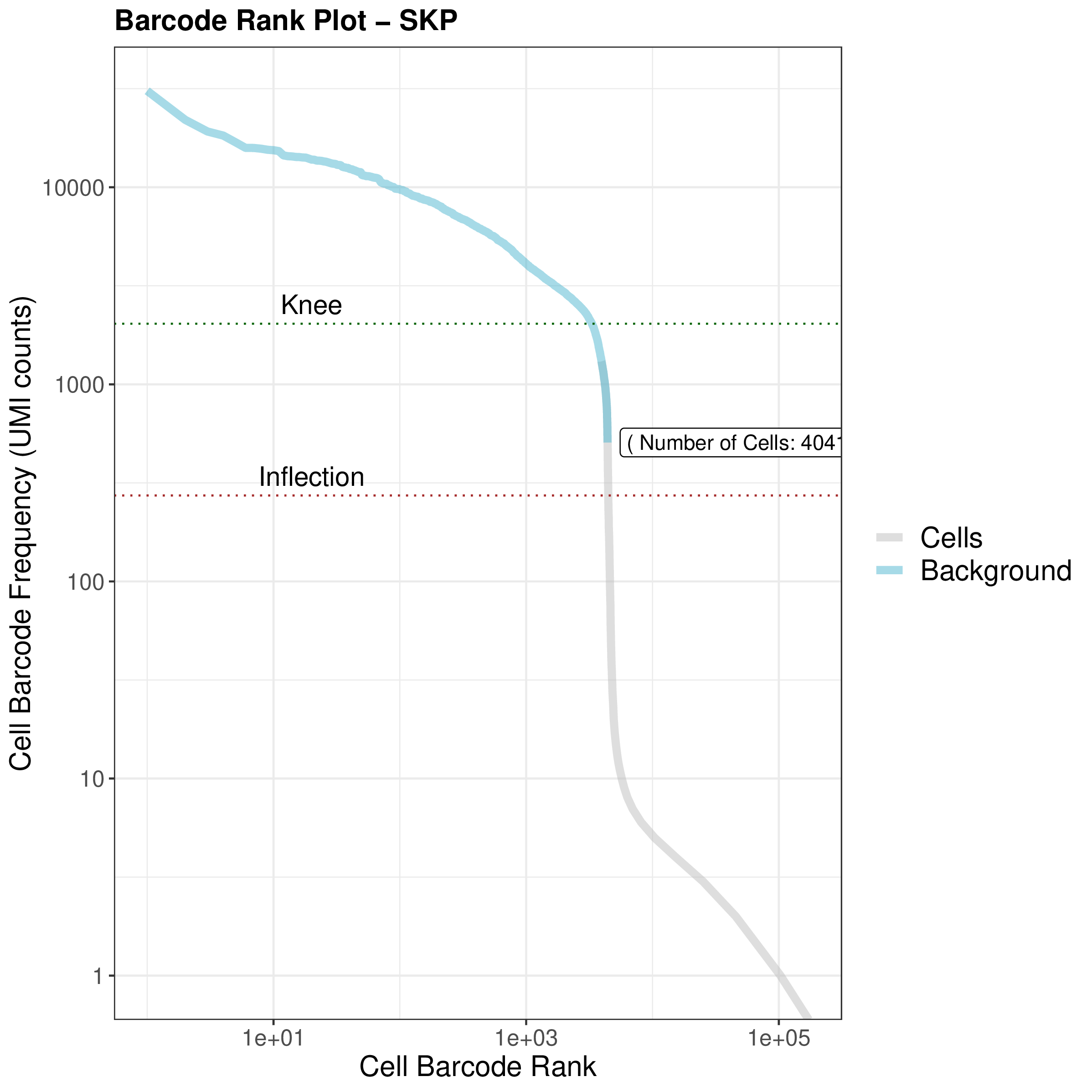

Supplement: Supplementary file 2 — Additional file 2: Supplementary file 2. To demonstrate the utility of scQCEA, we apply the workflow to the sixteen gene expression profiles of eight patients with metastatic melanoma, prepared from pre- and post-treatment experimental batches. You can find the QC interactive report at: https://github.com/isarnassiri/scQCEA/tree/Example-of-Application. Download and unzip the OGC_Interactive_QC_Report_P180121.zip file. You can open CLICK_ME.html file without using rStudio/R. [file 12864_2023_9447_MOESM2_ESM.zip › Inputs/10X-gex/500667_40/P180121-keep_500667_40_BarcodeRankPlot_10X.png]

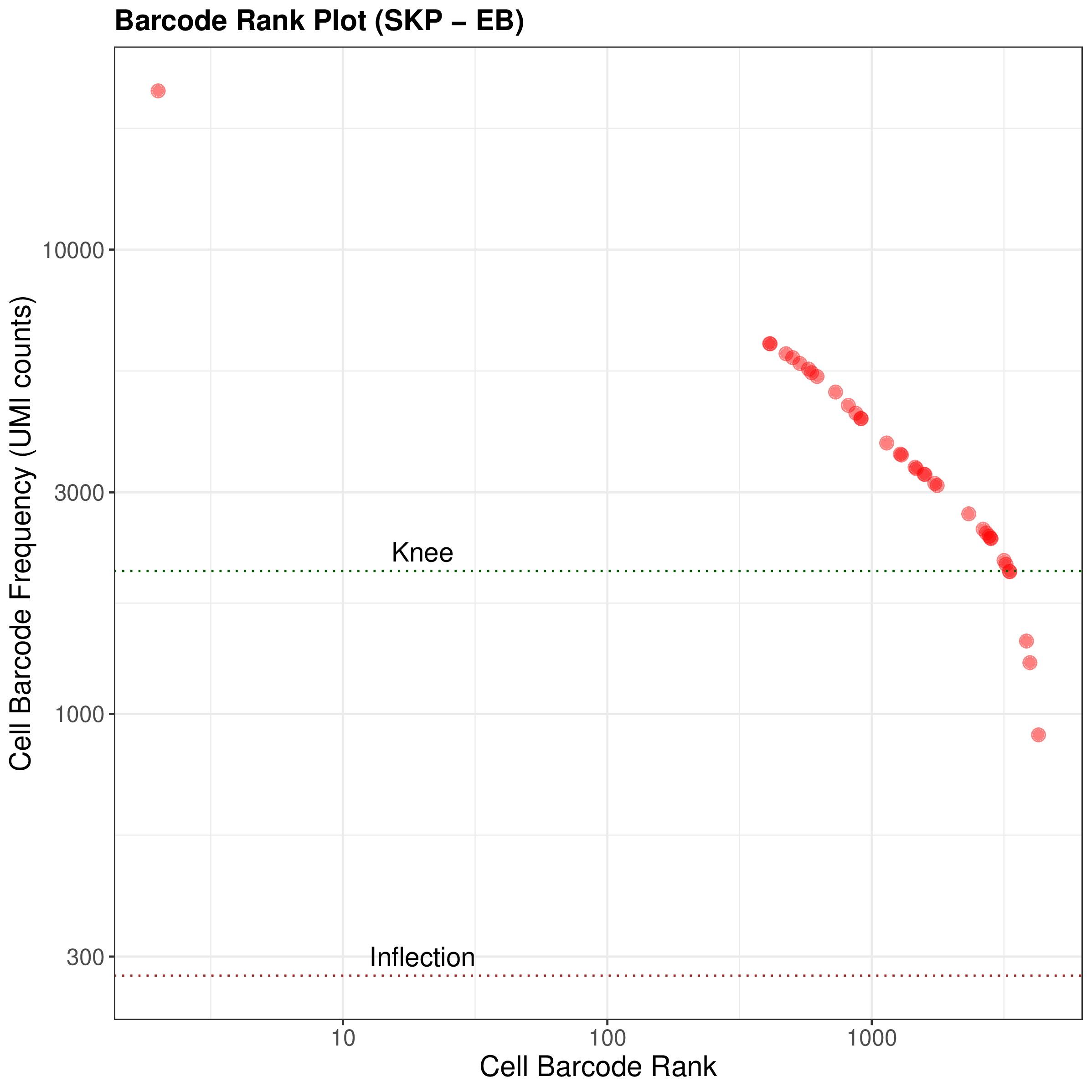

Supplement: Supplementary file 2 — Additional file 2: Supplementary file 2. To demonstrate the utility of scQCEA, we apply the workflow to the sixteen gene expression profiles of eight patients with metastatic melanoma, prepared from pre- and post-treatment experimental batches. You can find the QC interactive report at: https://github.com/isarnassiri/scQCEA/tree/Example-of-Application. Download and unzip the OGC_Interactive_QC_Report_P180121.zip file. You can open CLICK_ME.html file without using rStudio/R. [file 12864_2023_9447_MOESM2_ESM.zip › Inputs/10X-gex/500667_40/P180121-keep_500667_40_BarcodeRankPlot_EB_FilterOut.png]

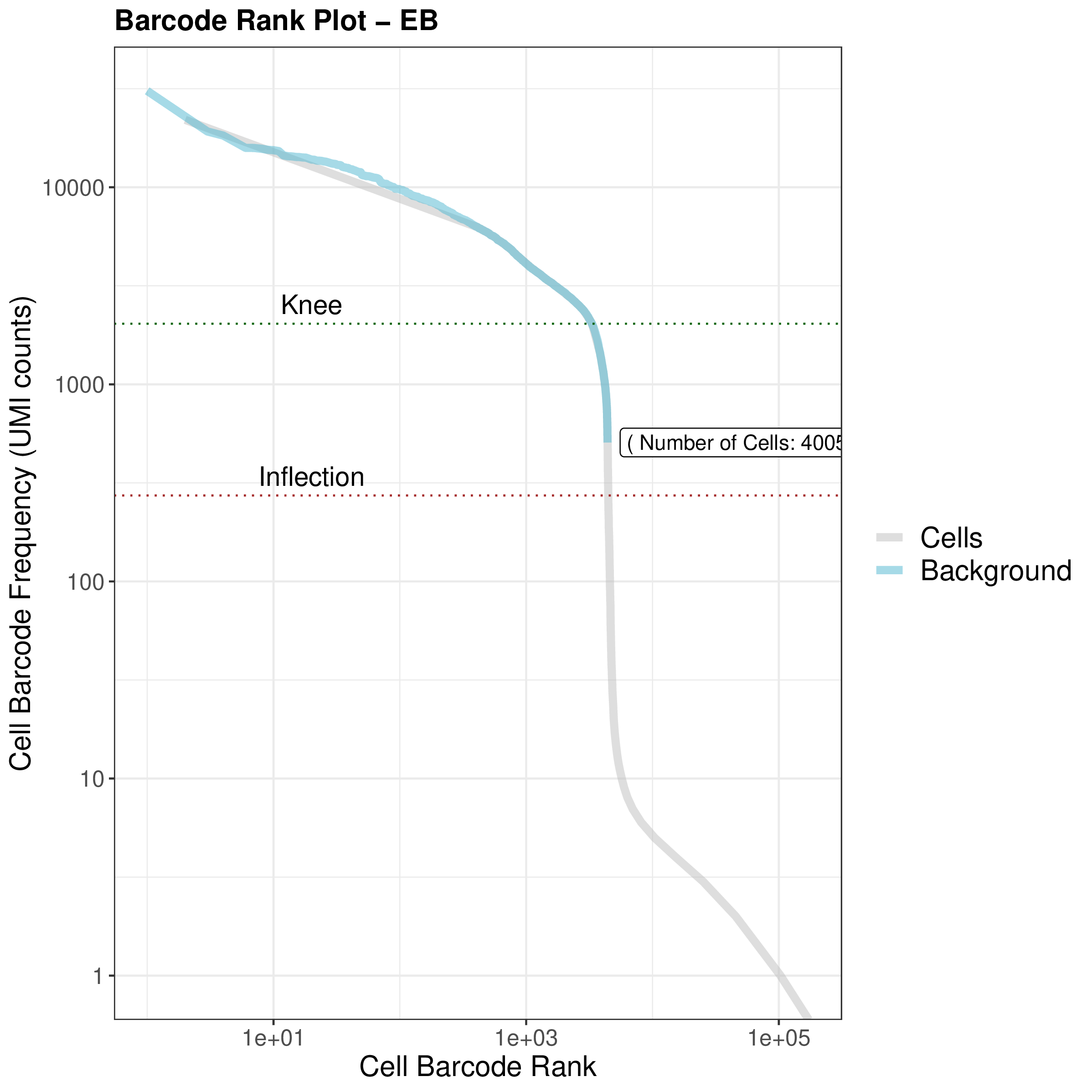

Supplement: Supplementary file 2 — Additional file 2: Supplementary file 2. To demonstrate the utility of scQCEA, we apply the workflow to the sixteen gene expression profiles of eight patients with metastatic melanoma, prepared from pre- and post-treatment experimental batches. You can find the QC interactive report at: https://github.com/isarnassiri/scQCEA/tree/Example-of-Application. Download and unzip the OGC_Interactive_QC_Report_P180121.zip file. You can open CLICK_ME.html file without using rStudio/R. [file 12864_2023_9447_MOESM2_ESM.zip › Inputs/10X-gex/500667_40/P180121-keep_500667_40_BarcodeRankPlot_EB.png]

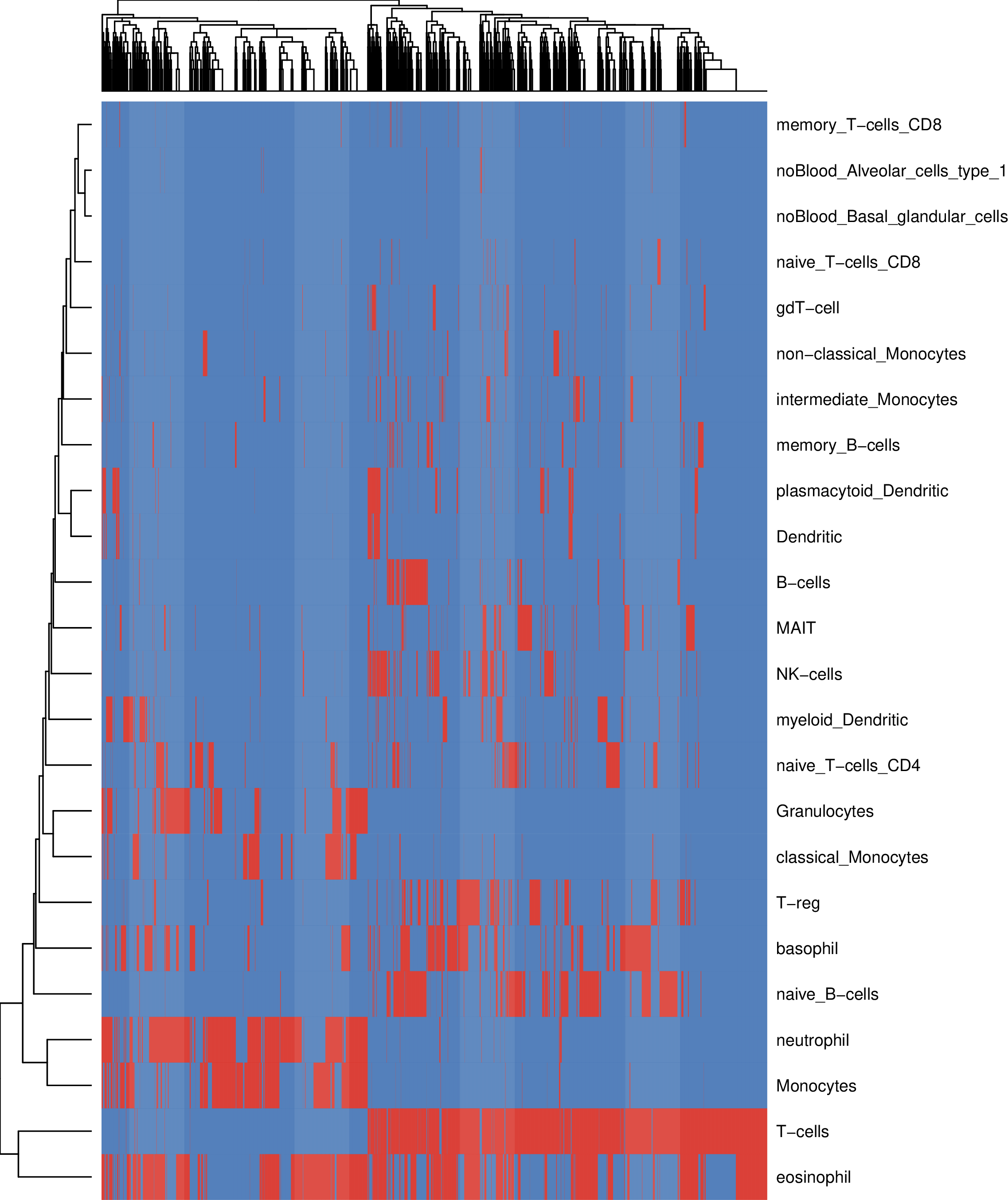

Supplement: Supplementary file 2 — Additional file 2: Supplementary file 2. To demonstrate the utility of scQCEA, we apply the workflow to the sixteen gene expression profiles of eight patients with metastatic melanoma, prepared from pre- and post-treatment experimental batches. You can find the QC interactive report at: https://github.com/isarnassiri/scQCEA/tree/Example-of-Application. Download and unzip the OGC_Interactive_QC_Report_P180121.zip file. You can open CLICK_ME.html file without using rStudio/R. [file 12864_2023_9447_MOESM2_ESM.zip › Inputs/10X-gex/500667_40/P180121-keep_500667_40_Celltype_assignment_HeatMap.png]

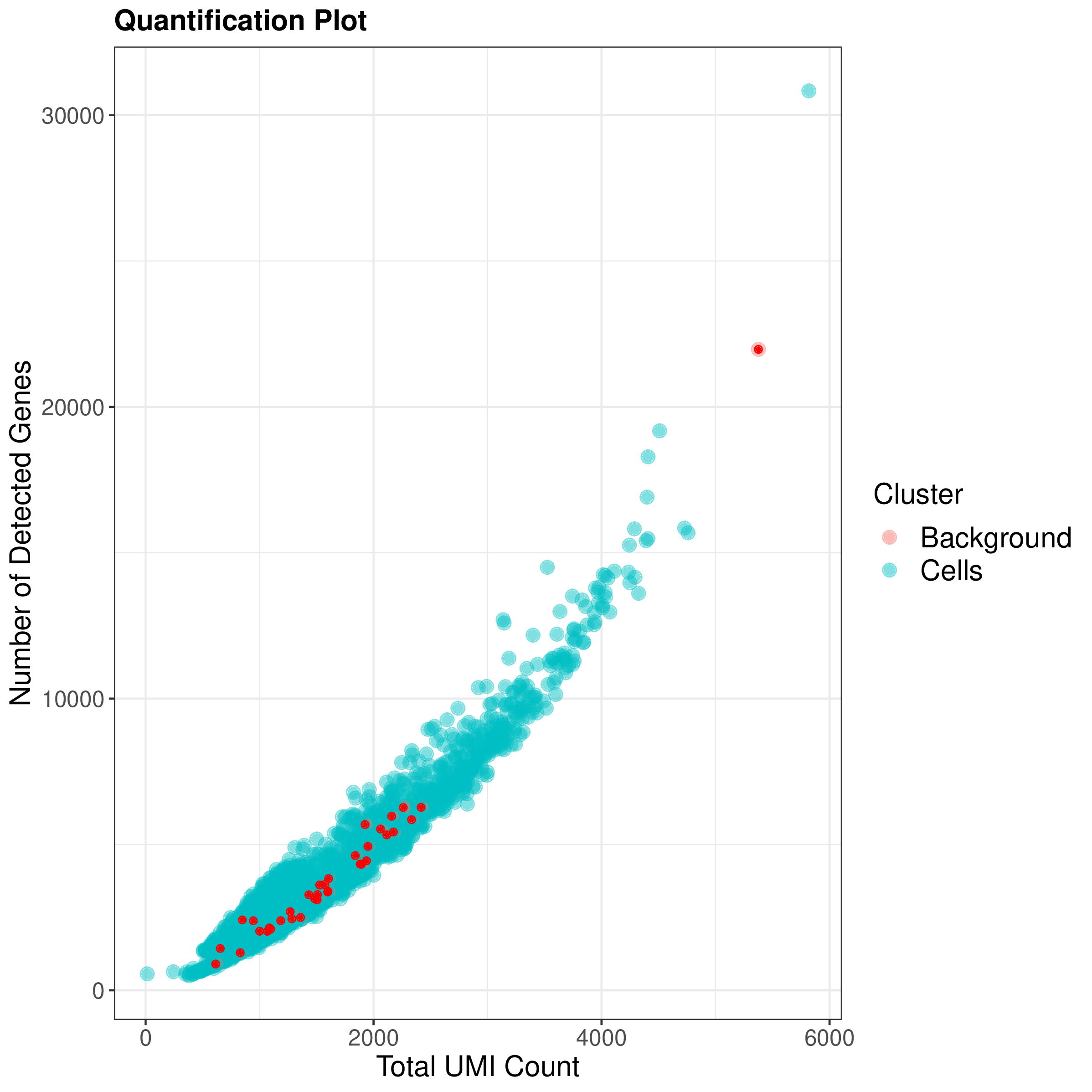

Supplement: Supplementary file 2 — Additional file 2: Supplementary file 2. To demonstrate the utility of scQCEA, we apply the workflow to the sixteen gene expression profiles of eight patients with metastatic melanoma, prepared from pre- and post-treatment experimental batches. You can find the QC interactive report at: https://github.com/isarnassiri/scQCEA/tree/Example-of-Application. Download and unzip the OGC_Interactive_QC_Report_P180121.zip file. You can open CLICK_ME.html file without using rStudio/R. [file 12864_2023_9447_MOESM2_ESM.zip › Inputs/10X-gex/500667_40/P180121-keep_500667_40_TotalUMIvsDetectedGenes.png]

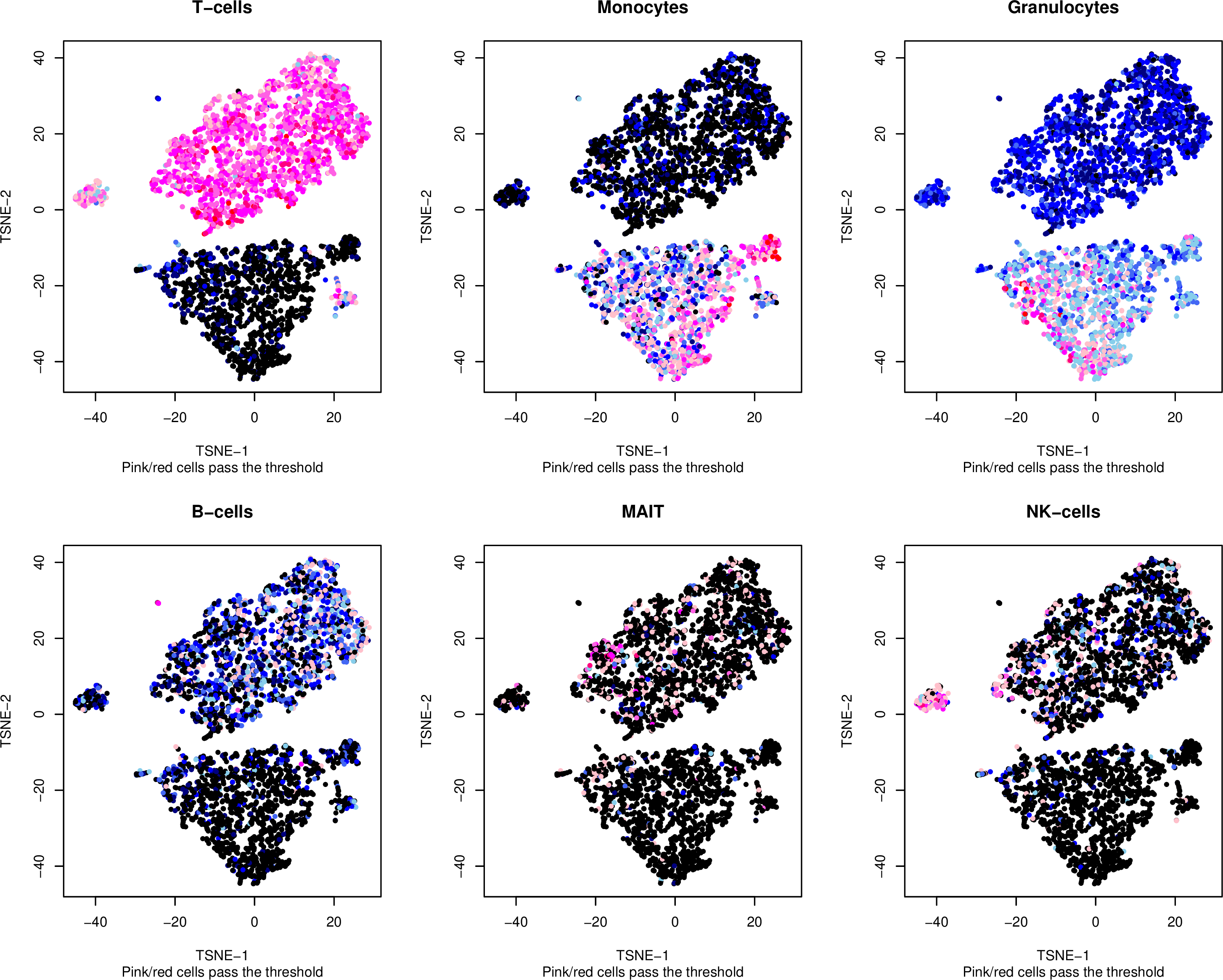

Supplement: Supplementary file 2 — Additional file 2: Supplementary file 2. To demonstrate the utility of scQCEA, we apply the workflow to the sixteen gene expression profiles of eight patients with metastatic melanoma, prepared from pre- and post-treatment experimental batches. You can find the QC interactive report at: https://github.com/isarnassiri/scQCEA/tree/Example-of-Application. Download and unzip the OGC_Interactive_QC_Report_P180121.zip file. You can open CLICK_ME.html file without using rStudio/R. [file 12864_2023_9447_MOESM2_ESM.zip › Inputs/10X-gex/500667_40/P180121-keep_500667_40_tSNE_Plot.png]
