# Supplementary figures and images for: scQCEA: a framework for annotation and quality control report of single-cell RNA-sequencing data (part 1 of 2)
Source: BMC Genomics. 2023 Jul 6;24:381. doi: 10.1186/s12864-023-09447-6 (PMC10327311; doi:10.1186/s12864-023-09447-6)

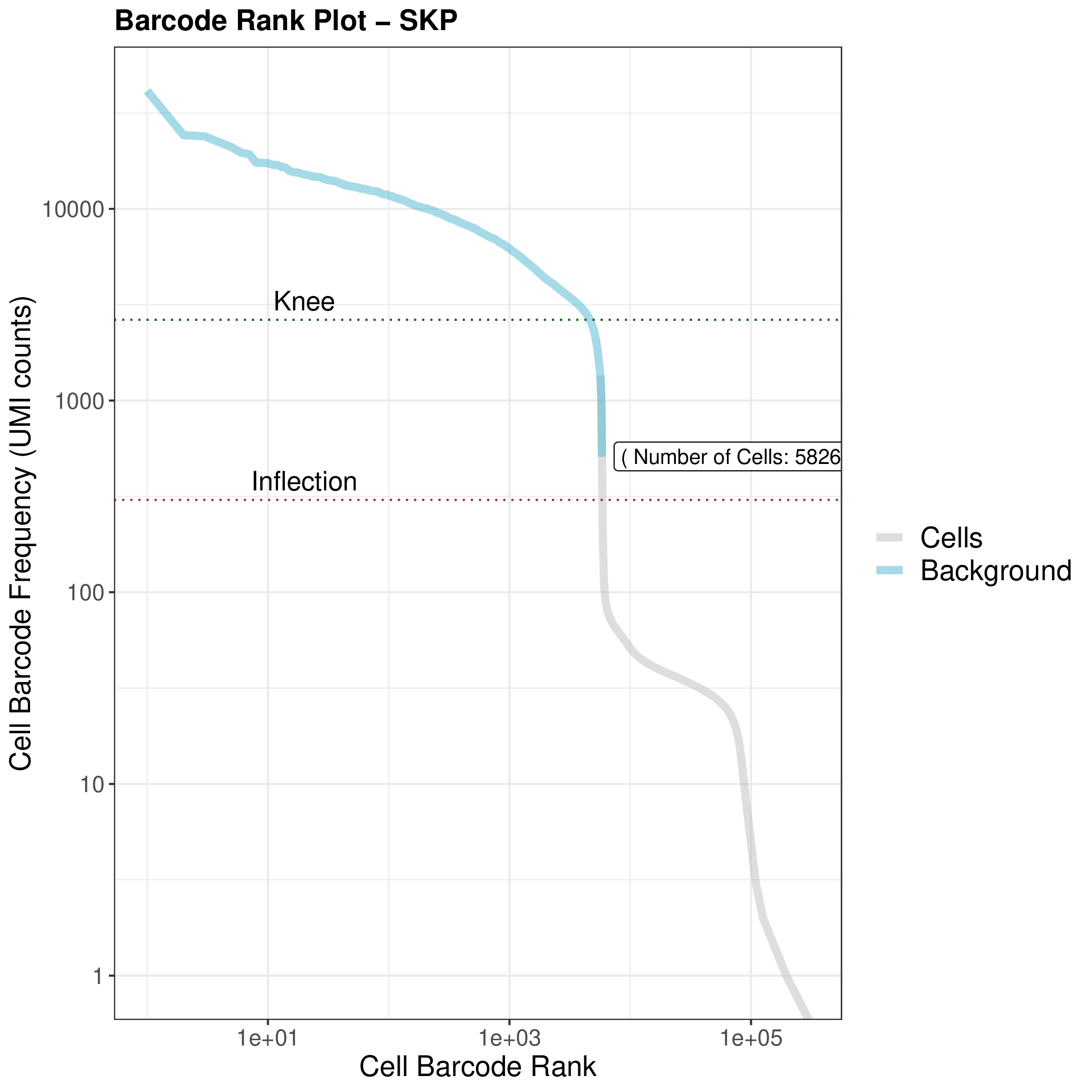

Supplement: Supplementary file 2 — Additional file 2: Supplementary file 2. To demonstrate the utility of scQCEA, we apply the workflow to the sixteen gene expression profiles of eight patients with metastatic melanoma, prepared from pre- and post-treatment experimental batches. You can find the QC interactive report at: https://github.com/isarnassiri/scQCEA/tree/Example-of-Application. Download and unzip the OGC_Interactive_QC_Report_P180121.zip file. You can open CLICK_ME.html file without using rStudio/R. [file 12864_2023_9447_MOESM2_ESM.zip › Inputs/10X-gex-grouped/FAI5649A17/P180121-keep_FAI5649A17_BarcodeRankPlot_10X.png]

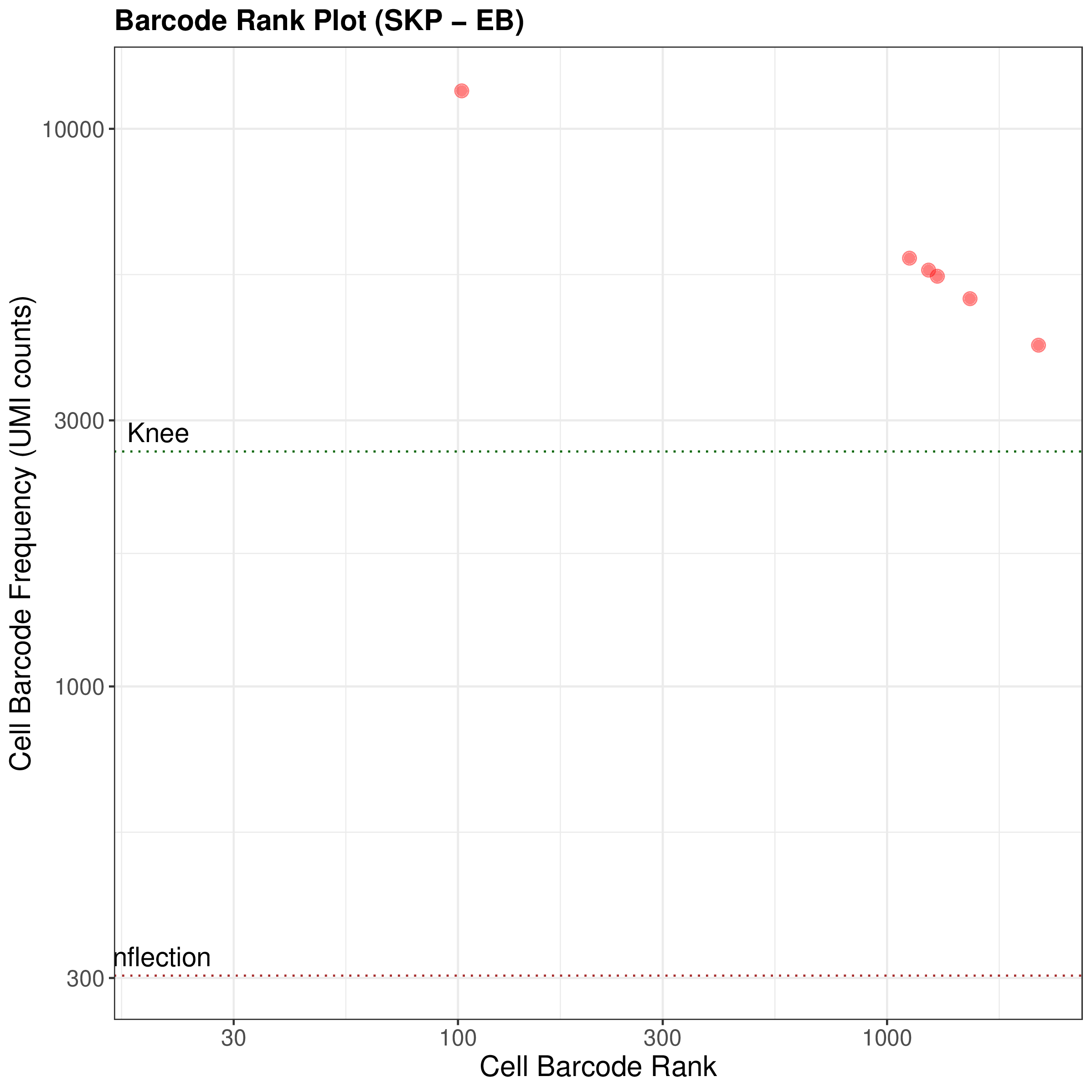

Supplement: Supplementary file 2 — Additional file 2: Supplementary file 2. To demonstrate the utility of scQCEA, we apply the workflow to the sixteen gene expression profiles of eight patients with metastatic melanoma, prepared from pre- and post-treatment experimental batches. You can find the QC interactive report at: https://github.com/isarnassiri/scQCEA/tree/Example-of-Application. Download and unzip the OGC_Interactive_QC_Report_P180121.zip file. You can open CLICK_ME.html file without using rStudio/R. [file 12864_2023_9447_MOESM2_ESM.zip › Inputs/10X-gex-grouped/FAI5649A17/P180121-keep_FAI5649A17_BarcodeRankPlot_EB_FilterOut.png]

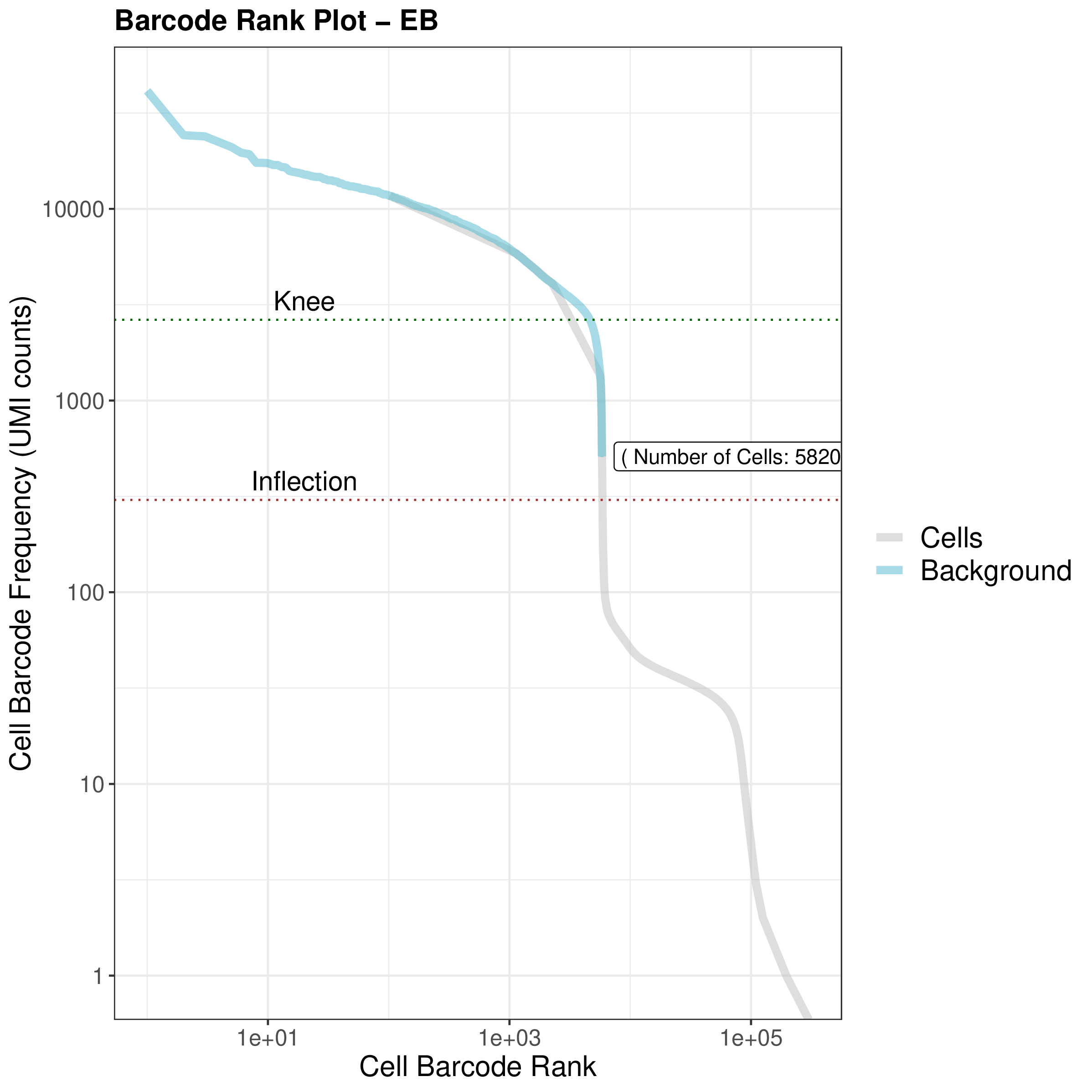

Supplement: Supplementary file 2 — Additional file 2: Supplementary file 2. To demonstrate the utility of scQCEA, we apply the workflow to the sixteen gene expression profiles of eight patients with metastatic melanoma, prepared from pre- and post-treatment experimental batches. You can find the QC interactive report at: https://github.com/isarnassiri/scQCEA/tree/Example-of-Application. Download and unzip the OGC_Interactive_QC_Report_P180121.zip file. You can open CLICK_ME.html file without using rStudio/R. [file 12864_2023_9447_MOESM2_ESM.zip › Inputs/10X-gex-grouped/FAI5649A17/P180121-keep_FAI5649A17_BarcodeRankPlot_EB.png]

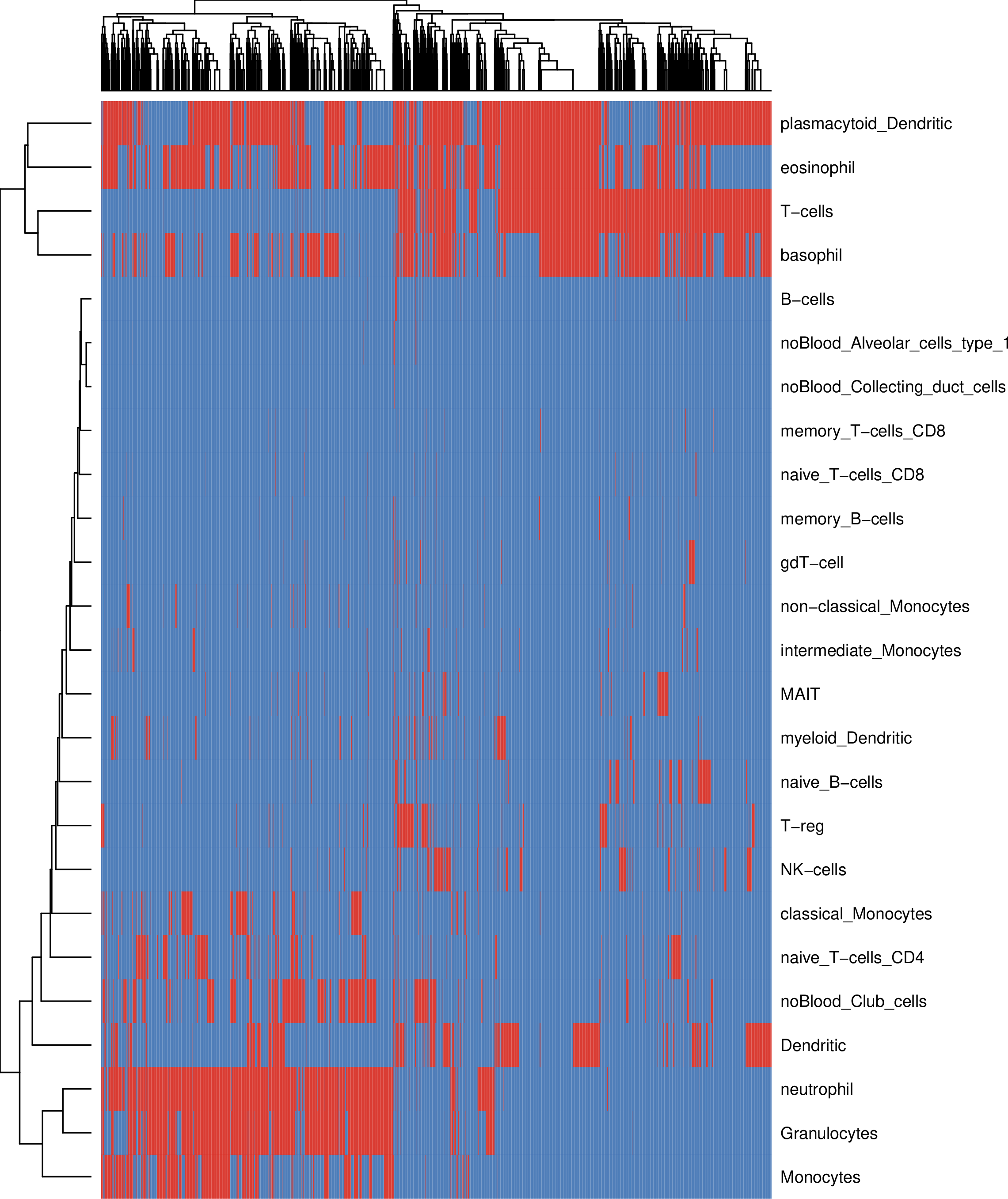

Supplement: Supplementary file 2 — Additional file 2: Supplementary file 2. To demonstrate the utility of scQCEA, we apply the workflow to the sixteen gene expression profiles of eight patients with metastatic melanoma, prepared from pre- and post-treatment experimental batches. You can find the QC interactive report at: https://github.com/isarnassiri/scQCEA/tree/Example-of-Application. Download and unzip the OGC_Interactive_QC_Report_P180121.zip file. You can open CLICK_ME.html file without using rStudio/R. [file 12864_2023_9447_MOESM2_ESM.zip › Inputs/10X-gex-grouped/FAI5649A17/P180121-keep_FAI5649A17_Celltype_assignment_HeatMap.png]

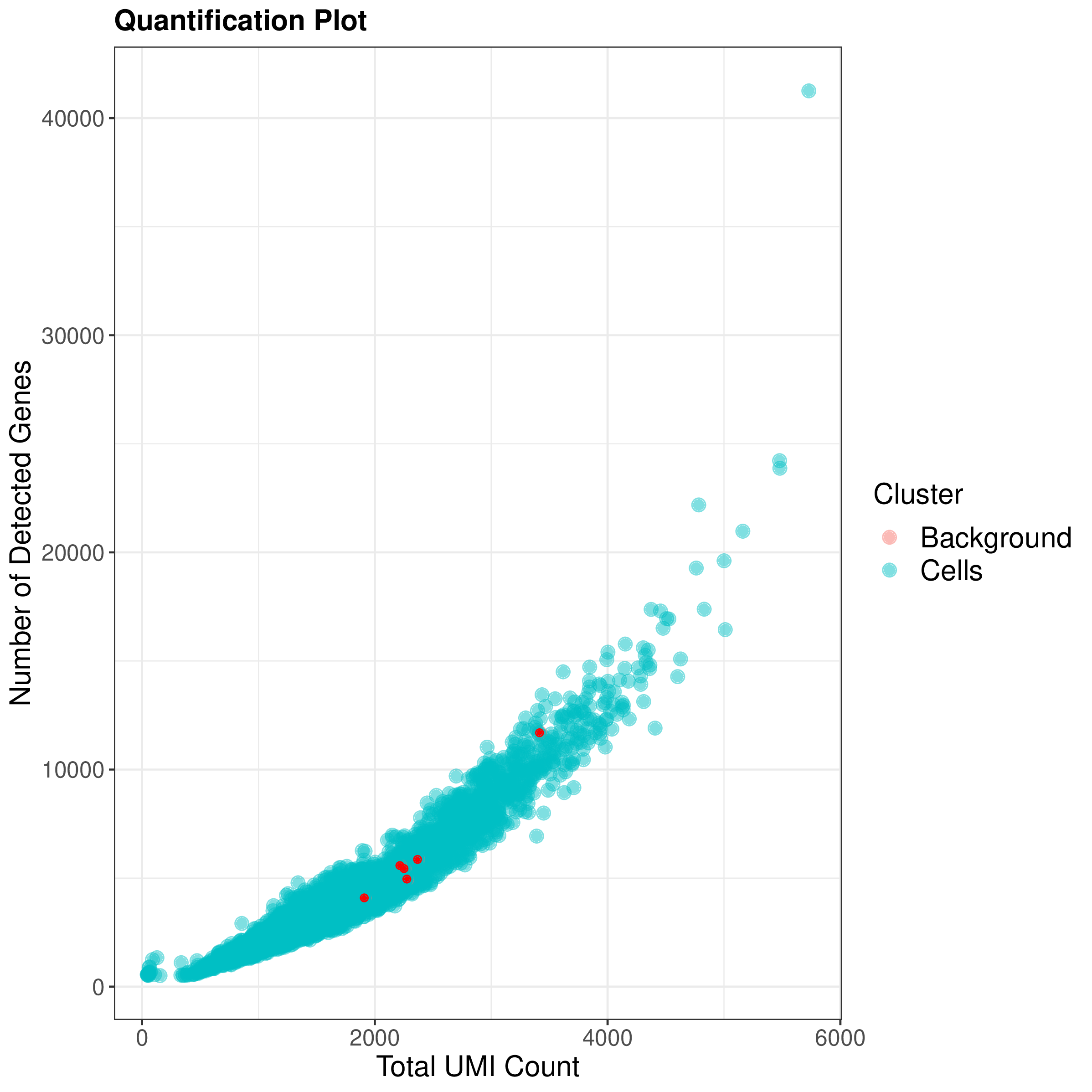

Supplement: Supplementary file 2 — Additional file 2: Supplementary file 2. To demonstrate the utility of scQCEA, we apply the workflow to the sixteen gene expression profiles of eight patients with metastatic melanoma, prepared from pre- and post-treatment experimental batches. You can find the QC interactive report at: https://github.com/isarnassiri/scQCEA/tree/Example-of-Application. Download and unzip the OGC_Interactive_QC_Report_P180121.zip file. You can open CLICK_ME.html file without using rStudio/R. [file 12864_2023_9447_MOESM2_ESM.zip › Inputs/10X-gex-grouped/FAI5649A17/P180121-keep_FAI5649A17_TotalUMIvsDetectedGenes.png]

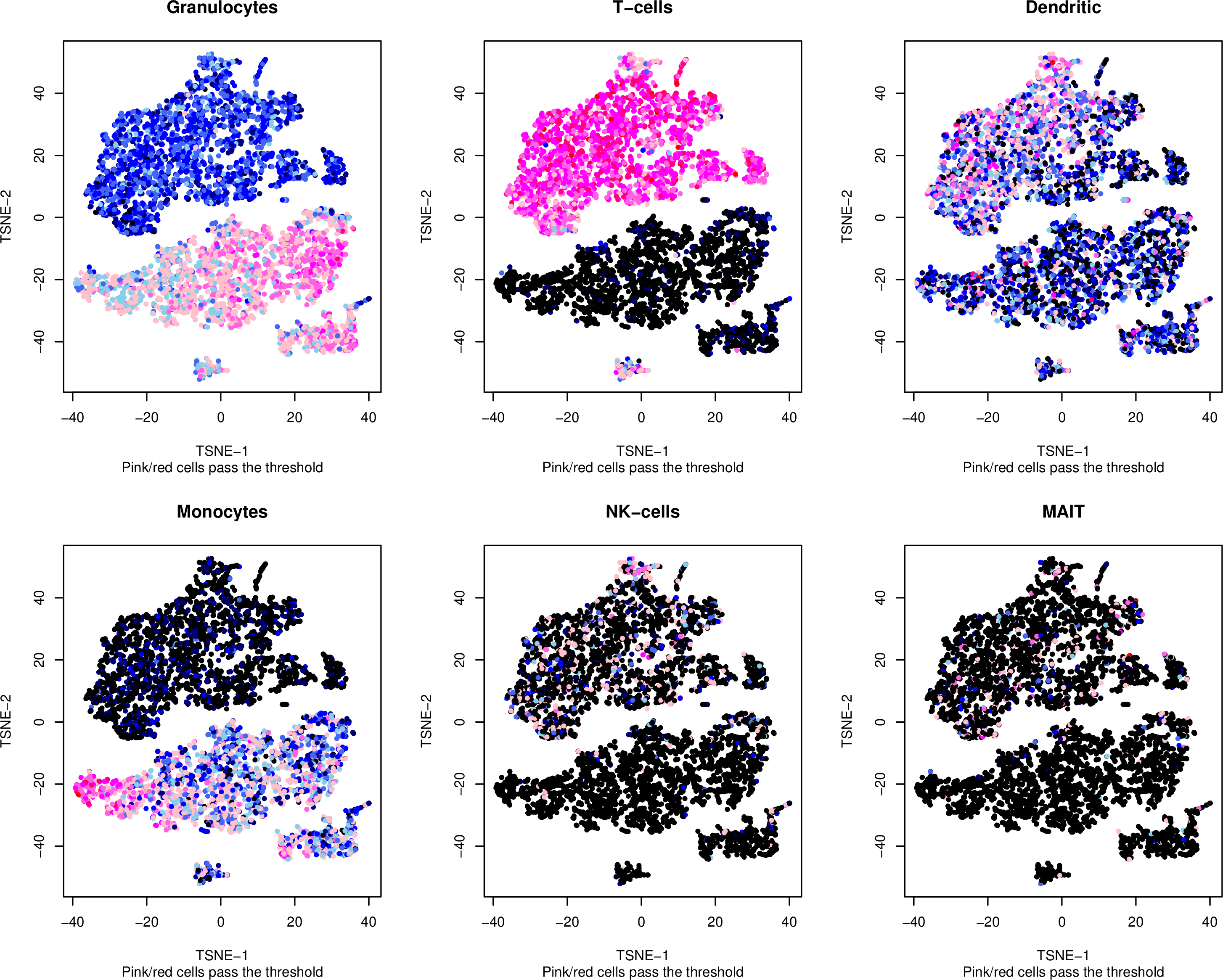

Supplement: Supplementary file 2 — Additional file 2: Supplementary file 2. To demonstrate the utility of scQCEA, we apply the workflow to the sixteen gene expression profiles of eight patients with metastatic melanoma, prepared from pre- and post-treatment experimental batches. You can find the QC interactive report at: https://github.com/isarnassiri/scQCEA/tree/Example-of-Application. Download and unzip the OGC_Interactive_QC_Report_P180121.zip file. You can open CLICK_ME.html file without using rStudio/R. [file 12864_2023_9447_MOESM2_ESM.zip › Inputs/10X-gex-grouped/FAI5649A17/P180121-keep_FAI5649A17_tSNE_Plot.png]

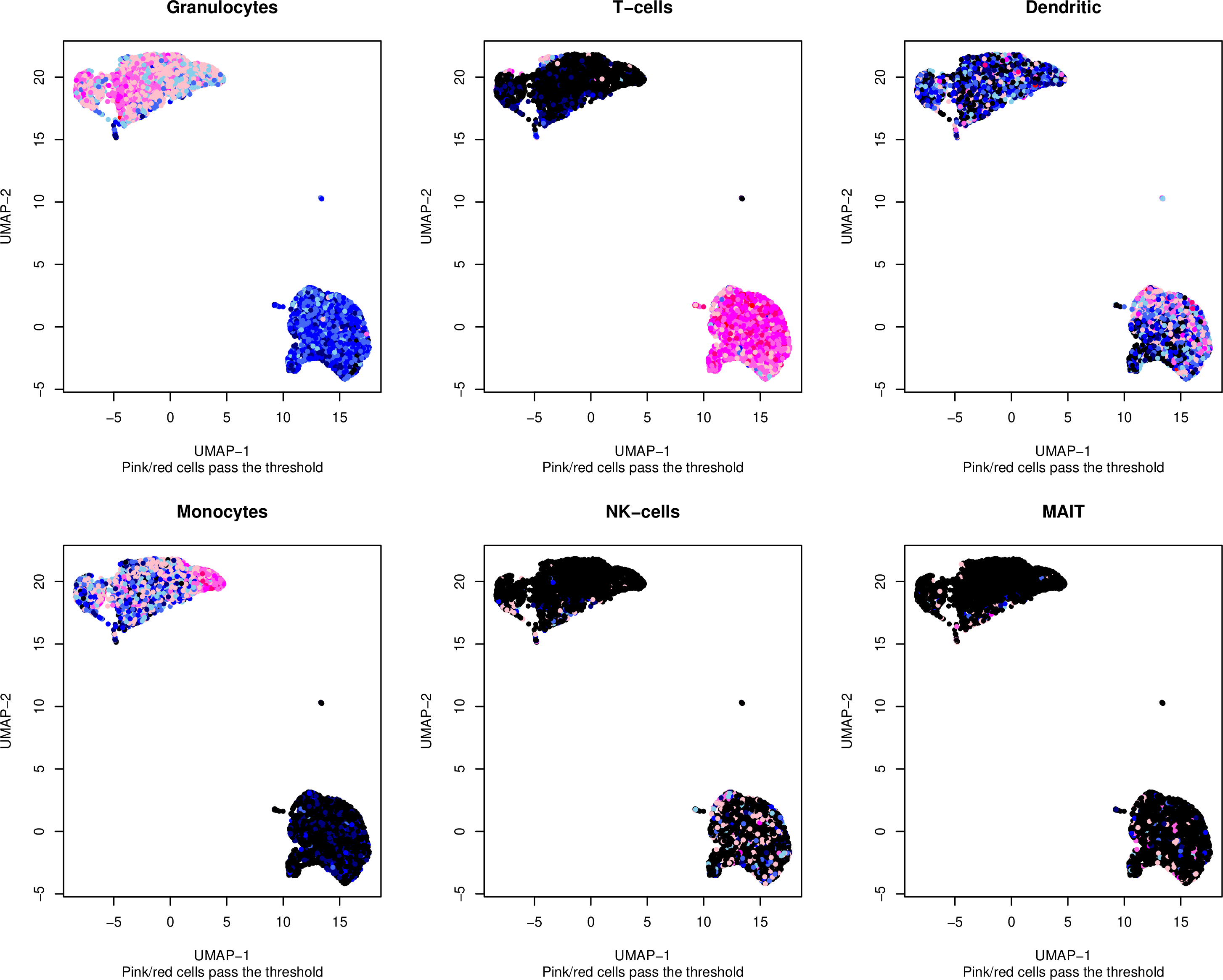

Supplement: Supplementary file 2 — Additional file 2: Supplementary file 2. To demonstrate the utility of scQCEA, we apply the workflow to the sixteen gene expression profiles of eight patients with metastatic melanoma, prepared from pre- and post-treatment experimental batches. You can find the QC interactive report at: https://github.com/isarnassiri/scQCEA/tree/Example-of-Application. Download and unzip the OGC_Interactive_QC_Report_P180121.zip file. You can open CLICK_ME.html file without using rStudio/R. [file 12864_2023_9447_MOESM2_ESM.zip › Inputs/10X-gex-grouped/FAI5649A17/P180121-keep_FAI5649A17_UMAP_Plot.png]

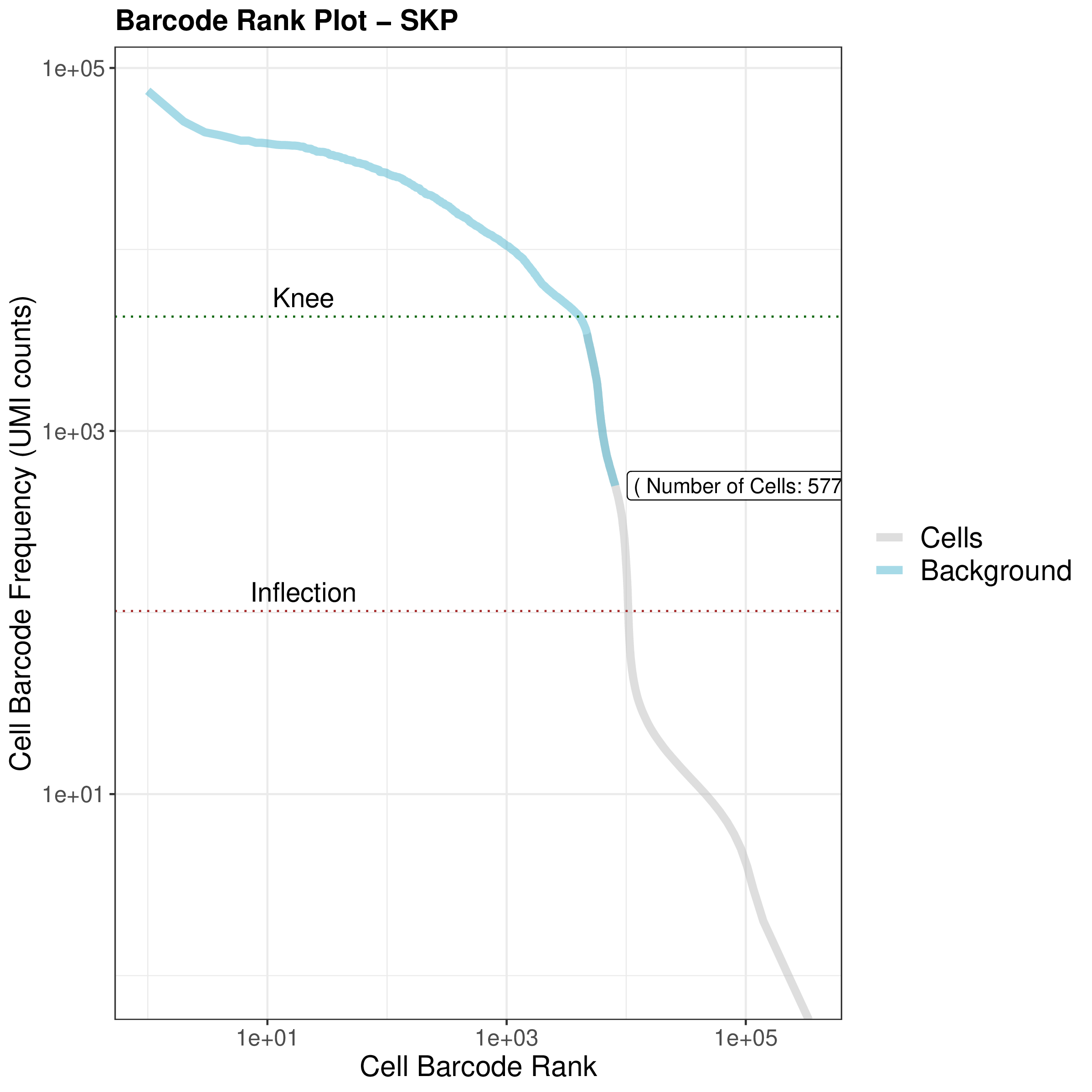

Supplement: Supplementary file 2 — Additional file 2: Supplementary file 2. To demonstrate the utility of scQCEA, we apply the workflow to the sixteen gene expression profiles of eight patients with metastatic melanoma, prepared from pre- and post-treatment experimental batches. You can find the QC interactive report at: https://github.com/isarnassiri/scQCEA/tree/Example-of-Application. Download and unzip the OGC_Interactive_QC_Report_P180121.zip file. You can open CLICK_ME.html file without using rStudio/R. [file 12864_2023_9447_MOESM2_ESM.zip › Inputs/10X-gex-grouped/FAI5649A18/P180121-keep_FAI5649A18_BarcodeRankPlot_10X.png]

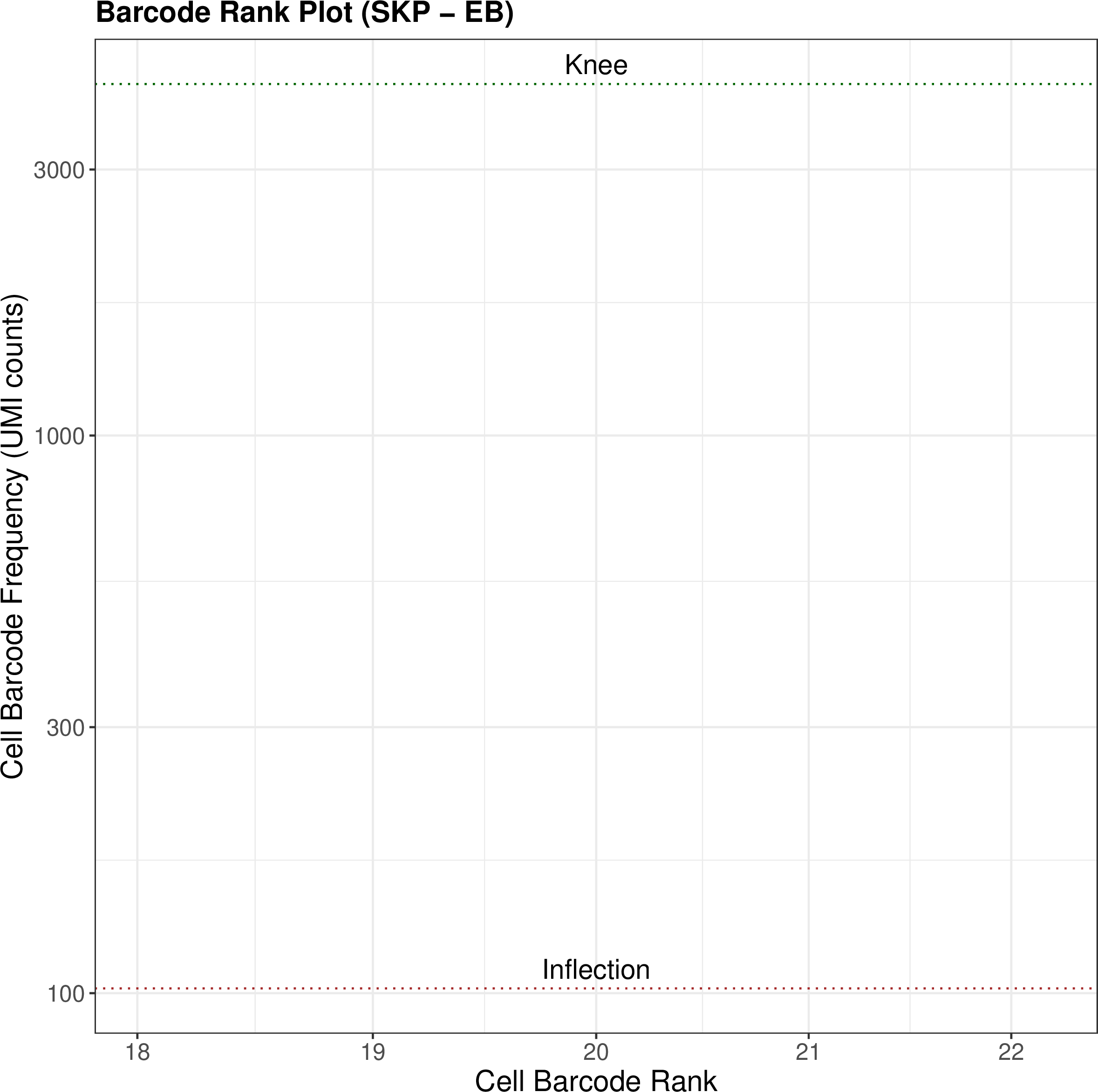

Supplement: Supplementary file 2 — Additional file 2: Supplementary file 2. To demonstrate the utility of scQCEA, we apply the workflow to the sixteen gene expression profiles of eight patients with metastatic melanoma, prepared from pre- and post-treatment experimental batches. You can find the QC interactive report at: https://github.com/isarnassiri/scQCEA/tree/Example-of-Application. Download and unzip the OGC_Interactive_QC_Report_P180121.zip file. You can open CLICK_ME.html file without using rStudio/R. [file 12864_2023_9447_MOESM2_ESM.zip › Inputs/10X-gex-grouped/FAI5649A18/P180121-keep_FAI5649A18_BarcodeRankPlot_EB_FilterOut.png]

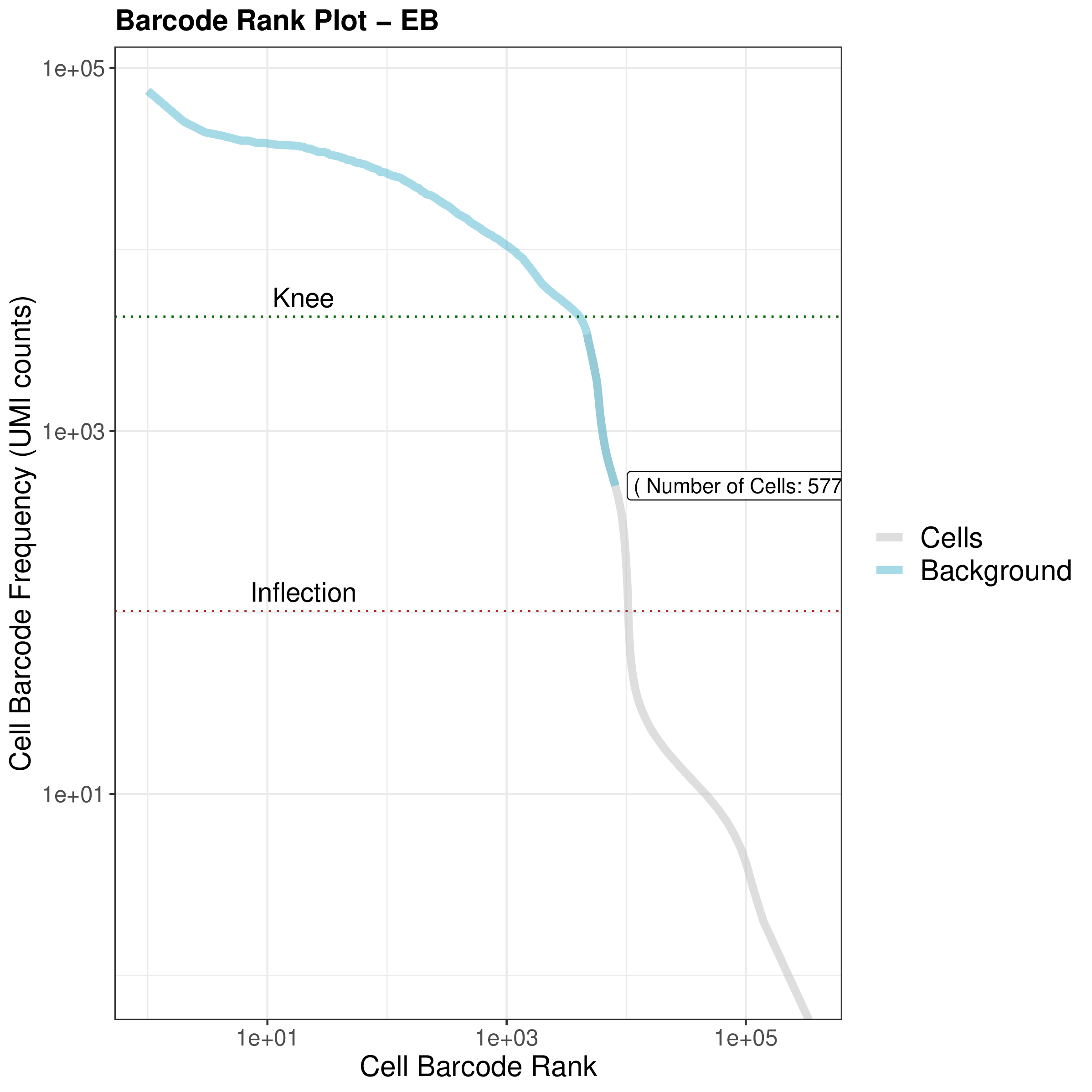

Supplement: Supplementary file 2 — Additional file 2: Supplementary file 2. To demonstrate the utility of scQCEA, we apply the workflow to the sixteen gene expression profiles of eight patients with metastatic melanoma, prepared from pre- and post-treatment experimental batches. You can find the QC interactive report at: https://github.com/isarnassiri/scQCEA/tree/Example-of-Application. Download and unzip the OGC_Interactive_QC_Report_P180121.zip file. You can open CLICK_ME.html file without using rStudio/R. [file 12864_2023_9447_MOESM2_ESM.zip › Inputs/10X-gex-grouped/FAI5649A18/P180121-keep_FAI5649A18_BarcodeRankPlot_EB.png]

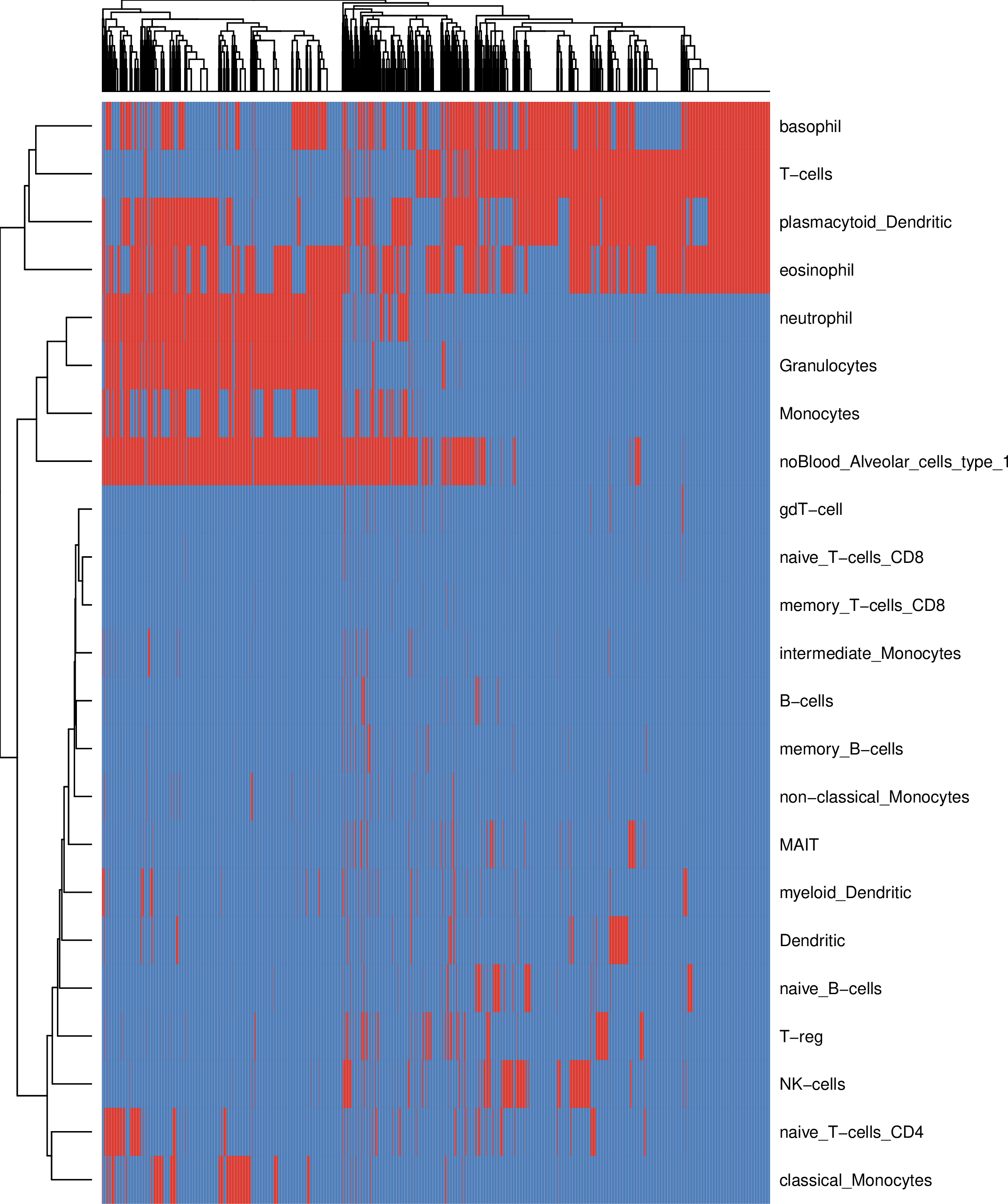

Supplement: Supplementary file 2 — Additional file 2: Supplementary file 2. To demonstrate the utility of scQCEA, we apply the workflow to the sixteen gene expression profiles of eight patients with metastatic melanoma, prepared from pre- and post-treatment experimental batches. You can find the QC interactive report at: https://github.com/isarnassiri/scQCEA/tree/Example-of-Application. Download and unzip the OGC_Interactive_QC_Report_P180121.zip file. You can open CLICK_ME.html file without using rStudio/R. [file 12864_2023_9447_MOESM2_ESM.zip › Inputs/10X-gex-grouped/FAI5649A18/P180121-keep_FAI5649A18_Celltype_assignment_HeatMap.png]

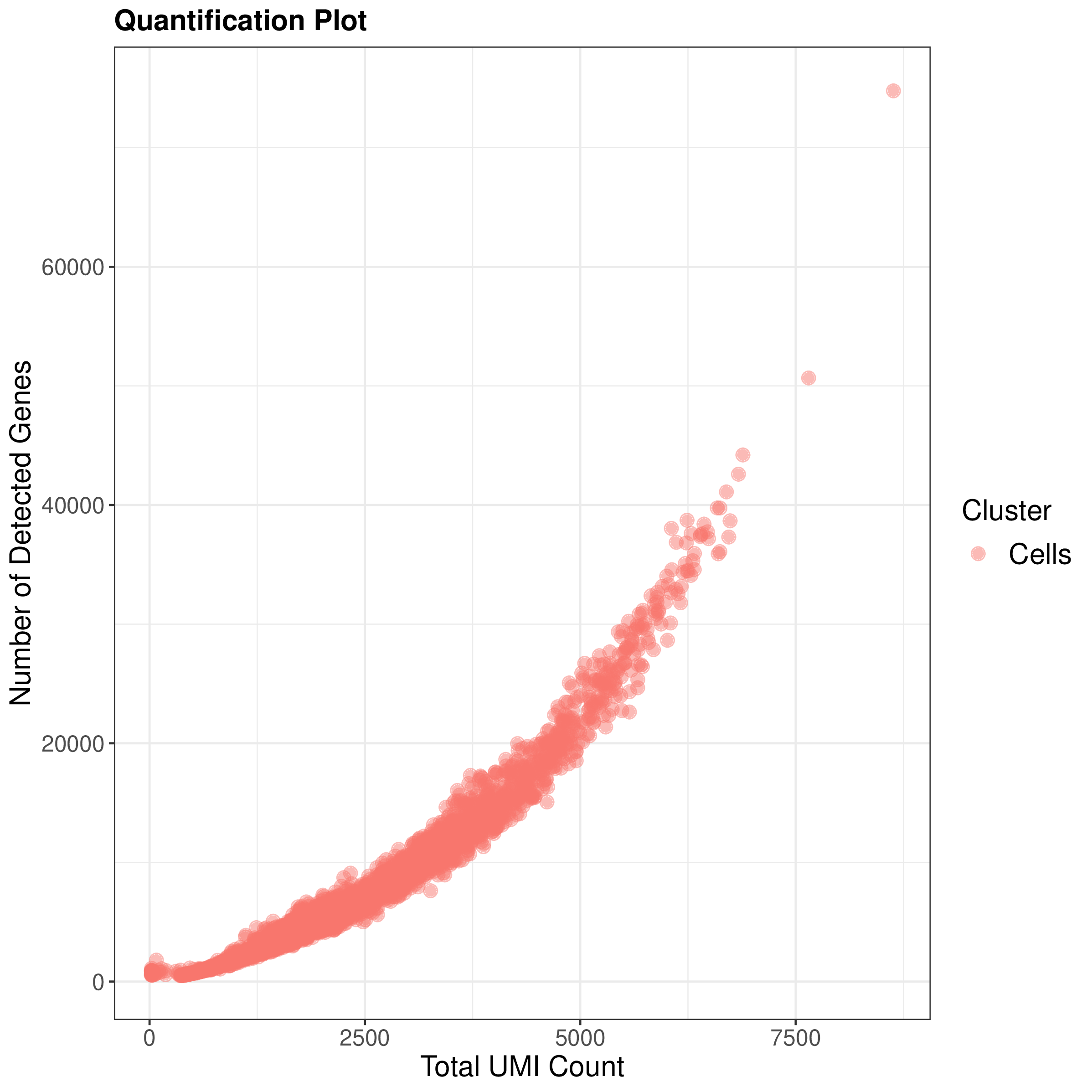

Supplement: Supplementary file 2 — Additional file 2: Supplementary file 2. To demonstrate the utility of scQCEA, we apply the workflow to the sixteen gene expression profiles of eight patients with metastatic melanoma, prepared from pre- and post-treatment experimental batches. You can find the QC interactive report at: https://github.com/isarnassiri/scQCEA/tree/Example-of-Application. Download and unzip the OGC_Interactive_QC_Report_P180121.zip file. You can open CLICK_ME.html file without using rStudio/R. [file 12864_2023_9447_MOESM2_ESM.zip › Inputs/10X-gex-grouped/FAI5649A18/P180121-keep_FAI5649A18_TotalUMIvsDetectedGenes.png]

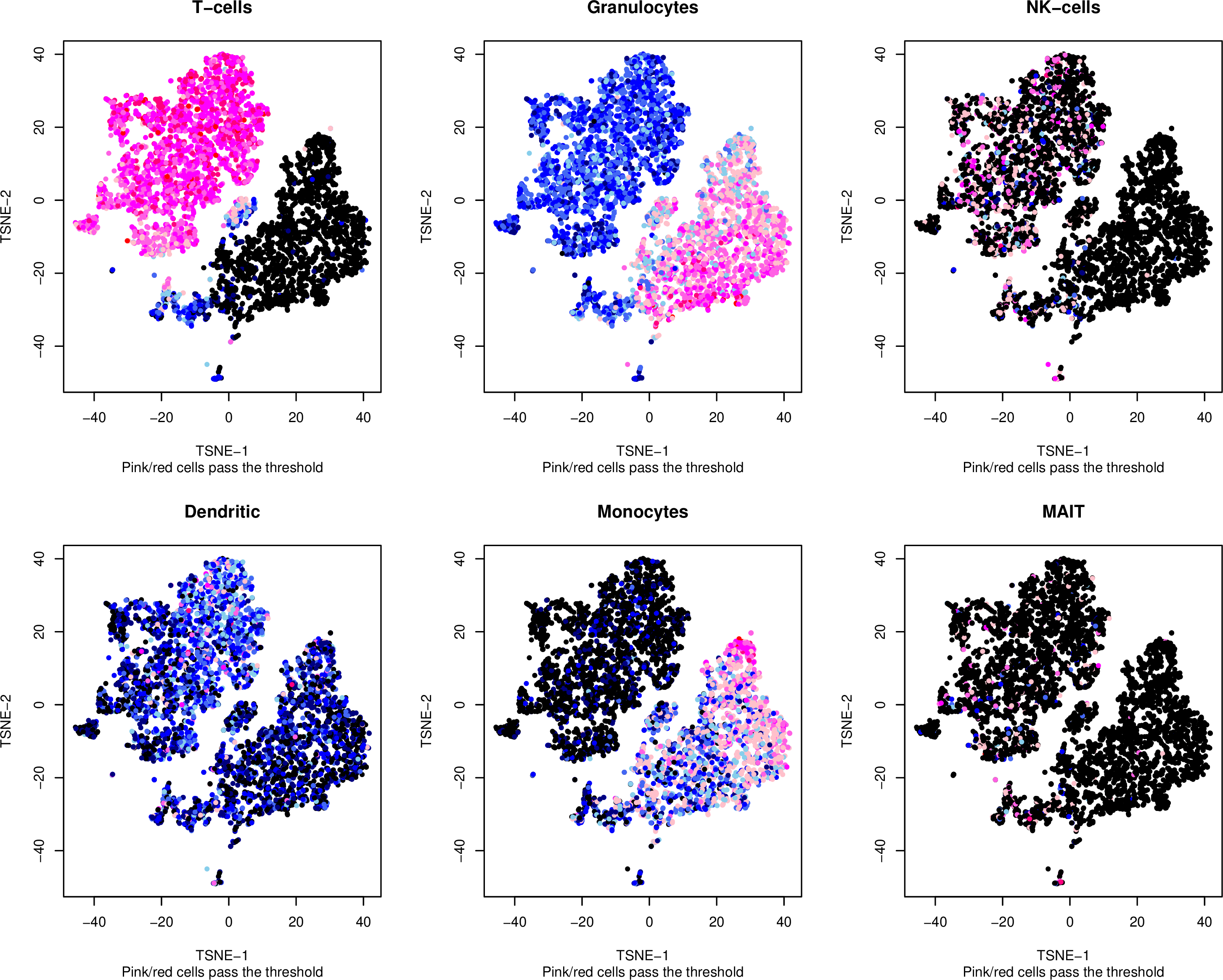

Supplement: Supplementary file 2 — Additional file 2: Supplementary file 2. To demonstrate the utility of scQCEA, we apply the workflow to the sixteen gene expression profiles of eight patients with metastatic melanoma, prepared from pre- and post-treatment experimental batches. You can find the QC interactive report at: https://github.com/isarnassiri/scQCEA/tree/Example-of-Application. Download and unzip the OGC_Interactive_QC_Report_P180121.zip file. You can open CLICK_ME.html file without using rStudio/R. [file 12864_2023_9447_MOESM2_ESM.zip › Inputs/10X-gex-grouped/FAI5649A18/P180121-keep_FAI5649A18_tSNE_Plot.png]

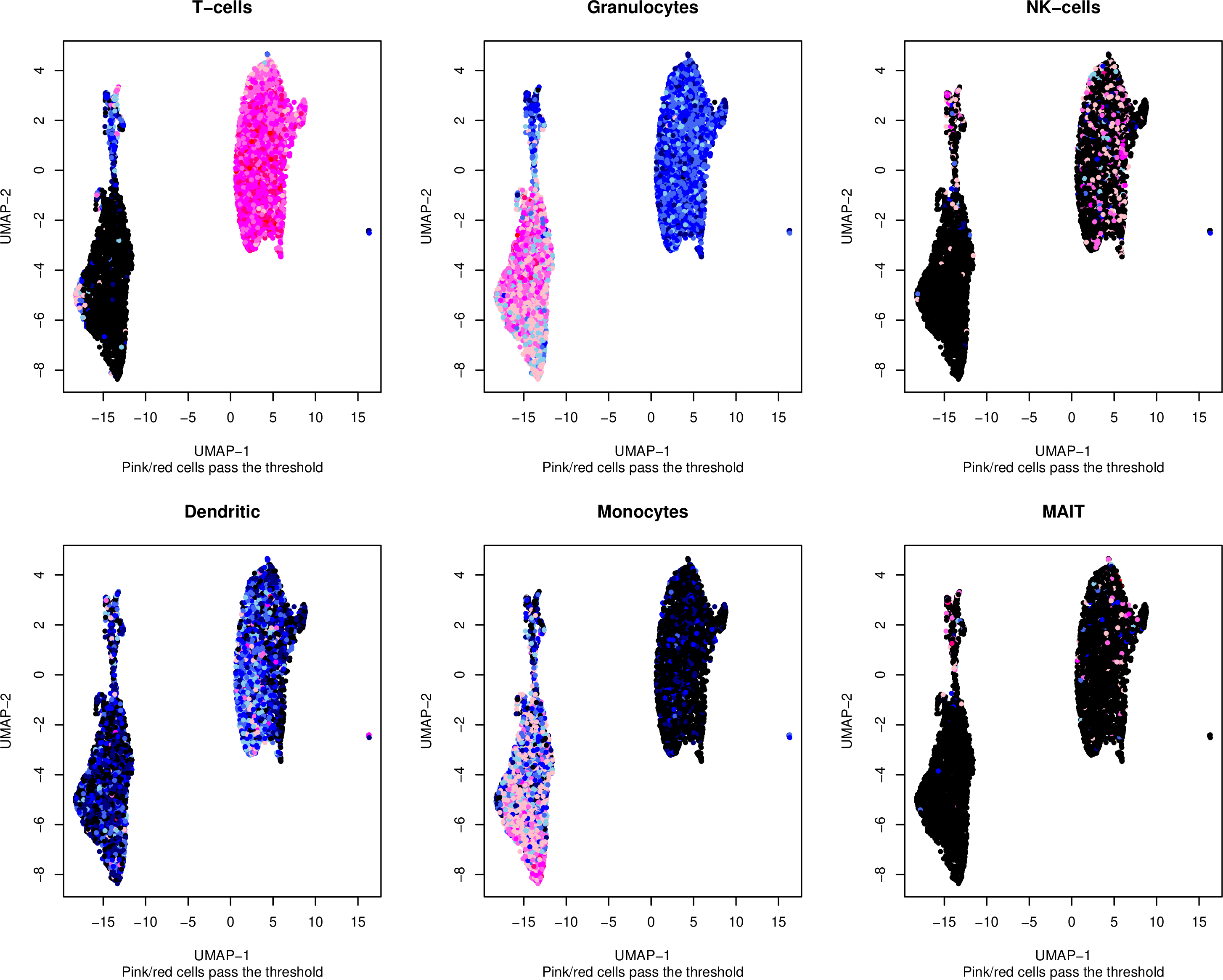

Supplement: Supplementary file 2 — Additional file 2: Supplementary file 2. To demonstrate the utility of scQCEA, we apply the workflow to the sixteen gene expression profiles of eight patients with metastatic melanoma, prepared from pre- and post-treatment experimental batches. You can find the QC interactive report at: https://github.com/isarnassiri/scQCEA/tree/Example-of-Application. Download and unzip the OGC_Interactive_QC_Report_P180121.zip file. You can open CLICK_ME.html file without using rStudio/R. [file 12864_2023_9447_MOESM2_ESM.zip › Inputs/10X-gex-grouped/FAI5649A18/P180121-keep_FAI5649A18_UMAP_Plot.png]

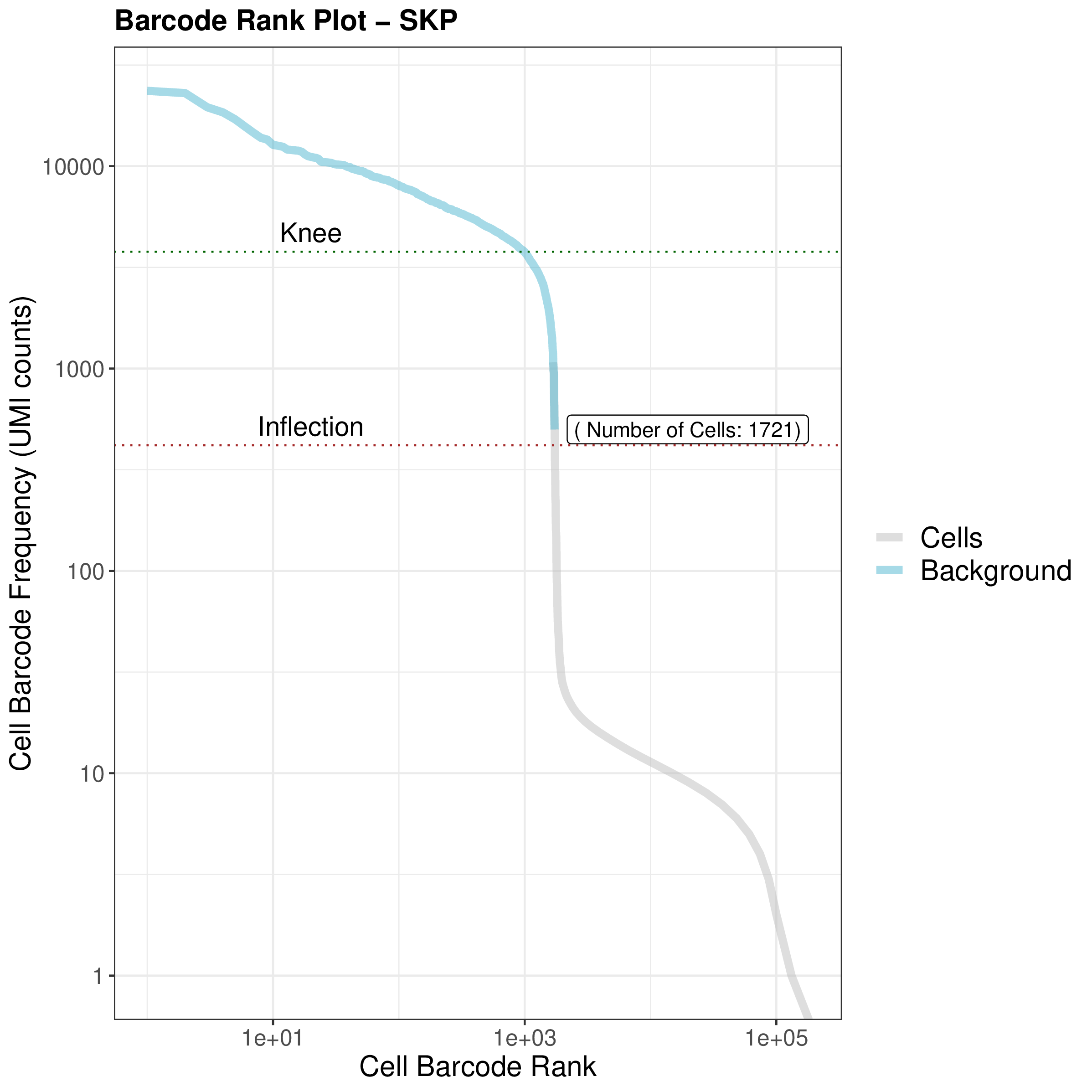

Supplement: Supplementary file 2 — Additional file 2: Supplementary file 2. To demonstrate the utility of scQCEA, we apply the workflow to the sixteen gene expression profiles of eight patients with metastatic melanoma, prepared from pre- and post-treatment experimental batches. You can find the QC interactive report at: https://github.com/isarnassiri/scQCEA/tree/Example-of-Application. Download and unzip the OGC_Interactive_QC_Report_P180121.zip file. You can open CLICK_ME.html file without using rStudio/R. [file 12864_2023_9447_MOESM2_ESM.zip › Inputs/10X-gex-grouped/FAI5649A19/P180121-keep_FAI5649A19_BarcodeRankPlot_10X.png]

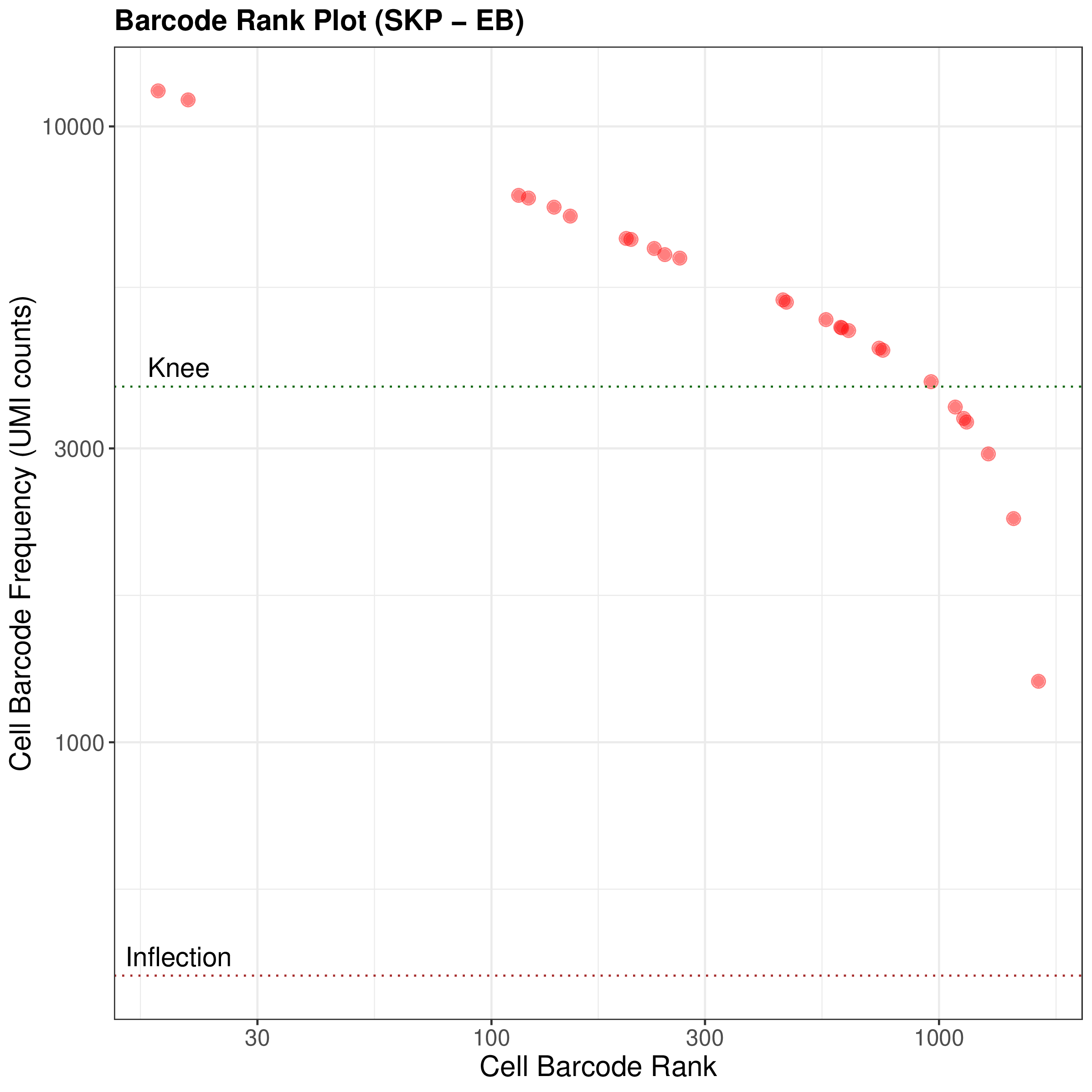

Supplement: Supplementary file 2 — Additional file 2: Supplementary file 2. To demonstrate the utility of scQCEA, we apply the workflow to the sixteen gene expression profiles of eight patients with metastatic melanoma, prepared from pre- and post-treatment experimental batches. You can find the QC interactive report at: https://github.com/isarnassiri/scQCEA/tree/Example-of-Application. Download and unzip the OGC_Interactive_QC_Report_P180121.zip file. You can open CLICK_ME.html file without using rStudio/R. [file 12864_2023_9447_MOESM2_ESM.zip › Inputs/10X-gex-grouped/FAI5649A19/P180121-keep_FAI5649A19_BarcodeRankPlot_EB_FilterOut.png]

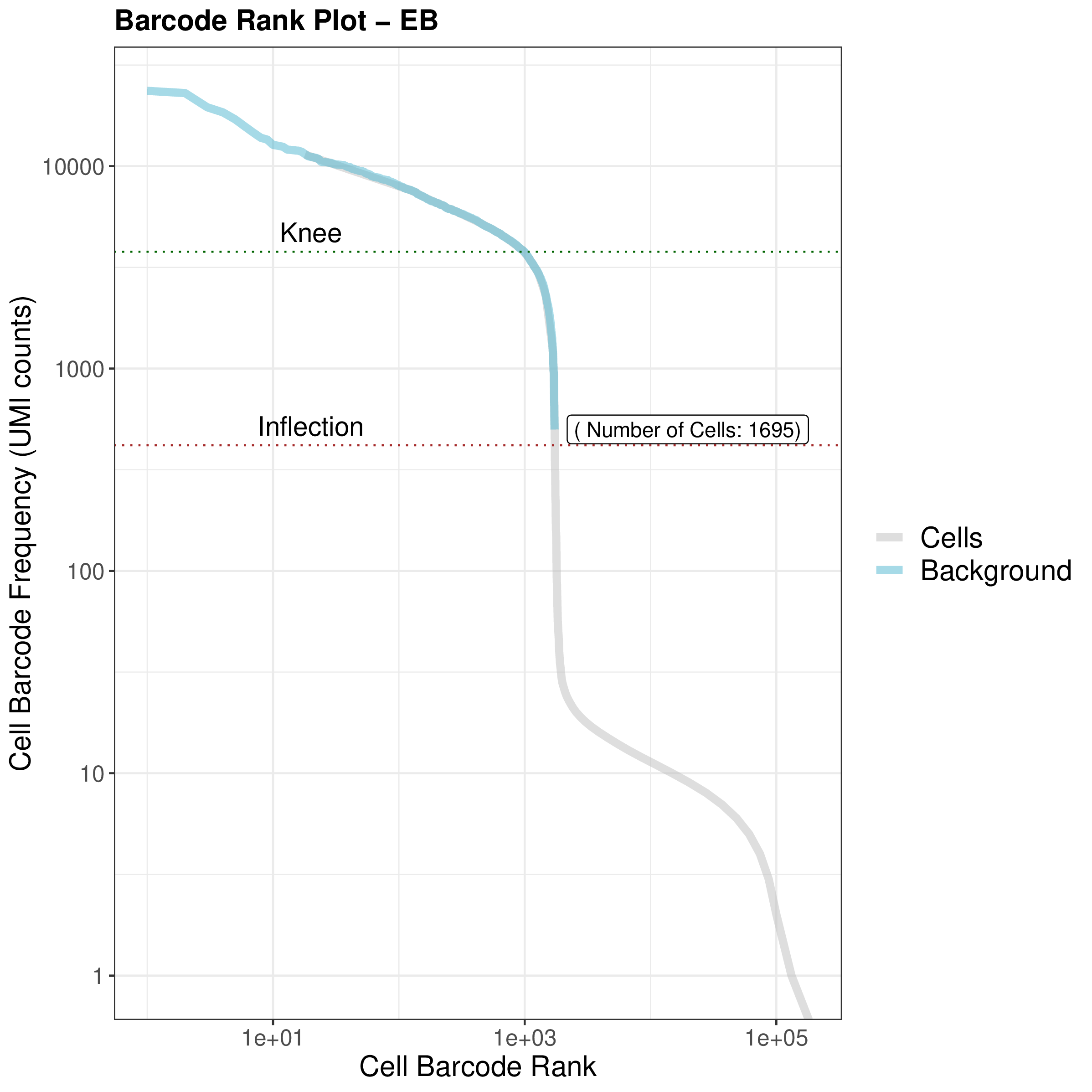

Supplement: Supplementary file 2 — Additional file 2: Supplementary file 2. To demonstrate the utility of scQCEA, we apply the workflow to the sixteen gene expression profiles of eight patients with metastatic melanoma, prepared from pre- and post-treatment experimental batches. You can find the QC interactive report at: https://github.com/isarnassiri/scQCEA/tree/Example-of-Application. Download and unzip the OGC_Interactive_QC_Report_P180121.zip file. You can open CLICK_ME.html file without using rStudio/R. [file 12864_2023_9447_MOESM2_ESM.zip › Inputs/10X-gex-grouped/FAI5649A19/P180121-keep_FAI5649A19_BarcodeRankPlot_EB.png]

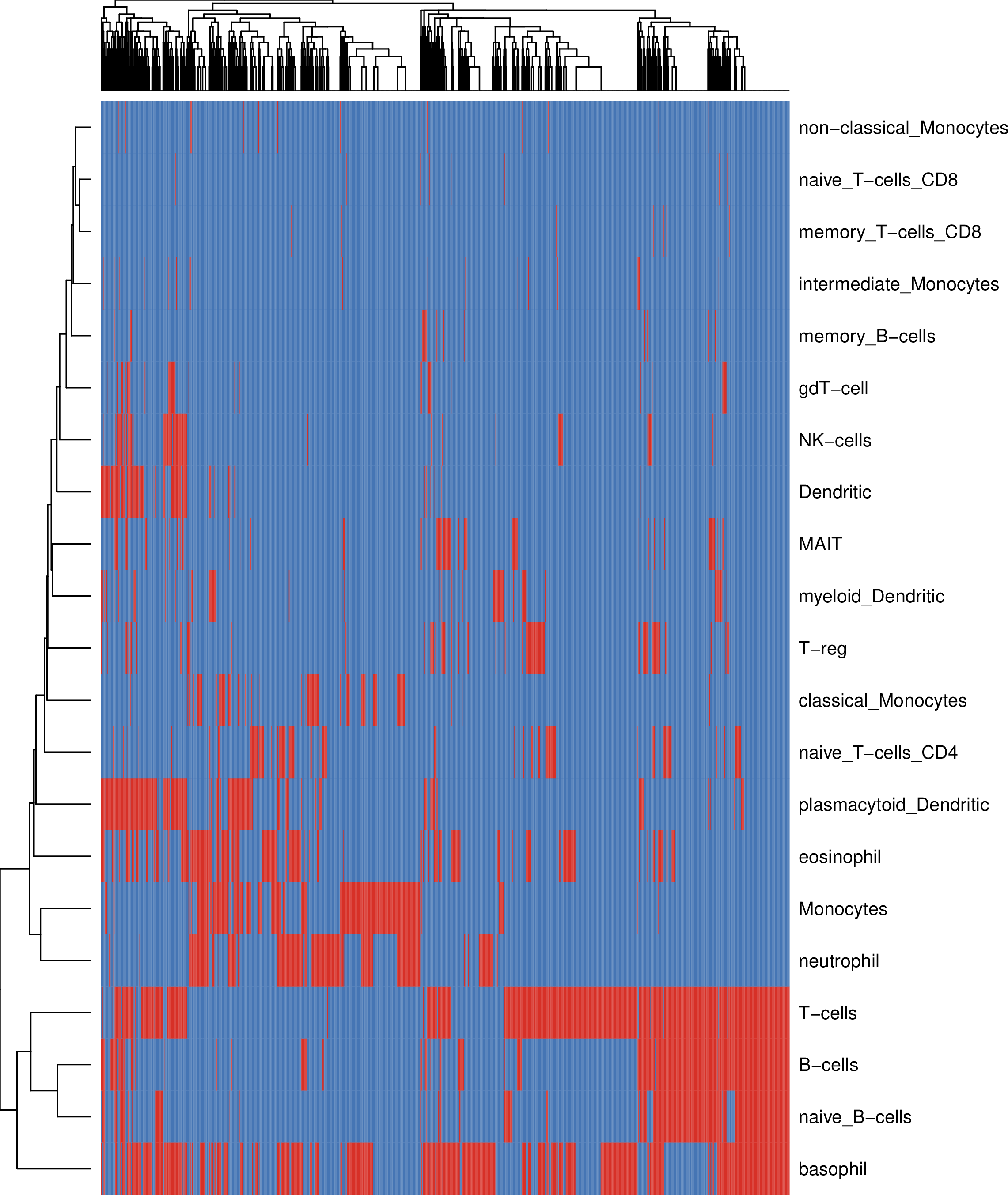

Supplement: Supplementary file 2 — Additional file 2: Supplementary file 2. To demonstrate the utility of scQCEA, we apply the workflow to the sixteen gene expression profiles of eight patients with metastatic melanoma, prepared from pre- and post-treatment experimental batches. You can find the QC interactive report at: https://github.com/isarnassiri/scQCEA/tree/Example-of-Application. Download and unzip the OGC_Interactive_QC_Report_P180121.zip file. You can open CLICK_ME.html file without using rStudio/R. [file 12864_2023_9447_MOESM2_ESM.zip › Inputs/10X-gex-grouped/FAI5649A19/P180121-keep_FAI5649A19_Celltype_assignment_HeatMap.png]

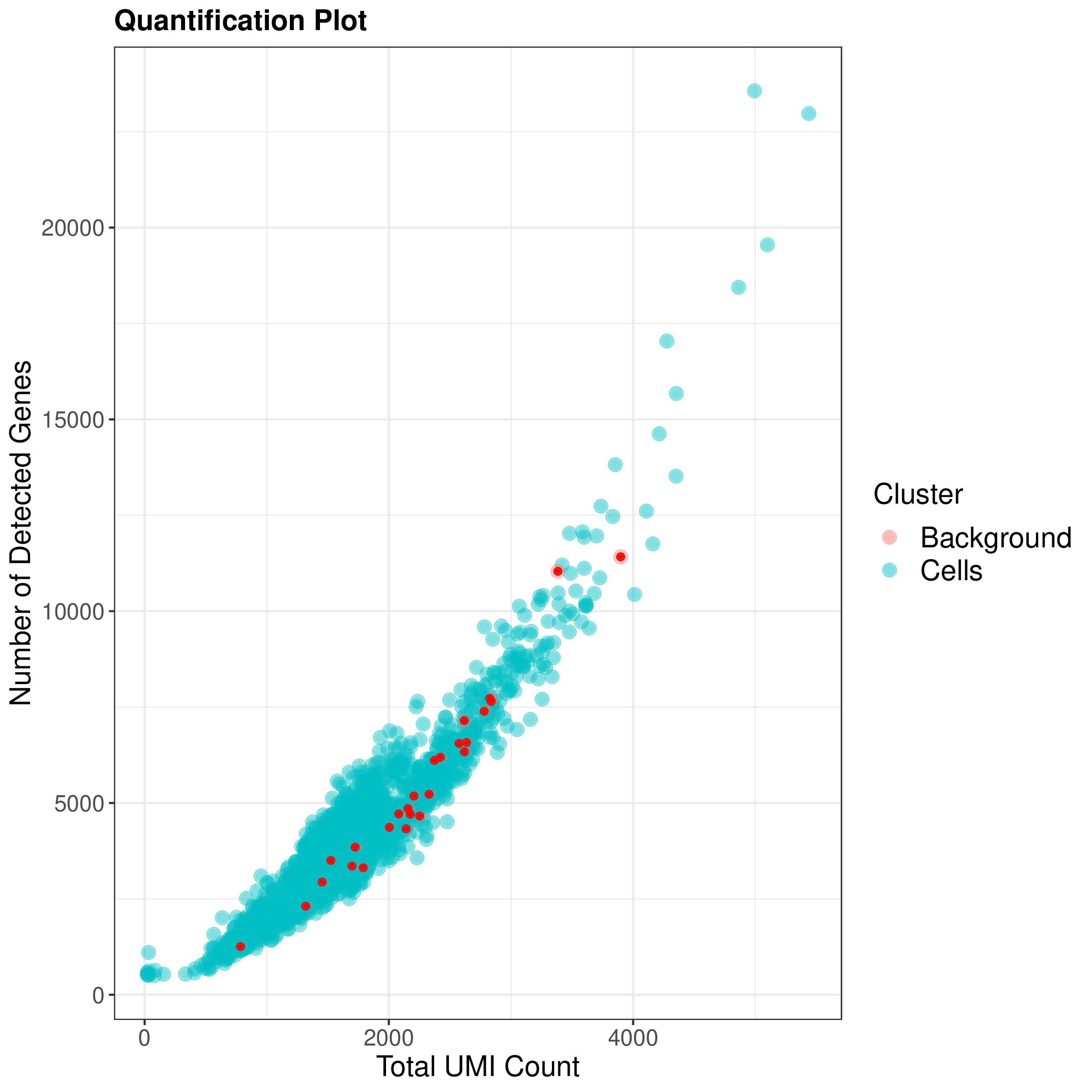

Supplement: Supplementary file 2 — Additional file 2: Supplementary file 2. To demonstrate the utility of scQCEA, we apply the workflow to the sixteen gene expression profiles of eight patients with metastatic melanoma, prepared from pre- and post-treatment experimental batches. You can find the QC interactive report at: https://github.com/isarnassiri/scQCEA/tree/Example-of-Application. Download and unzip the OGC_Interactive_QC_Report_P180121.zip file. You can open CLICK_ME.html file without using rStudio/R. [file 12864_2023_9447_MOESM2_ESM.zip › Inputs/10X-gex-grouped/FAI5649A19/P180121-keep_FAI5649A19_TotalUMIvsDetectedGenes.png]

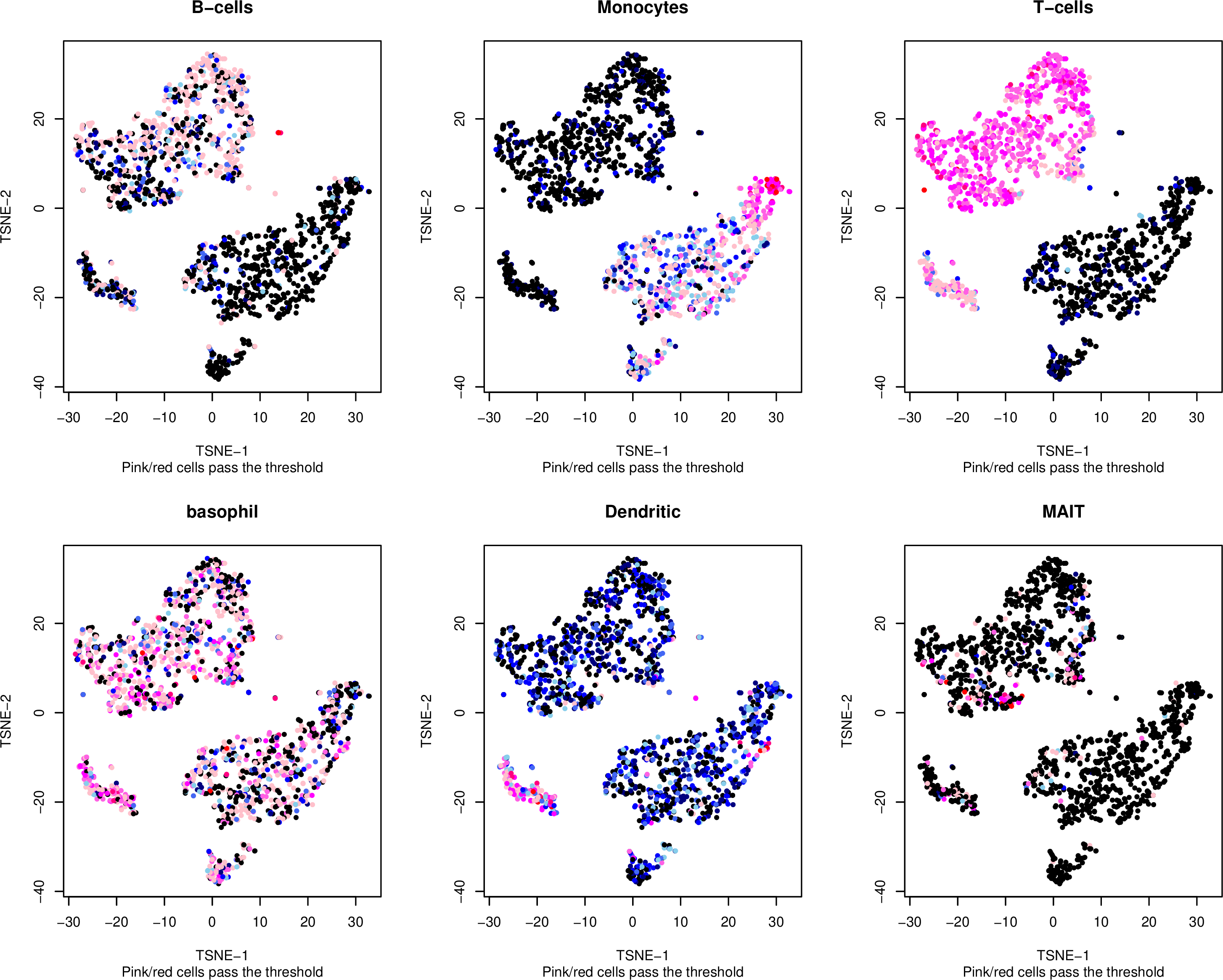

Supplement: Supplementary file 2 — Additional file 2: Supplementary file 2. To demonstrate the utility of scQCEA, we apply the workflow to the sixteen gene expression profiles of eight patients with metastatic melanoma, prepared from pre- and post-treatment experimental batches. You can find the QC interactive report at: https://github.com/isarnassiri/scQCEA/tree/Example-of-Application. Download and unzip the OGC_Interactive_QC_Report_P180121.zip file. You can open CLICK_ME.html file without using rStudio/R. [file 12864_2023_9447_MOESM2_ESM.zip › Inputs/10X-gex-grouped/FAI5649A19/P180121-keep_FAI5649A19_tSNE_Plot.png]

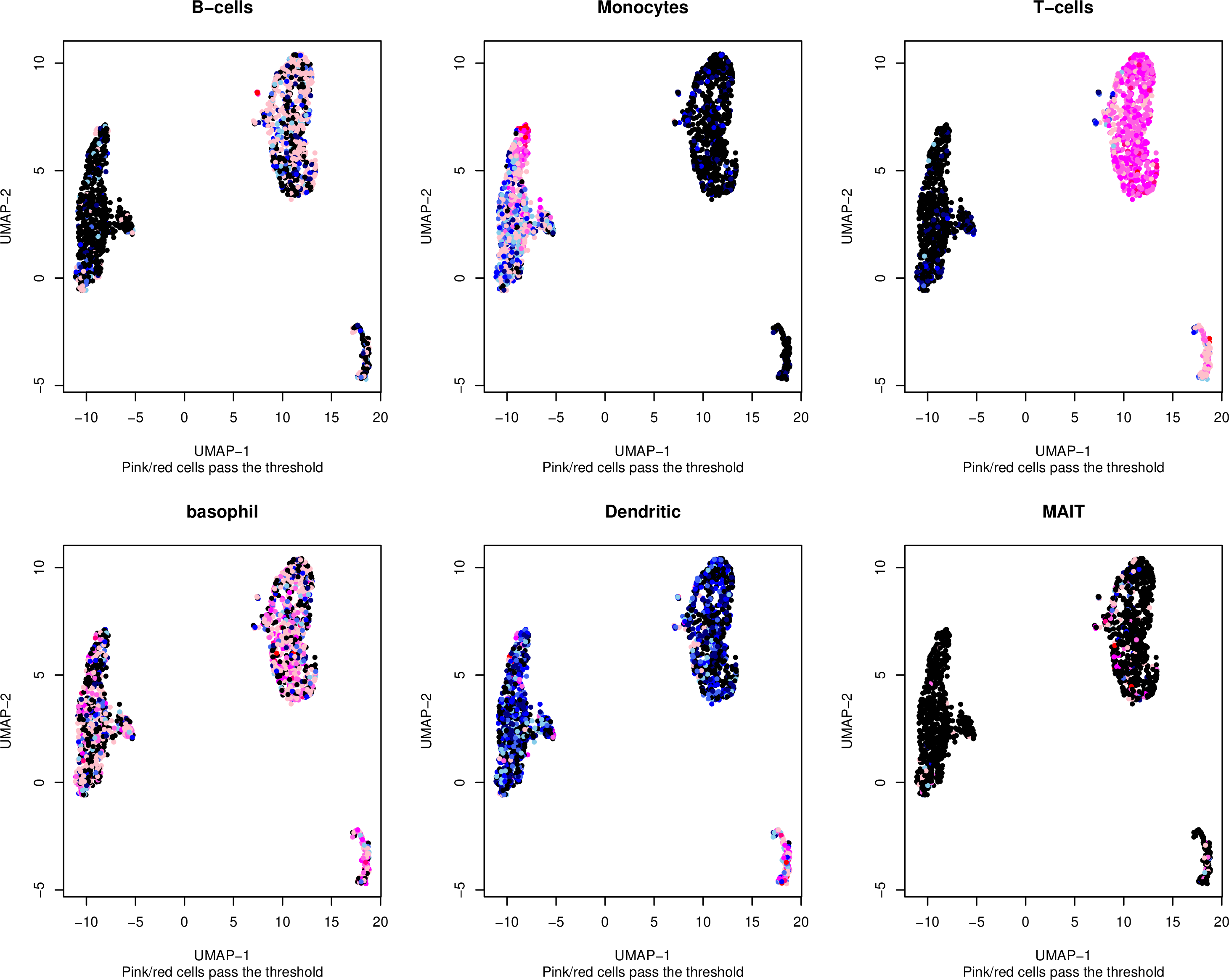

Supplement: Supplementary file 2 — Additional file 2: Supplementary file 2. To demonstrate the utility of scQCEA, we apply the workflow to the sixteen gene expression profiles of eight patients with metastatic melanoma, prepared from pre- and post-treatment experimental batches. You can find the QC interactive report at: https://github.com/isarnassiri/scQCEA/tree/Example-of-Application. Download and unzip the OGC_Interactive_QC_Report_P180121.zip file. You can open CLICK_ME.html file without using rStudio/R. [file 12864_2023_9447_MOESM2_ESM.zip › Inputs/10X-gex-grouped/FAI5649A19/P180121-keep_FAI5649A19_UMAP_Plot.png]

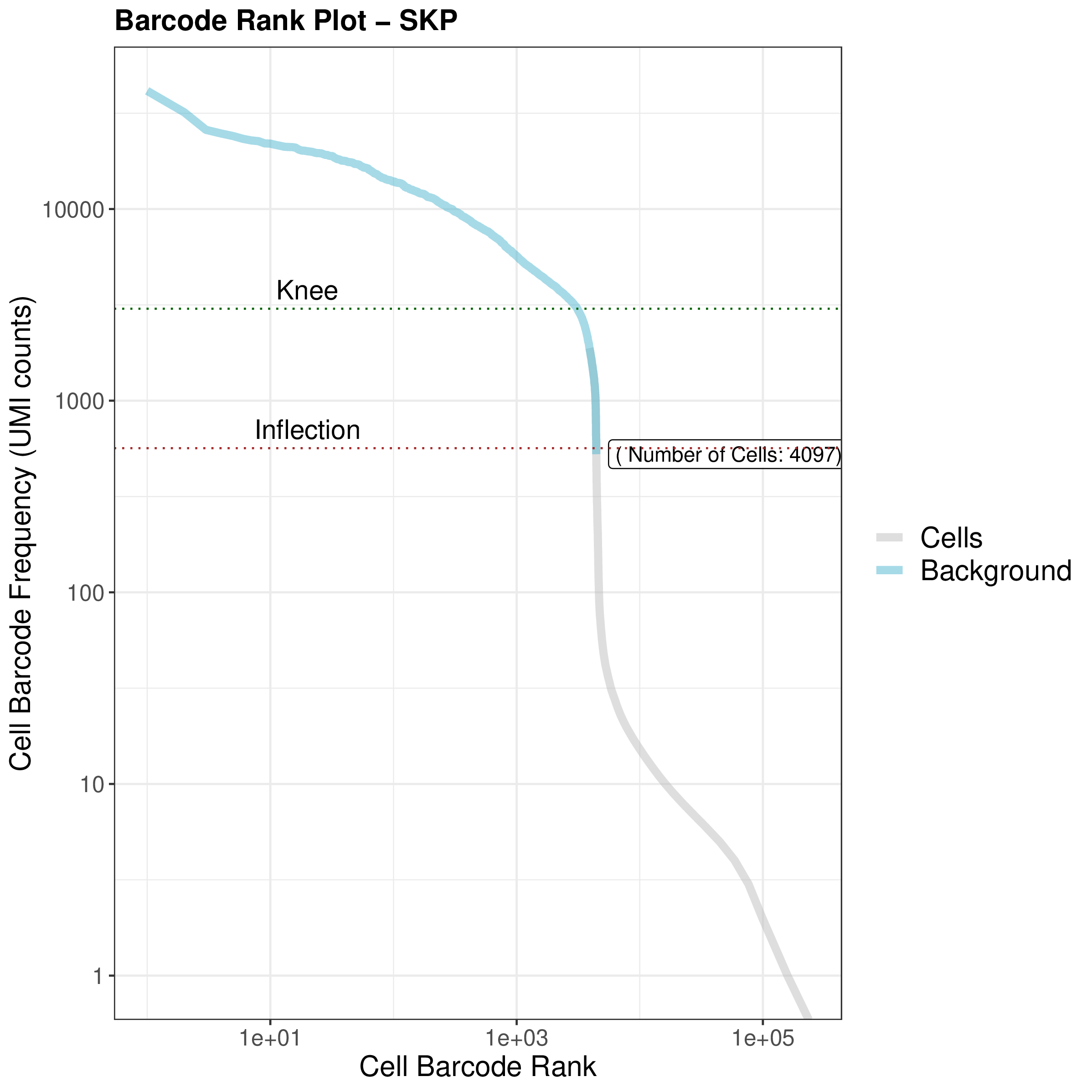

Supplement: Supplementary file 2 — Additional file 2: Supplementary file 2. To demonstrate the utility of scQCEA, we apply the workflow to the sixteen gene expression profiles of eight patients with metastatic melanoma, prepared from pre- and post-treatment experimental batches. You can find the QC interactive report at: https://github.com/isarnassiri/scQCEA/tree/Example-of-Application. Download and unzip the OGC_Interactive_QC_Report_P180121.zip file. You can open CLICK_ME.html file without using rStudio/R. [file 12864_2023_9447_MOESM2_ESM.zip › Inputs/10X-gex-grouped/FAI5649A20/P180121-keep_FAI5649A20_BarcodeRankPlot_10X.png]

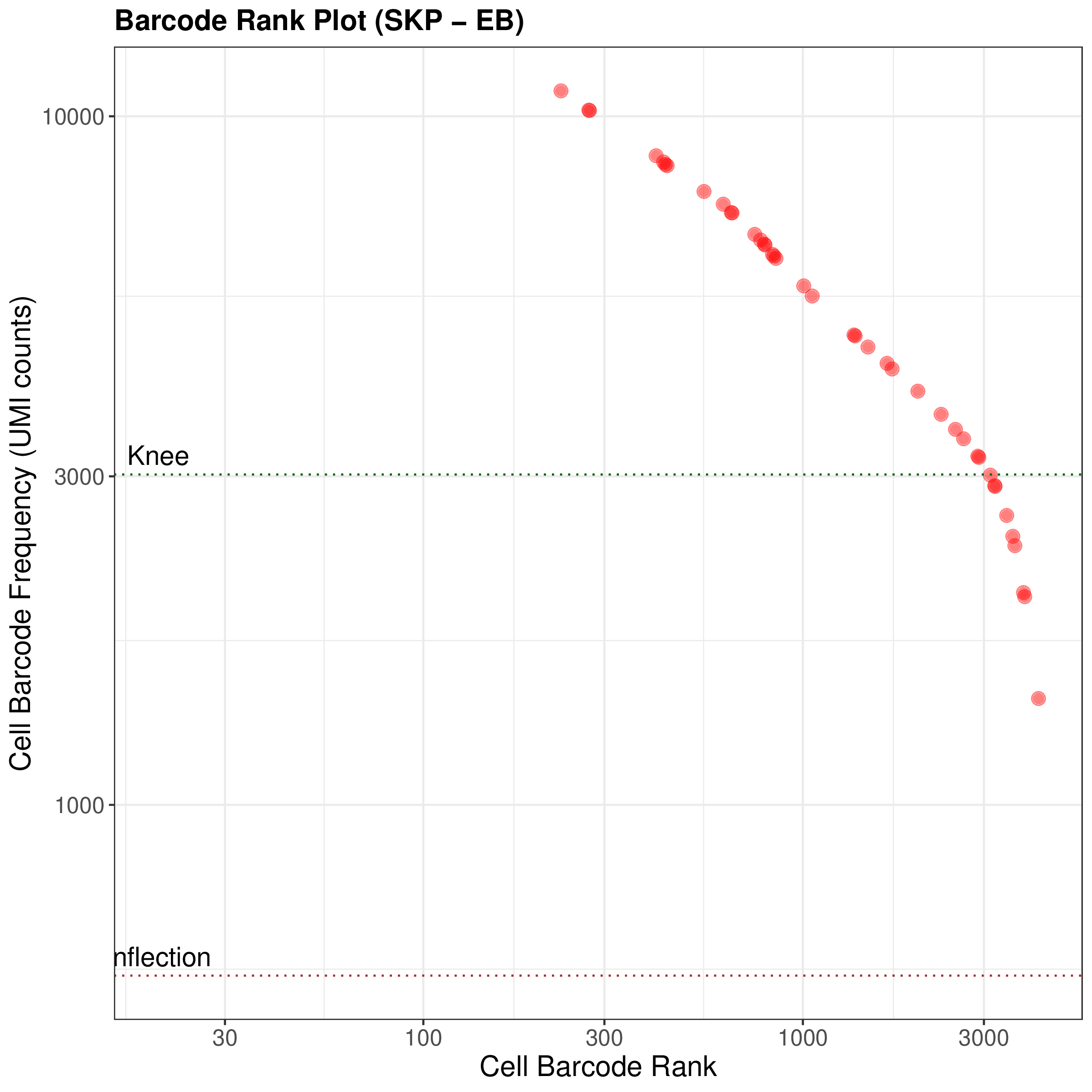

Supplement: Supplementary file 2 — Additional file 2: Supplementary file 2. To demonstrate the utility of scQCEA, we apply the workflow to the sixteen gene expression profiles of eight patients with metastatic melanoma, prepared from pre- and post-treatment experimental batches. You can find the QC interactive report at: https://github.com/isarnassiri/scQCEA/tree/Example-of-Application. Download and unzip the OGC_Interactive_QC_Report_P180121.zip file. You can open CLICK_ME.html file without using rStudio/R. [file 12864_2023_9447_MOESM2_ESM.zip › Inputs/10X-gex-grouped/FAI5649A20/P180121-keep_FAI5649A20_BarcodeRankPlot_EB_FilterOut.png]

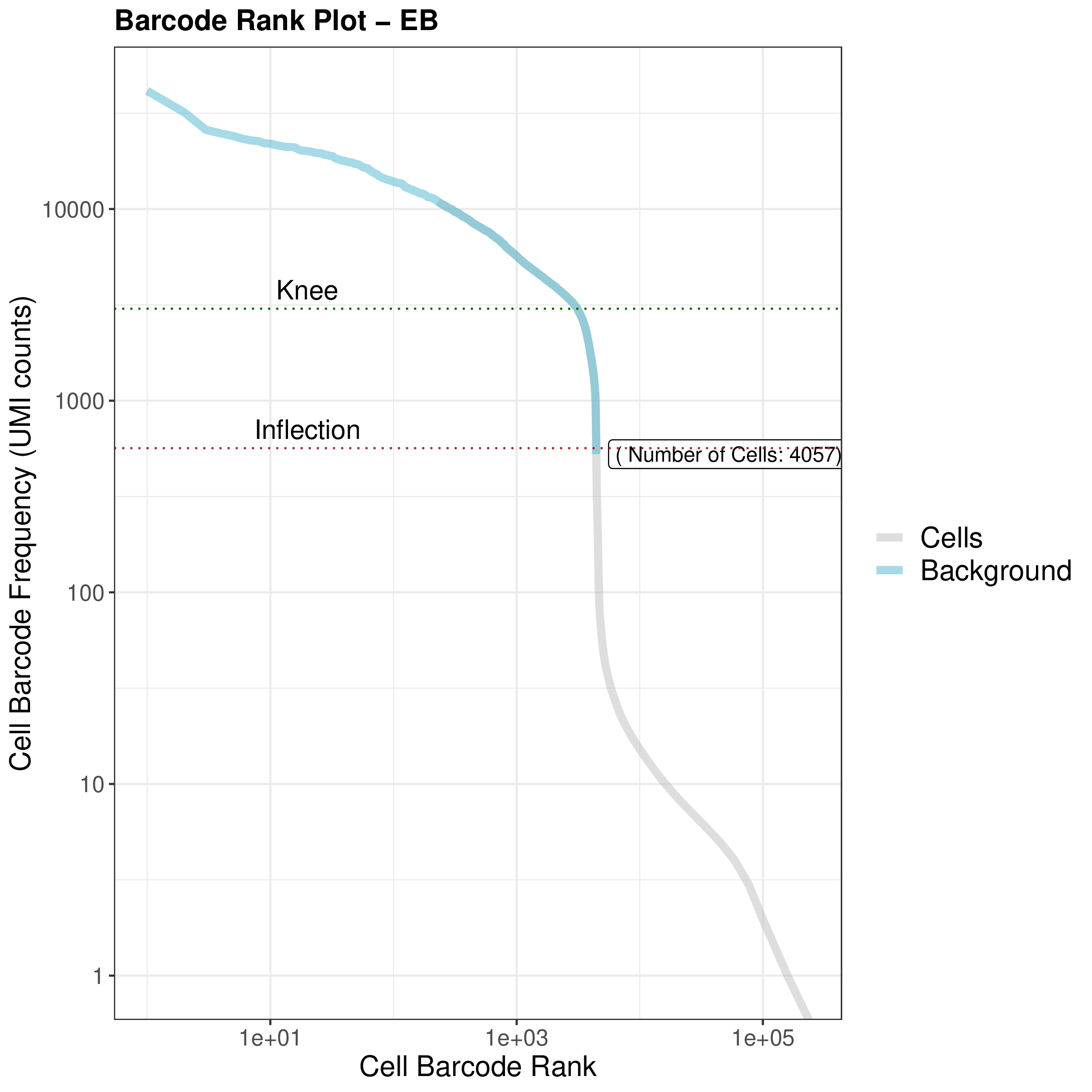

Supplement: Supplementary file 2 — Additional file 2: Supplementary file 2. To demonstrate the utility of scQCEA, we apply the workflow to the sixteen gene expression profiles of eight patients with metastatic melanoma, prepared from pre- and post-treatment experimental batches. You can find the QC interactive report at: https://github.com/isarnassiri/scQCEA/tree/Example-of-Application. Download and unzip the OGC_Interactive_QC_Report_P180121.zip file. You can open CLICK_ME.html file without using rStudio/R. [file 12864_2023_9447_MOESM2_ESM.zip › Inputs/10X-gex-grouped/FAI5649A20/P180121-keep_FAI5649A20_BarcodeRankPlot_EB.png]

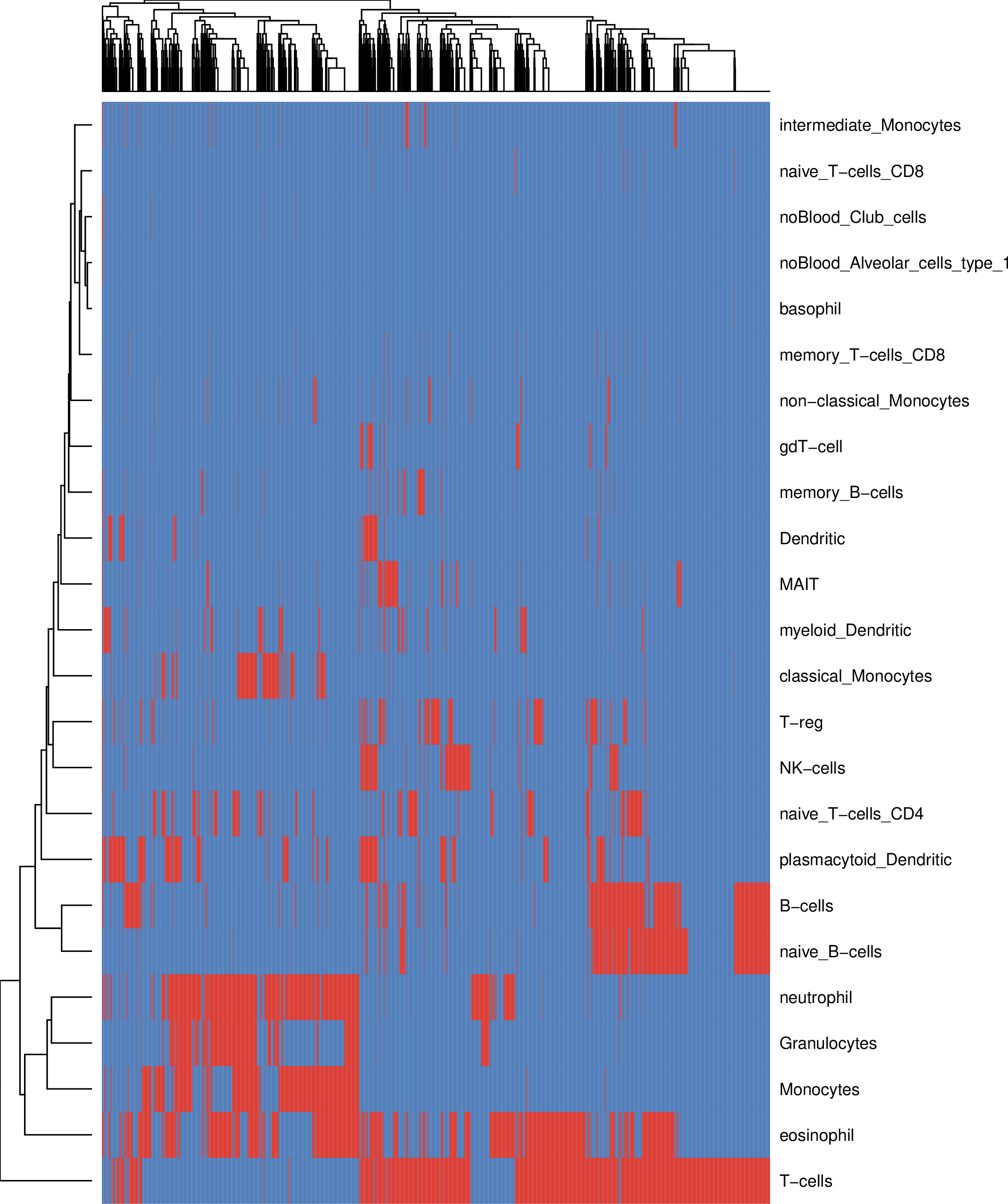

Supplement: Supplementary file 2 — Additional file 2: Supplementary file 2. To demonstrate the utility of scQCEA, we apply the workflow to the sixteen gene expression profiles of eight patients with metastatic melanoma, prepared from pre- and post-treatment experimental batches. You can find the QC interactive report at: https://github.com/isarnassiri/scQCEA/tree/Example-of-Application. Download and unzip the OGC_Interactive_QC_Report_P180121.zip file. You can open CLICK_ME.html file without using rStudio/R. [file 12864_2023_9447_MOESM2_ESM.zip › Inputs/10X-gex-grouped/FAI5649A20/P180121-keep_FAI5649A20_Celltype_assignment_HeatMap.png]

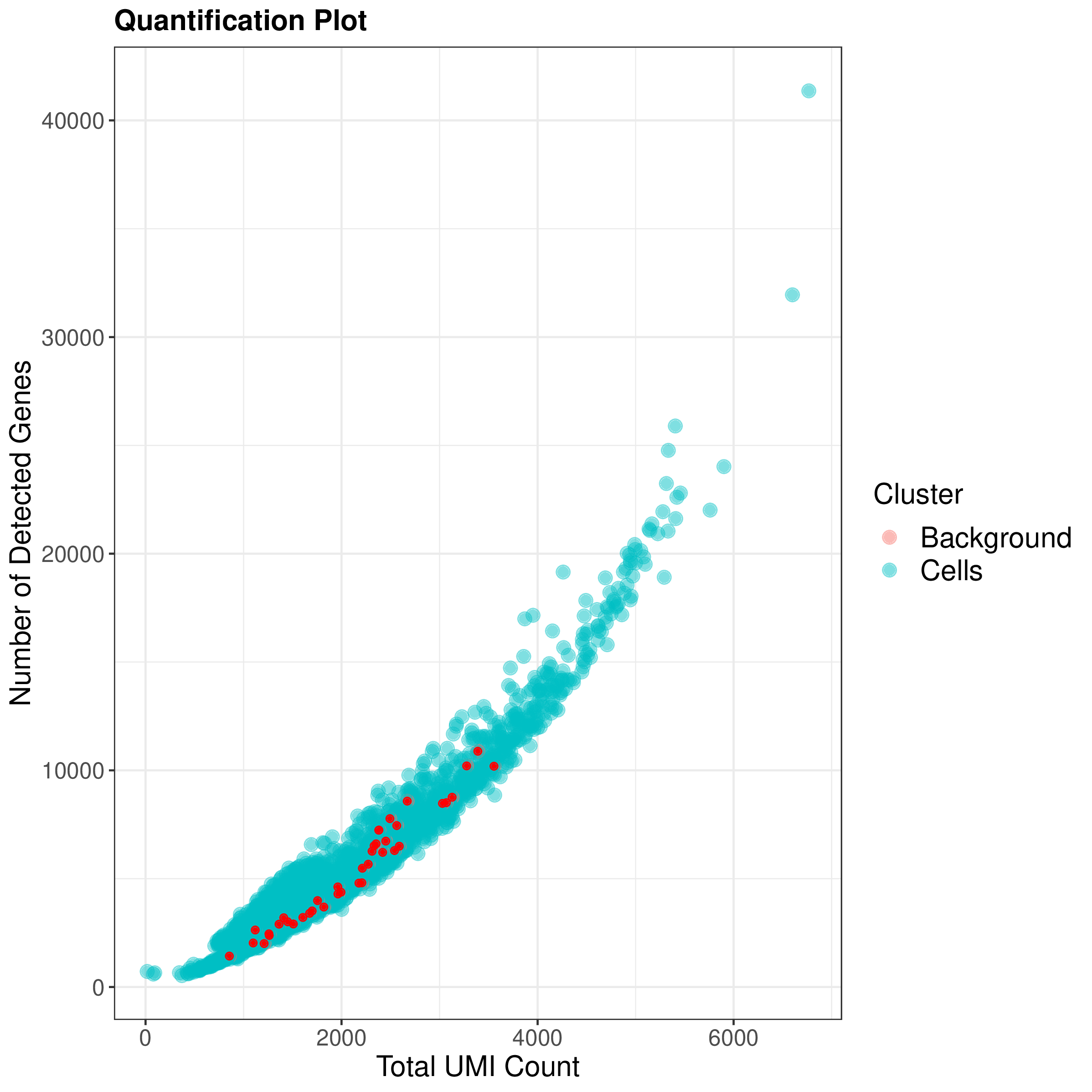

Supplement: Supplementary file 2 — Additional file 2: Supplementary file 2. To demonstrate the utility of scQCEA, we apply the workflow to the sixteen gene expression profiles of eight patients with metastatic melanoma, prepared from pre- and post-treatment experimental batches. You can find the QC interactive report at: https://github.com/isarnassiri/scQCEA/tree/Example-of-Application. Download and unzip the OGC_Interactive_QC_Report_P180121.zip file. You can open CLICK_ME.html file without using rStudio/R. [file 12864_2023_9447_MOESM2_ESM.zip › Inputs/10X-gex-grouped/FAI5649A20/P180121-keep_FAI5649A20_TotalUMIvsDetectedGenes.png]

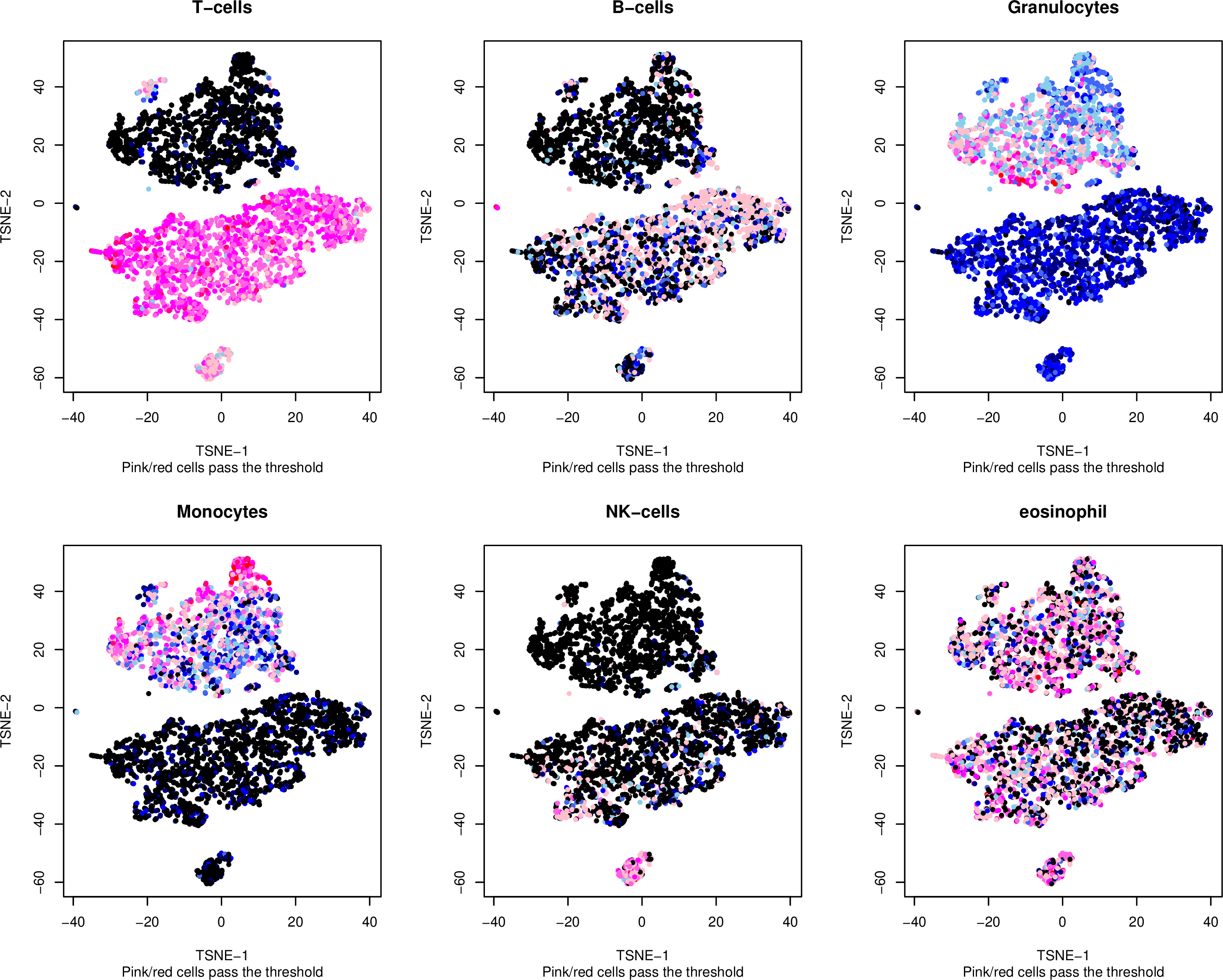

Supplement: Supplementary file 2 — Additional file 2: Supplementary file 2. To demonstrate the utility of scQCEA, we apply the workflow to the sixteen gene expression profiles of eight patients with metastatic melanoma, prepared from pre- and post-treatment experimental batches. You can find the QC interactive report at: https://github.com/isarnassiri/scQCEA/tree/Example-of-Application. Download and unzip the OGC_Interactive_QC_Report_P180121.zip file. You can open CLICK_ME.html file without using rStudio/R. [file 12864_2023_9447_MOESM2_ESM.zip › Inputs/10X-gex-grouped/FAI5649A20/P180121-keep_FAI5649A20_tSNE_Plot.png]

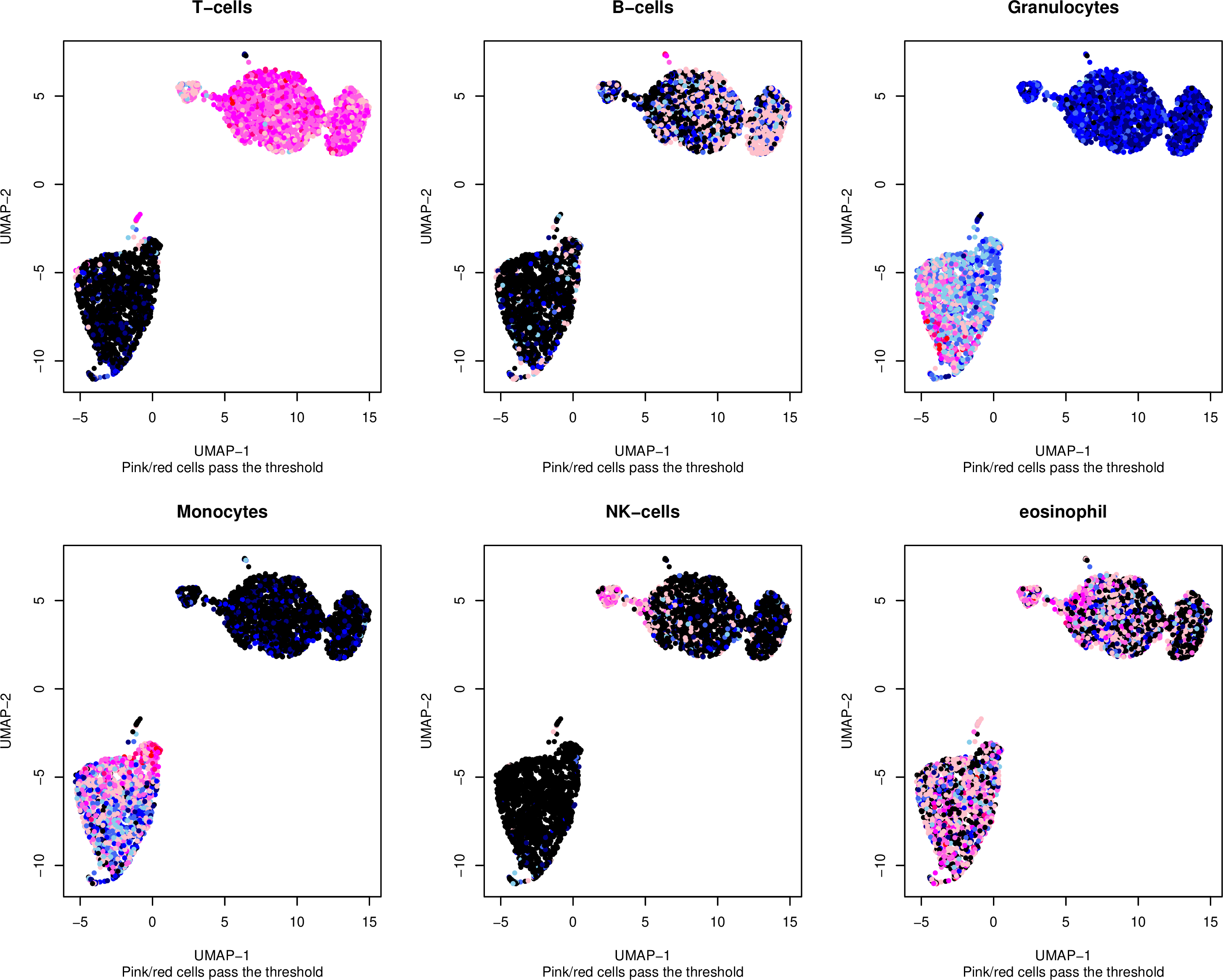

Supplement: Supplementary file 2 — Additional file 2: Supplementary file 2. To demonstrate the utility of scQCEA, we apply the workflow to the sixteen gene expression profiles of eight patients with metastatic melanoma, prepared from pre- and post-treatment experimental batches. You can find the QC interactive report at: https://github.com/isarnassiri/scQCEA/tree/Example-of-Application. Download and unzip the OGC_Interactive_QC_Report_P180121.zip file. You can open CLICK_ME.html file without using rStudio/R. [file 12864_2023_9447_MOESM2_ESM.zip › Inputs/10X-gex-grouped/FAI5649A20/P180121-keep_FAI5649A20_UMAP_Plot.png]

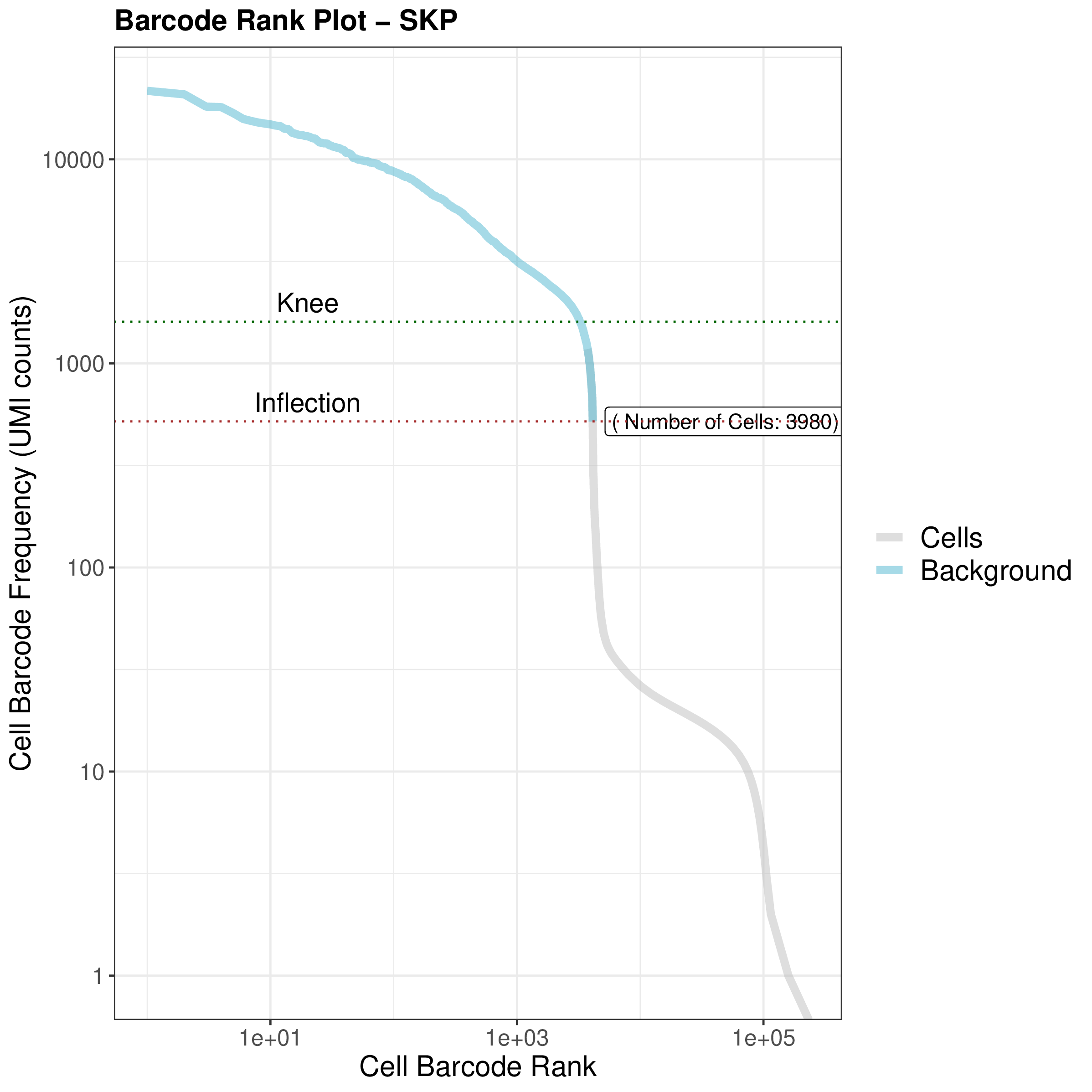

Supplement: Supplementary file 2 — Additional file 2: Supplementary file 2. To demonstrate the utility of scQCEA, we apply the workflow to the sixteen gene expression profiles of eight patients with metastatic melanoma, prepared from pre- and post-treatment experimental batches. You can find the QC interactive report at: https://github.com/isarnassiri/scQCEA/tree/Example-of-Application. Download and unzip the OGC_Interactive_QC_Report_P180121.zip file. You can open CLICK_ME.html file without using rStudio/R. [file 12864_2023_9447_MOESM2_ESM.zip › Inputs/10X-gex-grouped/FAI5649A21/P180121-keep_FAI5649A21_BarcodeRankPlot_10X.png]

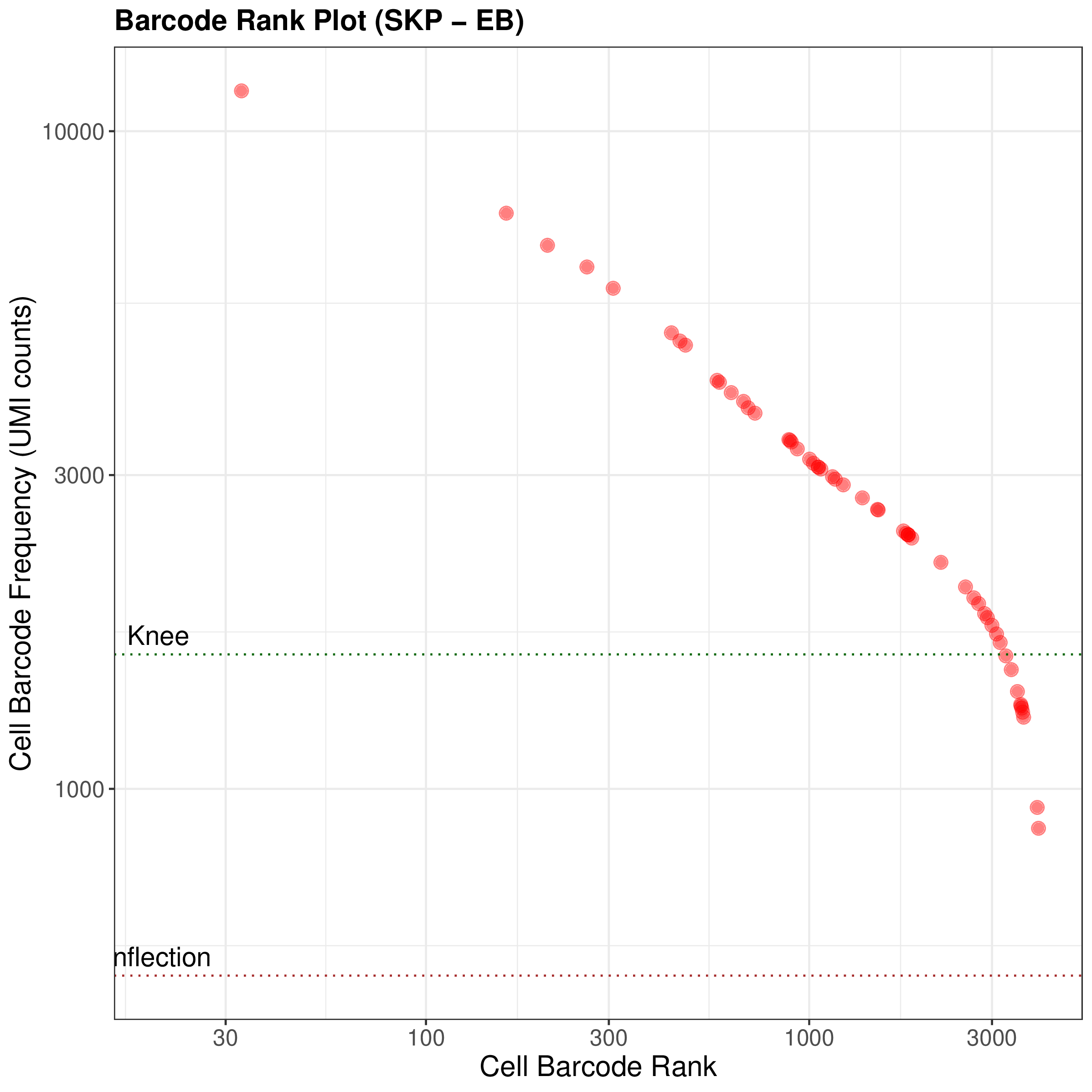

Supplement: Supplementary file 2 — Additional file 2: Supplementary file 2. To demonstrate the utility of scQCEA, we apply the workflow to the sixteen gene expression profiles of eight patients with metastatic melanoma, prepared from pre- and post-treatment experimental batches. You can find the QC interactive report at: https://github.com/isarnassiri/scQCEA/tree/Example-of-Application. Download and unzip the OGC_Interactive_QC_Report_P180121.zip file. You can open CLICK_ME.html file without using rStudio/R. [file 12864_2023_9447_MOESM2_ESM.zip › Inputs/10X-gex-grouped/FAI5649A21/P180121-keep_FAI5649A21_BarcodeRankPlot_EB_FilterOut.png]

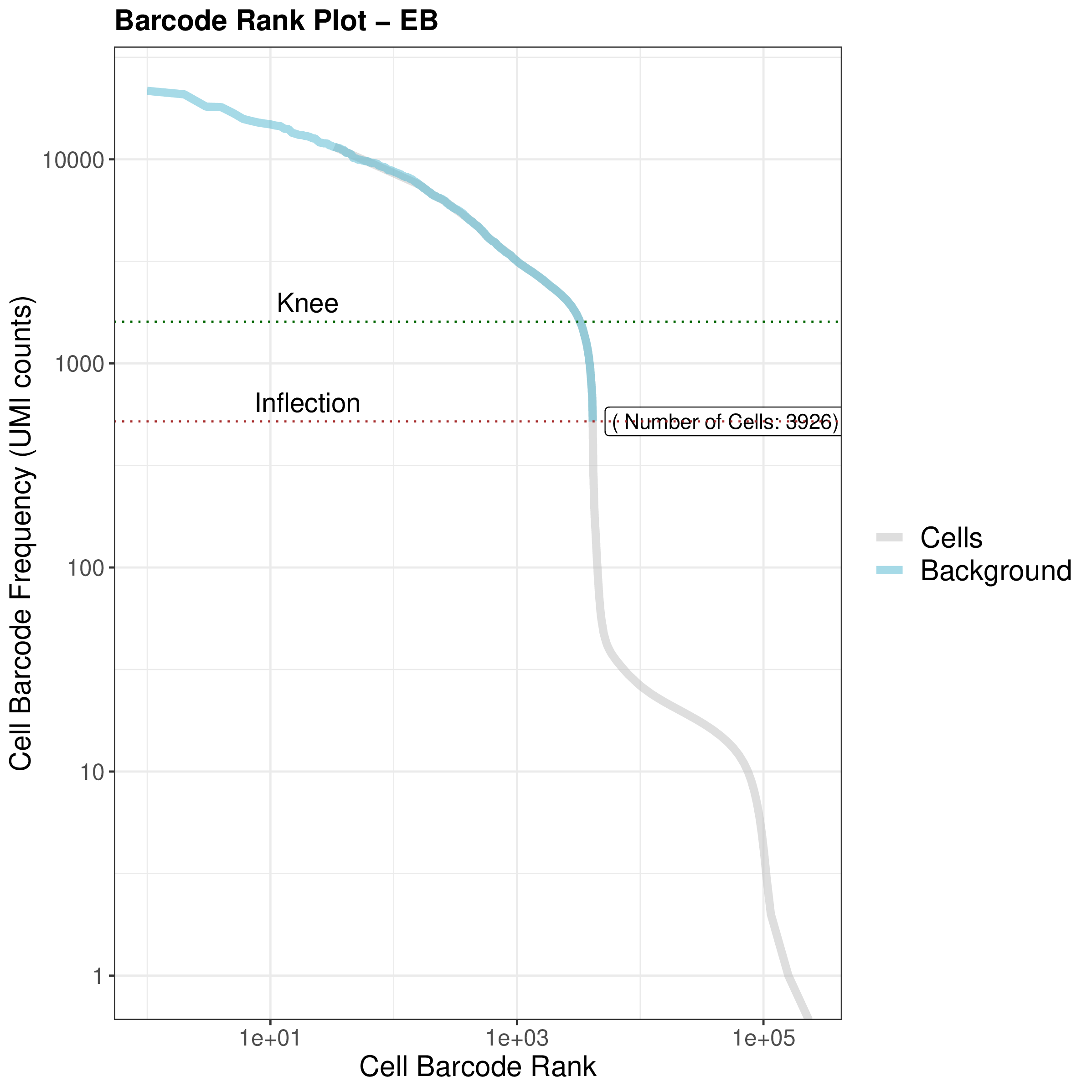

Supplement: Supplementary file 2 — Additional file 2: Supplementary file 2. To demonstrate the utility of scQCEA, we apply the workflow to the sixteen gene expression profiles of eight patients with metastatic melanoma, prepared from pre- and post-treatment experimental batches. You can find the QC interactive report at: https://github.com/isarnassiri/scQCEA/tree/Example-of-Application. Download and unzip the OGC_Interactive_QC_Report_P180121.zip file. You can open CLICK_ME.html file without using rStudio/R. [file 12864_2023_9447_MOESM2_ESM.zip › Inputs/10X-gex-grouped/FAI5649A21/P180121-keep_FAI5649A21_BarcodeRankPlot_EB.png]

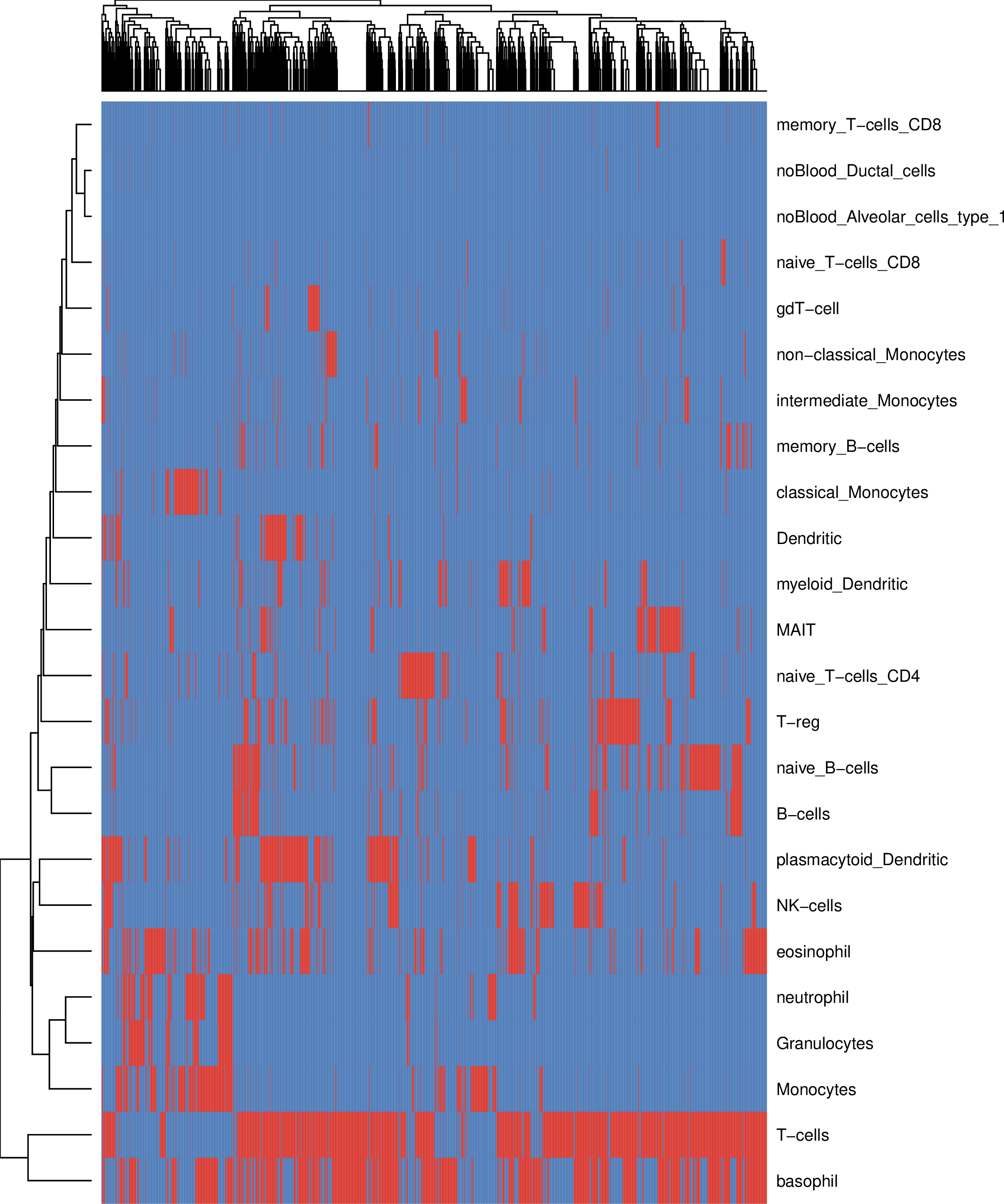

Supplement: Supplementary file 2 — Additional file 2: Supplementary file 2. To demonstrate the utility of scQCEA, we apply the workflow to the sixteen gene expression profiles of eight patients with metastatic melanoma, prepared from pre- and post-treatment experimental batches. You can find the QC interactive report at: https://github.com/isarnassiri/scQCEA/tree/Example-of-Application. Download and unzip the OGC_Interactive_QC_Report_P180121.zip file. You can open CLICK_ME.html file without using rStudio/R. [file 12864_2023_9447_MOESM2_ESM.zip › Inputs/10X-gex-grouped/FAI5649A21/P180121-keep_FAI5649A21_Celltype_assignment_HeatMap.png]

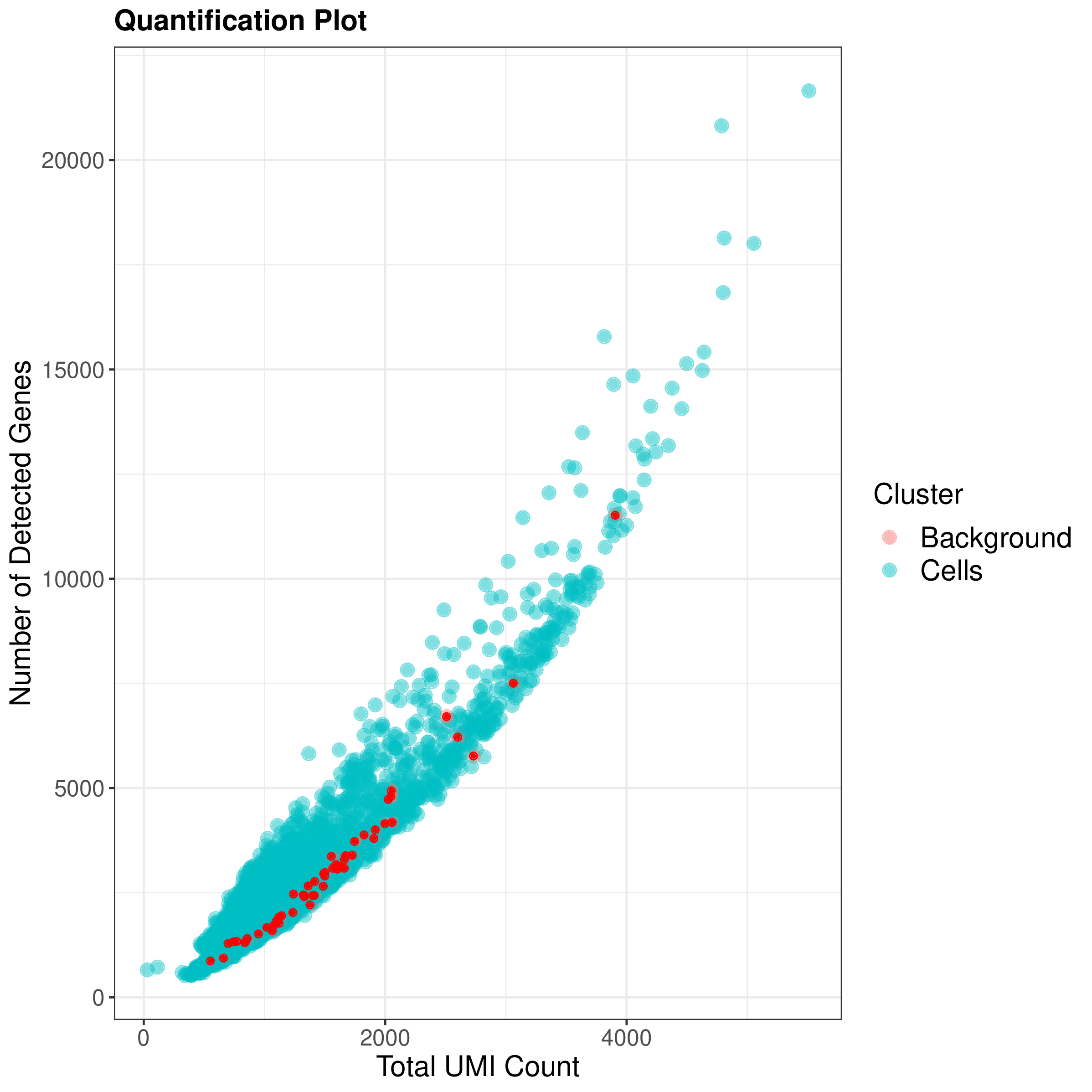

Supplement: Supplementary file 2 — Additional file 2: Supplementary file 2. To demonstrate the utility of scQCEA, we apply the workflow to the sixteen gene expression profiles of eight patients with metastatic melanoma, prepared from pre- and post-treatment experimental batches. You can find the QC interactive report at: https://github.com/isarnassiri/scQCEA/tree/Example-of-Application. Download and unzip the OGC_Interactive_QC_Report_P180121.zip file. You can open CLICK_ME.html file without using rStudio/R. [file 12864_2023_9447_MOESM2_ESM.zip › Inputs/10X-gex-grouped/FAI5649A21/P180121-keep_FAI5649A21_TotalUMIvsDetectedGenes.png]

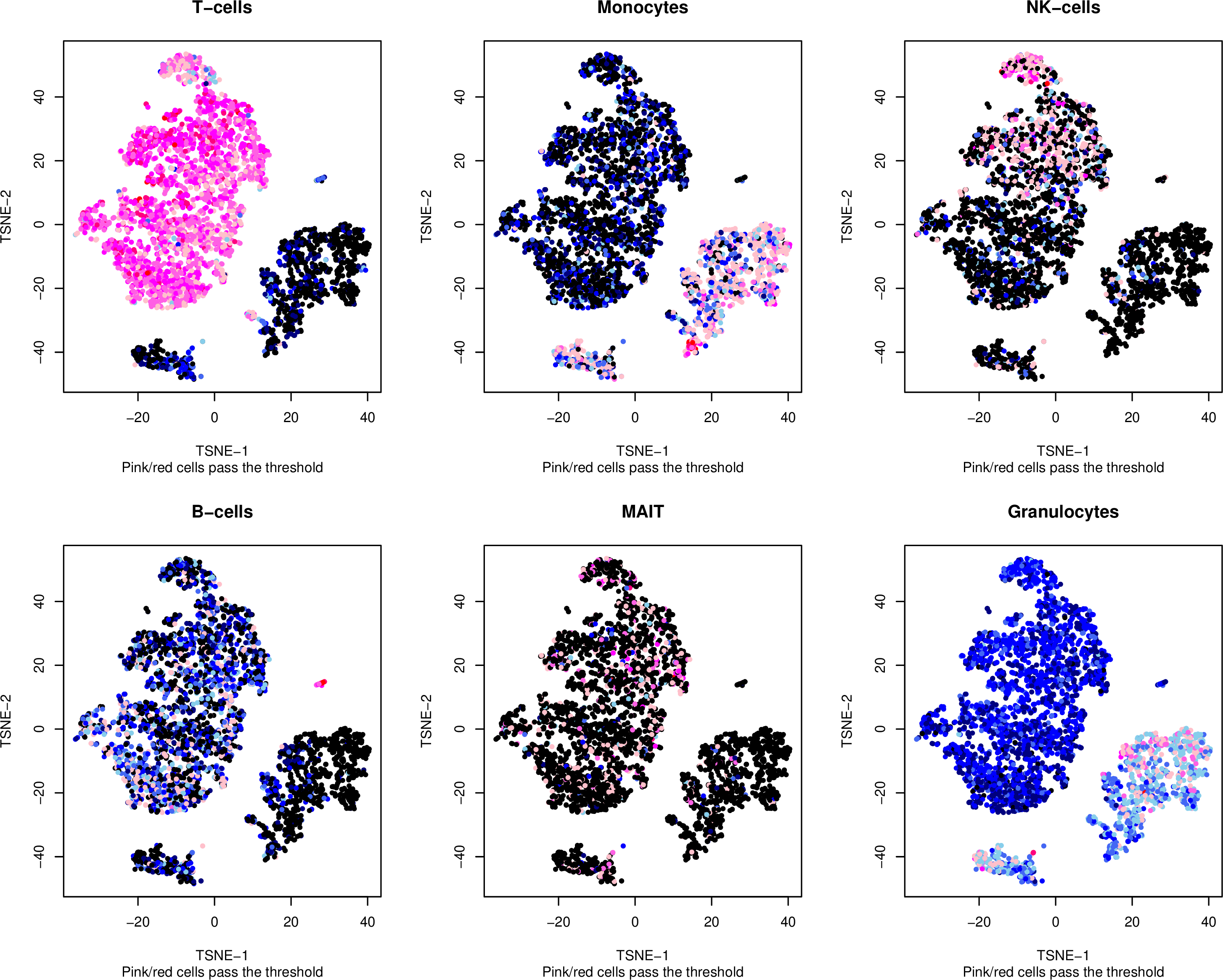

Supplement: Supplementary file 2 — Additional file 2: Supplementary file 2. To demonstrate the utility of scQCEA, we apply the workflow to the sixteen gene expression profiles of eight patients with metastatic melanoma, prepared from pre- and post-treatment experimental batches. You can find the QC interactive report at: https://github.com/isarnassiri/scQCEA/tree/Example-of-Application. Download and unzip the OGC_Interactive_QC_Report_P180121.zip file. You can open CLICK_ME.html file without using rStudio/R. [file 12864_2023_9447_MOESM2_ESM.zip › Inputs/10X-gex-grouped/FAI5649A21/P180121-keep_FAI5649A21_tSNE_Plot.png]

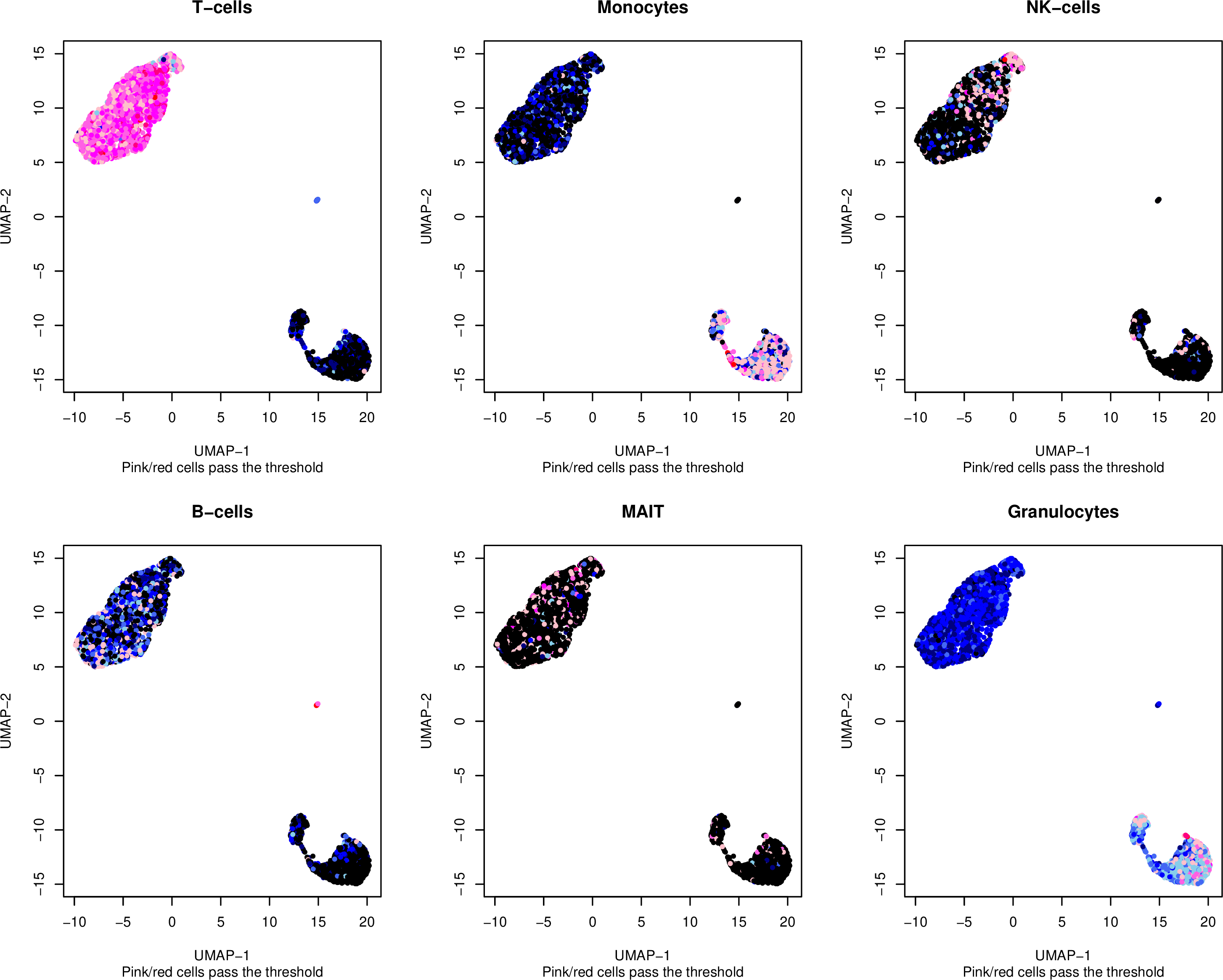

Supplement: Supplementary file 2 — Additional file 2: Supplementary file 2. To demonstrate the utility of scQCEA, we apply the workflow to the sixteen gene expression profiles of eight patients with metastatic melanoma, prepared from pre- and post-treatment experimental batches. You can find the QC interactive report at: https://github.com/isarnassiri/scQCEA/tree/Example-of-Application. Download and unzip the OGC_Interactive_QC_Report_P180121.zip file. You can open CLICK_ME.html file without using rStudio/R. [file 12864_2023_9447_MOESM2_ESM.zip › Inputs/10X-gex-grouped/FAI5649A21/P180121-keep_FAI5649A21_UMAP_Plot.png]

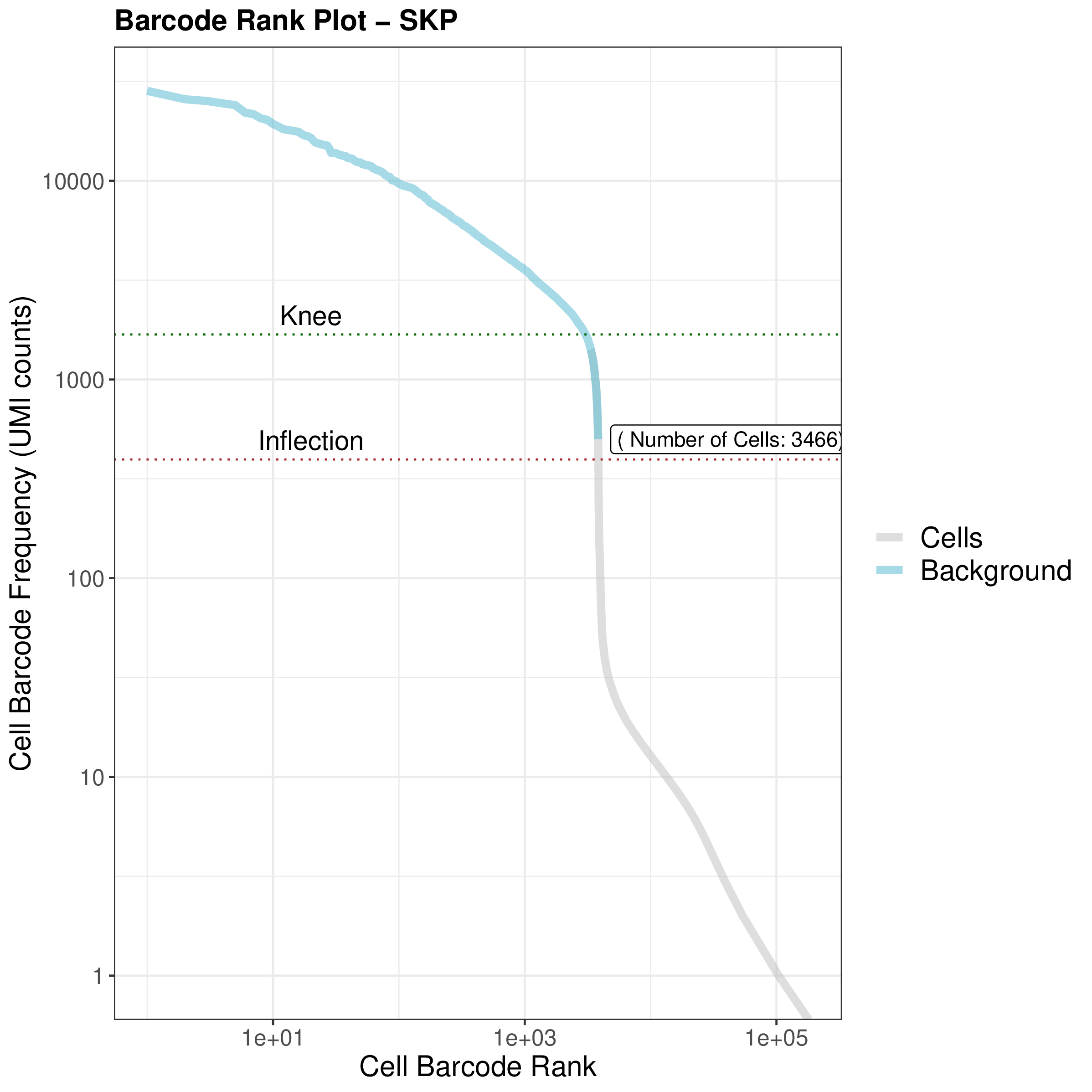

Supplement: Supplementary file 2 — Additional file 2: Supplementary file 2. To demonstrate the utility of scQCEA, we apply the workflow to the sixteen gene expression profiles of eight patients with metastatic melanoma, prepared from pre- and post-treatment experimental batches. You can find the QC interactive report at: https://github.com/isarnassiri/scQCEA/tree/Example-of-Application. Download and unzip the OGC_Interactive_QC_Report_P180121.zip file. You can open CLICK_ME.html file without using rStudio/R. [file 12864_2023_9447_MOESM2_ESM.zip › Inputs/10X-gex-grouped/FAI5649A22/P180121-keep_FAI5649A22_BarcodeRankPlot_10X.png]

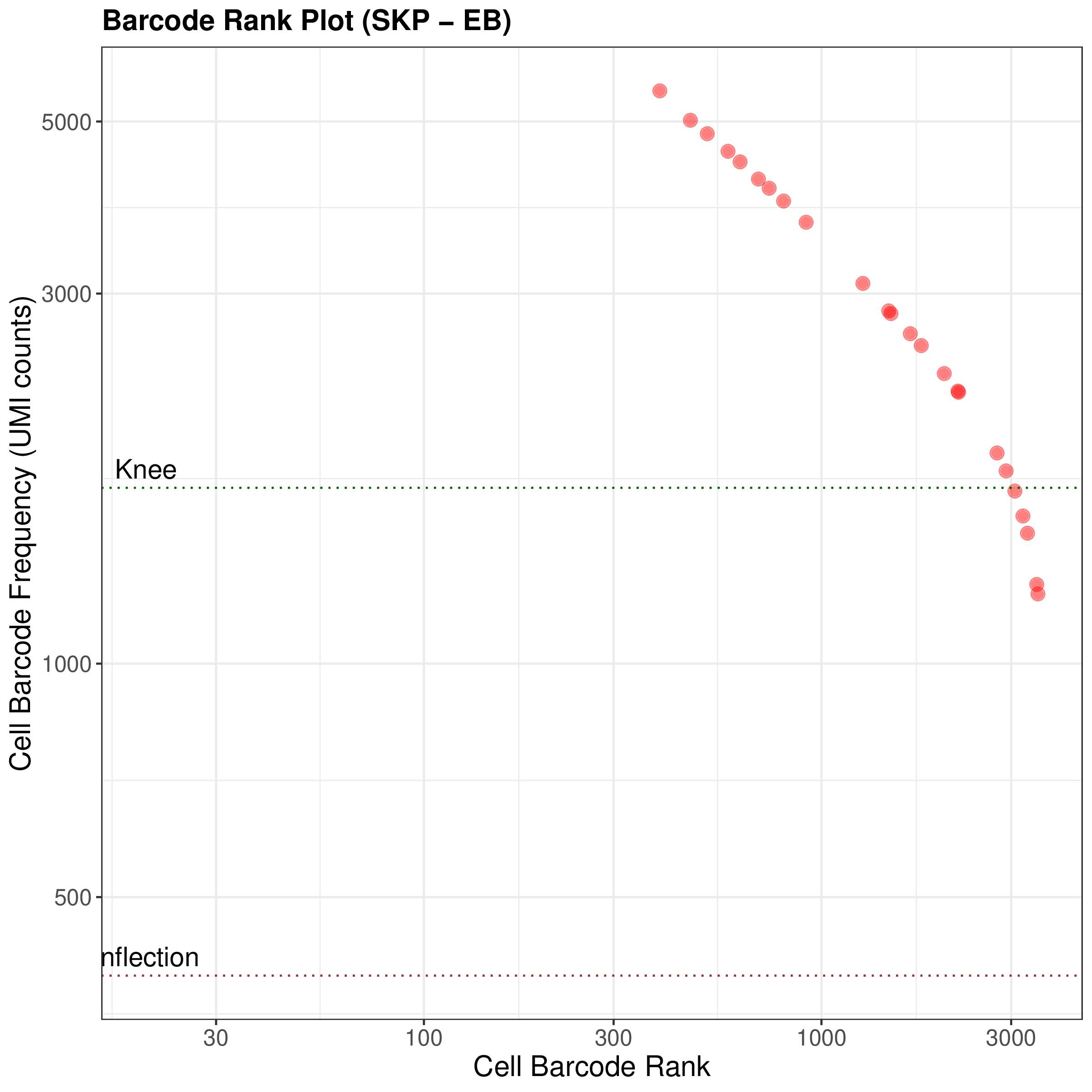

Supplement: Supplementary file 2 — Additional file 2: Supplementary file 2. To demonstrate the utility of scQCEA, we apply the workflow to the sixteen gene expression profiles of eight patients with metastatic melanoma, prepared from pre- and post-treatment experimental batches. You can find the QC interactive report at: https://github.com/isarnassiri/scQCEA/tree/Example-of-Application. Download and unzip the OGC_Interactive_QC_Report_P180121.zip file. You can open CLICK_ME.html file without using rStudio/R. [file 12864_2023_9447_MOESM2_ESM.zip › Inputs/10X-gex-grouped/FAI5649A22/P180121-keep_FAI5649A22_BarcodeRankPlot_EB_FilterOut.png]

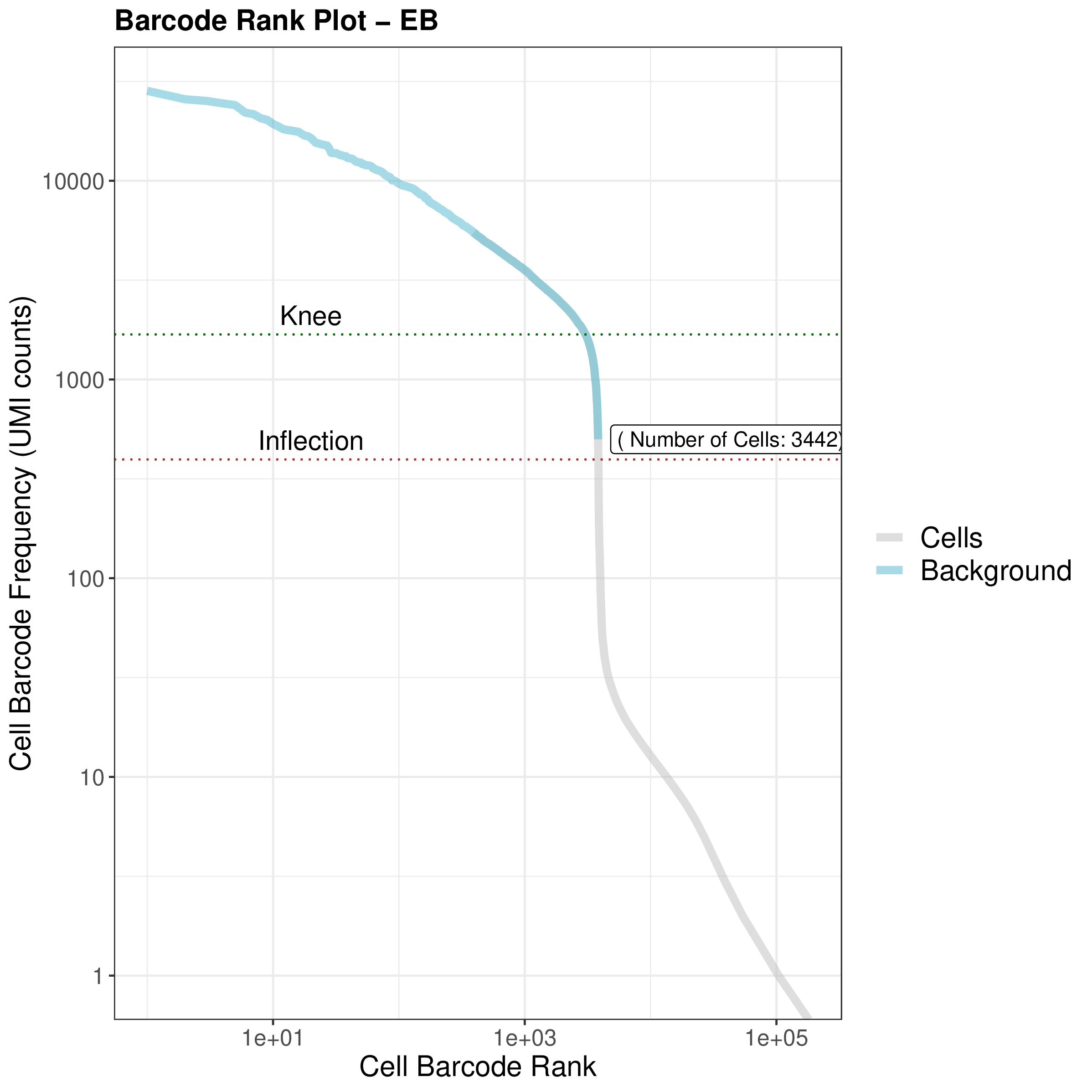

Supplement: Supplementary file 2 — Additional file 2: Supplementary file 2. To demonstrate the utility of scQCEA, we apply the workflow to the sixteen gene expression profiles of eight patients with metastatic melanoma, prepared from pre- and post-treatment experimental batches. You can find the QC interactive report at: https://github.com/isarnassiri/scQCEA/tree/Example-of-Application. Download and unzip the OGC_Interactive_QC_Report_P180121.zip file. You can open CLICK_ME.html file without using rStudio/R. [file 12864_2023_9447_MOESM2_ESM.zip › Inputs/10X-gex-grouped/FAI5649A22/P180121-keep_FAI5649A22_BarcodeRankPlot_EB.png]

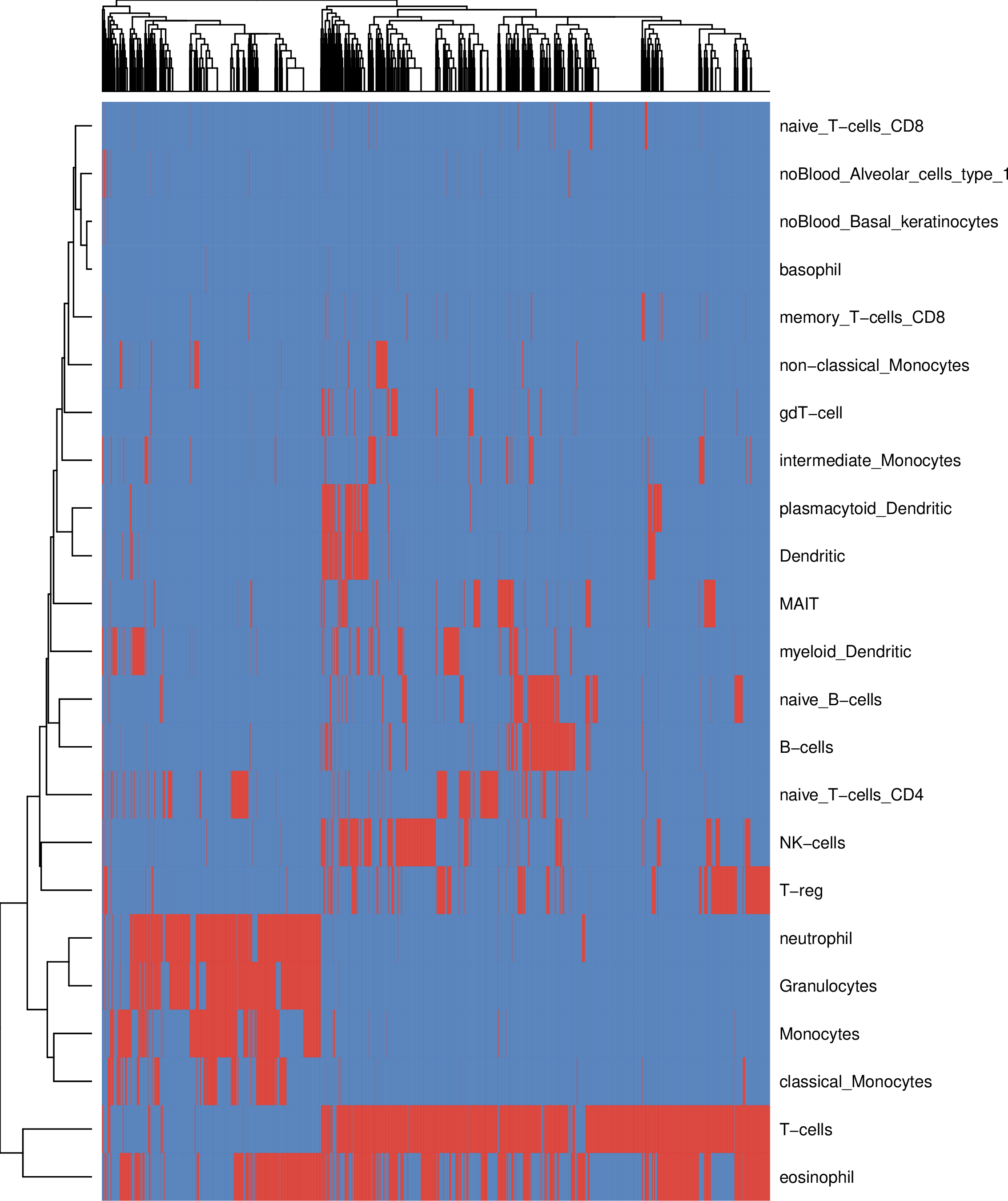

Supplement: Supplementary file 2 — Additional file 2: Supplementary file 2. To demonstrate the utility of scQCEA, we apply the workflow to the sixteen gene expression profiles of eight patients with metastatic melanoma, prepared from pre- and post-treatment experimental batches. You can find the QC interactive report at: https://github.com/isarnassiri/scQCEA/tree/Example-of-Application. Download and unzip the OGC_Interactive_QC_Report_P180121.zip file. You can open CLICK_ME.html file without using rStudio/R. [file 12864_2023_9447_MOESM2_ESM.zip › Inputs/10X-gex-grouped/FAI5649A22/P180121-keep_FAI5649A22_Celltype_assignment_HeatMap.png]

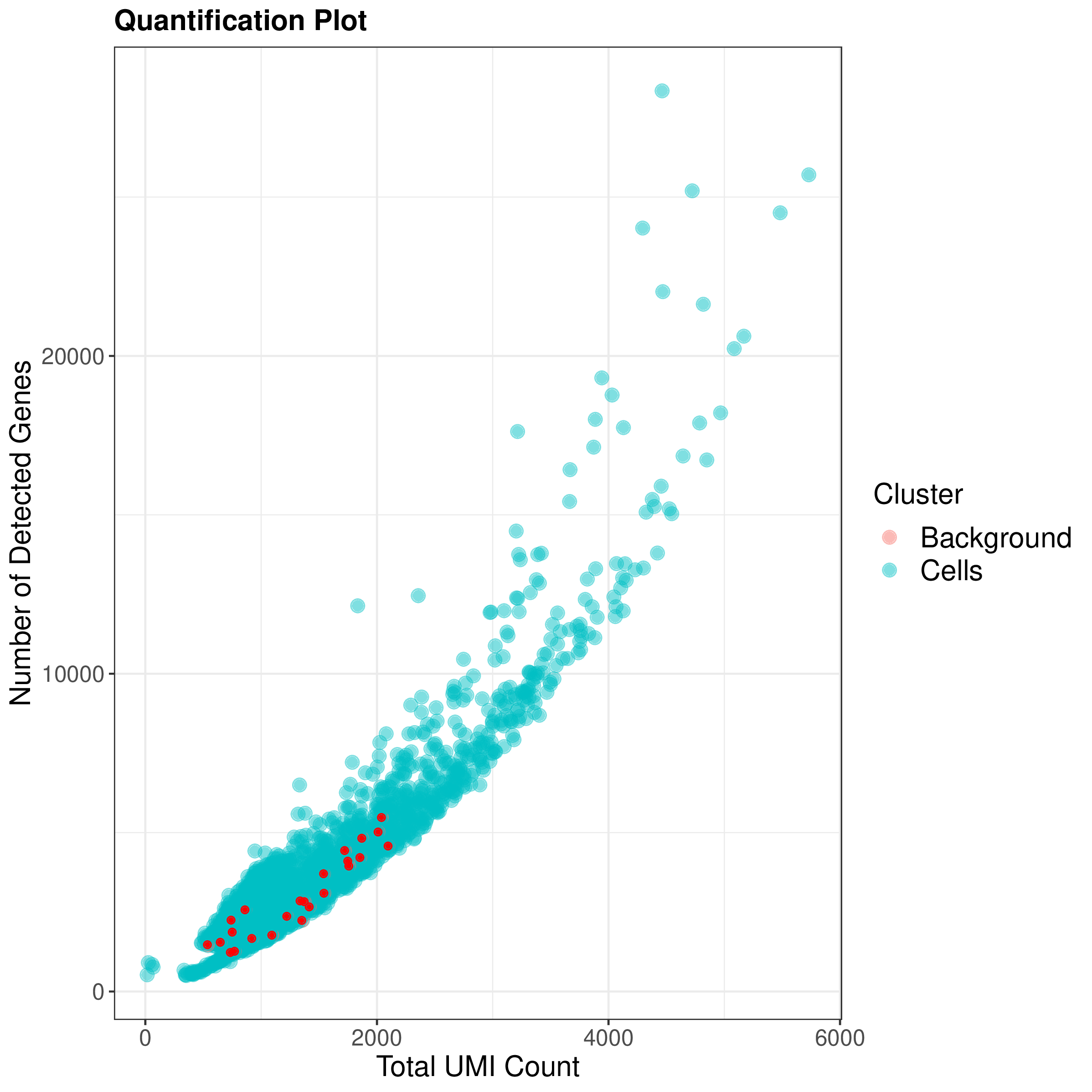

Supplement: Supplementary file 2 — Additional file 2: Supplementary file 2. To demonstrate the utility of scQCEA, we apply the workflow to the sixteen gene expression profiles of eight patients with metastatic melanoma, prepared from pre- and post-treatment experimental batches. You can find the QC interactive report at: https://github.com/isarnassiri/scQCEA/tree/Example-of-Application. Download and unzip the OGC_Interactive_QC_Report_P180121.zip file. You can open CLICK_ME.html file without using rStudio/R. [file 12864_2023_9447_MOESM2_ESM.zip › Inputs/10X-gex-grouped/FAI5649A22/P180121-keep_FAI5649A22_TotalUMIvsDetectedGenes.png]

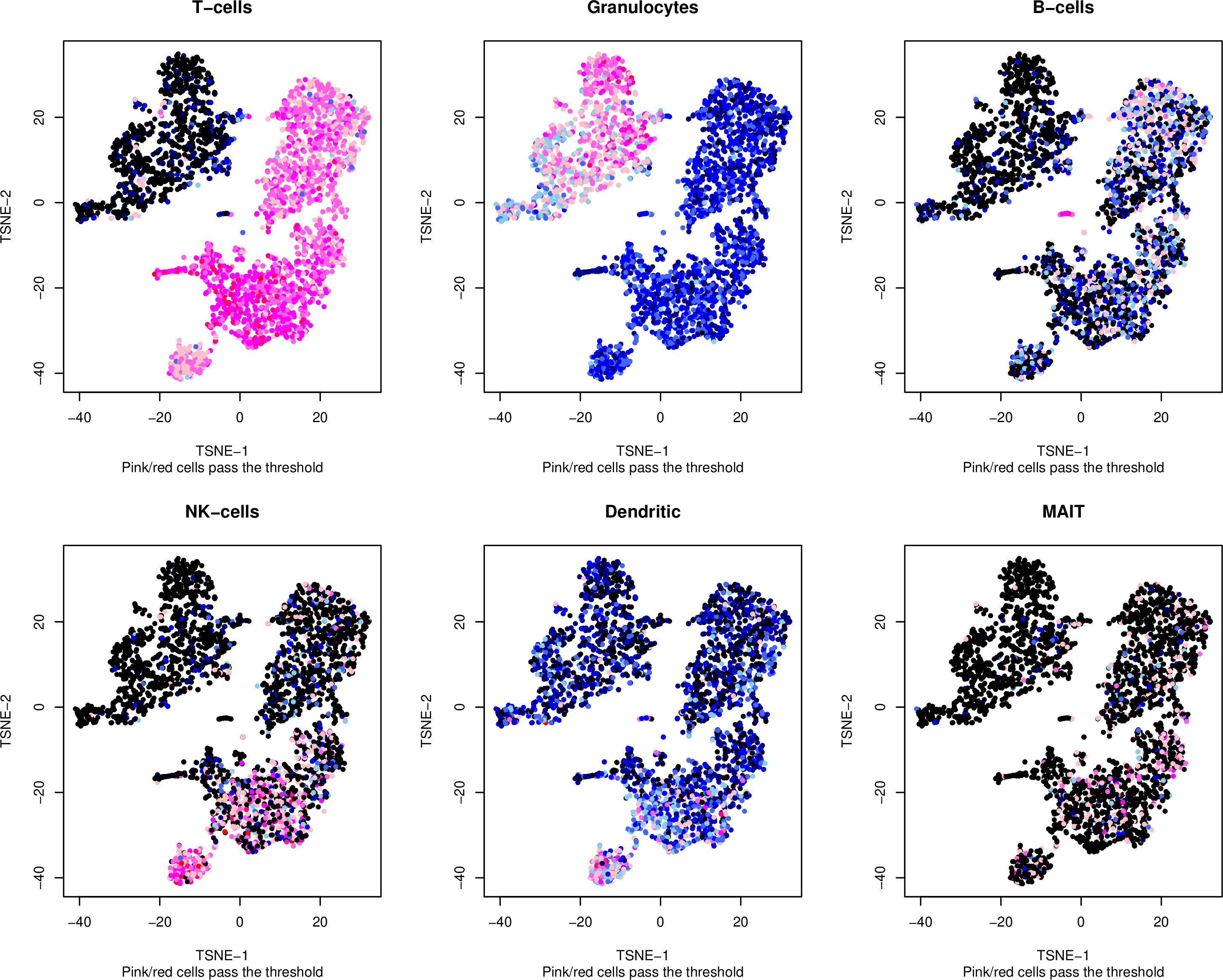

Supplement: Supplementary file 2 — Additional file 2: Supplementary file 2. To demonstrate the utility of scQCEA, we apply the workflow to the sixteen gene expression profiles of eight patients with metastatic melanoma, prepared from pre- and post-treatment experimental batches. You can find the QC interactive report at: https://github.com/isarnassiri/scQCEA/tree/Example-of-Application. Download and unzip the OGC_Interactive_QC_Report_P180121.zip file. You can open CLICK_ME.html file without using rStudio/R. [file 12864_2023_9447_MOESM2_ESM.zip › Inputs/10X-gex-grouped/FAI5649A22/P180121-keep_FAI5649A22_tSNE_Plot.png]

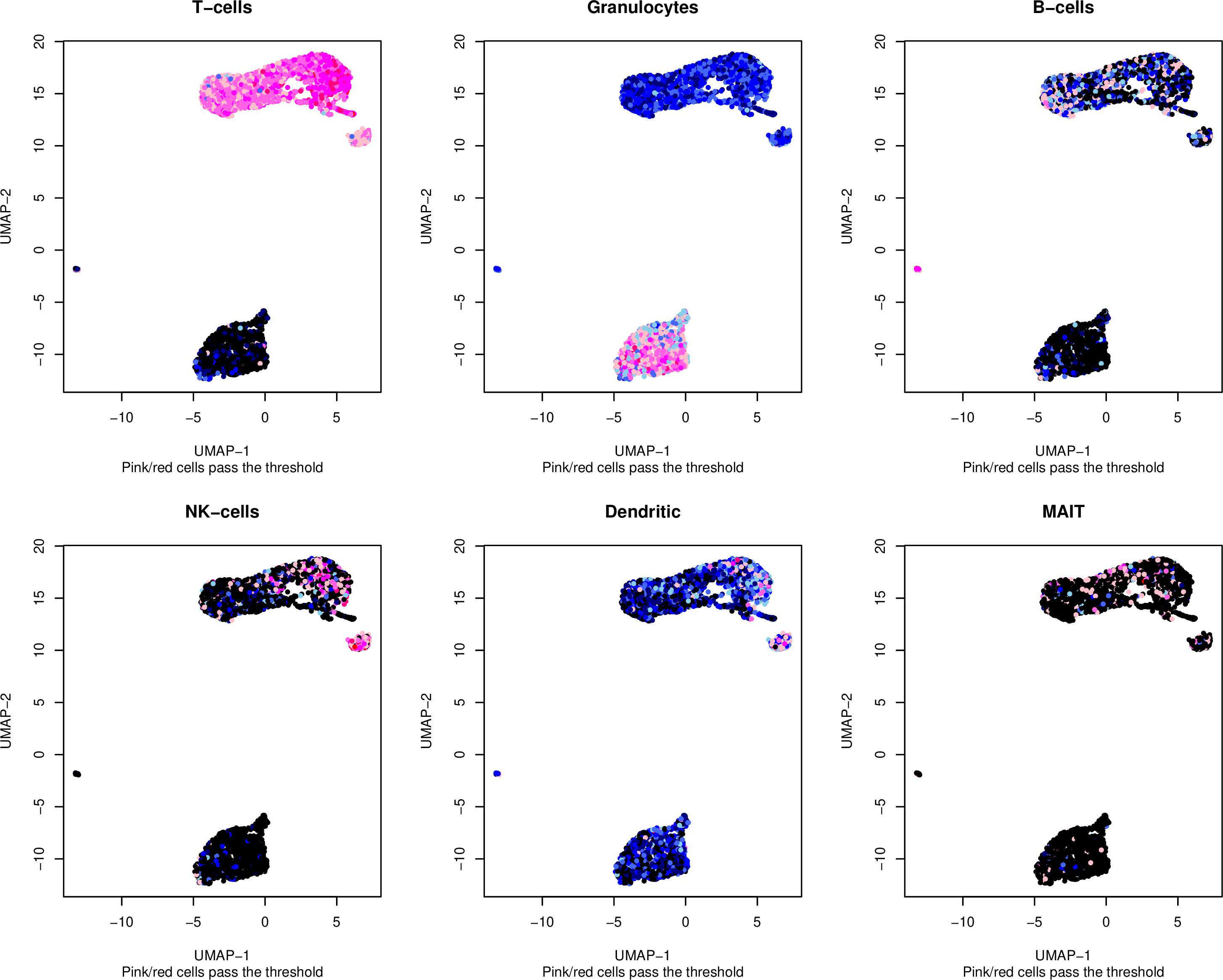

Supplement: Supplementary file 2 — Additional file 2: Supplementary file 2. To demonstrate the utility of scQCEA, we apply the workflow to the sixteen gene expression profiles of eight patients with metastatic melanoma, prepared from pre- and post-treatment experimental batches. You can find the QC interactive report at: https://github.com/isarnassiri/scQCEA/tree/Example-of-Application. Download and unzip the OGC_Interactive_QC_Report_P180121.zip file. You can open CLICK_ME.html file without using rStudio/R. [file 12864_2023_9447_MOESM2_ESM.zip › Inputs/10X-gex-grouped/FAI5649A22/P180121-keep_FAI5649A22_UMAP_Plot.png]

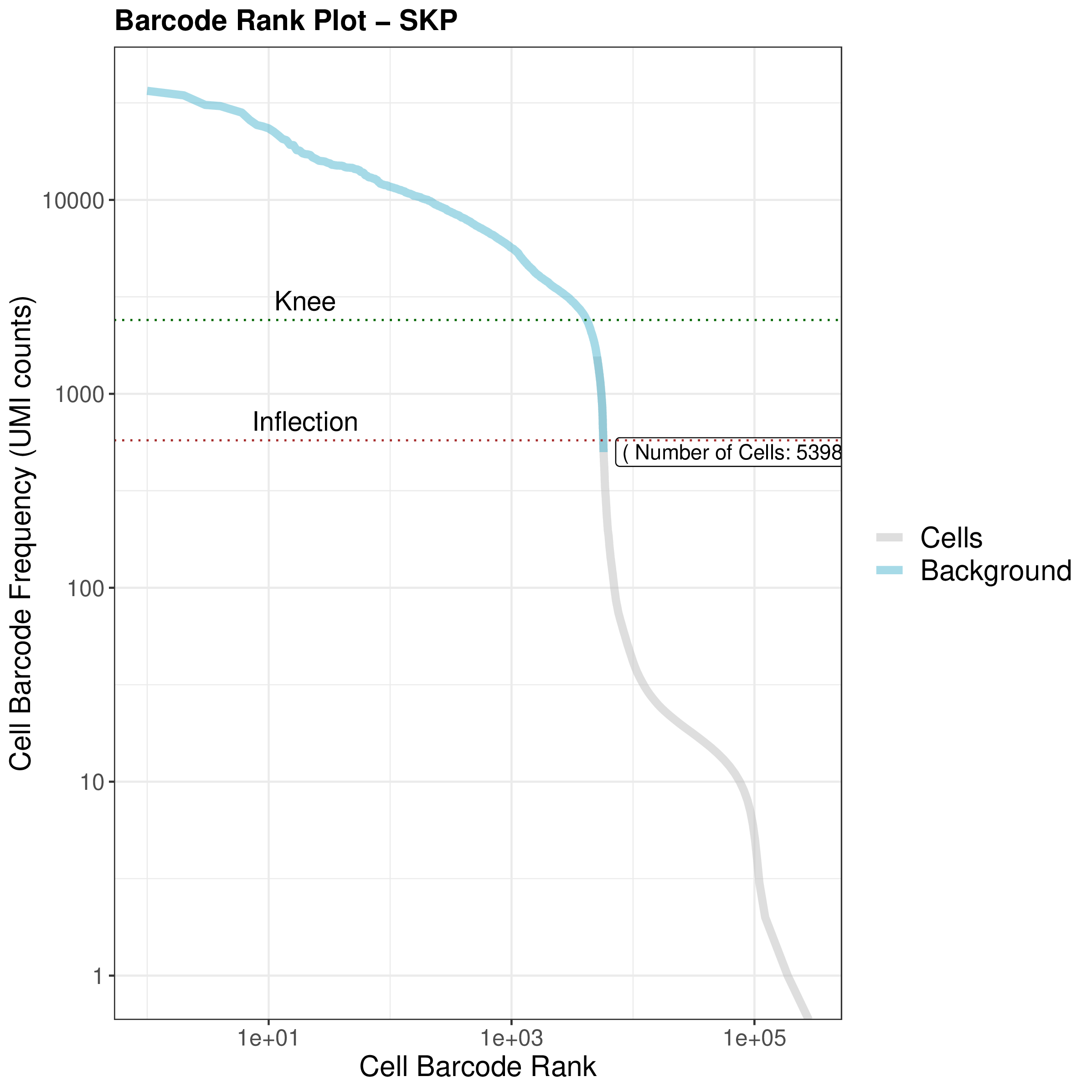

Supplement: Supplementary file 2 — Additional file 2: Supplementary file 2. To demonstrate the utility of scQCEA, we apply the workflow to the sixteen gene expression profiles of eight patients with metastatic melanoma, prepared from pre- and post-treatment experimental batches. You can find the QC interactive report at: https://github.com/isarnassiri/scQCEA/tree/Example-of-Application. Download and unzip the OGC_Interactive_QC_Report_P180121.zip file. You can open CLICK_ME.html file without using rStudio/R. [file 12864_2023_9447_MOESM2_ESM.zip › Inputs/10X-gex-grouped/FAI5649A23/P180121-keep_FAI5649A23_BarcodeRankPlot_10X.png]

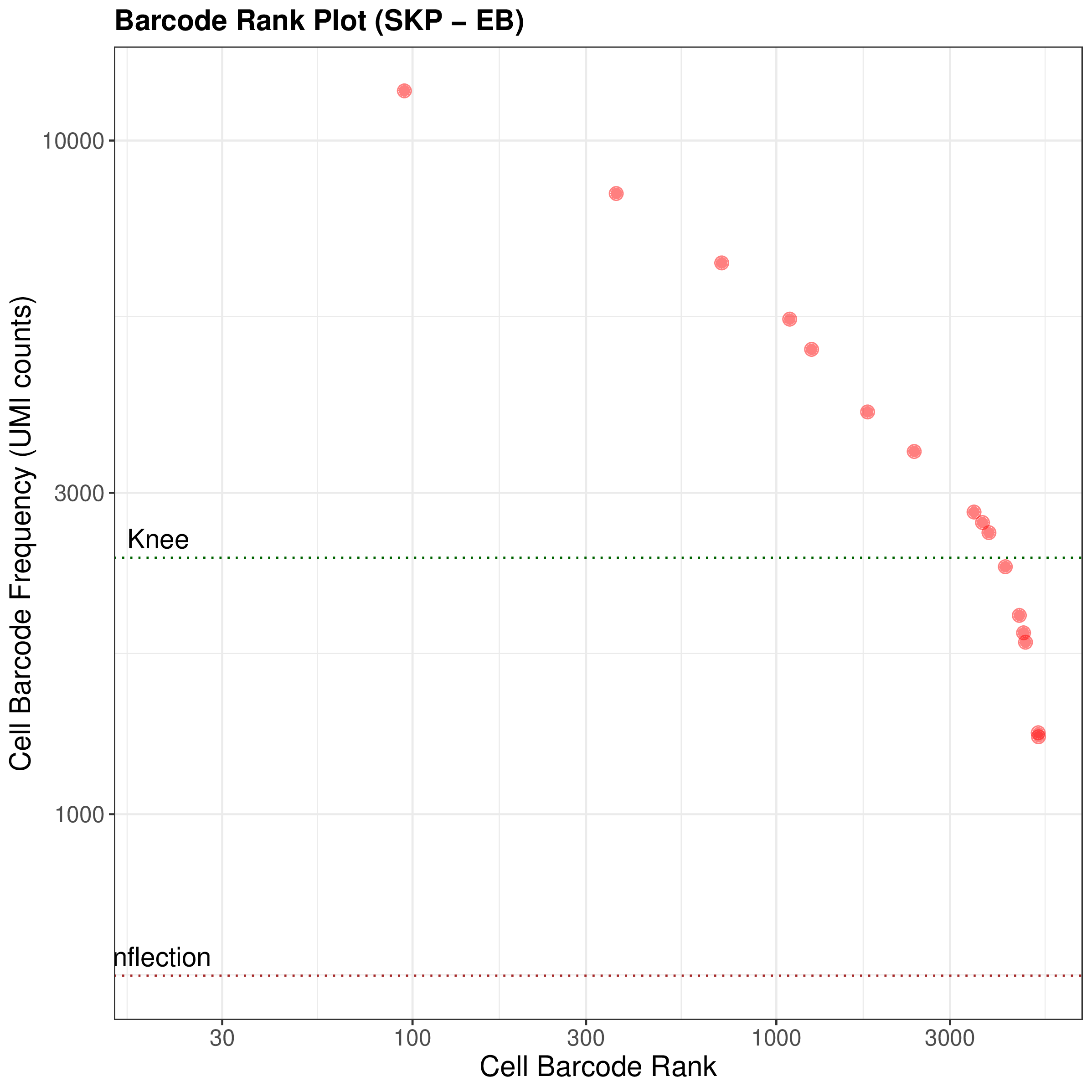

Supplement: Supplementary file 2 — Additional file 2: Supplementary file 2. To demonstrate the utility of scQCEA, we apply the workflow to the sixteen gene expression profiles of eight patients with metastatic melanoma, prepared from pre- and post-treatment experimental batches. You can find the QC interactive report at: https://github.com/isarnassiri/scQCEA/tree/Example-of-Application. Download and unzip the OGC_Interactive_QC_Report_P180121.zip file. You can open CLICK_ME.html file without using rStudio/R. [file 12864_2023_9447_MOESM2_ESM.zip › Inputs/10X-gex-grouped/FAI5649A23/P180121-keep_FAI5649A23_BarcodeRankPlot_EB_FilterOut.png]

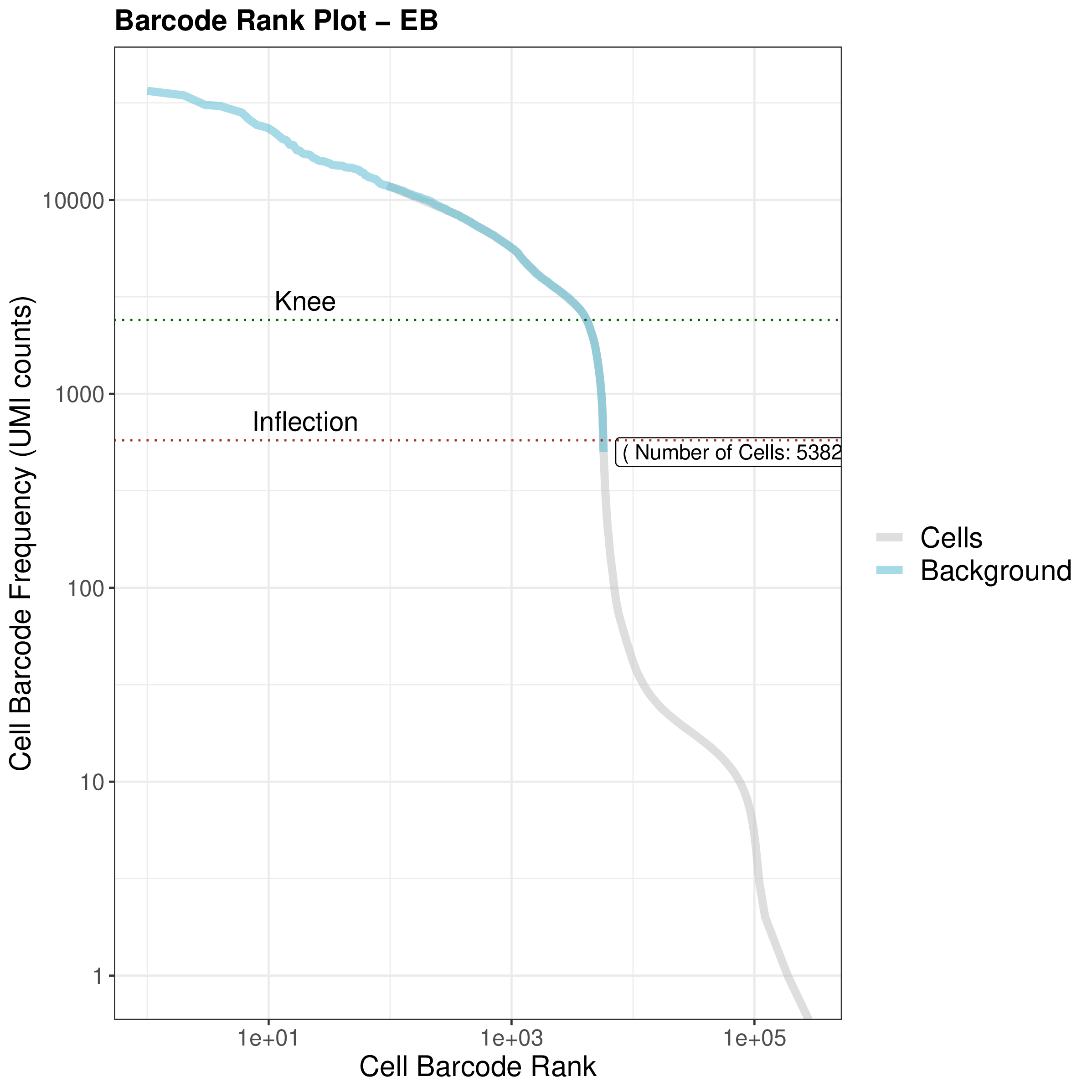

Supplement: Supplementary file 2 — Additional file 2: Supplementary file 2. To demonstrate the utility of scQCEA, we apply the workflow to the sixteen gene expression profiles of eight patients with metastatic melanoma, prepared from pre- and post-treatment experimental batches. You can find the QC interactive report at: https://github.com/isarnassiri/scQCEA/tree/Example-of-Application. Download and unzip the OGC_Interactive_QC_Report_P180121.zip file. You can open CLICK_ME.html file without using rStudio/R. [file 12864_2023_9447_MOESM2_ESM.zip › Inputs/10X-gex-grouped/FAI5649A23/P180121-keep_FAI5649A23_BarcodeRankPlot_EB.png]

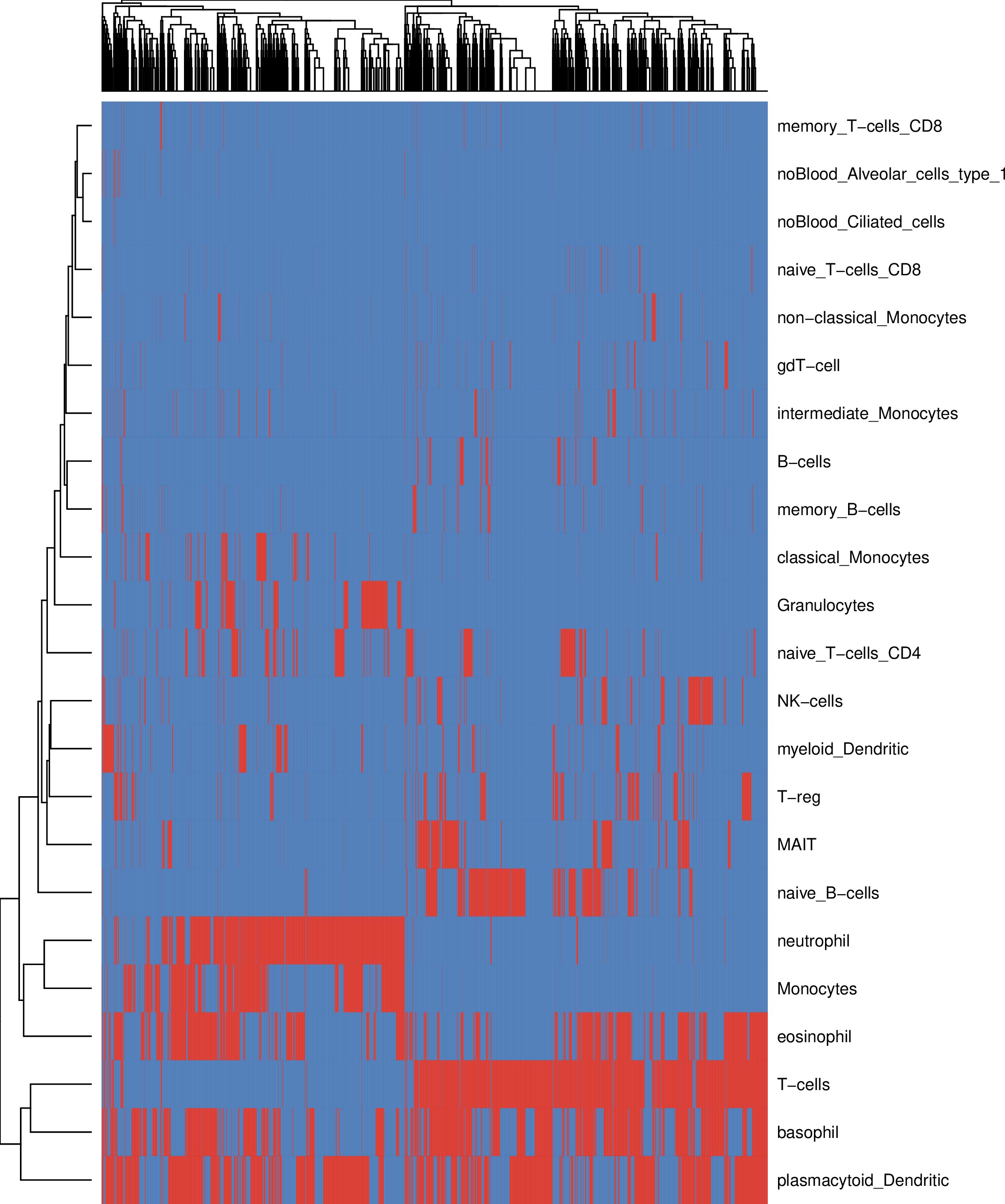

Supplement: Supplementary file 2 — Additional file 2: Supplementary file 2. To demonstrate the utility of scQCEA, we apply the workflow to the sixteen gene expression profiles of eight patients with metastatic melanoma, prepared from pre- and post-treatment experimental batches. You can find the QC interactive report at: https://github.com/isarnassiri/scQCEA/tree/Example-of-Application. Download and unzip the OGC_Interactive_QC_Report_P180121.zip file. You can open CLICK_ME.html file without using rStudio/R. [file 12864_2023_9447_MOESM2_ESM.zip › Inputs/10X-gex-grouped/FAI5649A23/P180121-keep_FAI5649A23_Celltype_assignment_HeatMap.png]

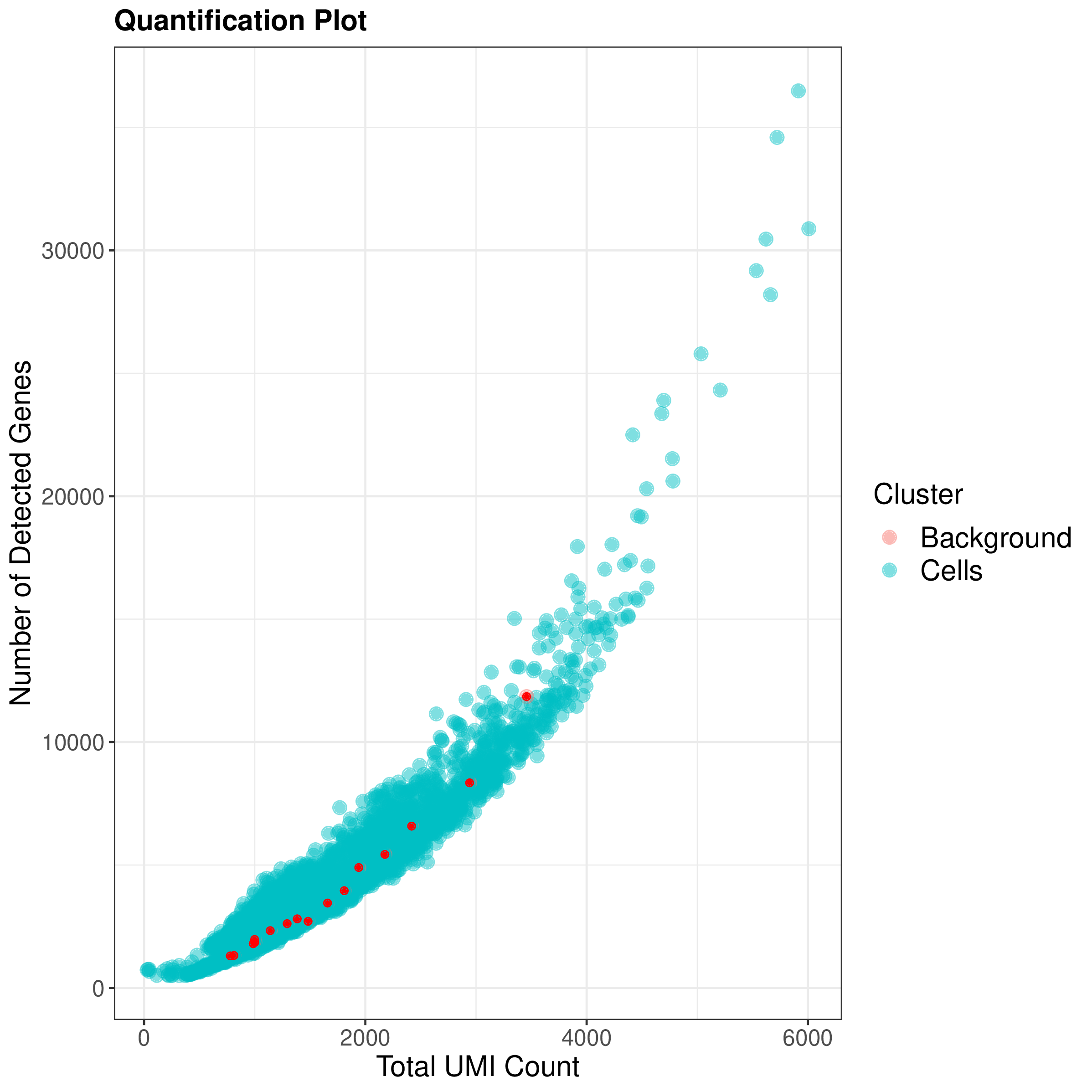

Supplement: Supplementary file 2 — Additional file 2: Supplementary file 2. To demonstrate the utility of scQCEA, we apply the workflow to the sixteen gene expression profiles of eight patients with metastatic melanoma, prepared from pre- and post-treatment experimental batches. You can find the QC interactive report at: https://github.com/isarnassiri/scQCEA/tree/Example-of-Application. Download and unzip the OGC_Interactive_QC_Report_P180121.zip file. You can open CLICK_ME.html file without using rStudio/R. [file 12864_2023_9447_MOESM2_ESM.zip › Inputs/10X-gex-grouped/FAI5649A23/P180121-keep_FAI5649A23_TotalUMIvsDetectedGenes.png]

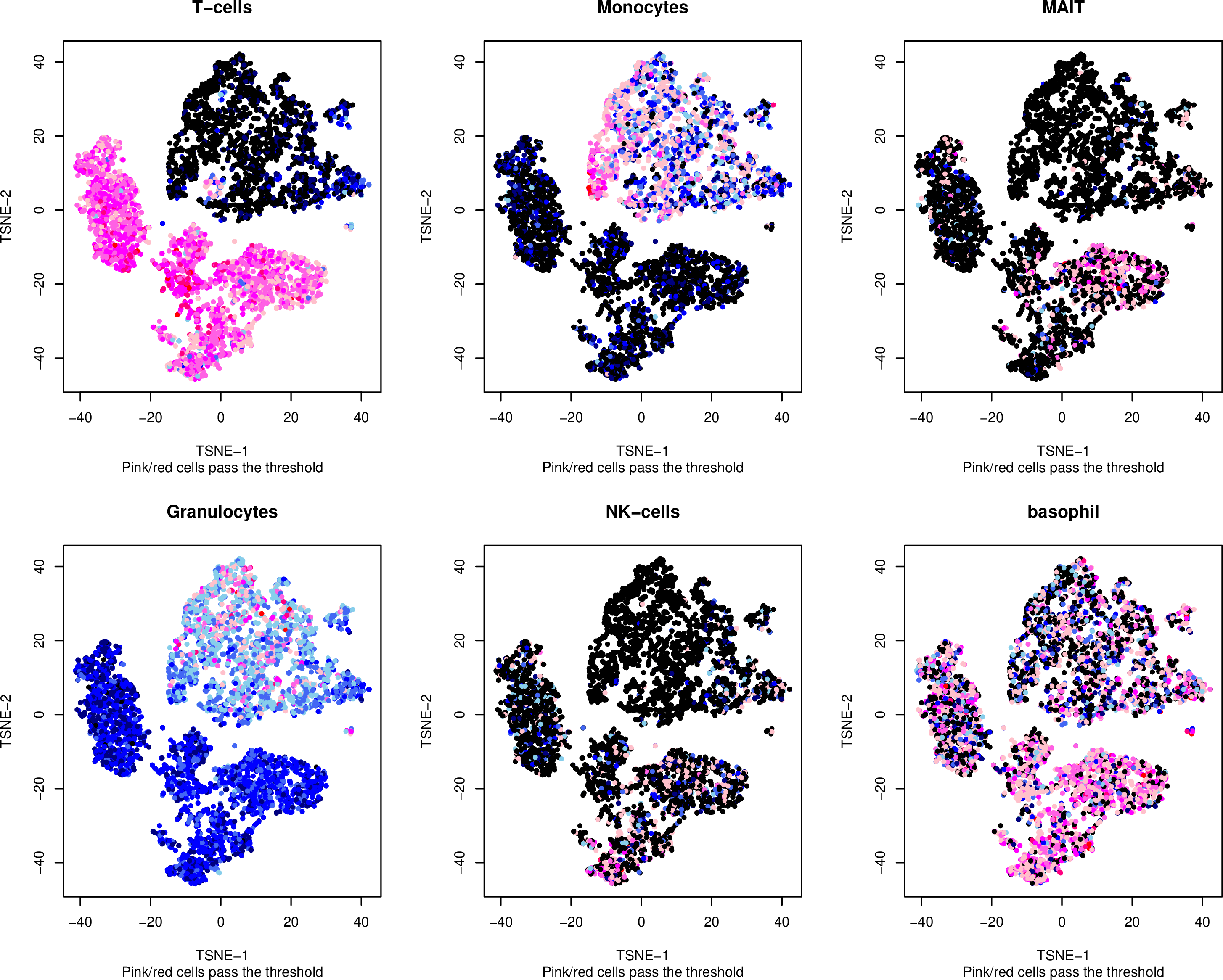

Supplement: Supplementary file 2 — Additional file 2: Supplementary file 2. To demonstrate the utility of scQCEA, we apply the workflow to the sixteen gene expression profiles of eight patients with metastatic melanoma, prepared from pre- and post-treatment experimental batches. You can find the QC interactive report at: https://github.com/isarnassiri/scQCEA/tree/Example-of-Application. Download and unzip the OGC_Interactive_QC_Report_P180121.zip file. You can open CLICK_ME.html file without using rStudio/R. [file 12864_2023_9447_MOESM2_ESM.zip › Inputs/10X-gex-grouped/FAI5649A23/P180121-keep_FAI5649A23_tSNE_Plot.png]

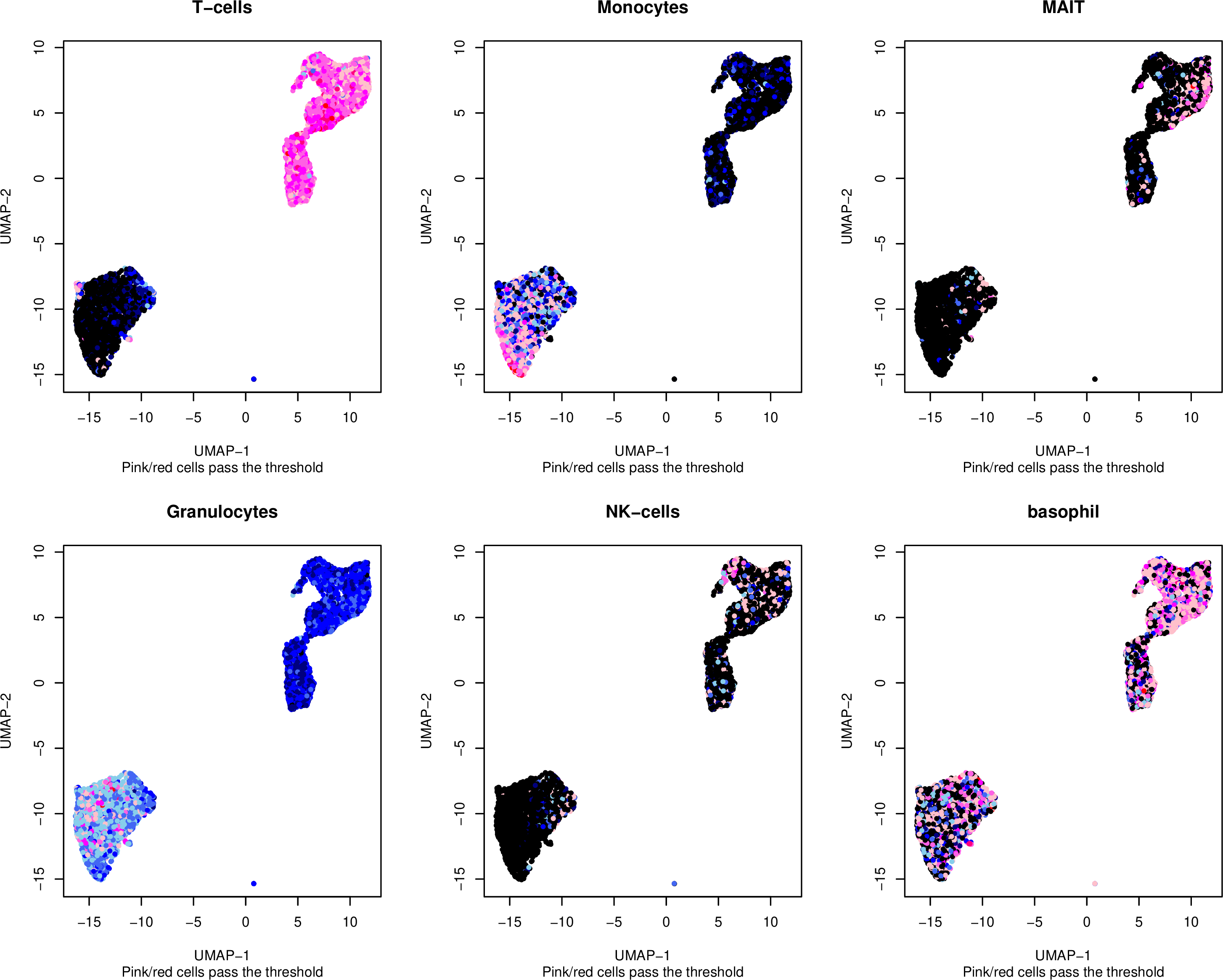

Supplement: Supplementary file 2 — Additional file 2: Supplementary file 2. To demonstrate the utility of scQCEA, we apply the workflow to the sixteen gene expression profiles of eight patients with metastatic melanoma, prepared from pre- and post-treatment experimental batches. You can find the QC interactive report at: https://github.com/isarnassiri/scQCEA/tree/Example-of-Application. Download and unzip the OGC_Interactive_QC_Report_P180121.zip file. You can open CLICK_ME.html file without using rStudio/R. [file 12864_2023_9447_MOESM2_ESM.zip › Inputs/10X-gex-grouped/FAI5649A23/P180121-keep_FAI5649A23_UMAP_Plot.png]

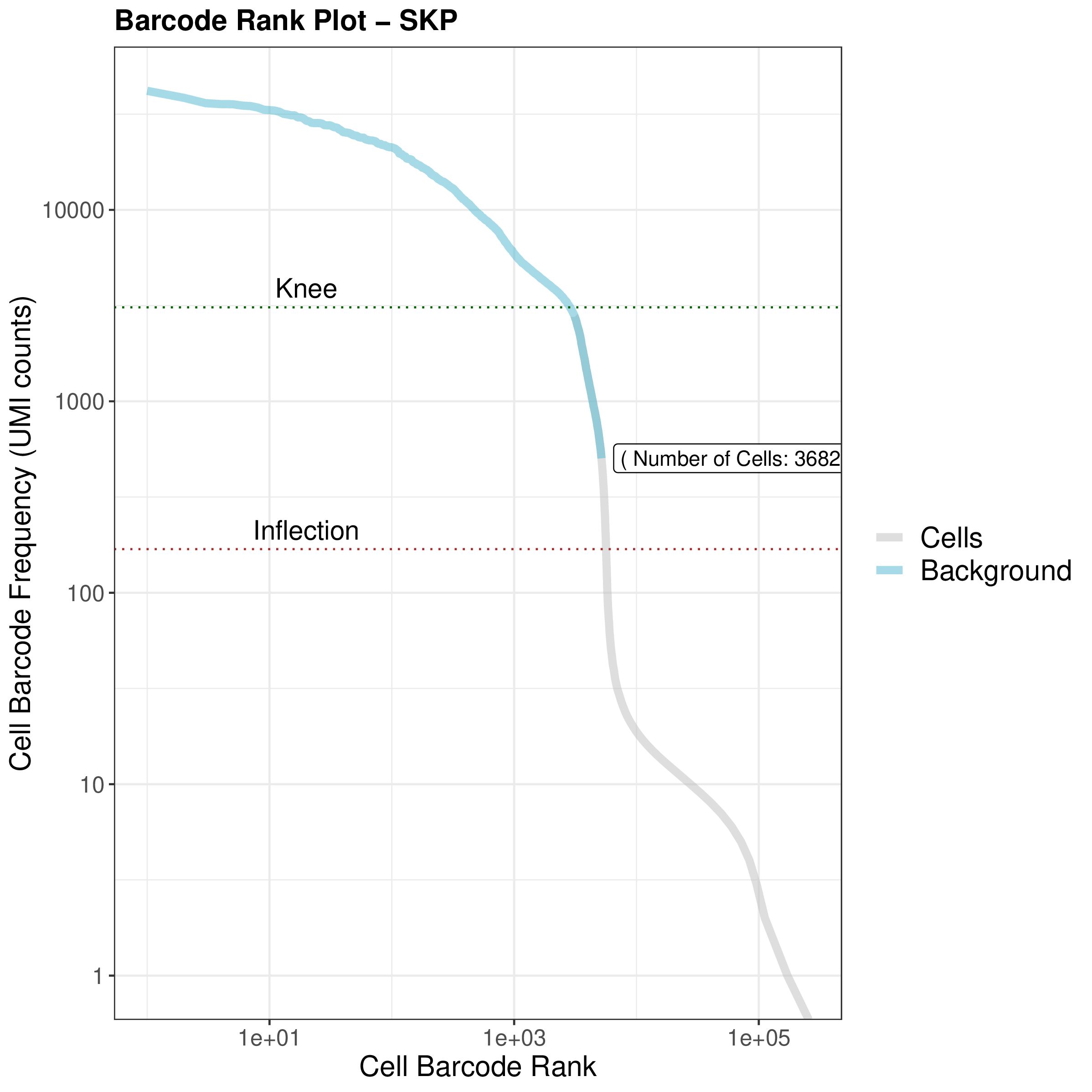

Supplement: Supplementary file 2 — Additional file 2: Supplementary file 2. To demonstrate the utility of scQCEA, we apply the workflow to the sixteen gene expression profiles of eight patients with metastatic melanoma, prepared from pre- and post-treatment experimental batches. You can find the QC interactive report at: https://github.com/isarnassiri/scQCEA/tree/Example-of-Application. Download and unzip the OGC_Interactive_QC_Report_P180121.zip file. You can open CLICK_ME.html file without using rStudio/R. [file 12864_2023_9447_MOESM2_ESM.zip › Inputs/10X-gex-grouped/FAI5649A24/P180121-keep_FAI5649A24_BarcodeRankPlot_10X.png]

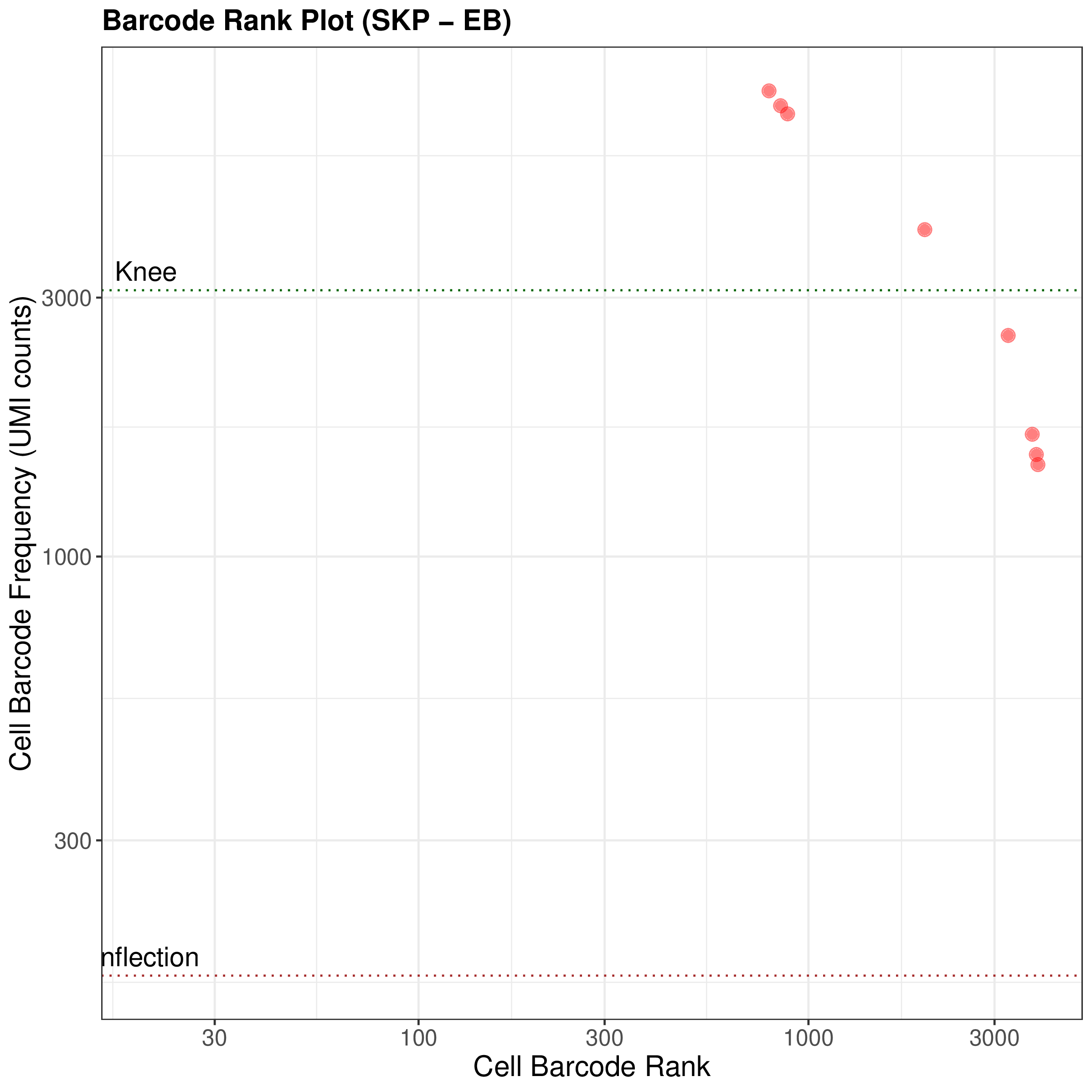

Supplement: Supplementary file 2 — Additional file 2: Supplementary file 2. To demonstrate the utility of scQCEA, we apply the workflow to the sixteen gene expression profiles of eight patients with metastatic melanoma, prepared from pre- and post-treatment experimental batches. You can find the QC interactive report at: https://github.com/isarnassiri/scQCEA/tree/Example-of-Application. Download and unzip the OGC_Interactive_QC_Report_P180121.zip file. You can open CLICK_ME.html file without using rStudio/R. [file 12864_2023_9447_MOESM2_ESM.zip › Inputs/10X-gex-grouped/FAI5649A24/P180121-keep_FAI5649A24_BarcodeRankPlot_EB_FilterOut.png]

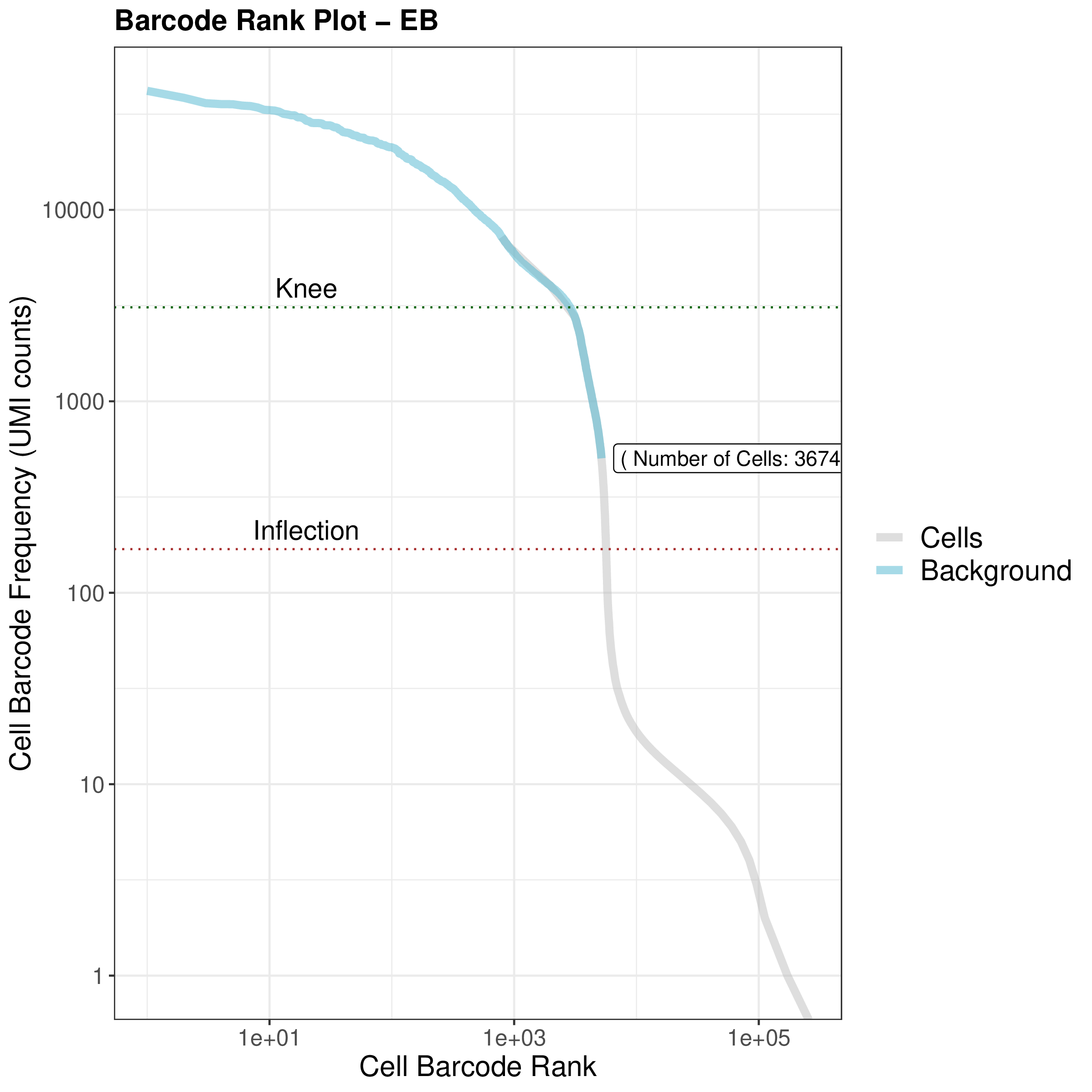

Supplement: Supplementary file 2 — Additional file 2: Supplementary file 2. To demonstrate the utility of scQCEA, we apply the workflow to the sixteen gene expression profiles of eight patients with metastatic melanoma, prepared from pre- and post-treatment experimental batches. You can find the QC interactive report at: https://github.com/isarnassiri/scQCEA/tree/Example-of-Application. Download and unzip the OGC_Interactive_QC_Report_P180121.zip file. You can open CLICK_ME.html file without using rStudio/R. [file 12864_2023_9447_MOESM2_ESM.zip › Inputs/10X-gex-grouped/FAI5649A24/P180121-keep_FAI5649A24_BarcodeRankPlot_EB.png]

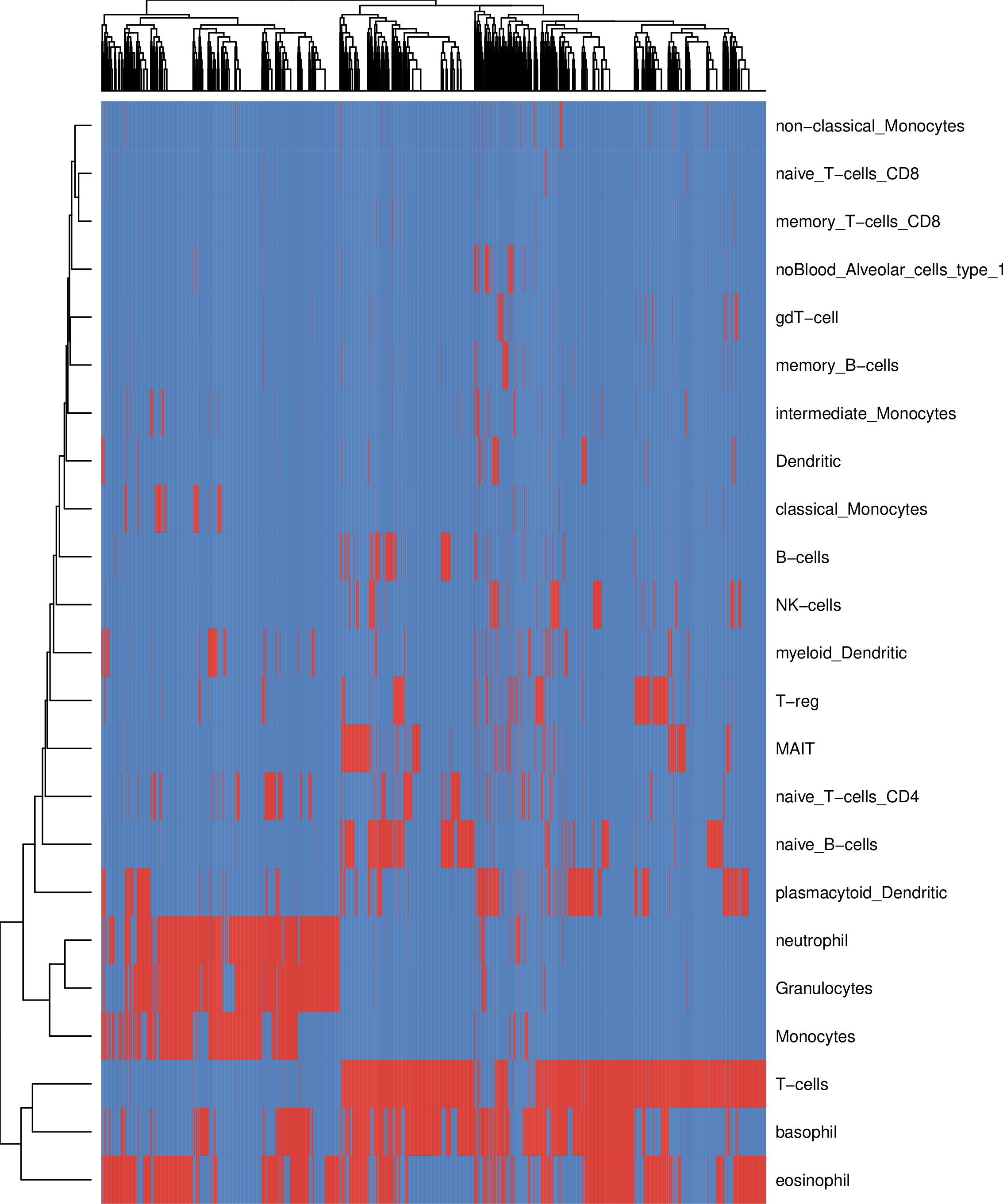

Supplement: Supplementary file 2 — Additional file 2: Supplementary file 2. To demonstrate the utility of scQCEA, we apply the workflow to the sixteen gene expression profiles of eight patients with metastatic melanoma, prepared from pre- and post-treatment experimental batches. You can find the QC interactive report at: https://github.com/isarnassiri/scQCEA/tree/Example-of-Application. Download and unzip the OGC_Interactive_QC_Report_P180121.zip file. You can open CLICK_ME.html file without using rStudio/R. [file 12864_2023_9447_MOESM2_ESM.zip › Inputs/10X-gex-grouped/FAI5649A24/P180121-keep_FAI5649A24_Celltype_assignment_HeatMap.png]

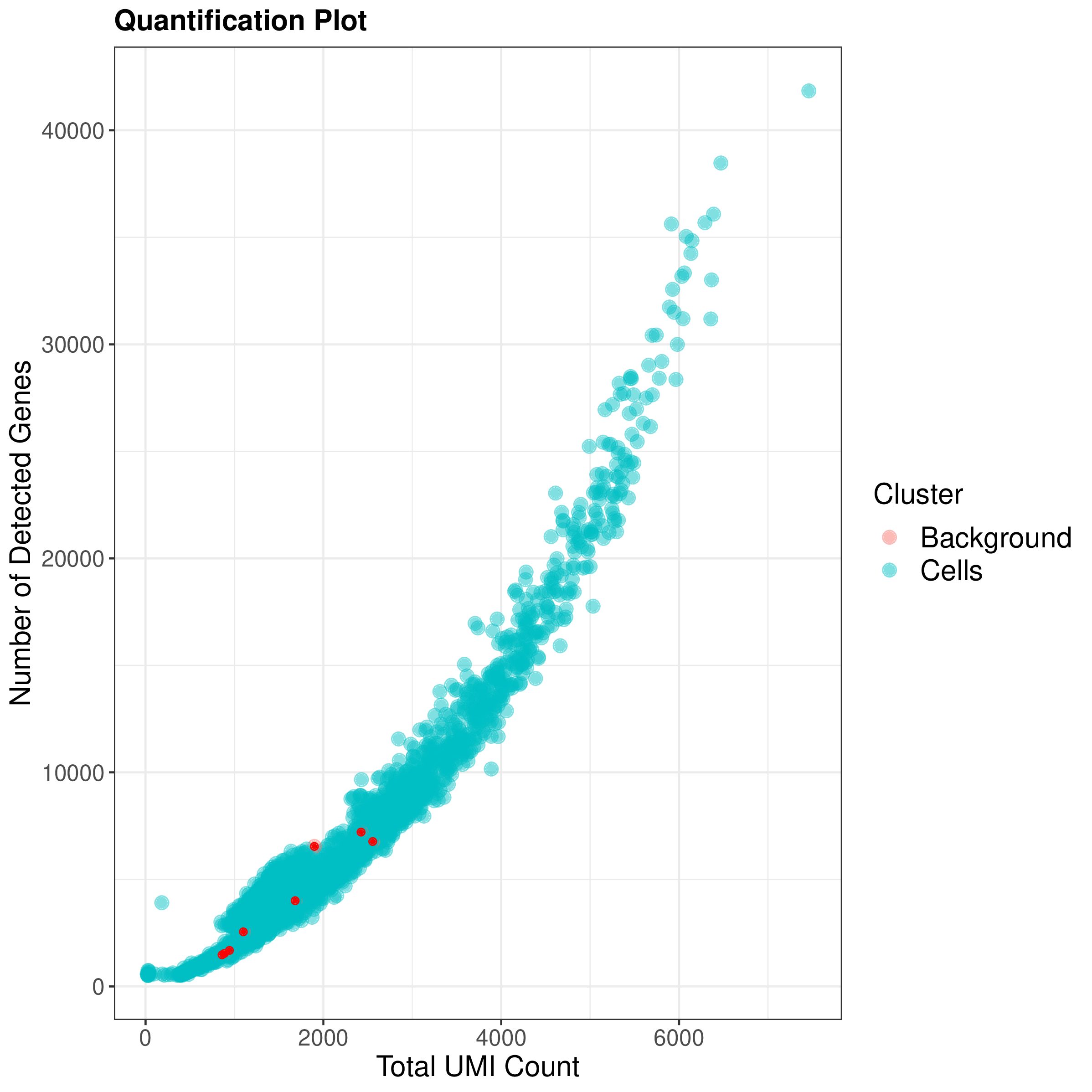

Supplement: Supplementary file 2 — Additional file 2: Supplementary file 2. To demonstrate the utility of scQCEA, we apply the workflow to the sixteen gene expression profiles of eight patients with metastatic melanoma, prepared from pre- and post-treatment experimental batches. You can find the QC interactive report at: https://github.com/isarnassiri/scQCEA/tree/Example-of-Application. Download and unzip the OGC_Interactive_QC_Report_P180121.zip file. You can open CLICK_ME.html file without using rStudio/R. [file 12864_2023_9447_MOESM2_ESM.zip › Inputs/10X-gex-grouped/FAI5649A24/P180121-keep_FAI5649A24_TotalUMIvsDetectedGenes.png]

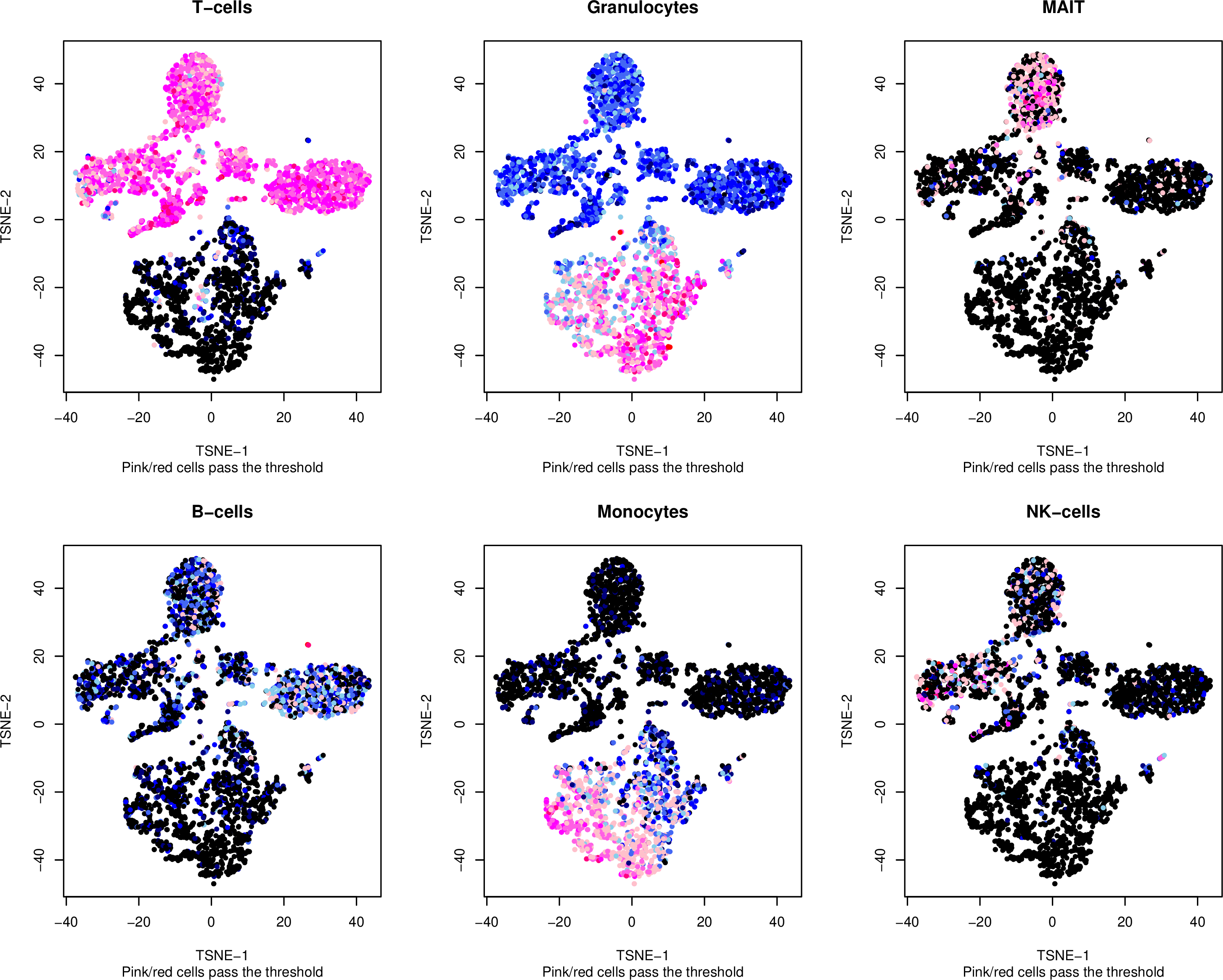

Supplement: Supplementary file 2 — Additional file 2: Supplementary file 2. To demonstrate the utility of scQCEA, we apply the workflow to the sixteen gene expression profiles of eight patients with metastatic melanoma, prepared from pre- and post-treatment experimental batches. You can find the QC interactive report at: https://github.com/isarnassiri/scQCEA/tree/Example-of-Application. Download and unzip the OGC_Interactive_QC_Report_P180121.zip file. You can open CLICK_ME.html file without using rStudio/R. [file 12864_2023_9447_MOESM2_ESM.zip › Inputs/10X-gex-grouped/FAI5649A24/P180121-keep_FAI5649A24_tSNE_Plot.png]

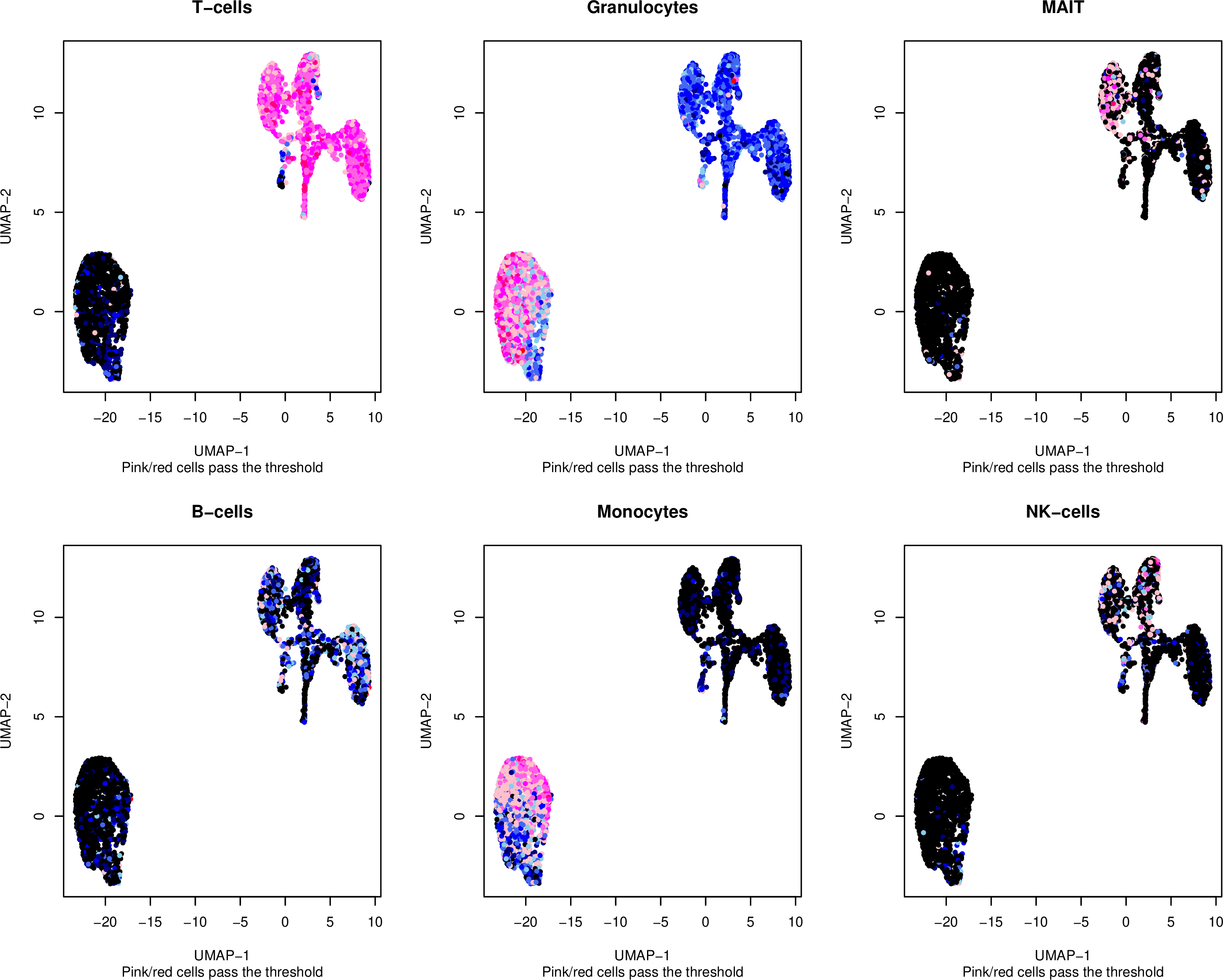

Supplement: Supplementary file 2 — Additional file 2: Supplementary file 2. To demonstrate the utility of scQCEA, we apply the workflow to the sixteen gene expression profiles of eight patients with metastatic melanoma, prepared from pre- and post-treatment experimental batches. You can find the QC interactive report at: https://github.com/isarnassiri/scQCEA/tree/Example-of-Application. Download and unzip the OGC_Interactive_QC_Report_P180121.zip file. You can open CLICK_ME.html file without using rStudio/R. [file 12864_2023_9447_MOESM2_ESM.zip › Inputs/10X-gex-grouped/FAI5649A24/P180121-keep_FAI5649A24_UMAP_Plot.png]

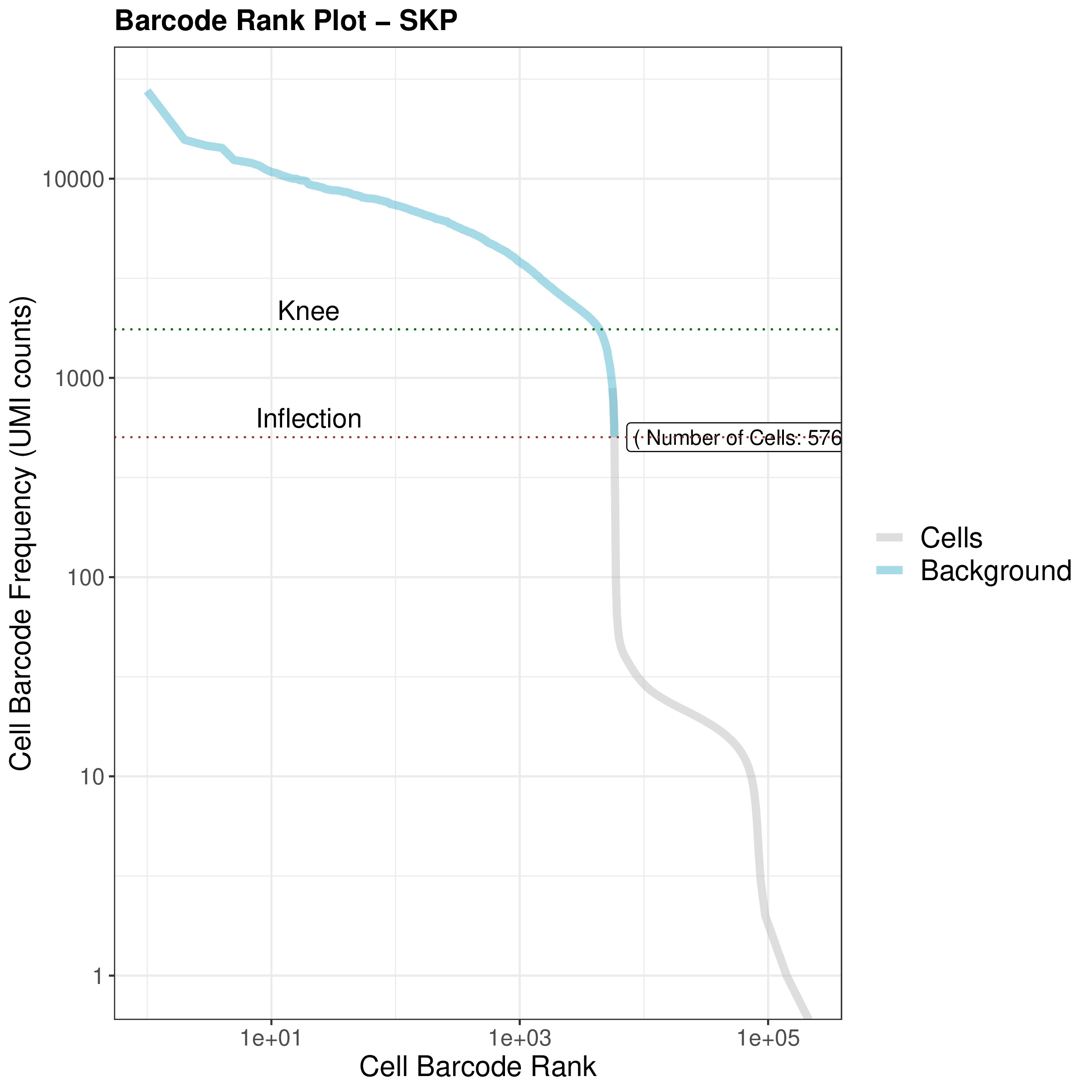

Supplement: Supplementary file 2 — Additional file 2: Supplementary file 2. To demonstrate the utility of scQCEA, we apply the workflow to the sixteen gene expression profiles of eight patients with metastatic melanoma, prepared from pre- and post-treatment experimental batches. You can find the QC interactive report at: https://github.com/isarnassiri/scQCEA/tree/Example-of-Application. Download and unzip the OGC_Interactive_QC_Report_P180121.zip file. You can open CLICK_ME.html file without using rStudio/R. [file 12864_2023_9447_MOESM2_ESM.zip › Inputs/10X-gex/481207_03/P180121-keep_481207_03_BarcodeRankPlot_10X.png]

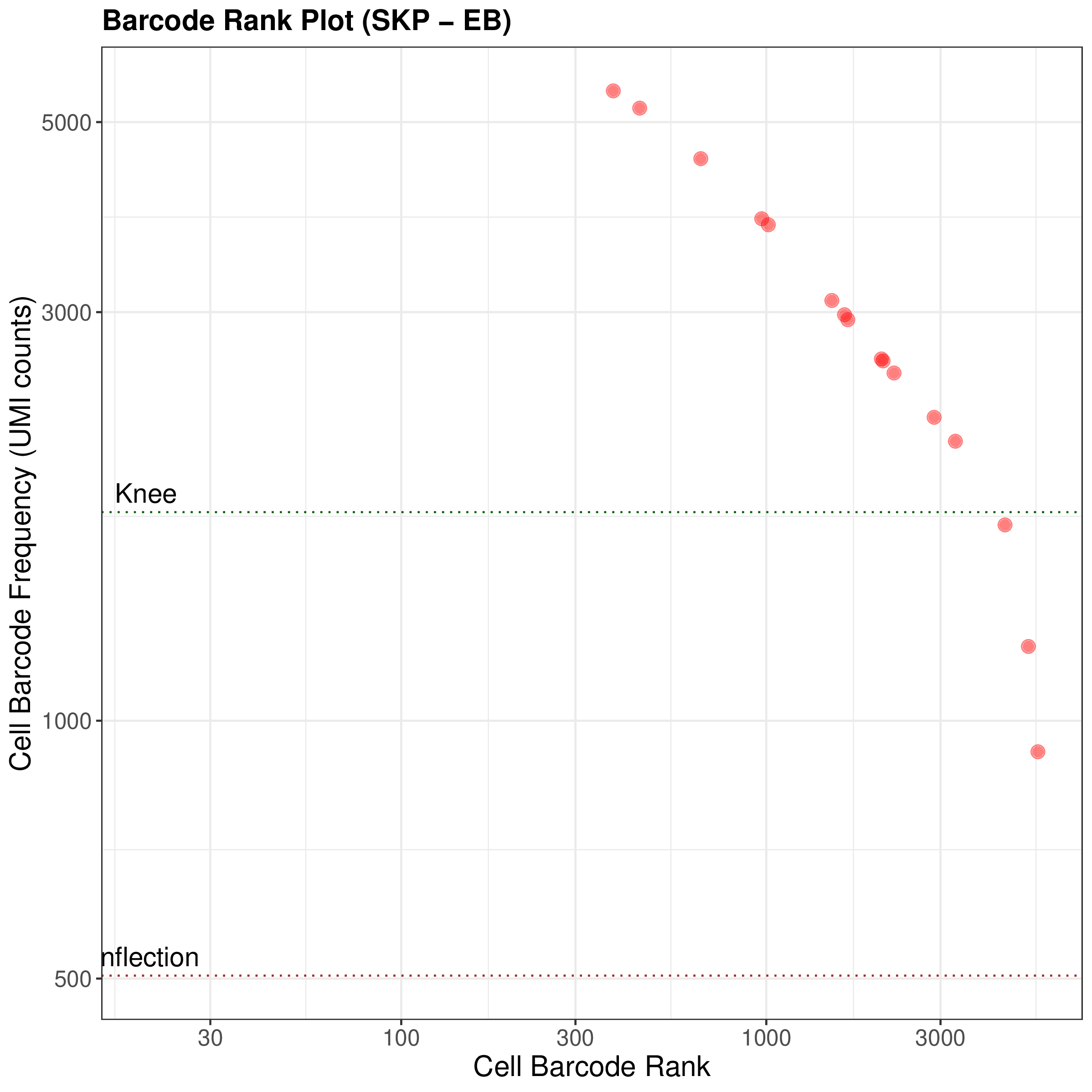

Supplement: Supplementary file 2 — Additional file 2: Supplementary file 2. To demonstrate the utility of scQCEA, we apply the workflow to the sixteen gene expression profiles of eight patients with metastatic melanoma, prepared from pre- and post-treatment experimental batches. You can find the QC interactive report at: https://github.com/isarnassiri/scQCEA/tree/Example-of-Application. Download and unzip the OGC_Interactive_QC_Report_P180121.zip file. You can open CLICK_ME.html file without using rStudio/R. [file 12864_2023_9447_MOESM2_ESM.zip › Inputs/10X-gex/481207_03/P180121-keep_481207_03_BarcodeRankPlot_EB_FilterOut.png]

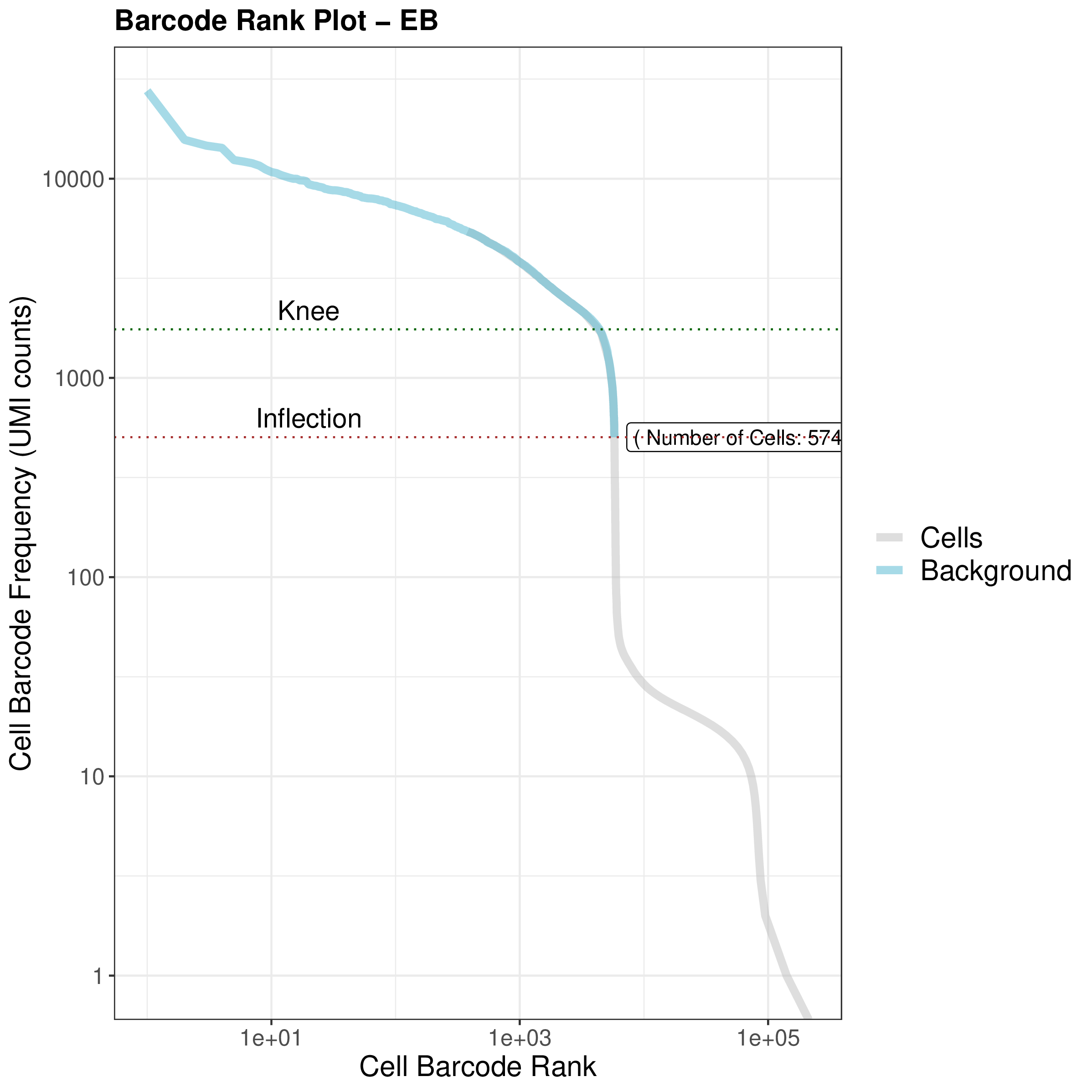

Supplement: Supplementary file 2 — Additional file 2: Supplementary file 2. To demonstrate the utility of scQCEA, we apply the workflow to the sixteen gene expression profiles of eight patients with metastatic melanoma, prepared from pre- and post-treatment experimental batches. You can find the QC interactive report at: https://github.com/isarnassiri/scQCEA/tree/Example-of-Application. Download and unzip the OGC_Interactive_QC_Report_P180121.zip file. You can open CLICK_ME.html file without using rStudio/R. [file 12864_2023_9447_MOESM2_ESM.zip › Inputs/10X-gex/481207_03/P180121-keep_481207_03_BarcodeRankPlot_EB.png]

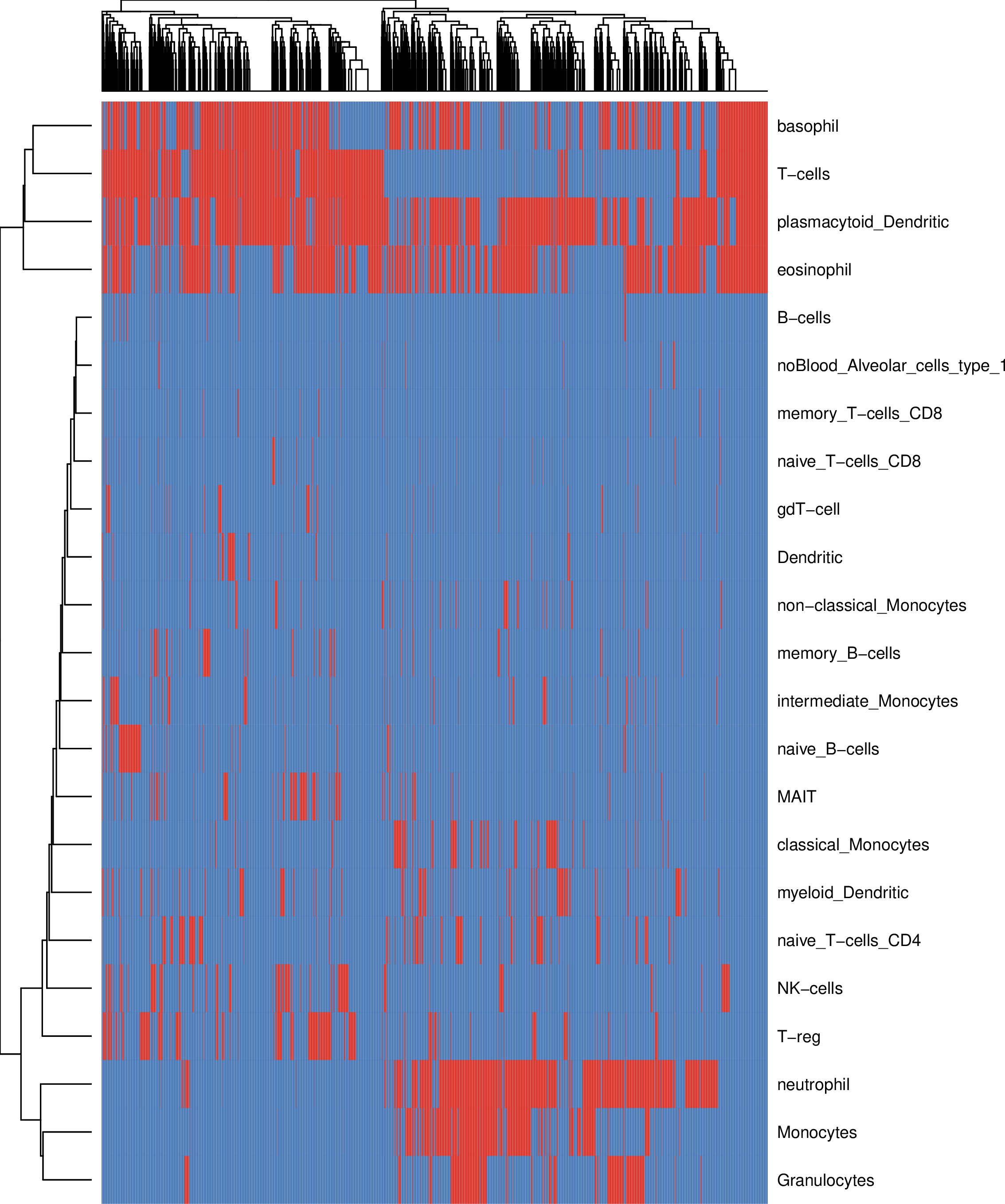

Supplement: Supplementary file 2 — Additional file 2: Supplementary file 2. To demonstrate the utility of scQCEA, we apply the workflow to the sixteen gene expression profiles of eight patients with metastatic melanoma, prepared from pre- and post-treatment experimental batches. You can find the QC interactive report at: https://github.com/isarnassiri/scQCEA/tree/Example-of-Application. Download and unzip the OGC_Interactive_QC_Report_P180121.zip file. You can open CLICK_ME.html file without using rStudio/R. [file 12864_2023_9447_MOESM2_ESM.zip › Inputs/10X-gex/481207_03/P180121-keep_481207_03_Celltype_assignment_HeatMap.png]

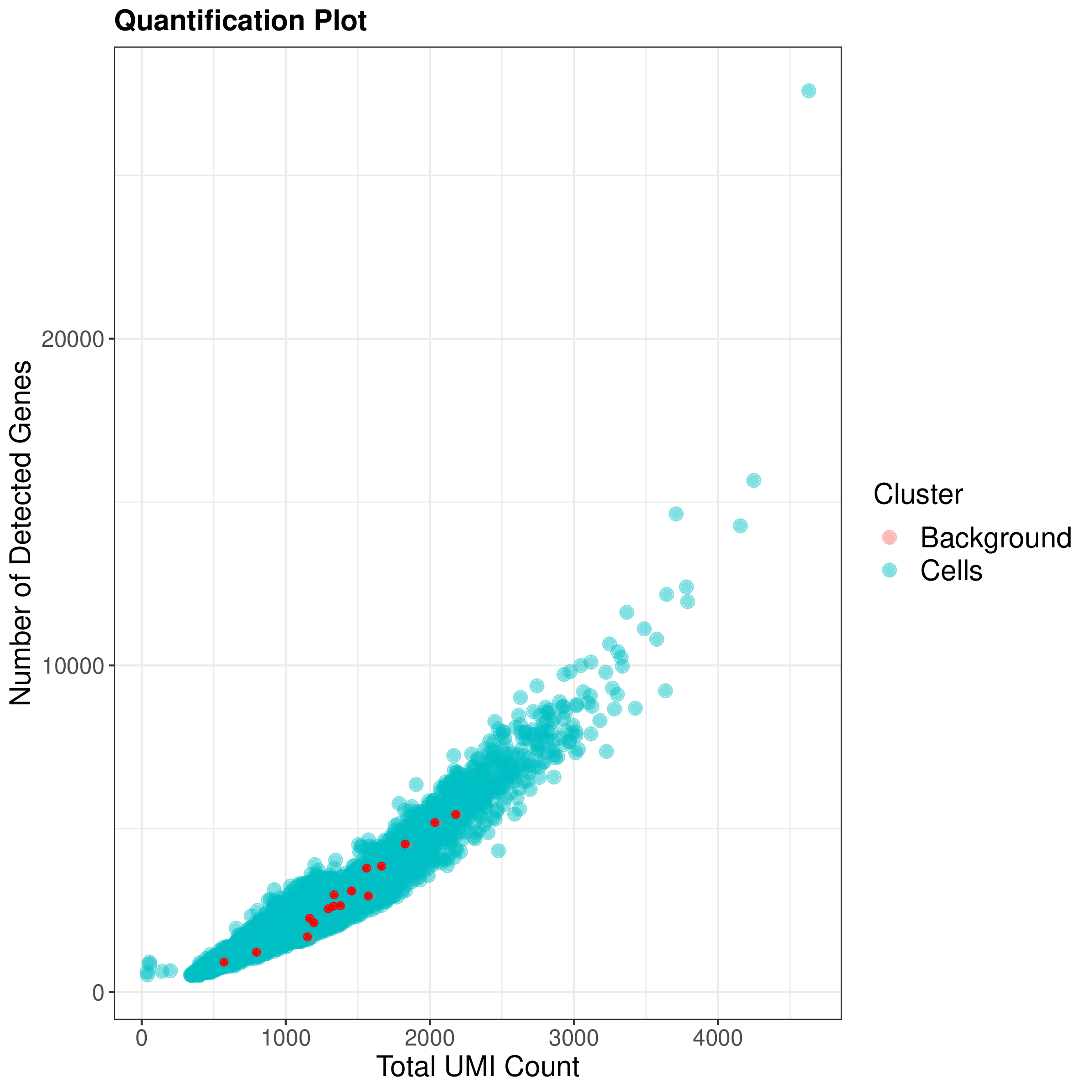

Supplement: Supplementary file 2 — Additional file 2: Supplementary file 2. To demonstrate the utility of scQCEA, we apply the workflow to the sixteen gene expression profiles of eight patients with metastatic melanoma, prepared from pre- and post-treatment experimental batches. You can find the QC interactive report at: https://github.com/isarnassiri/scQCEA/tree/Example-of-Application. Download and unzip the OGC_Interactive_QC_Report_P180121.zip file. You can open CLICK_ME.html file without using rStudio/R. [file 12864_2023_9447_MOESM2_ESM.zip › Inputs/10X-gex/481207_03/P180121-keep_481207_03_TotalUMIvsDetectedGenes.png]

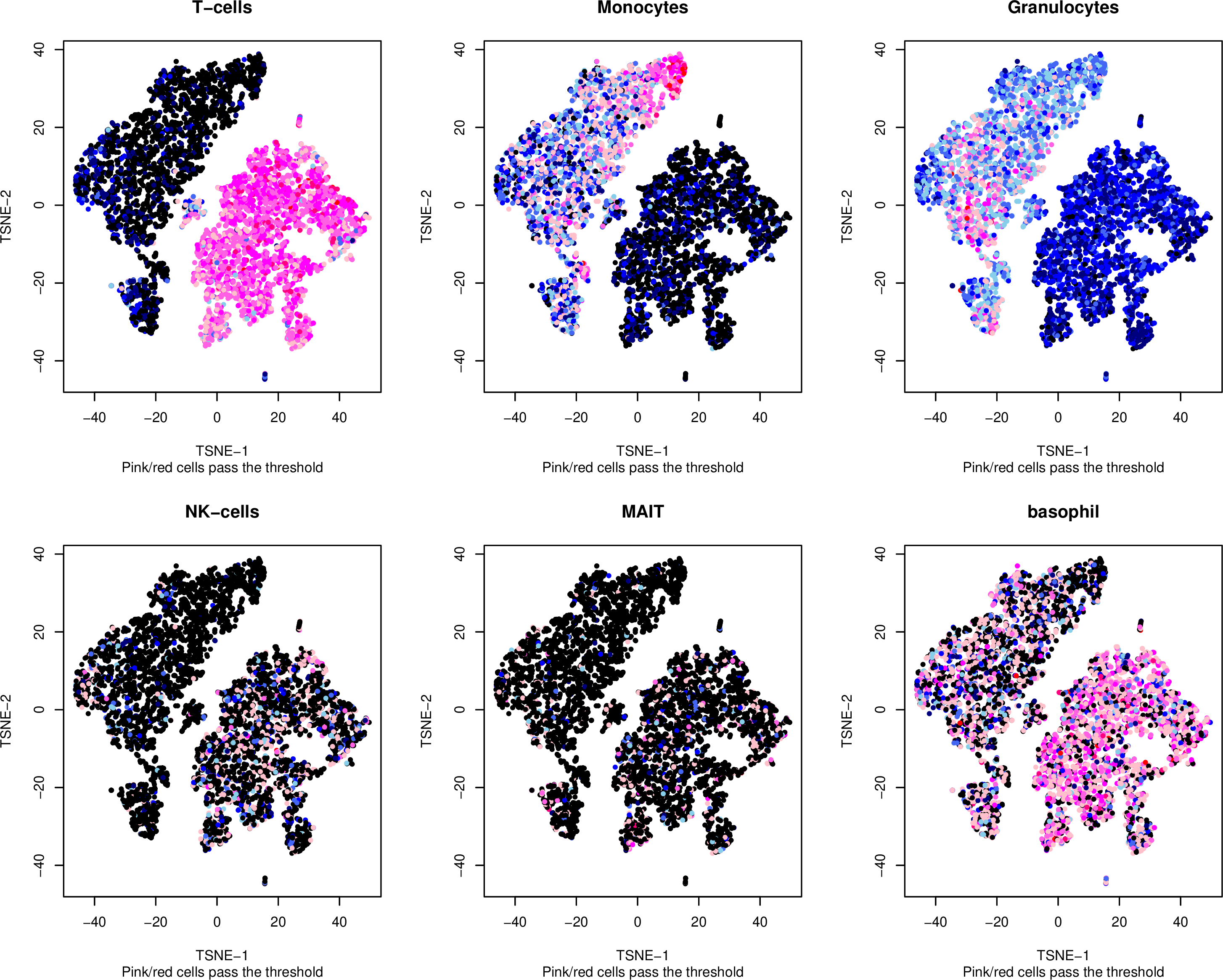

Supplement: Supplementary file 2 — Additional file 2: Supplementary file 2. To demonstrate the utility of scQCEA, we apply the workflow to the sixteen gene expression profiles of eight patients with metastatic melanoma, prepared from pre- and post-treatment experimental batches. You can find the QC interactive report at: https://github.com/isarnassiri/scQCEA/tree/Example-of-Application. Download and unzip the OGC_Interactive_QC_Report_P180121.zip file. You can open CLICK_ME.html file without using rStudio/R. [file 12864_2023_9447_MOESM2_ESM.zip › Inputs/10X-gex/481207_03/P180121-keep_481207_03_tSNE_Plot.png]

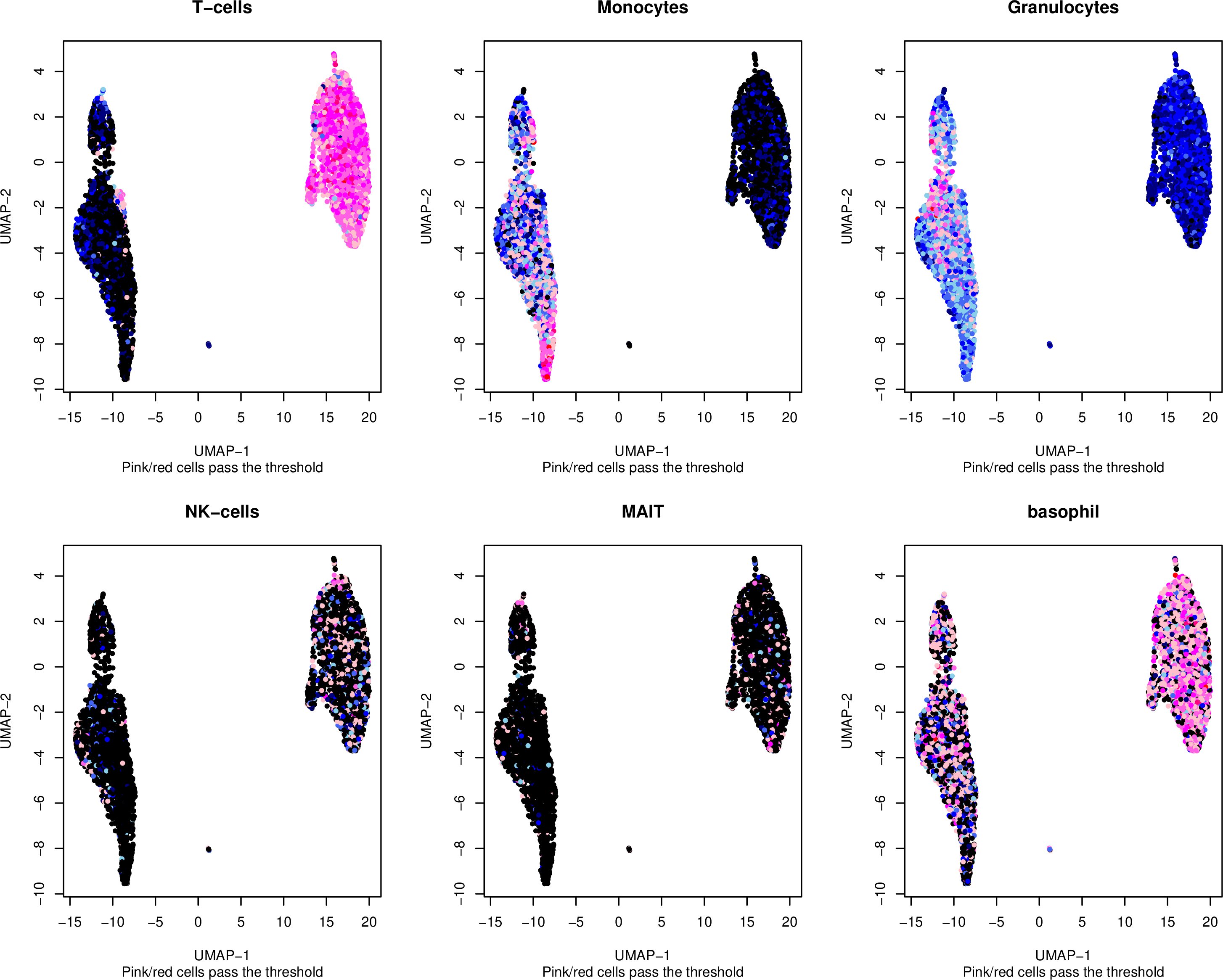

Supplement: Supplementary file 2 — Additional file 2: Supplementary file 2. To demonstrate the utility of scQCEA, we apply the workflow to the sixteen gene expression profiles of eight patients with metastatic melanoma, prepared from pre- and post-treatment experimental batches. You can find the QC interactive report at: https://github.com/isarnassiri/scQCEA/tree/Example-of-Application. Download and unzip the OGC_Interactive_QC_Report_P180121.zip file. You can open CLICK_ME.html file without using rStudio/R. [file 12864_2023_9447_MOESM2_ESM.zip › Inputs/10X-gex/481207_03/P180121-keep_481207_03_UMAP_Plot.png]

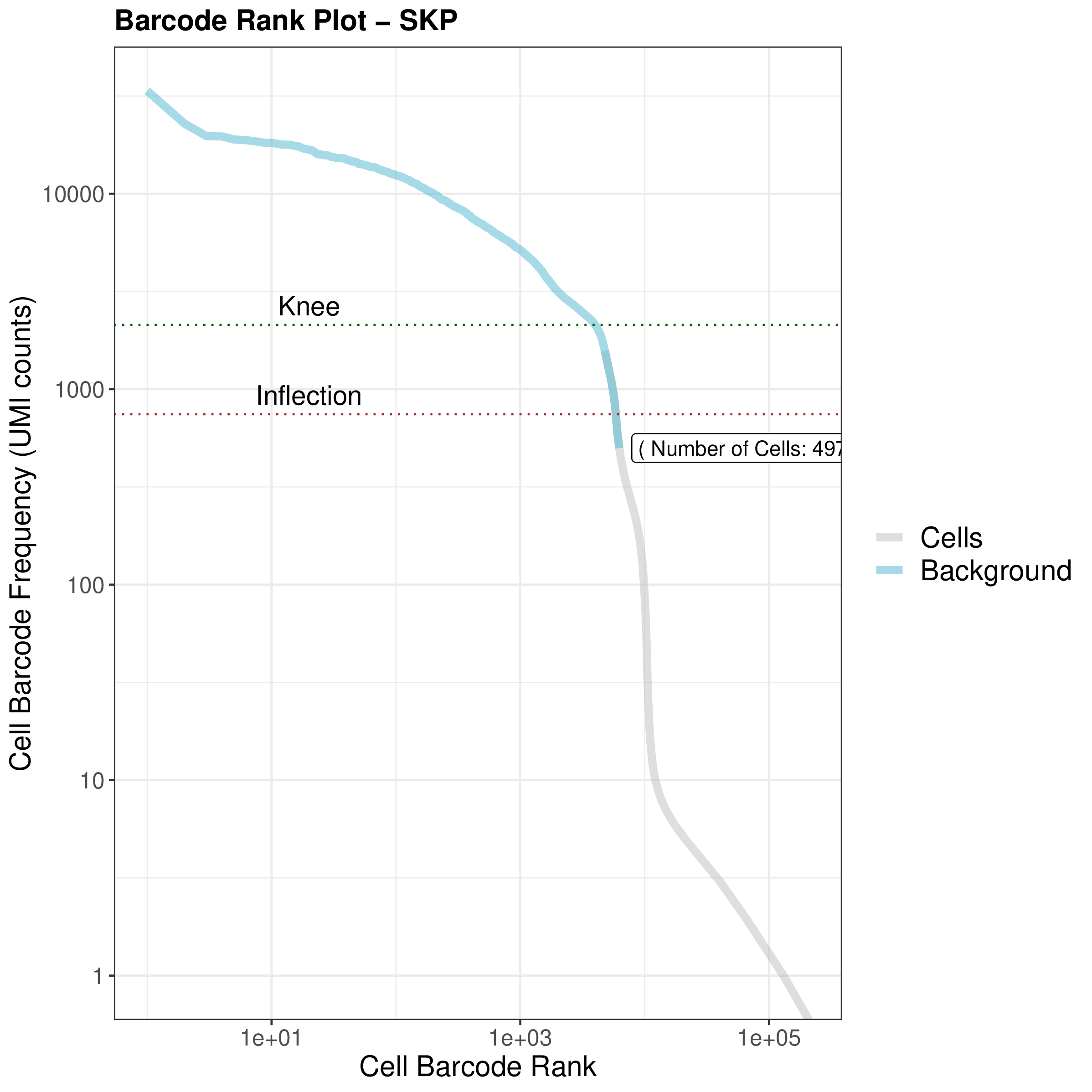

Supplement: Supplementary file 2 — Additional file 2: Supplementary file 2. To demonstrate the utility of scQCEA, we apply the workflow to the sixteen gene expression profiles of eight patients with metastatic melanoma, prepared from pre- and post-treatment experimental batches. You can find the QC interactive report at: https://github.com/isarnassiri/scQCEA/tree/Example-of-Application. Download and unzip the OGC_Interactive_QC_Report_P180121.zip file. You can open CLICK_ME.html file without using rStudio/R. [file 12864_2023_9447_MOESM2_ESM.zip › Inputs/10X-gex/481207_15/P180121-keep_481207_15_BarcodeRankPlot_10X.png]

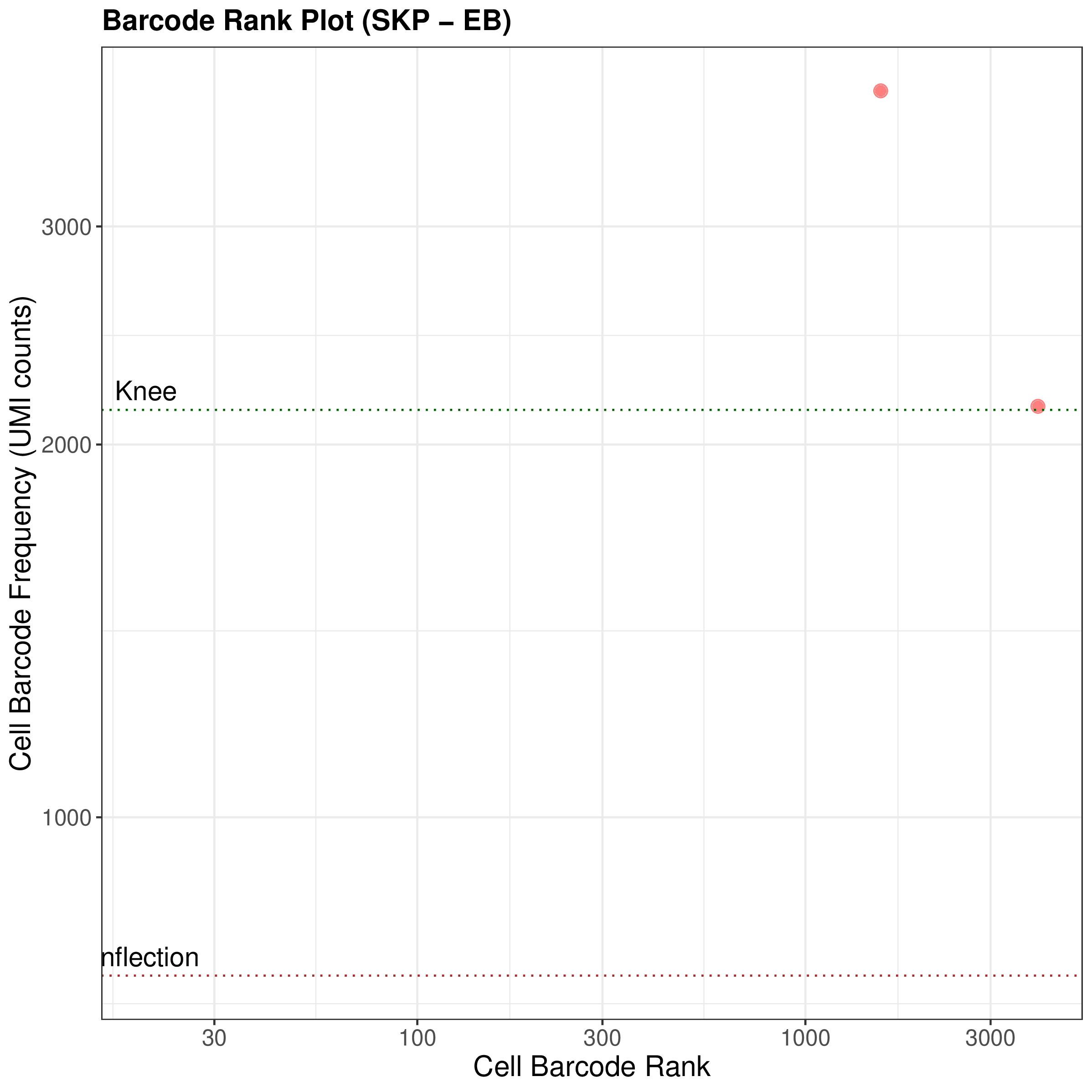

Supplement: Supplementary file 2 — Additional file 2: Supplementary file 2. To demonstrate the utility of scQCEA, we apply the workflow to the sixteen gene expression profiles of eight patients with metastatic melanoma, prepared from pre- and post-treatment experimental batches. You can find the QC interactive report at: https://github.com/isarnassiri/scQCEA/tree/Example-of-Application. Download and unzip the OGC_Interactive_QC_Report_P180121.zip file. You can open CLICK_ME.html file without using rStudio/R. [file 12864_2023_9447_MOESM2_ESM.zip › Inputs/10X-gex/481207_15/P180121-keep_481207_15_BarcodeRankPlot_EB_FilterOut.png]

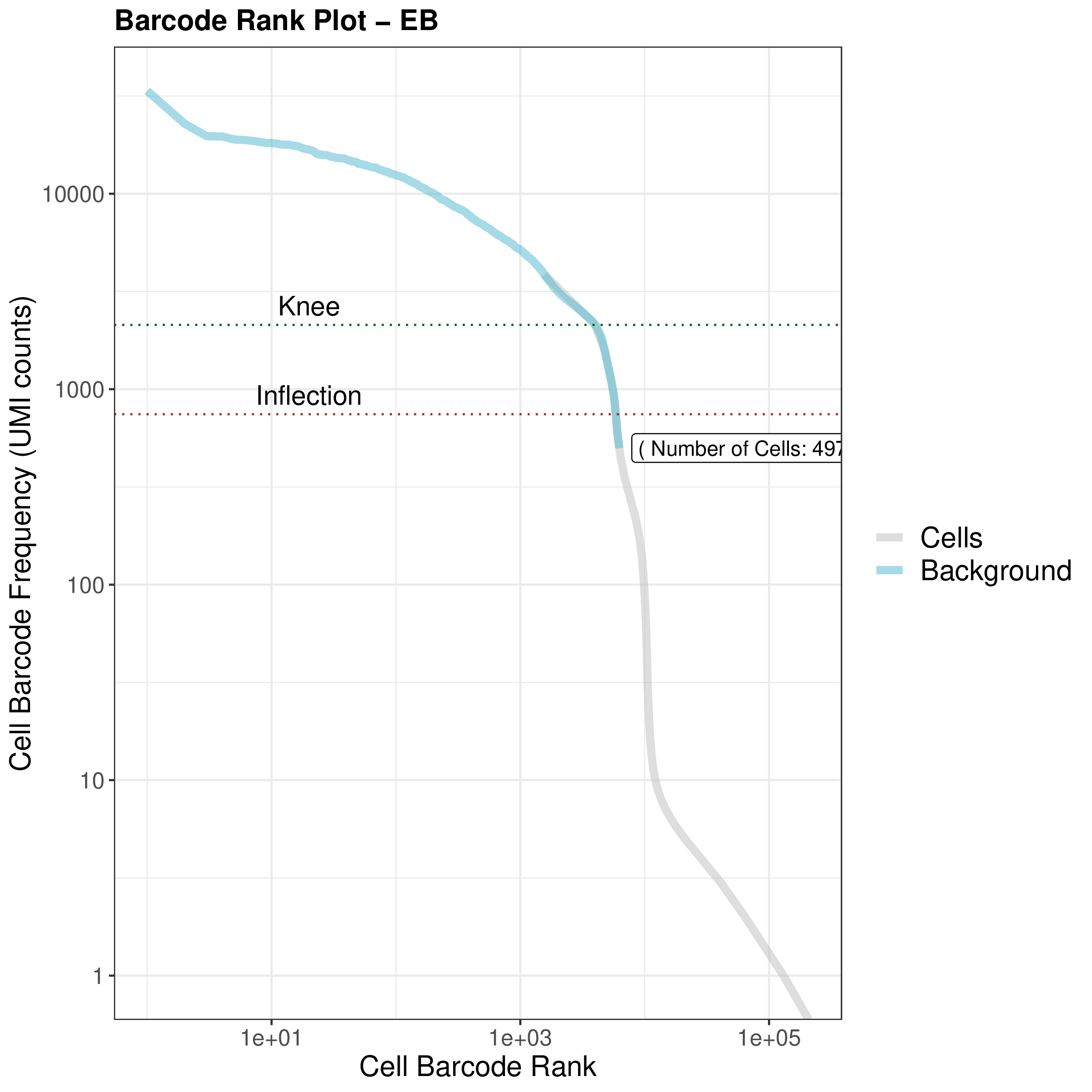

Supplement: Supplementary file 2 — Additional file 2: Supplementary file 2. To demonstrate the utility of scQCEA, we apply the workflow to the sixteen gene expression profiles of eight patients with metastatic melanoma, prepared from pre- and post-treatment experimental batches. You can find the QC interactive report at: https://github.com/isarnassiri/scQCEA/tree/Example-of-Application. Download and unzip the OGC_Interactive_QC_Report_P180121.zip file. You can open CLICK_ME.html file without using rStudio/R. [file 12864_2023_9447_MOESM2_ESM.zip › Inputs/10X-gex/481207_15/P180121-keep_481207_15_BarcodeRankPlot_EB.png]

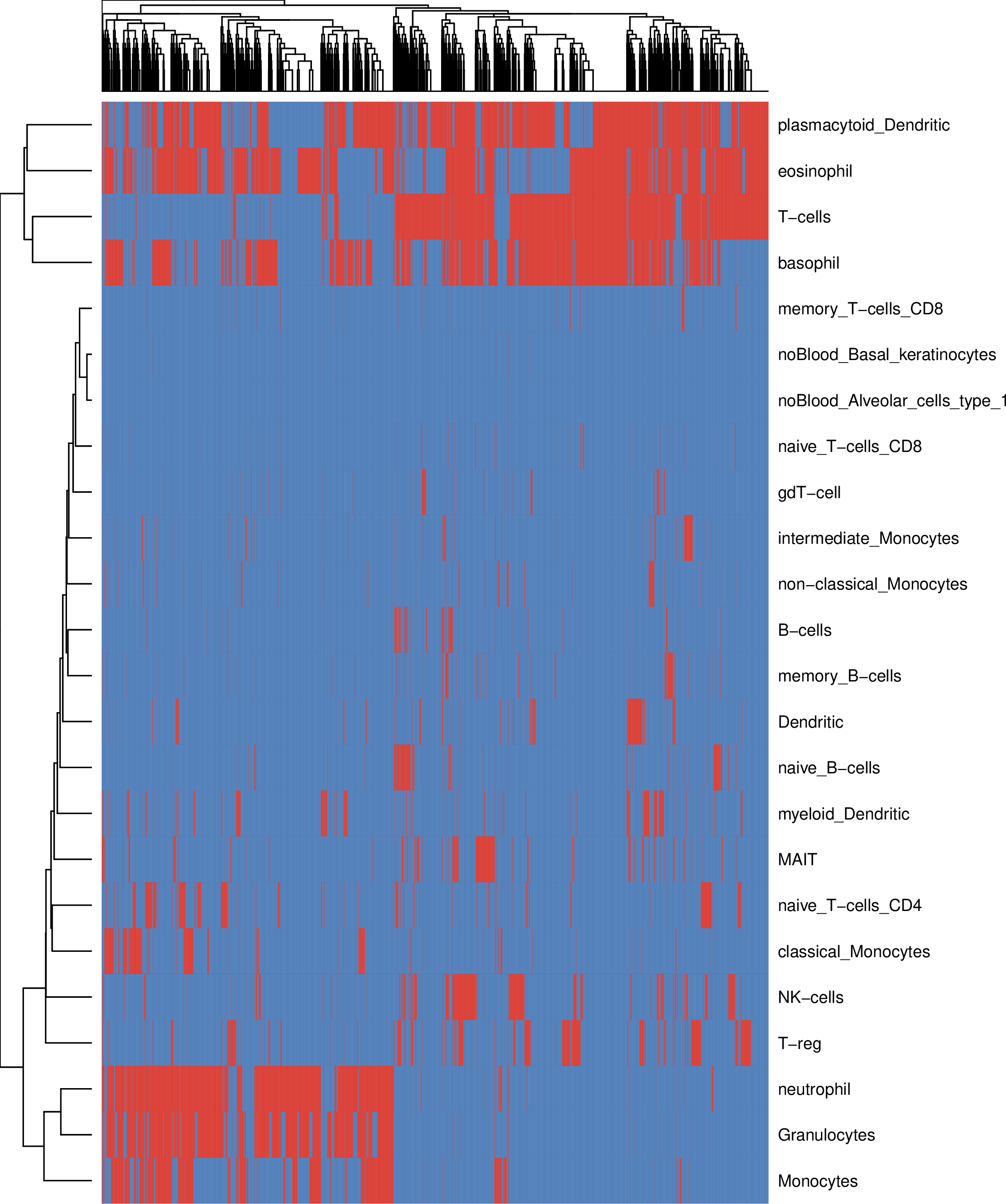

Supplement: Supplementary file 2 — Additional file 2: Supplementary file 2. To demonstrate the utility of scQCEA, we apply the workflow to the sixteen gene expression profiles of eight patients with metastatic melanoma, prepared from pre- and post-treatment experimental batches. You can find the QC interactive report at: https://github.com/isarnassiri/scQCEA/tree/Example-of-Application. Download and unzip the OGC_Interactive_QC_Report_P180121.zip file. You can open CLICK_ME.html file without using rStudio/R. [file 12864_2023_9447_MOESM2_ESM.zip › Inputs/10X-gex/481207_15/P180121-keep_481207_15_Celltype_assignment_HeatMap.png]

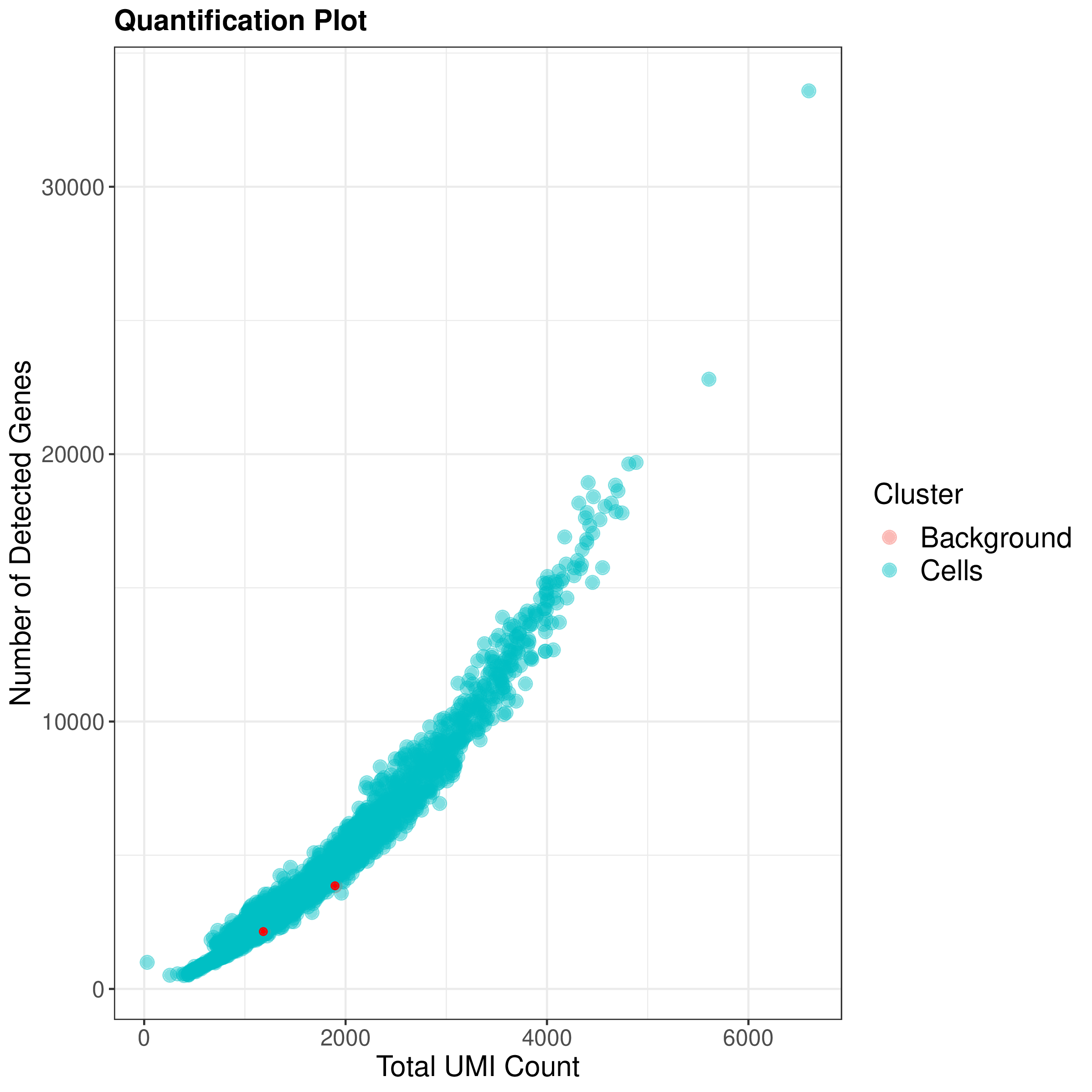

Supplement: Supplementary file 2 — Additional file 2: Supplementary file 2. To demonstrate the utility of scQCEA, we apply the workflow to the sixteen gene expression profiles of eight patients with metastatic melanoma, prepared from pre- and post-treatment experimental batches. You can find the QC interactive report at: https://github.com/isarnassiri/scQCEA/tree/Example-of-Application. Download and unzip the OGC_Interactive_QC_Report_P180121.zip file. You can open CLICK_ME.html file without using rStudio/R. [file 12864_2023_9447_MOESM2_ESM.zip › Inputs/10X-gex/481207_15/P180121-keep_481207_15_TotalUMIvsDetectedGenes.png]

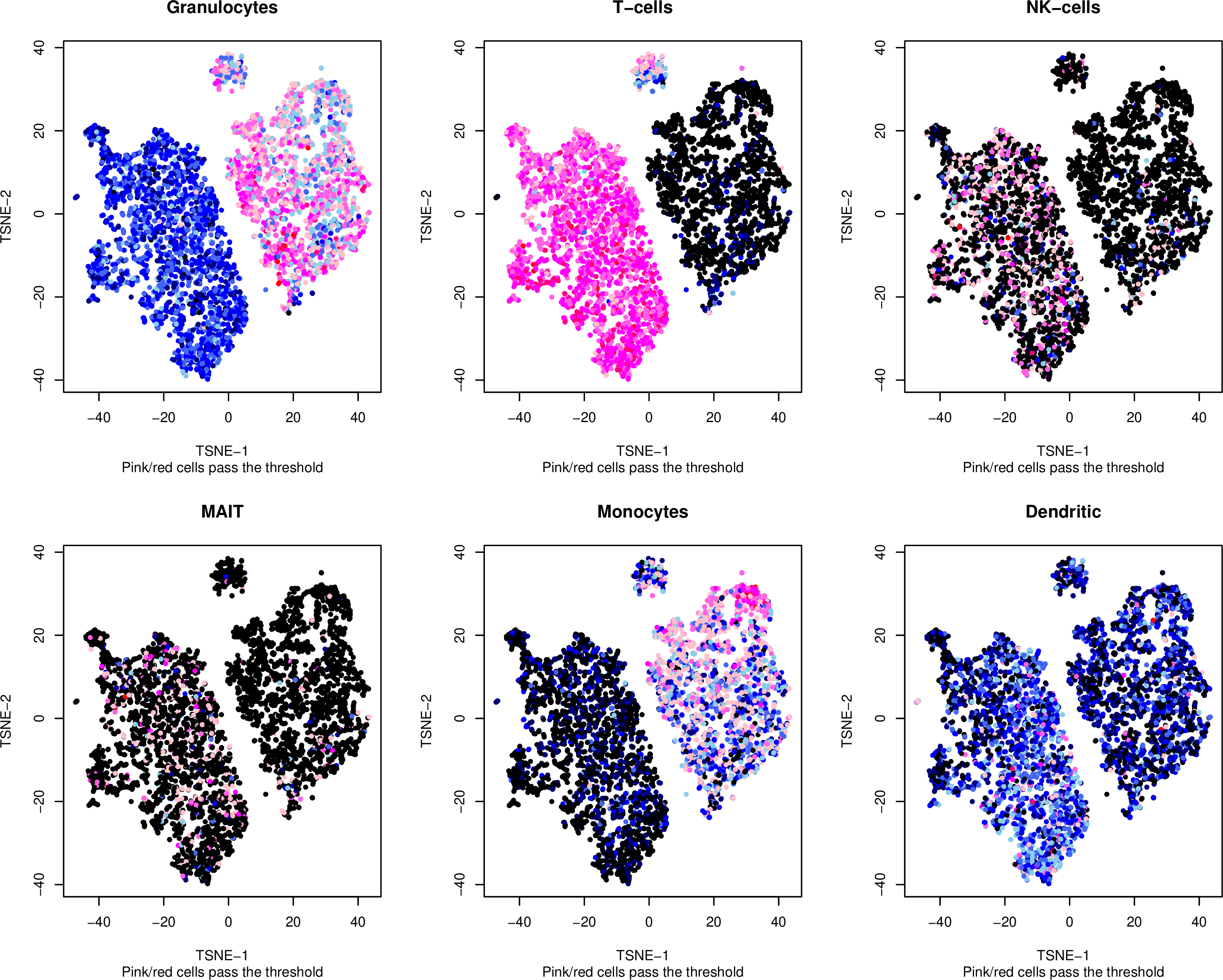

Supplement: Supplementary file 2 — Additional file 2: Supplementary file 2. To demonstrate the utility of scQCEA, we apply the workflow to the sixteen gene expression profiles of eight patients with metastatic melanoma, prepared from pre- and post-treatment experimental batches. You can find the QC interactive report at: https://github.com/isarnassiri/scQCEA/tree/Example-of-Application. Download and unzip the OGC_Interactive_QC_Report_P180121.zip file. You can open CLICK_ME.html file without using rStudio/R. [file 12864_2023_9447_MOESM2_ESM.zip › Inputs/10X-gex/481207_15/P180121-keep_481207_15_tSNE_Plot.png]

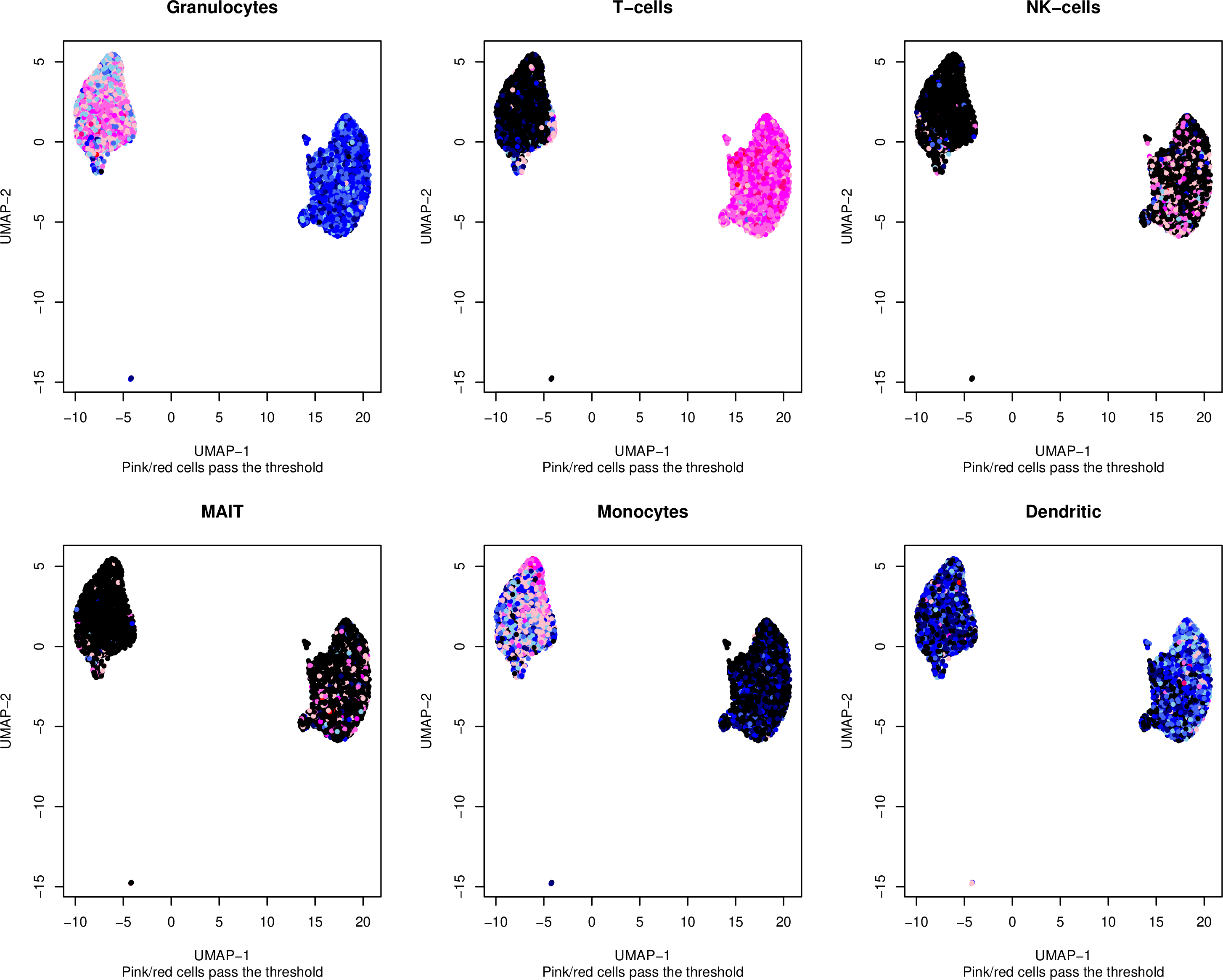

Supplement: Supplementary file 2 — Additional file 2: Supplementary file 2. To demonstrate the utility of scQCEA, we apply the workflow to the sixteen gene expression profiles of eight patients with metastatic melanoma, prepared from pre- and post-treatment experimental batches. You can find the QC interactive report at: https://github.com/isarnassiri/scQCEA/tree/Example-of-Application. Download and unzip the OGC_Interactive_QC_Report_P180121.zip file. You can open CLICK_ME.html file without using rStudio/R. [file 12864_2023_9447_MOESM2_ESM.zip › Inputs/10X-gex/481207_15/P180121-keep_481207_15_UMAP_Plot.png]

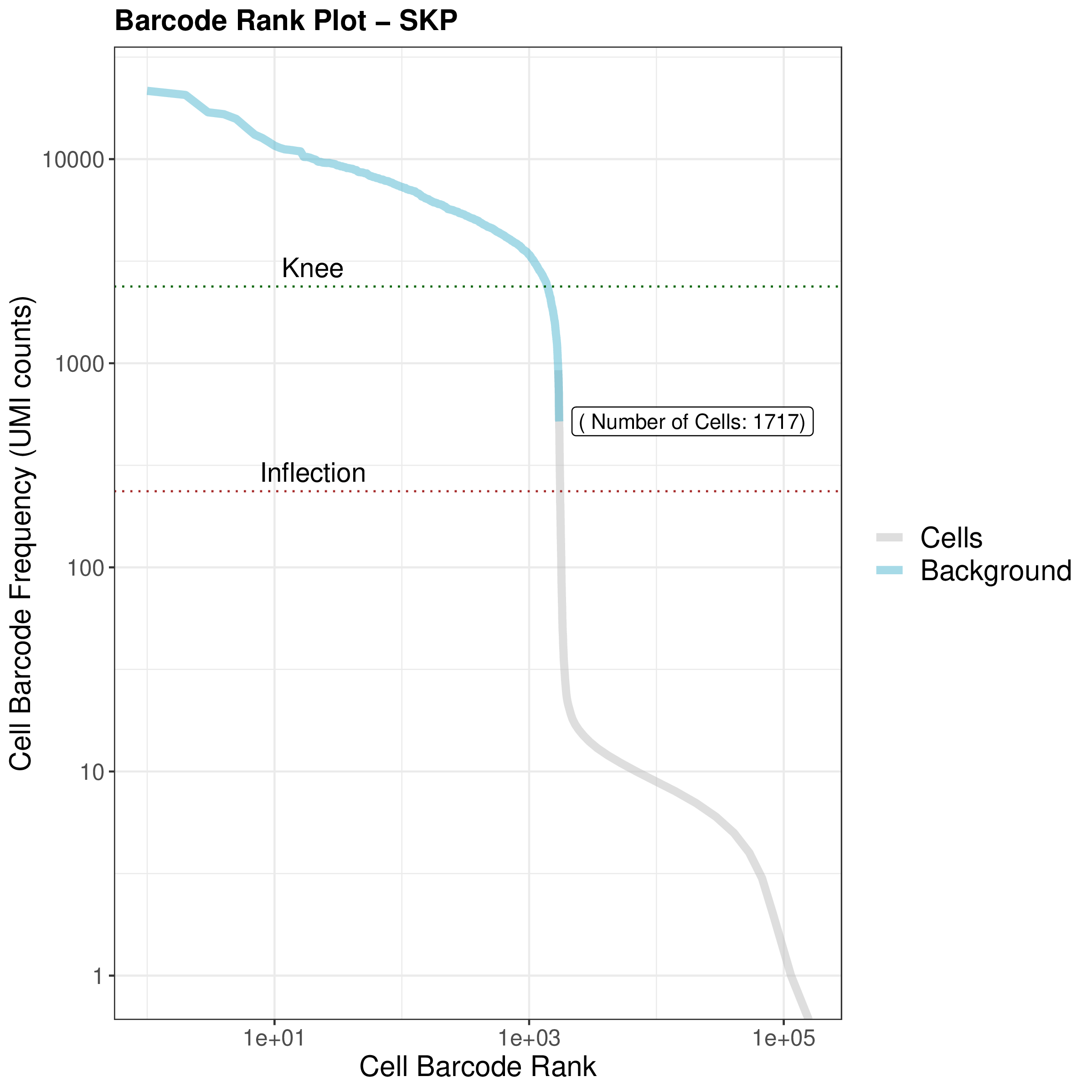

Supplement: Supplementary file 2 — Additional file 2: Supplementary file 2. To demonstrate the utility of scQCEA, we apply the workflow to the sixteen gene expression profiles of eight patients with metastatic melanoma, prepared from pre- and post-treatment experimental batches. You can find the QC interactive report at: https://github.com/isarnassiri/scQCEA/tree/Example-of-Application. Download and unzip the OGC_Interactive_QC_Report_P180121.zip file. You can open CLICK_ME.html file without using rStudio/R. [file 12864_2023_9447_MOESM2_ESM.zip › Inputs/10X-gex/481207_28/P180121-keep_481207_28_BarcodeRankPlot_10X.png]

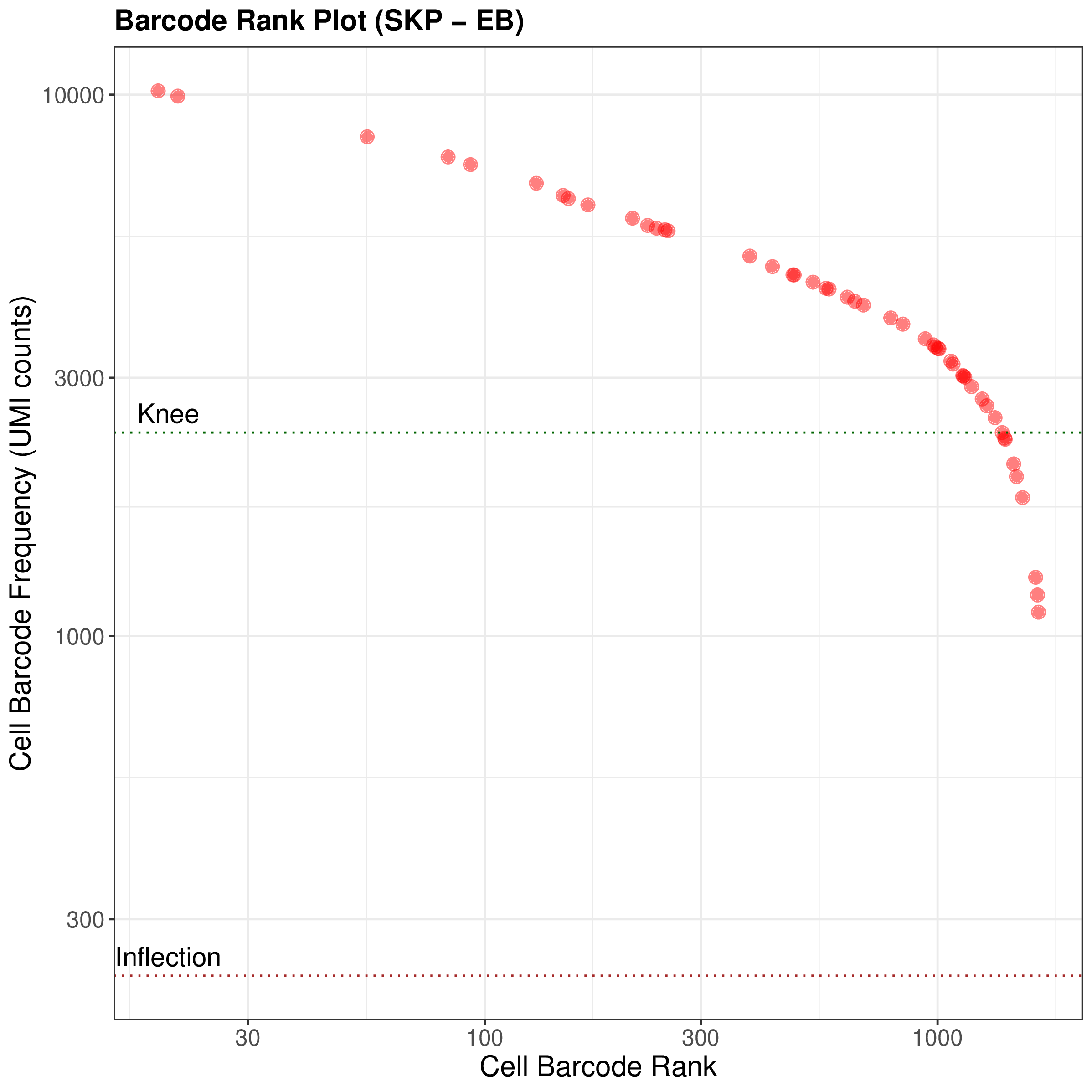

Supplement: Supplementary file 2 — Additional file 2: Supplementary file 2. To demonstrate the utility of scQCEA, we apply the workflow to the sixteen gene expression profiles of eight patients with metastatic melanoma, prepared from pre- and post-treatment experimental batches. You can find the QC interactive report at: https://github.com/isarnassiri/scQCEA/tree/Example-of-Application. Download and unzip the OGC_Interactive_QC_Report_P180121.zip file. You can open CLICK_ME.html file without using rStudio/R. [file 12864_2023_9447_MOESM2_ESM.zip › Inputs/10X-gex/481207_28/P180121-keep_481207_28_BarcodeRankPlot_EB_FilterOut.png]

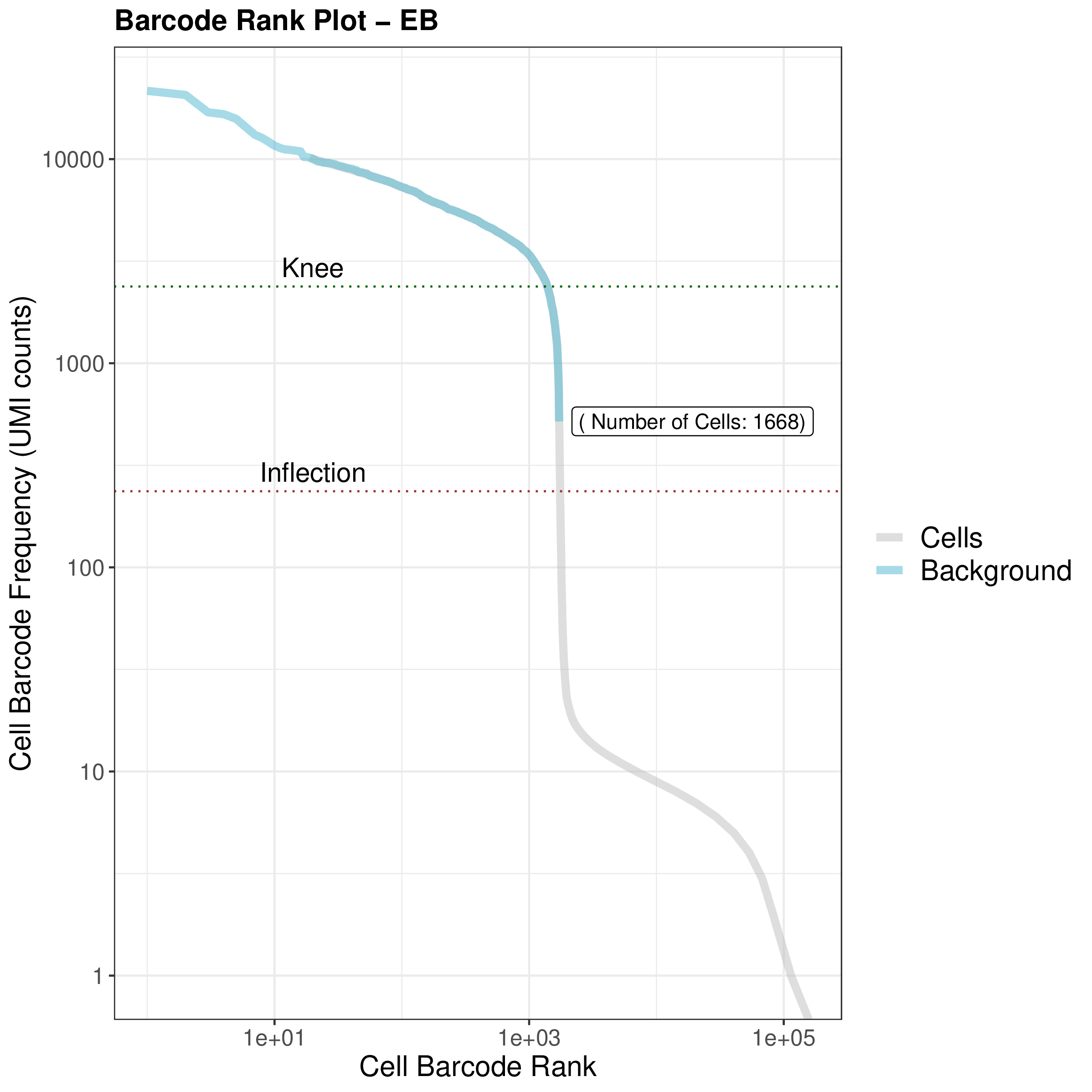

Supplement: Supplementary file 2 — Additional file 2: Supplementary file 2. To demonstrate the utility of scQCEA, we apply the workflow to the sixteen gene expression profiles of eight patients with metastatic melanoma, prepared from pre- and post-treatment experimental batches. You can find the QC interactive report at: https://github.com/isarnassiri/scQCEA/tree/Example-of-Application. Download and unzip the OGC_Interactive_QC_Report_P180121.zip file. You can open CLICK_ME.html file without using rStudio/R. [file 12864_2023_9447_MOESM2_ESM.zip › Inputs/10X-gex/481207_28/P180121-keep_481207_28_BarcodeRankPlot_EB.png]

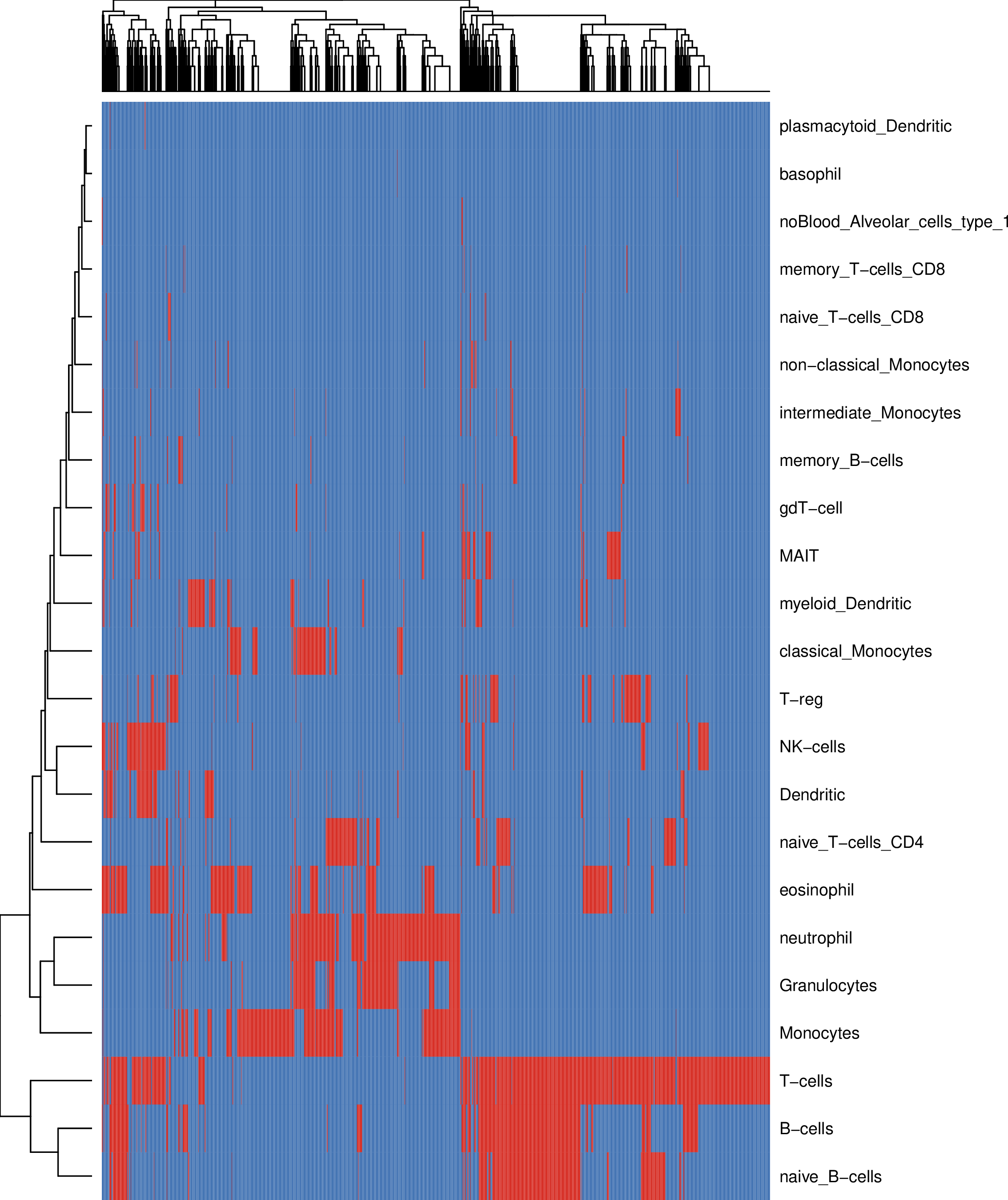

Supplement: Supplementary file 2 — Additional file 2: Supplementary file 2. To demonstrate the utility of scQCEA, we apply the workflow to the sixteen gene expression profiles of eight patients with metastatic melanoma, prepared from pre- and post-treatment experimental batches. You can find the QC interactive report at: https://github.com/isarnassiri/scQCEA/tree/Example-of-Application. Download and unzip the OGC_Interactive_QC_Report_P180121.zip file. You can open CLICK_ME.html file without using rStudio/R. [file 12864_2023_9447_MOESM2_ESM.zip › Inputs/10X-gex/481207_28/P180121-keep_481207_28_Celltype_assignment_HeatMap.png]

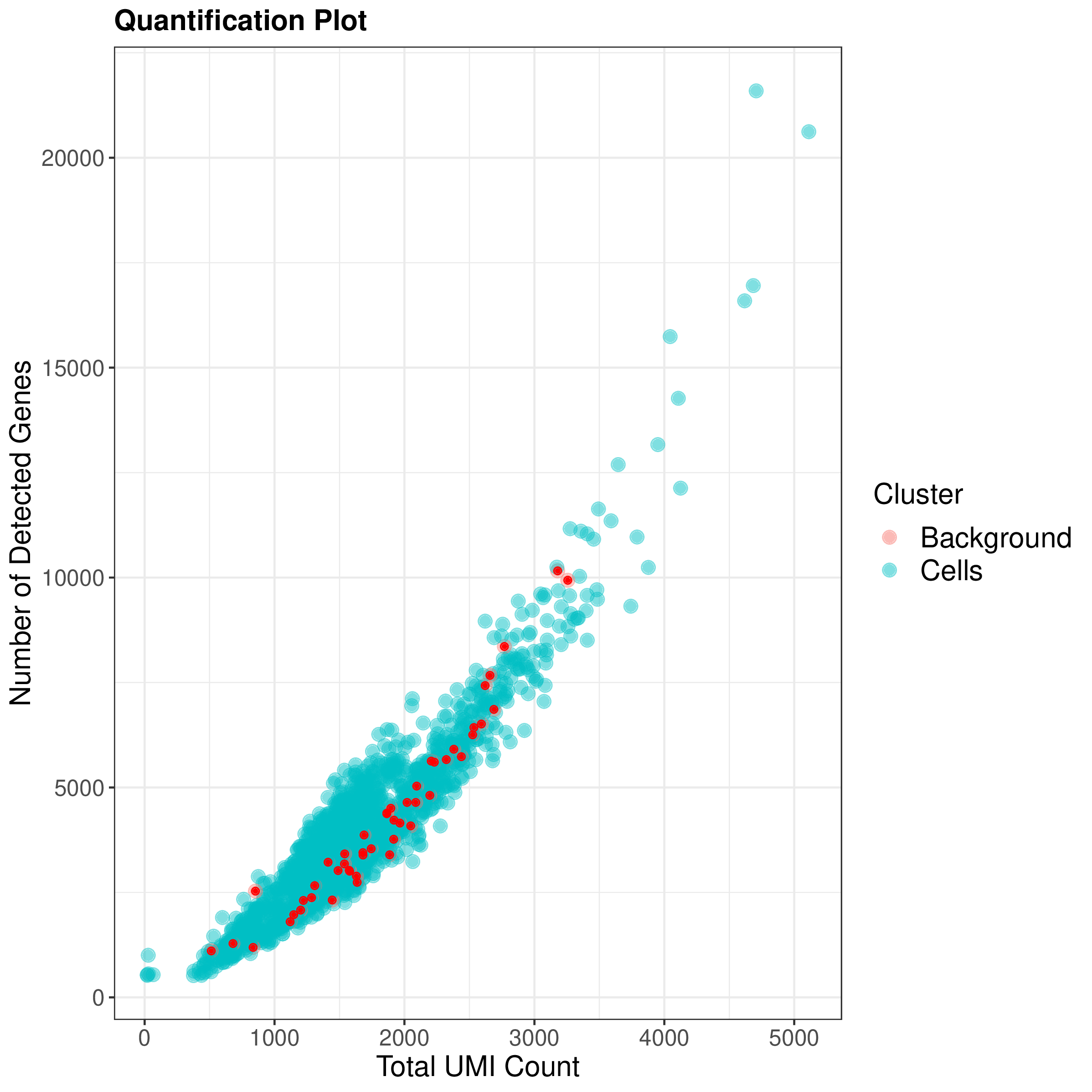

Supplement: Supplementary file 2 — Additional file 2: Supplementary file 2. To demonstrate the utility of scQCEA, we apply the workflow to the sixteen gene expression profiles of eight patients with metastatic melanoma, prepared from pre- and post-treatment experimental batches. You can find the QC interactive report at: https://github.com/isarnassiri/scQCEA/tree/Example-of-Application. Download and unzip the OGC_Interactive_QC_Report_P180121.zip file. You can open CLICK_ME.html file without using rStudio/R. [file 12864_2023_9447_MOESM2_ESM.zip › Inputs/10X-gex/481207_28/P180121-keep_481207_28_TotalUMIvsDetectedGenes.png]

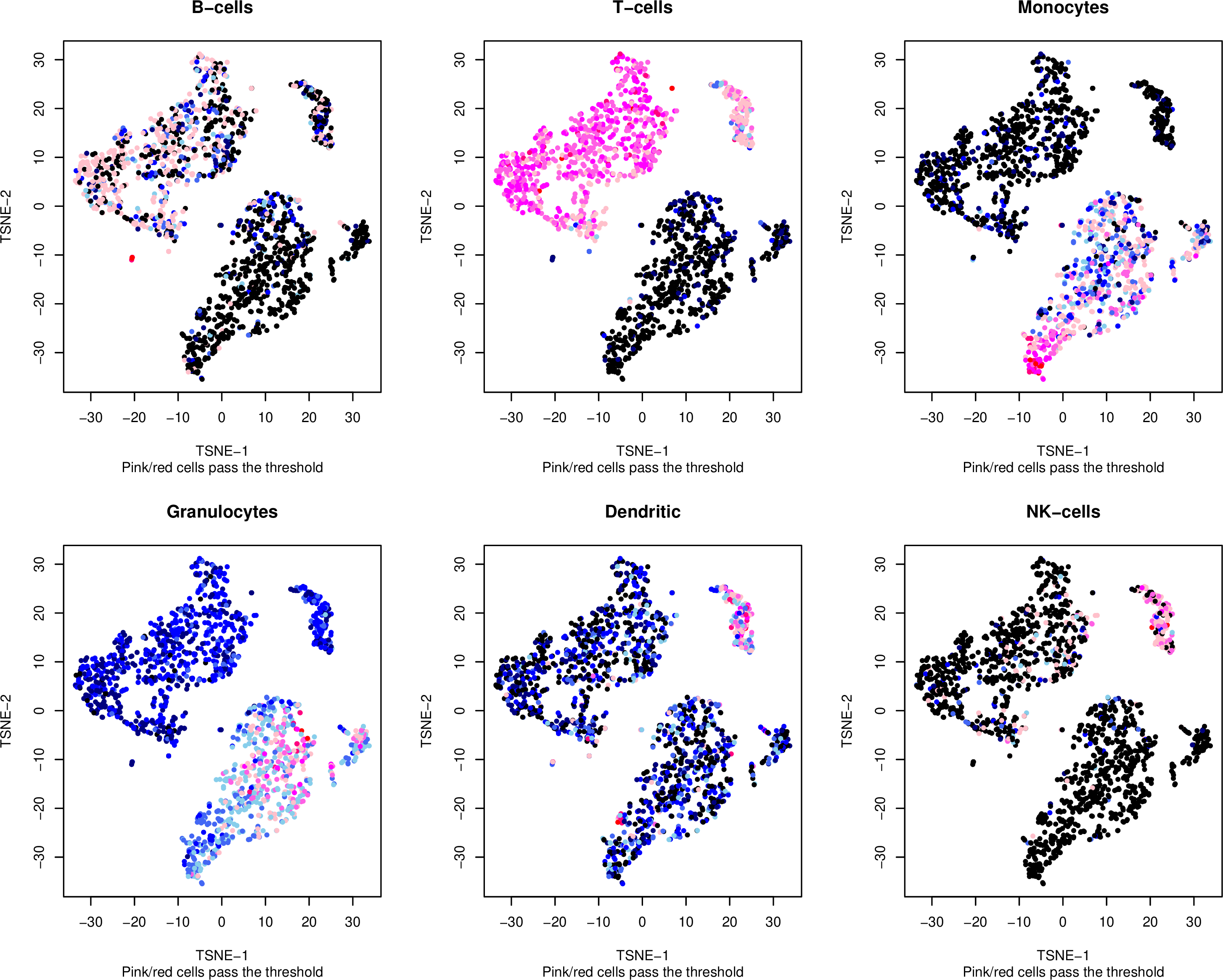

Supplement: Supplementary file 2 — Additional file 2: Supplementary file 2. To demonstrate the utility of scQCEA, we apply the workflow to the sixteen gene expression profiles of eight patients with metastatic melanoma, prepared from pre- and post-treatment experimental batches. You can find the QC interactive report at: https://github.com/isarnassiri/scQCEA/tree/Example-of-Application. Download and unzip the OGC_Interactive_QC_Report_P180121.zip file. You can open CLICK_ME.html file without using rStudio/R. [file 12864_2023_9447_MOESM2_ESM.zip › Inputs/10X-gex/481207_28/P180121-keep_481207_28_tSNE_Plot.png]

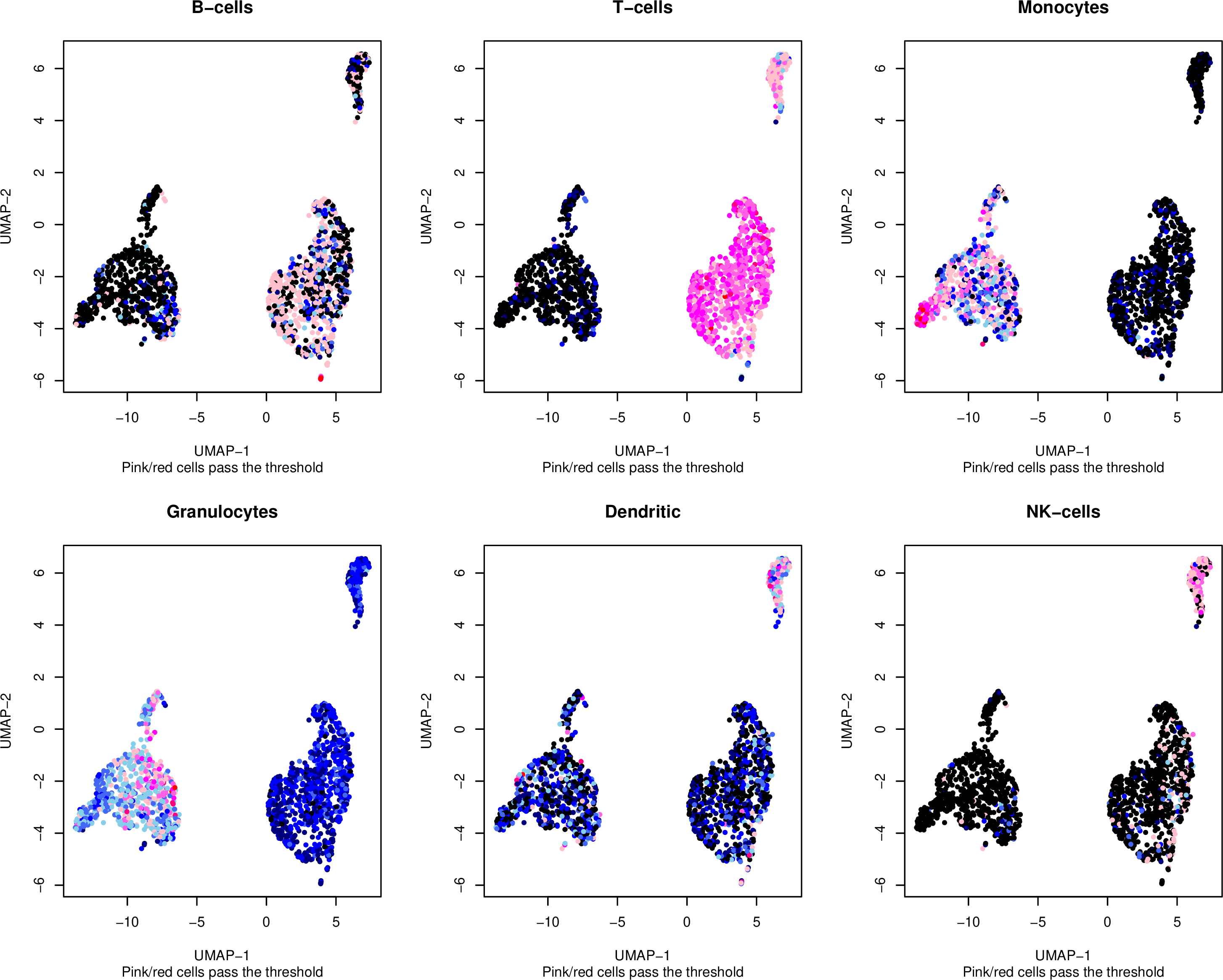

Supplement: Supplementary file 2 — Additional file 2: Supplementary file 2. To demonstrate the utility of scQCEA, we apply the workflow to the sixteen gene expression profiles of eight patients with metastatic melanoma, prepared from pre- and post-treatment experimental batches. You can find the QC interactive report at: https://github.com/isarnassiri/scQCEA/tree/Example-of-Application. Download and unzip the OGC_Interactive_QC_Report_P180121.zip file. You can open CLICK_ME.html file without using rStudio/R. [file 12864_2023_9447_MOESM2_ESM.zip › Inputs/10X-gex/481207_28/P180121-keep_481207_28_UMAP_Plot.png]

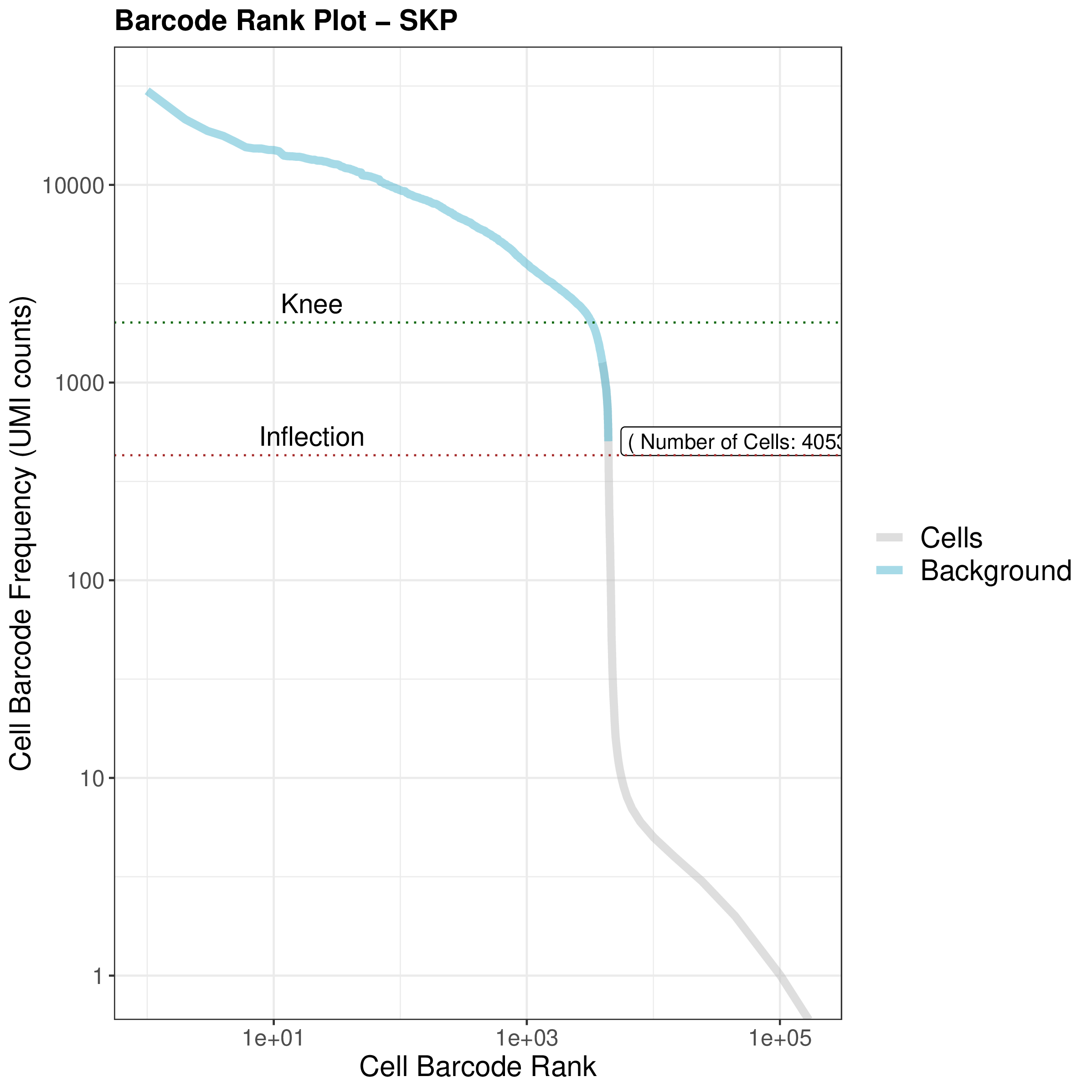

Supplement: Supplementary file 2 — Additional file 2: Supplementary file 2. To demonstrate the utility of scQCEA, we apply the workflow to the sixteen gene expression profiles of eight patients with metastatic melanoma, prepared from pre- and post-treatment experimental batches. You can find the QC interactive report at: https://github.com/isarnassiri/scQCEA/tree/Example-of-Application. Download and unzip the OGC_Interactive_QC_Report_P180121.zip file. You can open CLICK_ME.html file without using rStudio/R. [file 12864_2023_9447_MOESM2_ESM.zip › Inputs/10X-gex/481207_40/P180121-keep_481207_40_BarcodeRankPlot_10X.png]

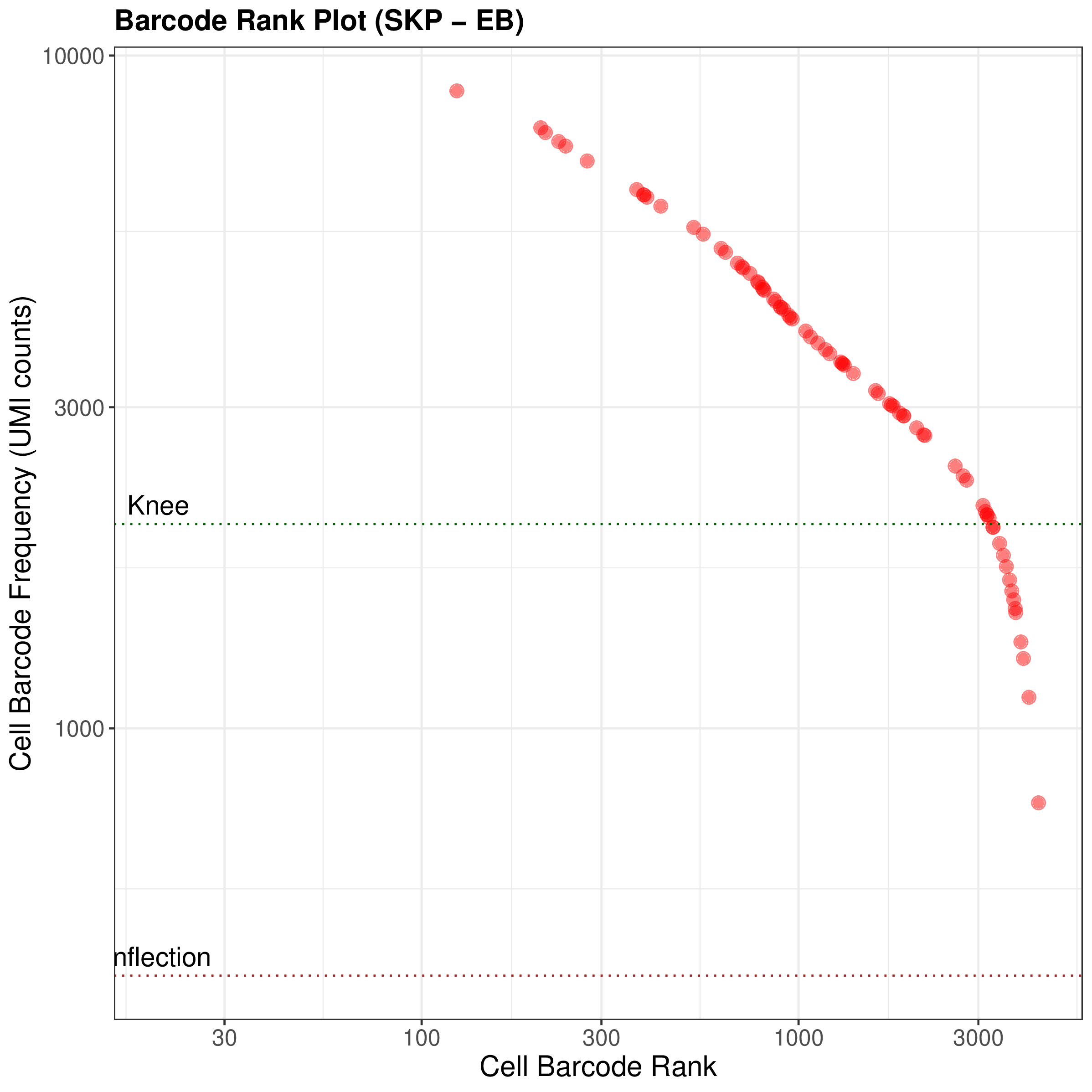

Supplement: Supplementary file 2 — Additional file 2: Supplementary file 2. To demonstrate the utility of scQCEA, we apply the workflow to the sixteen gene expression profiles of eight patients with metastatic melanoma, prepared from pre- and post-treatment experimental batches. You can find the QC interactive report at: https://github.com/isarnassiri/scQCEA/tree/Example-of-Application. Download and unzip the OGC_Interactive_QC_Report_P180121.zip file. You can open CLICK_ME.html file without using rStudio/R. [file 12864_2023_9447_MOESM2_ESM.zip › Inputs/10X-gex/481207_40/P180121-keep_481207_40_BarcodeRankPlot_EB_FilterOut.png]

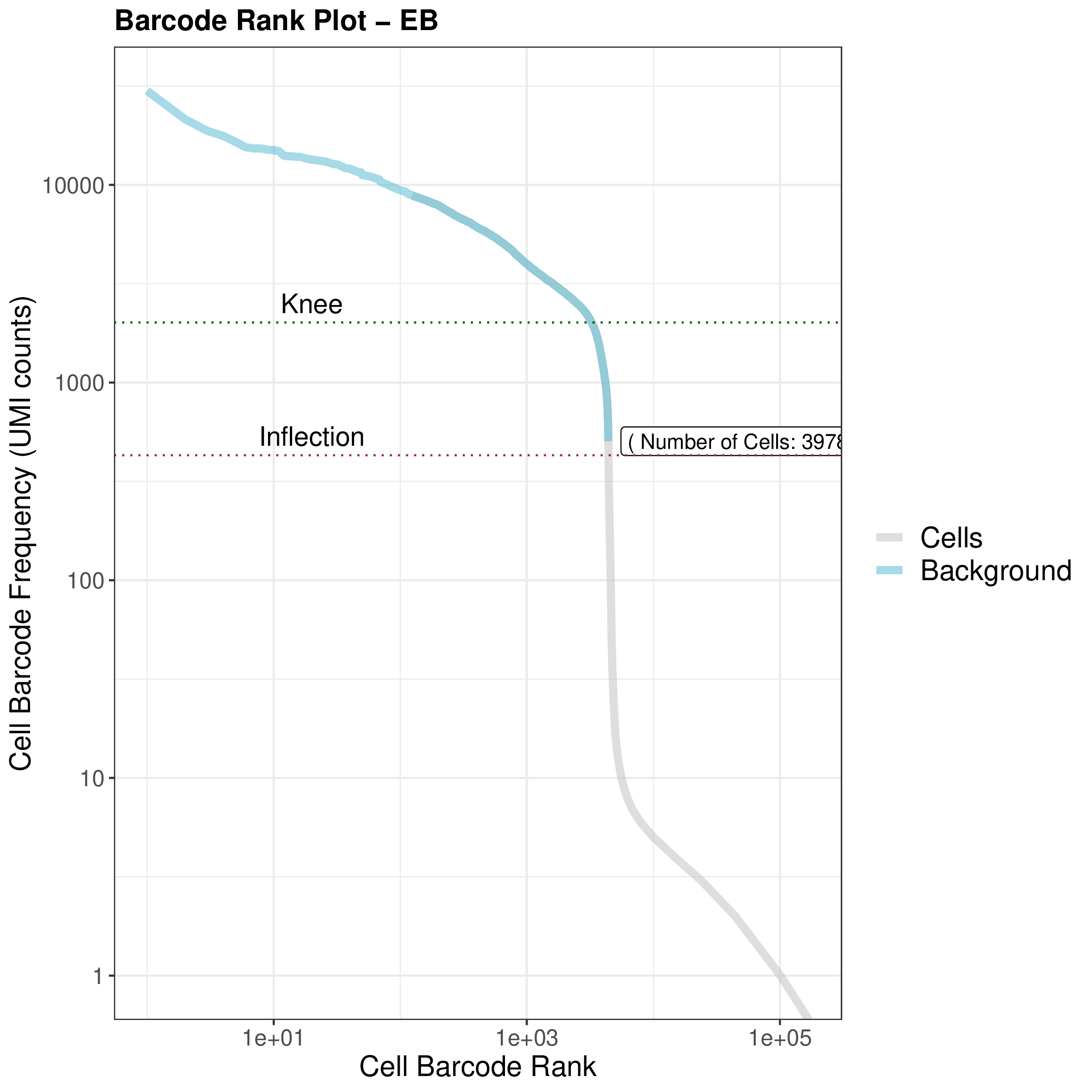

Supplement: Supplementary file 2 — Additional file 2: Supplementary file 2. To demonstrate the utility of scQCEA, we apply the workflow to the sixteen gene expression profiles of eight patients with metastatic melanoma, prepared from pre- and post-treatment experimental batches. You can find the QC interactive report at: https://github.com/isarnassiri/scQCEA/tree/Example-of-Application. Download and unzip the OGC_Interactive_QC_Report_P180121.zip file. You can open CLICK_ME.html file without using rStudio/R. [file 12864_2023_9447_MOESM2_ESM.zip › Inputs/10X-gex/481207_40/P180121-keep_481207_40_BarcodeRankPlot_EB.png]

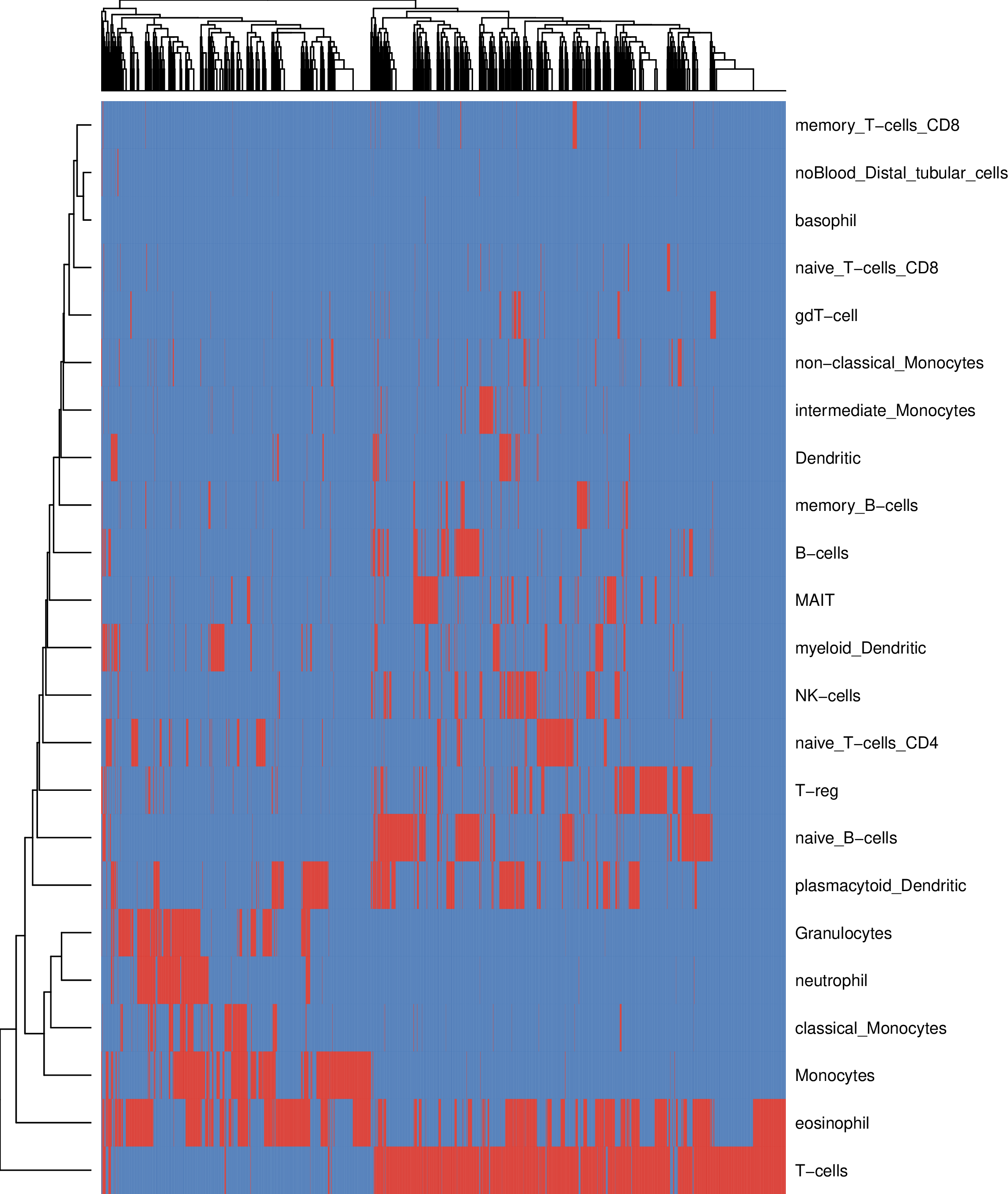

Supplement: Supplementary file 2 — Additional file 2: Supplementary file 2. To demonstrate the utility of scQCEA, we apply the workflow to the sixteen gene expression profiles of eight patients with metastatic melanoma, prepared from pre- and post-treatment experimental batches. You can find the QC interactive report at: https://github.com/isarnassiri/scQCEA/tree/Example-of-Application. Download and unzip the OGC_Interactive_QC_Report_P180121.zip file. You can open CLICK_ME.html file without using rStudio/R. [file 12864_2023_9447_MOESM2_ESM.zip › Inputs/10X-gex/481207_40/P180121-keep_481207_40_Celltype_assignment_HeatMap.png]

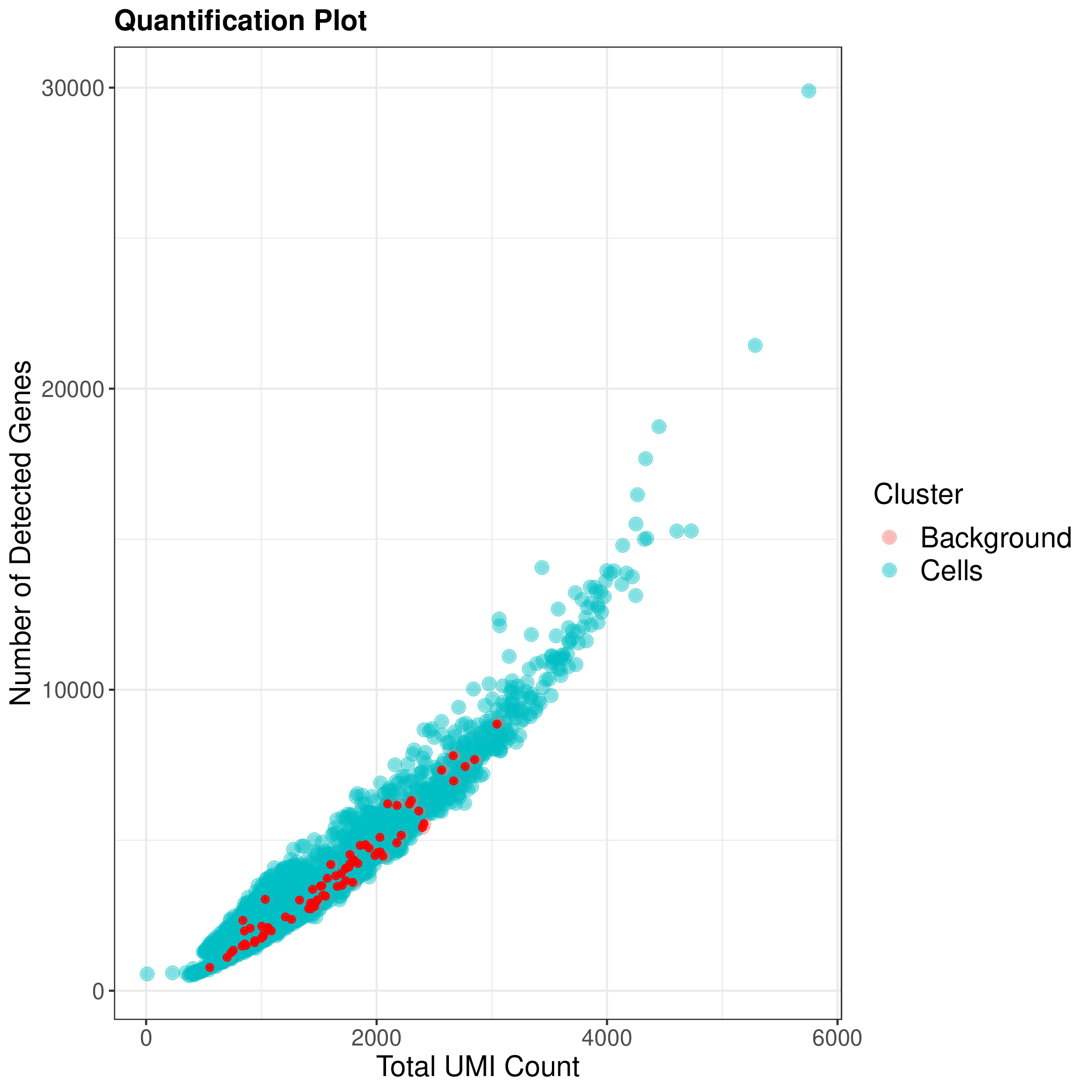

Supplement: Supplementary file 2 — Additional file 2: Supplementary file 2. To demonstrate the utility of scQCEA, we apply the workflow to the sixteen gene expression profiles of eight patients with metastatic melanoma, prepared from pre- and post-treatment experimental batches. You can find the QC interactive report at: https://github.com/isarnassiri/scQCEA/tree/Example-of-Application. Download and unzip the OGC_Interactive_QC_Report_P180121.zip file. You can open CLICK_ME.html file without using rStudio/R. [file 12864_2023_9447_MOESM2_ESM.zip › Inputs/10X-gex/481207_40/P180121-keep_481207_40_TotalUMIvsDetectedGenes.png]

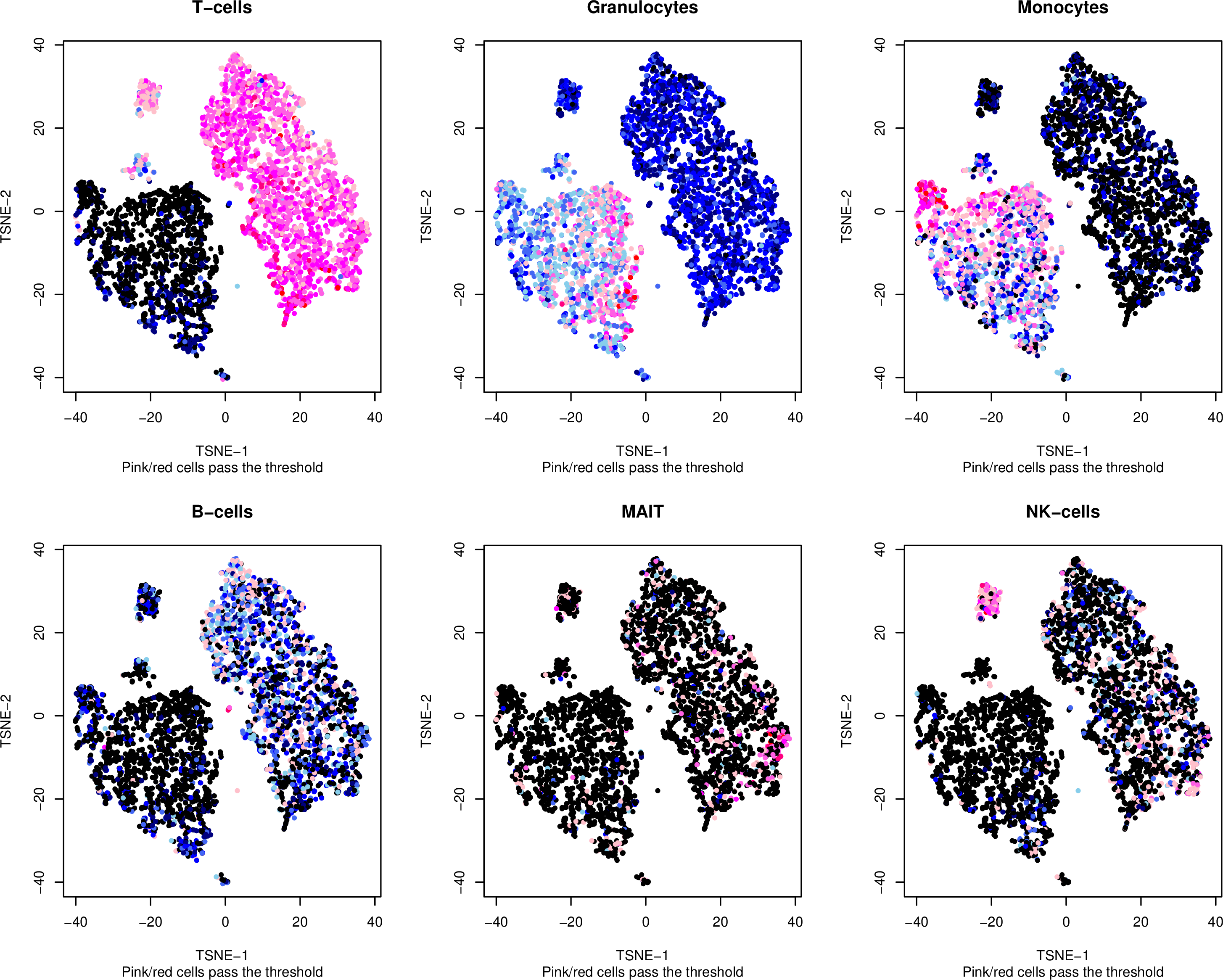

Supplement: Supplementary file 2 — Additional file 2: Supplementary file 2. To demonstrate the utility of scQCEA, we apply the workflow to the sixteen gene expression profiles of eight patients with metastatic melanoma, prepared from pre- and post-treatment experimental batches. You can find the QC interactive report at: https://github.com/isarnassiri/scQCEA/tree/Example-of-Application. Download and unzip the OGC_Interactive_QC_Report_P180121.zip file. You can open CLICK_ME.html file without using rStudio/R. [file 12864_2023_9447_MOESM2_ESM.zip › Inputs/10X-gex/481207_40/P180121-keep_481207_40_tSNE_Plot.png]

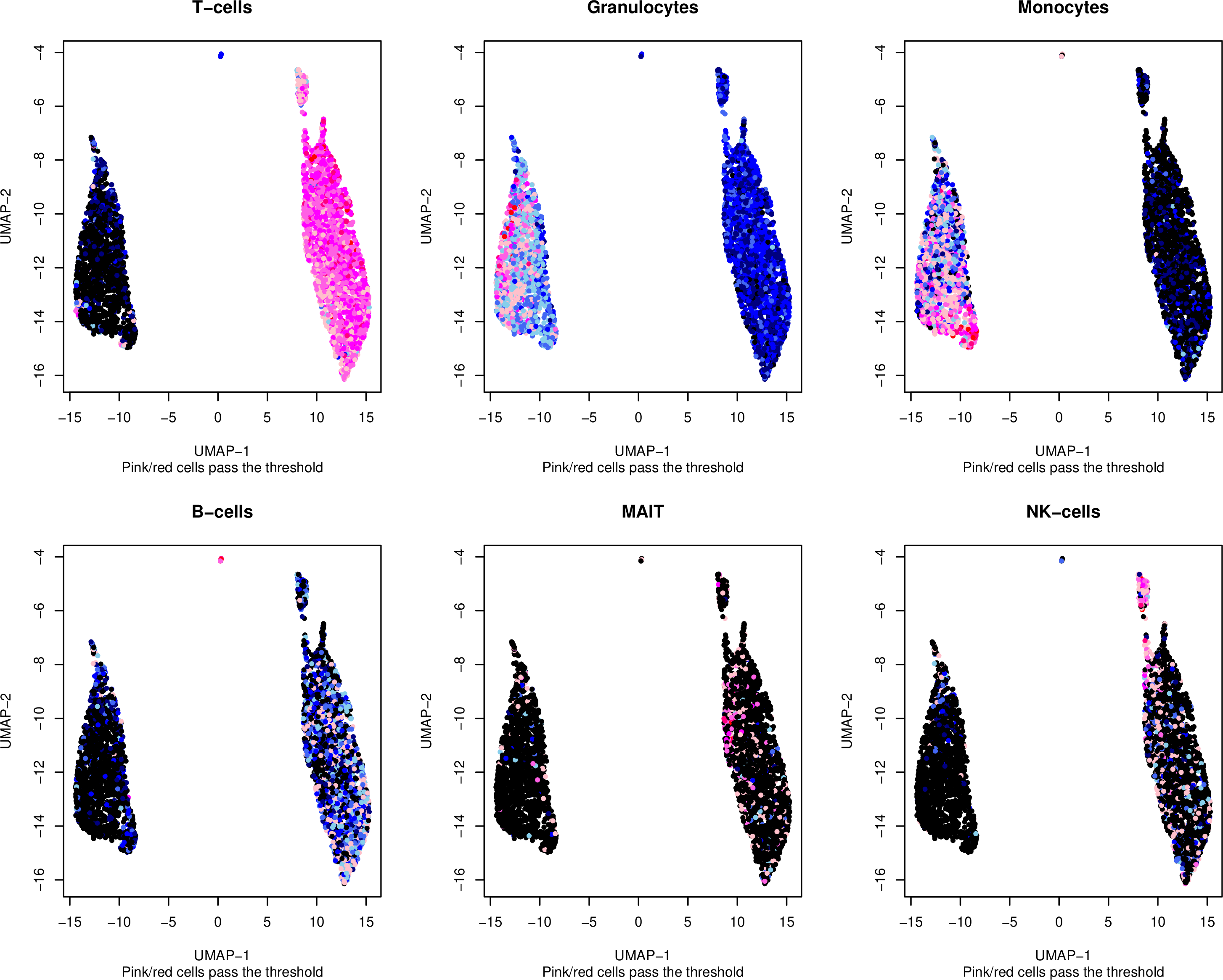

Supplement: Supplementary file 2 — Additional file 2: Supplementary file 2. To demonstrate the utility of scQCEA, we apply the workflow to the sixteen gene expression profiles of eight patients with metastatic melanoma, prepared from pre- and post-treatment experimental batches. You can find the QC interactive report at: https://github.com/isarnassiri/scQCEA/tree/Example-of-Application. Download and unzip the OGC_Interactive_QC_Report_P180121.zip file. You can open CLICK_ME.html file without using rStudio/R. [file 12864_2023_9447_MOESM2_ESM.zip › Inputs/10X-gex/481207_40/P180121-keep_481207_40_UMAP_Plot.png]

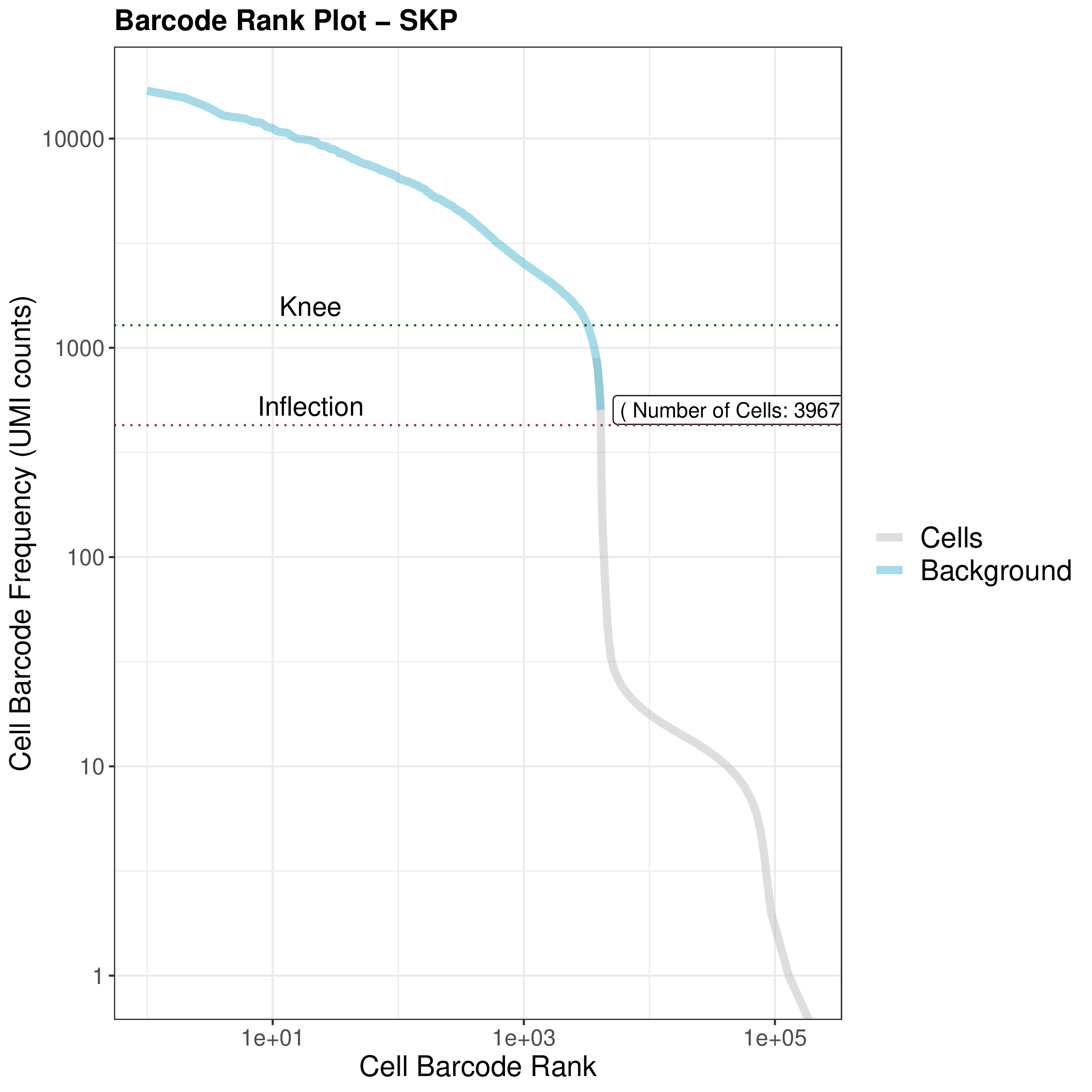

Supplement: Supplementary file 2 — Additional file 2: Supplementary file 2. To demonstrate the utility of scQCEA, we apply the workflow to the sixteen gene expression profiles of eight patients with metastatic melanoma, prepared from pre- and post-treatment experimental batches. You can find the QC interactive report at: https://github.com/isarnassiri/scQCEA/tree/Example-of-Application. Download and unzip the OGC_Interactive_QC_Report_P180121.zip file. You can open CLICK_ME.html file without using rStudio/R. [file 12864_2023_9447_MOESM2_ESM.zip › Inputs/10X-gex/481207_52/P180121-keep_481207_52_BarcodeRankPlot_10X.png]

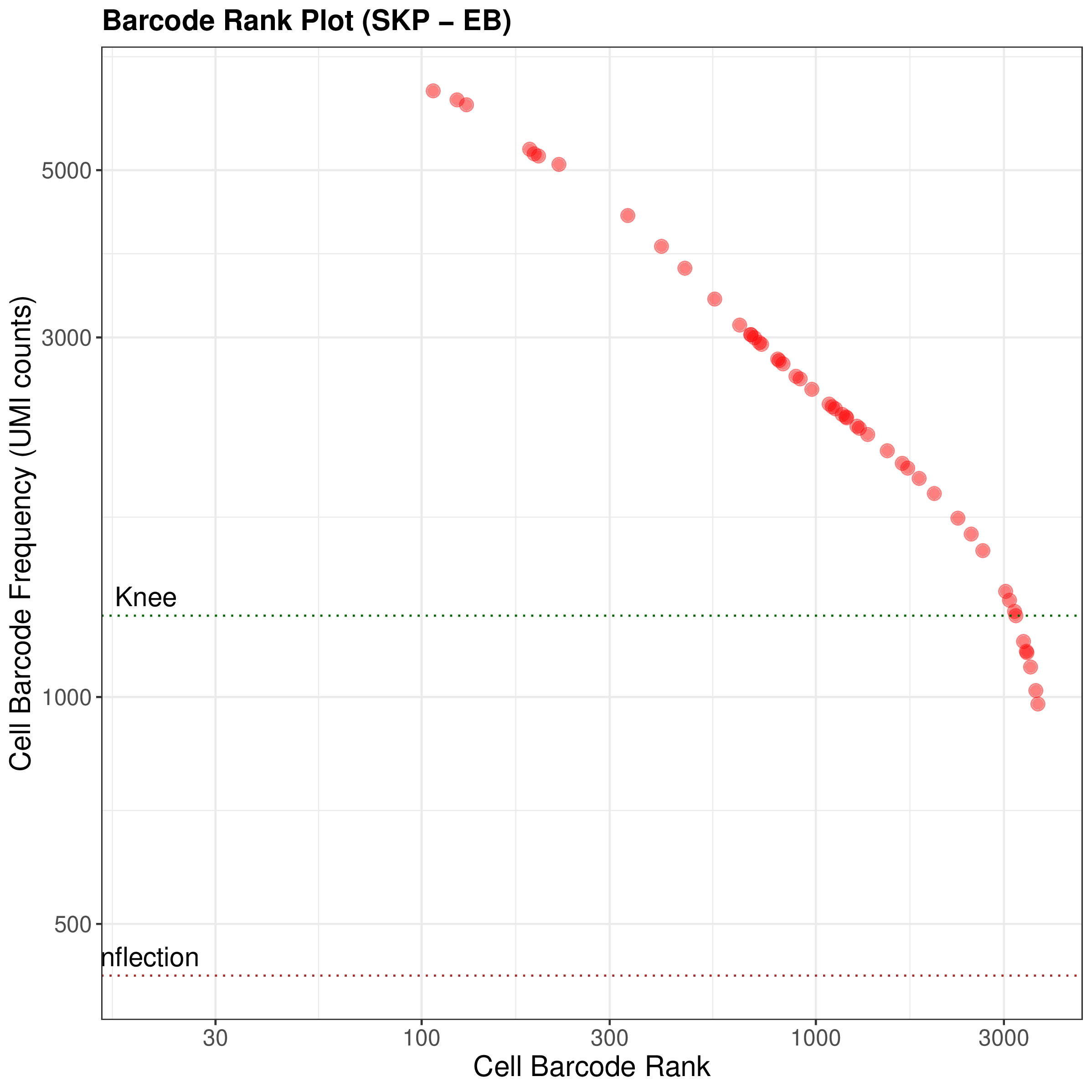

Supplement: Supplementary file 2 — Additional file 2: Supplementary file 2. To demonstrate the utility of scQCEA, we apply the workflow to the sixteen gene expression profiles of eight patients with metastatic melanoma, prepared from pre- and post-treatment experimental batches. You can find the QC interactive report at: https://github.com/isarnassiri/scQCEA/tree/Example-of-Application. Download and unzip the OGC_Interactive_QC_Report_P180121.zip file. You can open CLICK_ME.html file without using rStudio/R. [file 12864_2023_9447_MOESM2_ESM.zip › Inputs/10X-gex/481207_52/P180121-keep_481207_52_BarcodeRankPlot_EB_FilterOut.png]

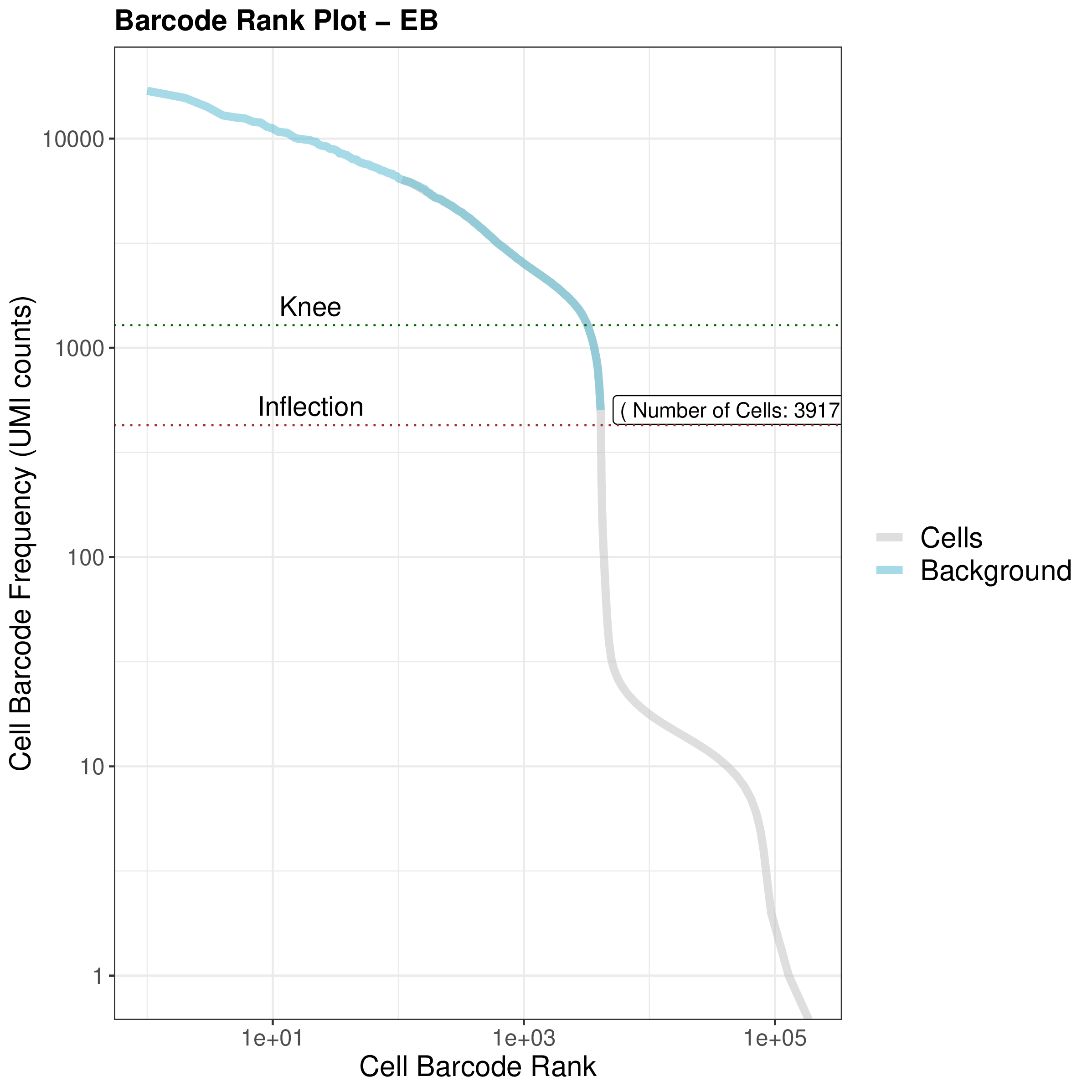

Supplement: Supplementary file 2 — Additional file 2: Supplementary file 2. To demonstrate the utility of scQCEA, we apply the workflow to the sixteen gene expression profiles of eight patients with metastatic melanoma, prepared from pre- and post-treatment experimental batches. You can find the QC interactive report at: https://github.com/isarnassiri/scQCEA/tree/Example-of-Application. Download and unzip the OGC_Interactive_QC_Report_P180121.zip file. You can open CLICK_ME.html file without using rStudio/R. [file 12864_2023_9447_MOESM2_ESM.zip › Inputs/10X-gex/481207_52/P180121-keep_481207_52_BarcodeRankPlot_EB.png]

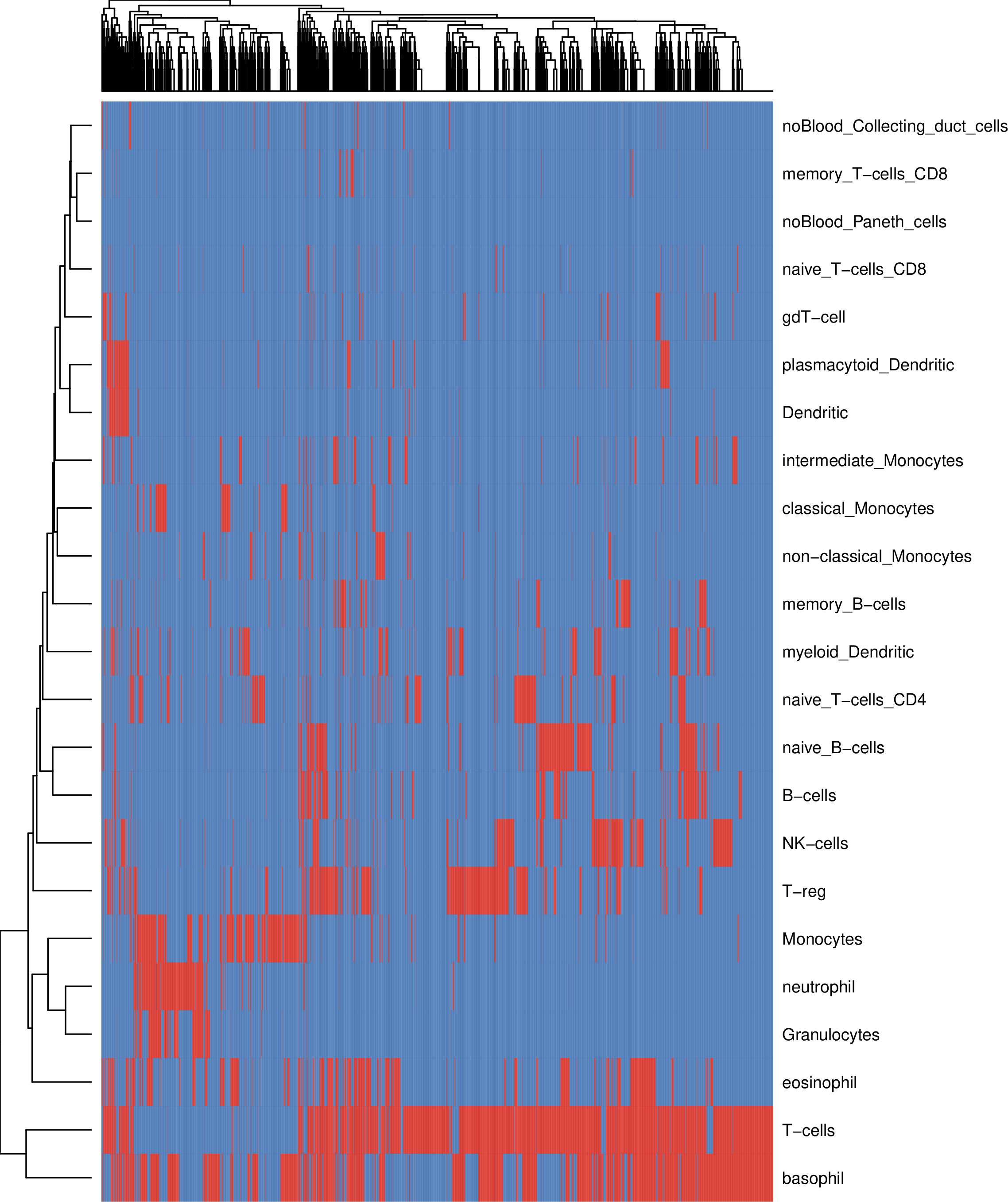

Supplement: Supplementary file 2 — Additional file 2: Supplementary file 2. To demonstrate the utility of scQCEA, we apply the workflow to the sixteen gene expression profiles of eight patients with metastatic melanoma, prepared from pre- and post-treatment experimental batches. You can find the QC interactive report at: https://github.com/isarnassiri/scQCEA/tree/Example-of-Application. Download and unzip the OGC_Interactive_QC_Report_P180121.zip file. You can open CLICK_ME.html file without using rStudio/R. [file 12864_2023_9447_MOESM2_ESM.zip › Inputs/10X-gex/481207_52/P180121-keep_481207_52_Celltype_assignment_HeatMap.png]

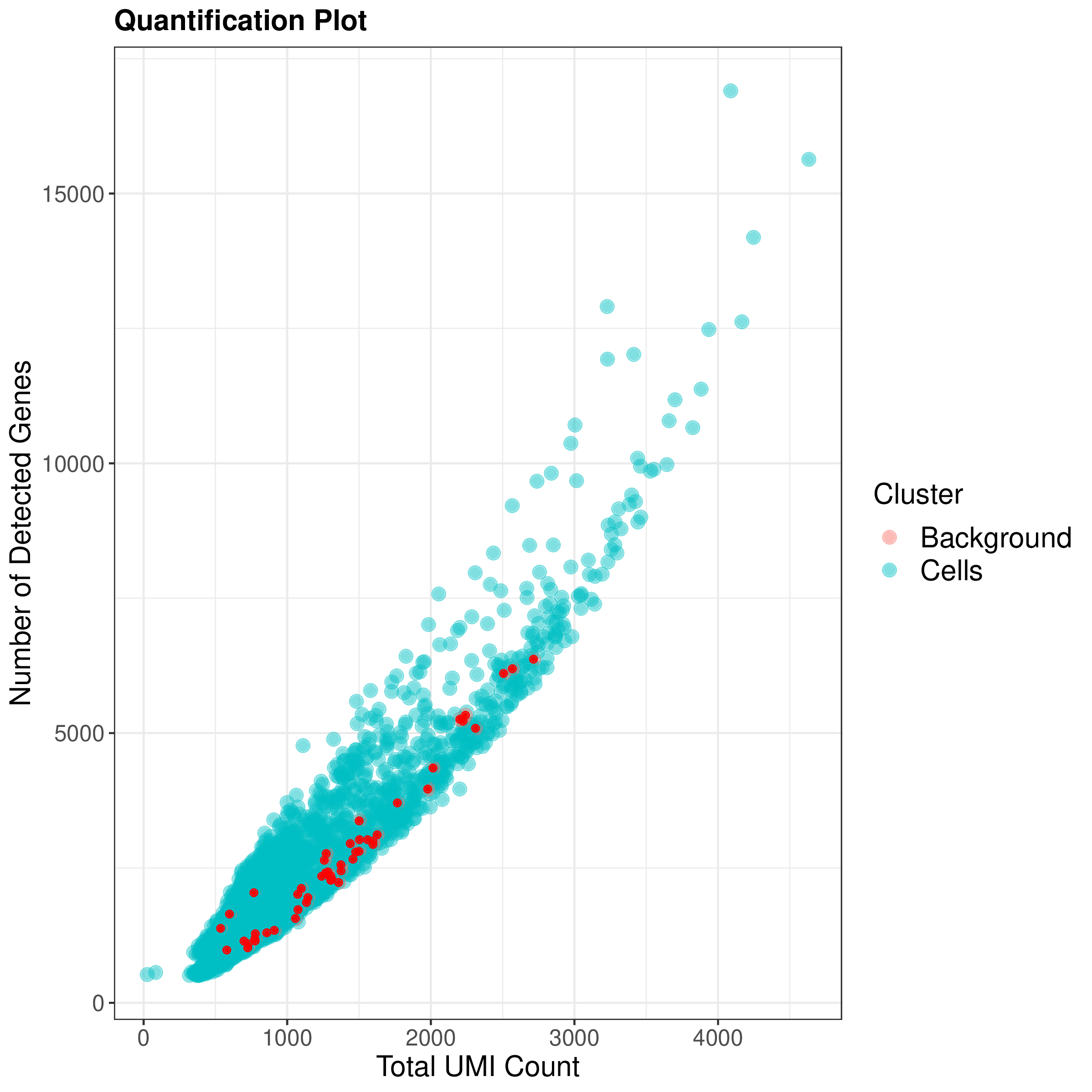

Supplement: Supplementary file 2 — Additional file 2: Supplementary file 2. To demonstrate the utility of scQCEA, we apply the workflow to the sixteen gene expression profiles of eight patients with metastatic melanoma, prepared from pre- and post-treatment experimental batches. You can find the QC interactive report at: https://github.com/isarnassiri/scQCEA/tree/Example-of-Application. Download and unzip the OGC_Interactive_QC_Report_P180121.zip file. You can open CLICK_ME.html file without using rStudio/R. [file 12864_2023_9447_MOESM2_ESM.zip › Inputs/10X-gex/481207_52/P180121-keep_481207_52_TotalUMIvsDetectedGenes.png]

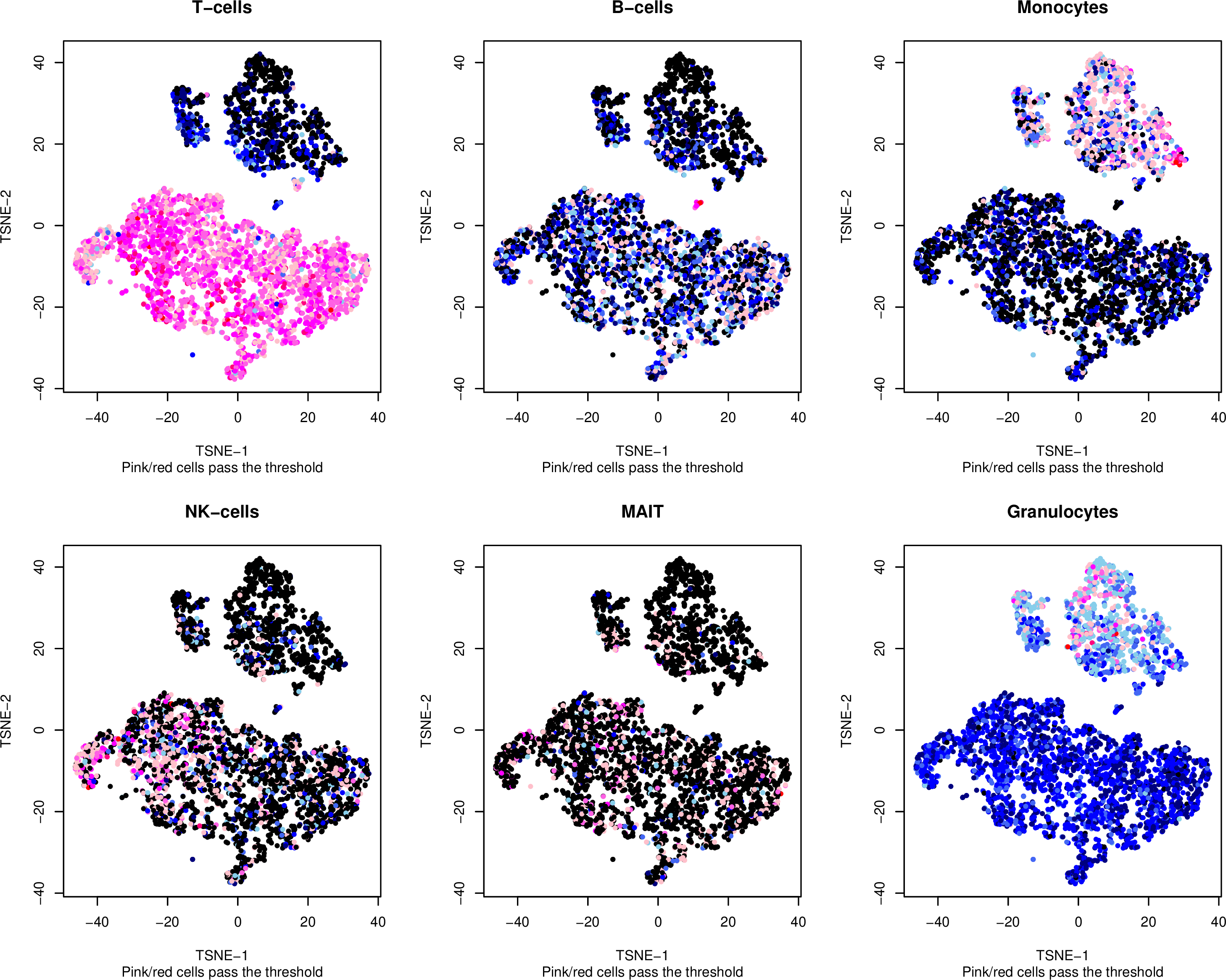

Supplement: Supplementary file 2 — Additional file 2: Supplementary file 2. To demonstrate the utility of scQCEA, we apply the workflow to the sixteen gene expression profiles of eight patients with metastatic melanoma, prepared from pre- and post-treatment experimental batches. You can find the QC interactive report at: https://github.com/isarnassiri/scQCEA/tree/Example-of-Application. Download and unzip the OGC_Interactive_QC_Report_P180121.zip file. You can open CLICK_ME.html file without using rStudio/R. [file 12864_2023_9447_MOESM2_ESM.zip › Inputs/10X-gex/481207_52/P180121-keep_481207_52_tSNE_Plot.png]

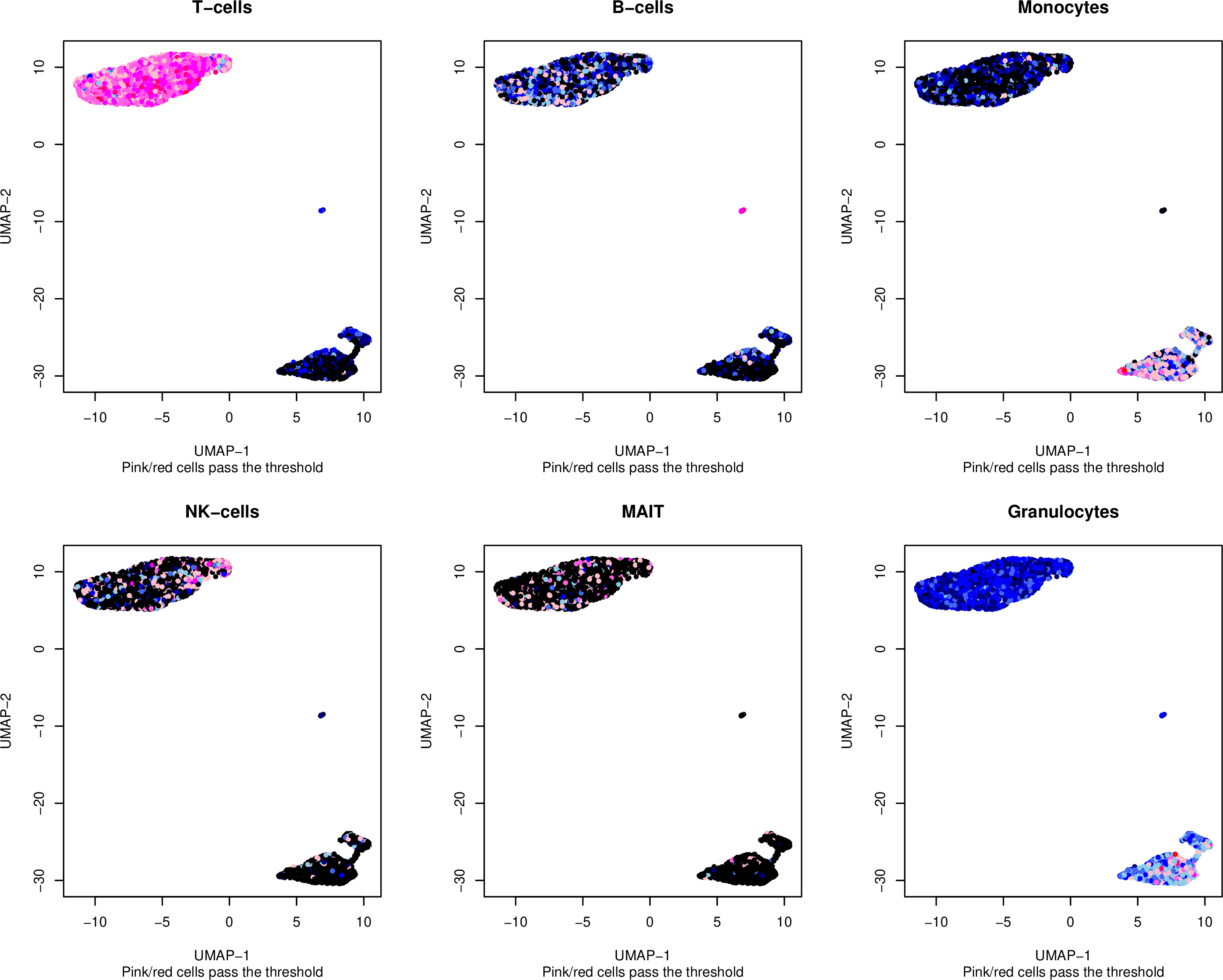

Supplement: Supplementary file 2 — Additional file 2: Supplementary file 2. To demonstrate the utility of scQCEA, we apply the workflow to the sixteen gene expression profiles of eight patients with metastatic melanoma, prepared from pre- and post-treatment experimental batches. You can find the QC interactive report at: https://github.com/isarnassiri/scQCEA/tree/Example-of-Application. Download and unzip the OGC_Interactive_QC_Report_P180121.zip file. You can open CLICK_ME.html file without using rStudio/R. [file 12864_2023_9447_MOESM2_ESM.zip › Inputs/10X-gex/481207_52/P180121-keep_481207_52_UMAP_Plot.png]

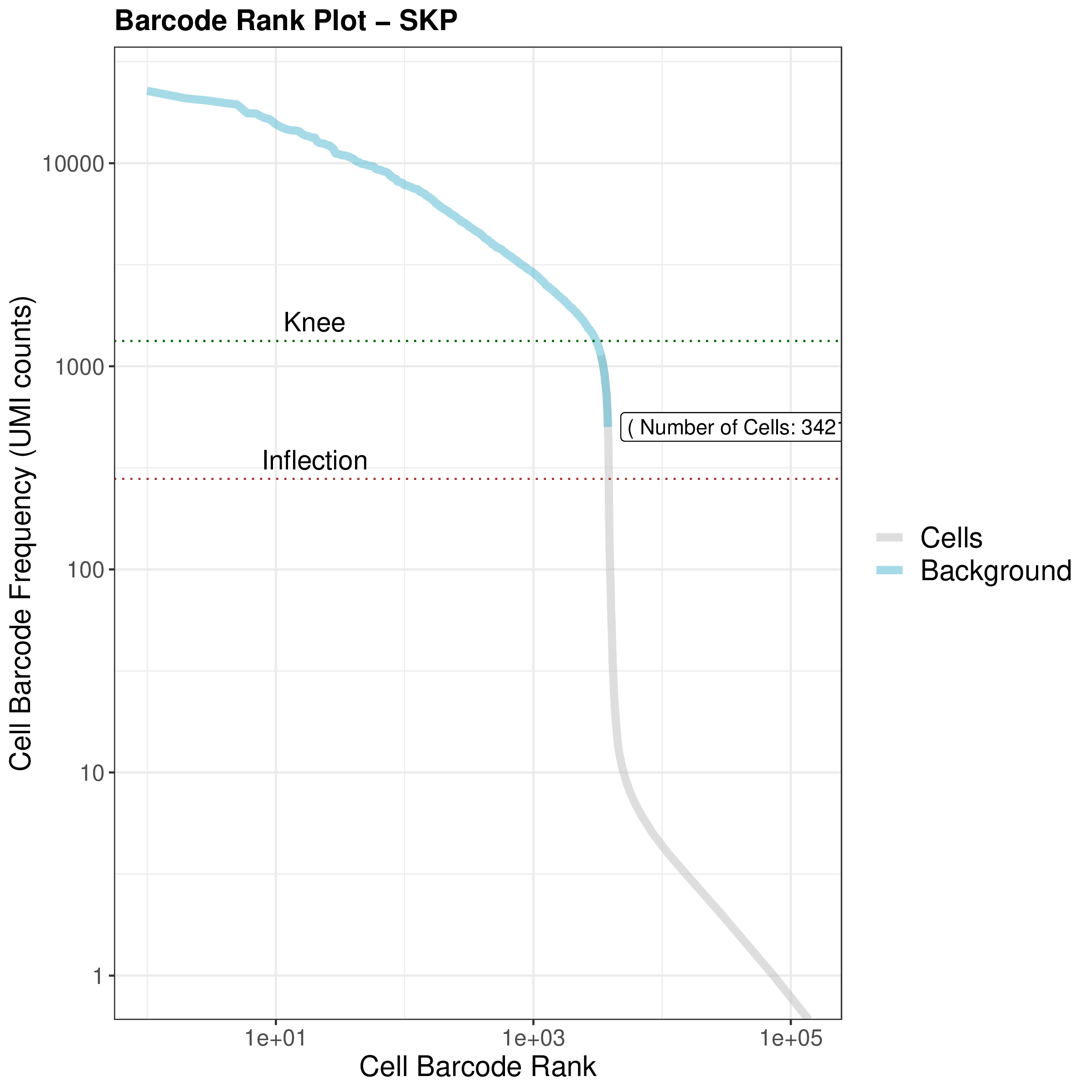

Supplement: Supplementary file 2 — Additional file 2: Supplementary file 2. To demonstrate the utility of scQCEA, we apply the workflow to the sixteen gene expression profiles of eight patients with metastatic melanoma, prepared from pre- and post-treatment experimental batches. You can find the QC interactive report at: https://github.com/isarnassiri/scQCEA/tree/Example-of-Application. Download and unzip the OGC_Interactive_QC_Report_P180121.zip file. You can open CLICK_ME.html file without using rStudio/R. [file 12864_2023_9447_MOESM2_ESM.zip › Inputs/10X-gex/481207_64/P180121-keep_481207_64_BarcodeRankPlot_10X.png]

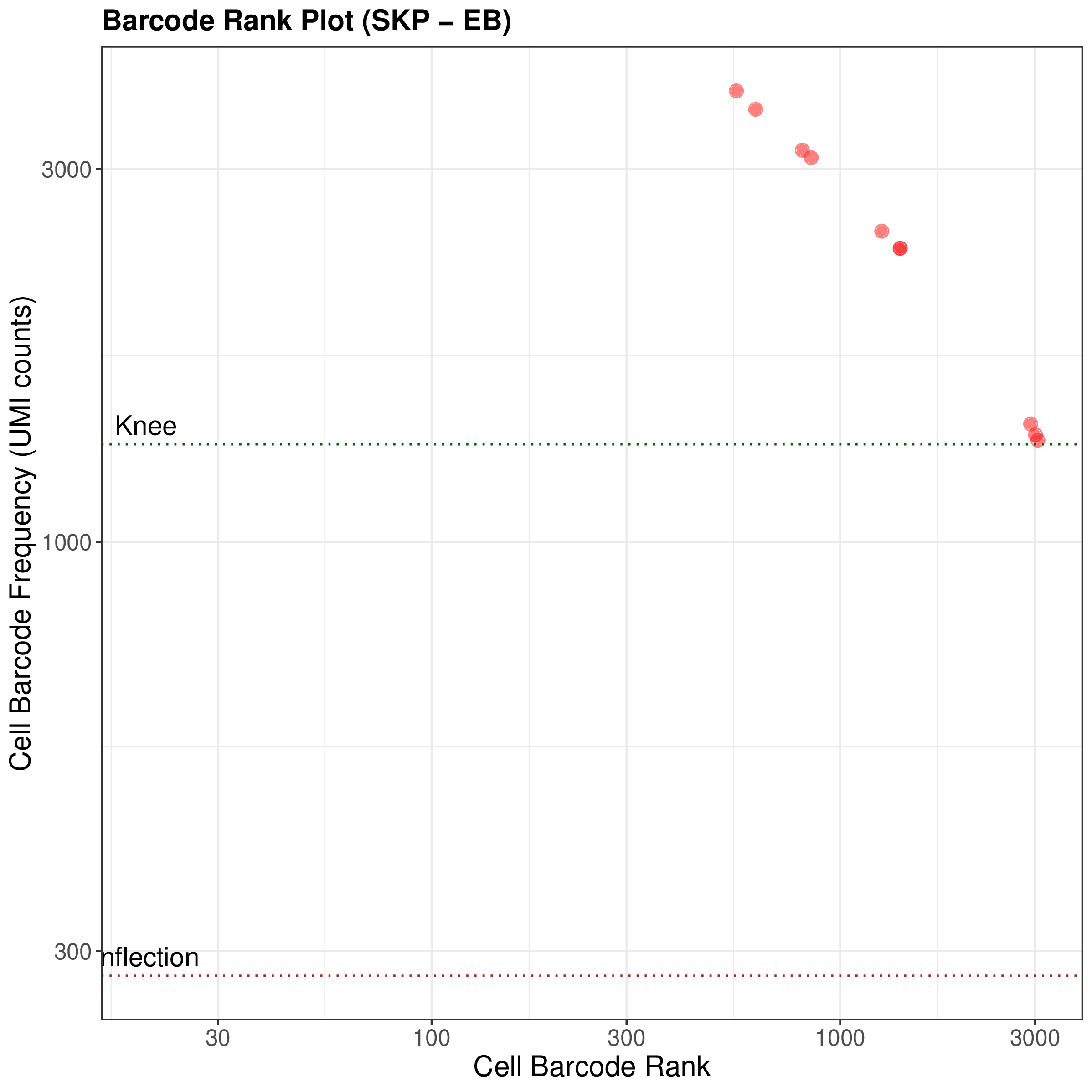

Supplement: Supplementary file 2 — Additional file 2: Supplementary file 2. To demonstrate the utility of scQCEA, we apply the workflow to the sixteen gene expression profiles of eight patients with metastatic melanoma, prepared from pre- and post-treatment experimental batches. You can find the QC interactive report at: https://github.com/isarnassiri/scQCEA/tree/Example-of-Application. Download and unzip the OGC_Interactive_QC_Report_P180121.zip file. You can open CLICK_ME.html file without using rStudio/R. [file 12864_2023_9447_MOESM2_ESM.zip › Inputs/10X-gex/481207_64/P180121-keep_481207_64_BarcodeRankPlot_EB_FilterOut.png]

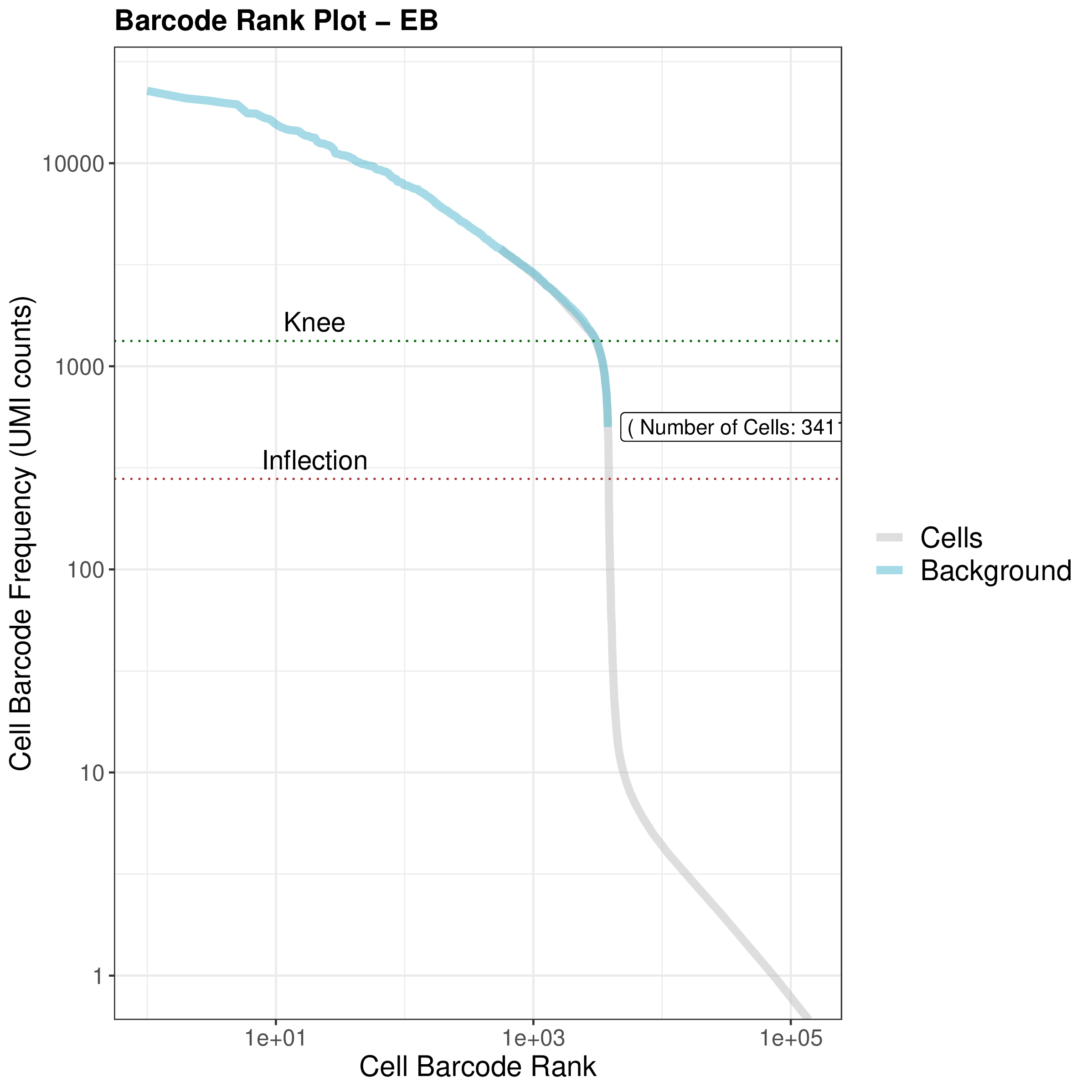

Supplement: Supplementary file 2 — Additional file 2: Supplementary file 2. To demonstrate the utility of scQCEA, we apply the workflow to the sixteen gene expression profiles of eight patients with metastatic melanoma, prepared from pre- and post-treatment experimental batches. You can find the QC interactive report at: https://github.com/isarnassiri/scQCEA/tree/Example-of-Application. Download and unzip the OGC_Interactive_QC_Report_P180121.zip file. You can open CLICK_ME.html file without using rStudio/R. [file 12864_2023_9447_MOESM2_ESM.zip › Inputs/10X-gex/481207_64/P180121-keep_481207_64_BarcodeRankPlot_EB.png]

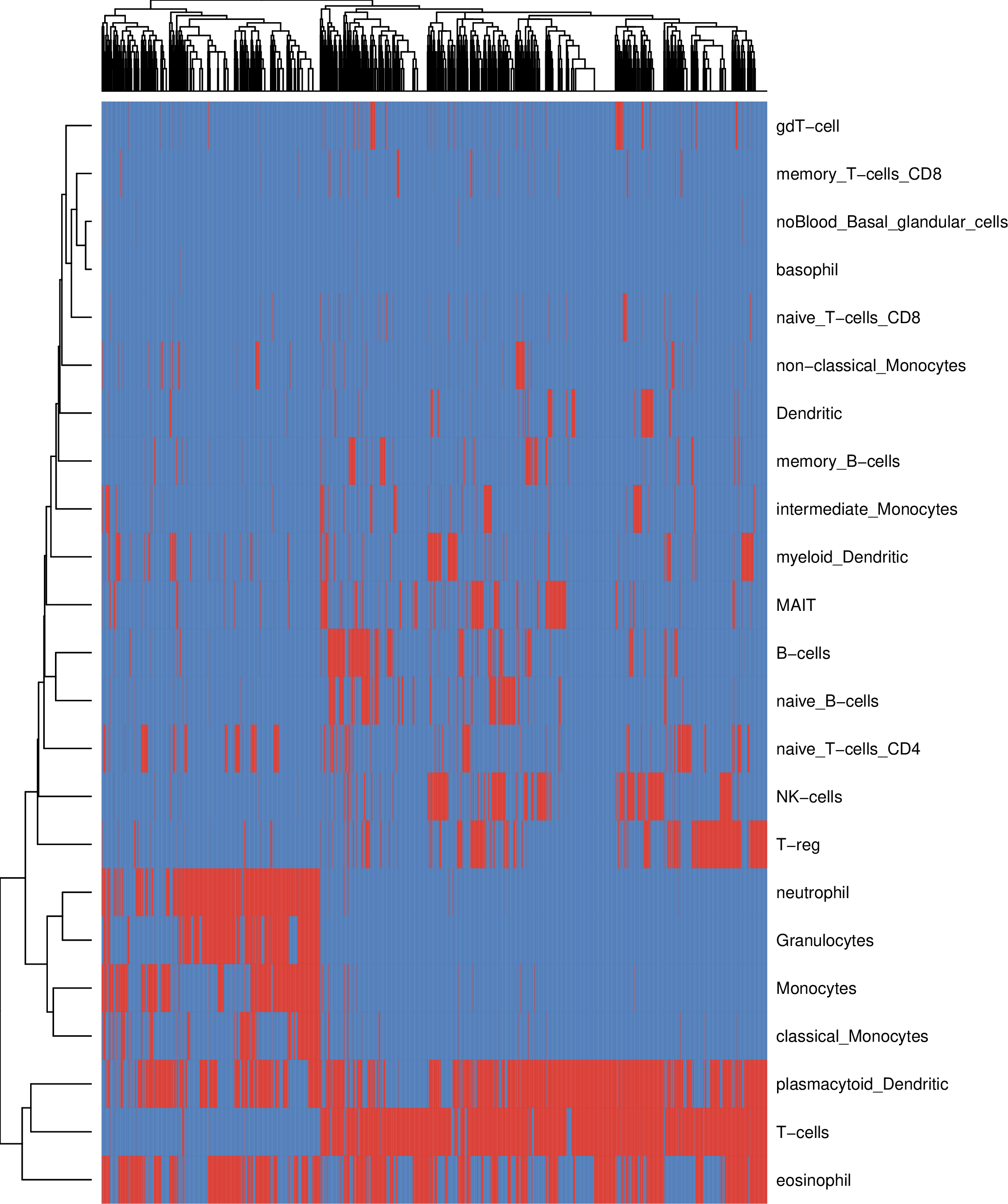

Supplement: Supplementary file 2 — Additional file 2: Supplementary file 2. To demonstrate the utility of scQCEA, we apply the workflow to the sixteen gene expression profiles of eight patients with metastatic melanoma, prepared from pre- and post-treatment experimental batches. You can find the QC interactive report at: https://github.com/isarnassiri/scQCEA/tree/Example-of-Application. Download and unzip the OGC_Interactive_QC_Report_P180121.zip file. You can open CLICK_ME.html file without using rStudio/R. [file 12864_2023_9447_MOESM2_ESM.zip › Inputs/10X-gex/481207_64/P180121-keep_481207_64_Celltype_assignment_HeatMap.png]

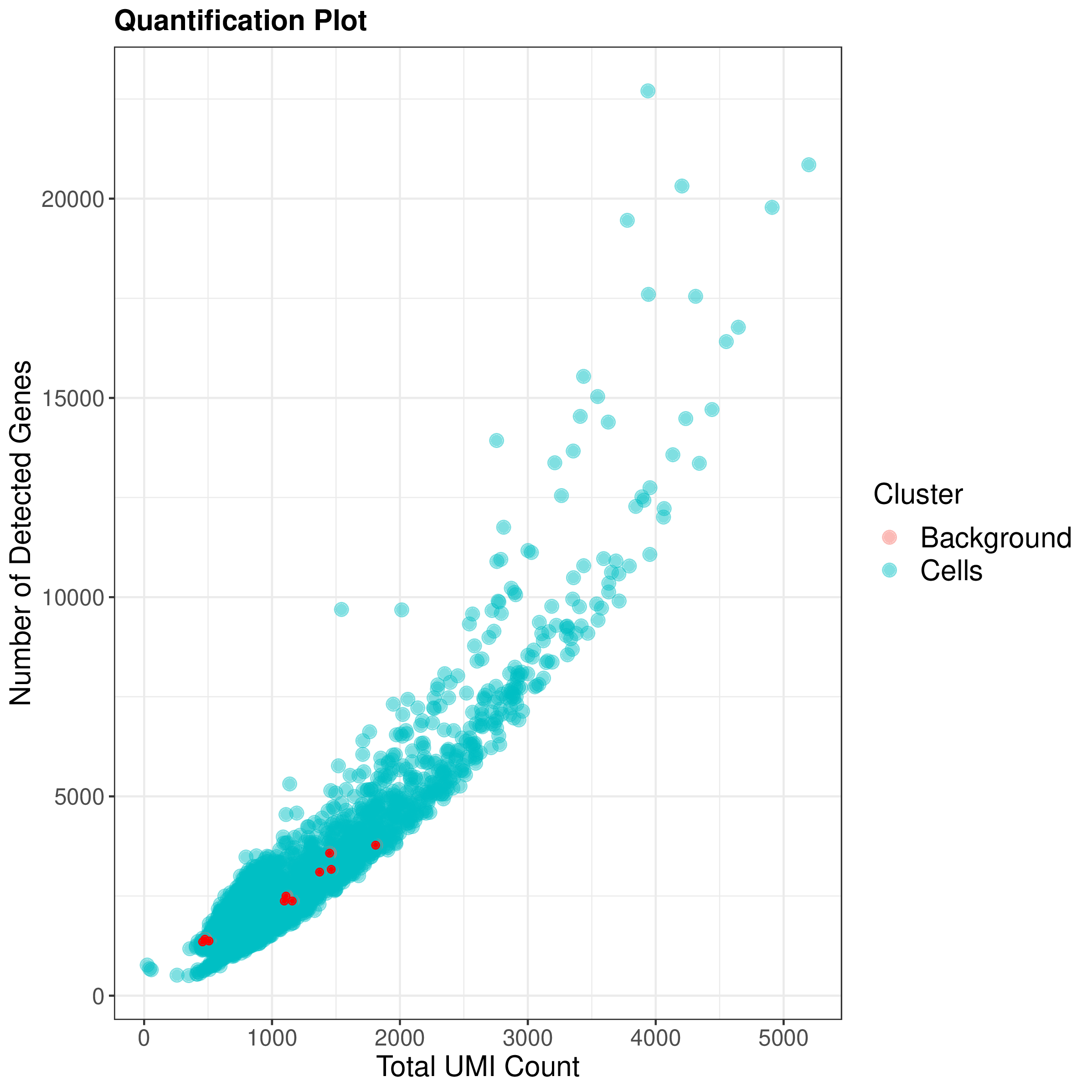

Supplement: Supplementary file 2 — Additional file 2: Supplementary file 2. To demonstrate the utility of scQCEA, we apply the workflow to the sixteen gene expression profiles of eight patients with metastatic melanoma, prepared from pre- and post-treatment experimental batches. You can find the QC interactive report at: https://github.com/isarnassiri/scQCEA/tree/Example-of-Application. Download and unzip the OGC_Interactive_QC_Report_P180121.zip file. You can open CLICK_ME.html file without using rStudio/R. [file 12864_2023_9447_MOESM2_ESM.zip › Inputs/10X-gex/481207_64/P180121-keep_481207_64_TotalUMIvsDetectedGenes.png]

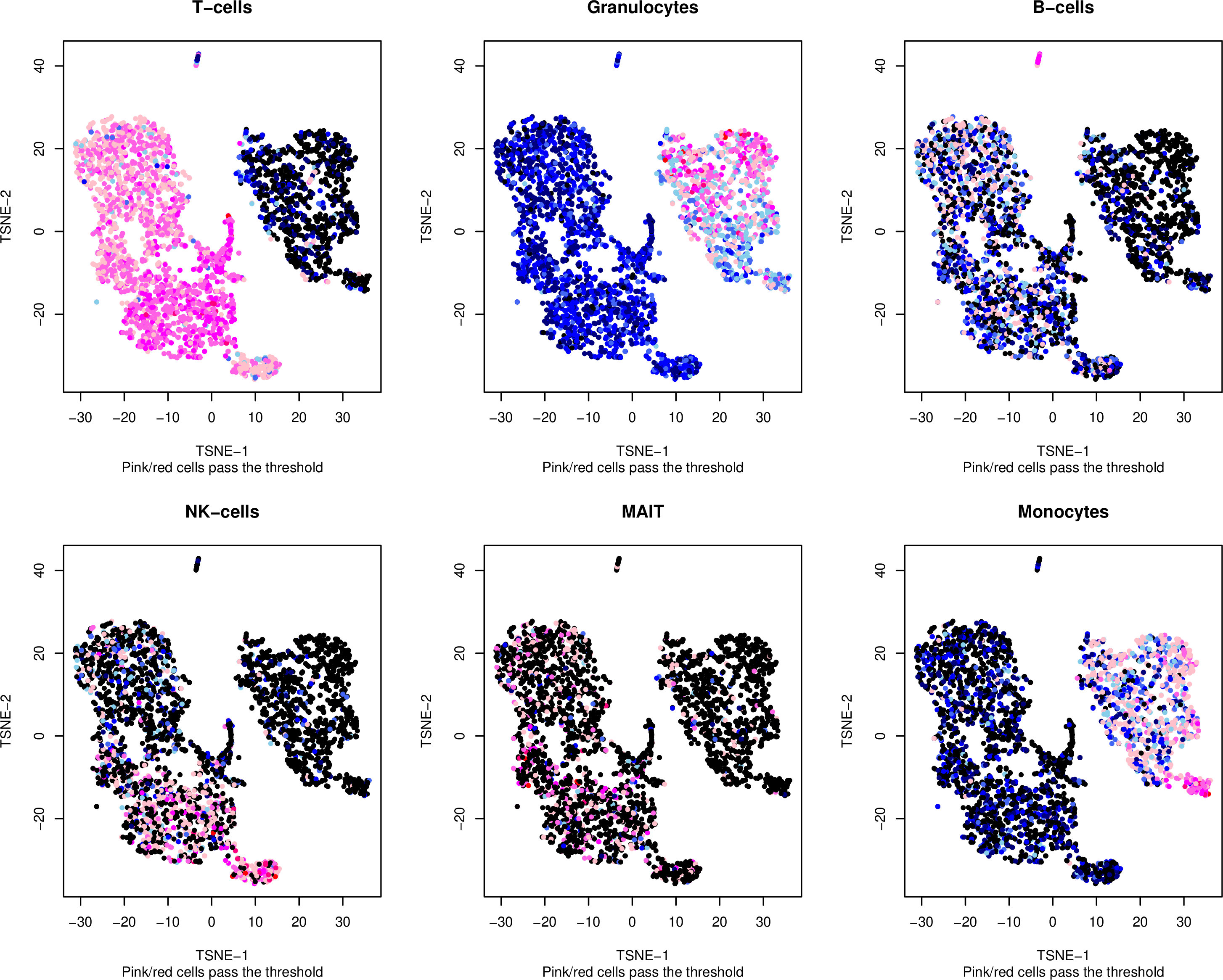

Supplement: Supplementary file 2 — Additional file 2: Supplementary file 2. To demonstrate the utility of scQCEA, we apply the workflow to the sixteen gene expression profiles of eight patients with metastatic melanoma, prepared from pre- and post-treatment experimental batches. You can find the QC interactive report at: https://github.com/isarnassiri/scQCEA/tree/Example-of-Application. Download and unzip the OGC_Interactive_QC_Report_P180121.zip file. You can open CLICK_ME.html file without using rStudio/R. [file 12864_2023_9447_MOESM2_ESM.zip › Inputs/10X-gex/481207_64/P180121-keep_481207_64_tSNE_Plot.png]

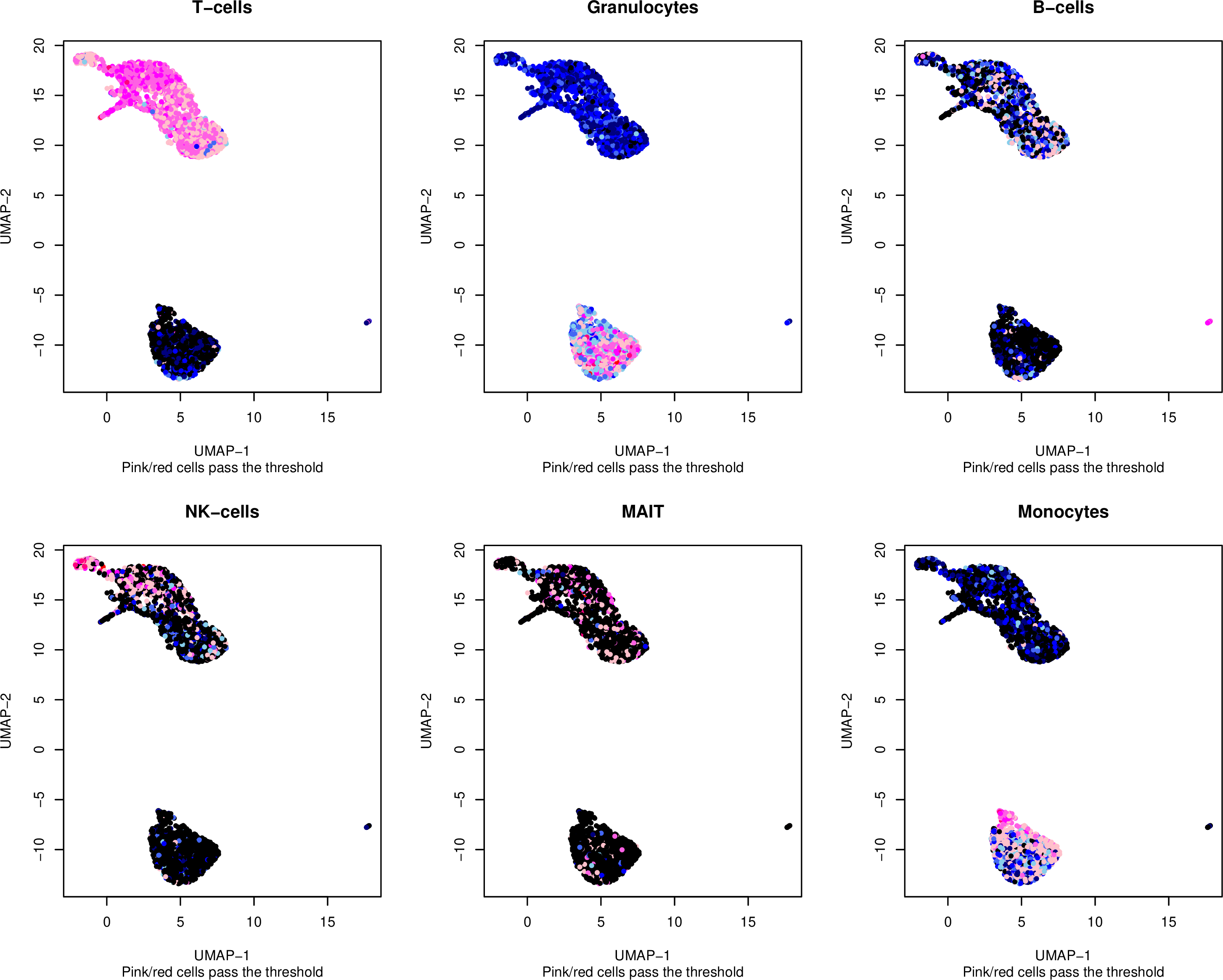

Supplement: Supplementary file 2 — Additional file 2: Supplementary file 2. To demonstrate the utility of scQCEA, we apply the workflow to the sixteen gene expression profiles of eight patients with metastatic melanoma, prepared from pre- and post-treatment experimental batches. You can find the QC interactive report at: https://github.com/isarnassiri/scQCEA/tree/Example-of-Application. Download and unzip the OGC_Interactive_QC_Report_P180121.zip file. You can open CLICK_ME.html file without using rStudio/R. [file 12864_2023_9447_MOESM2_ESM.zip › Inputs/10X-gex/481207_64/P180121-keep_481207_64_UMAP_Plot.png]

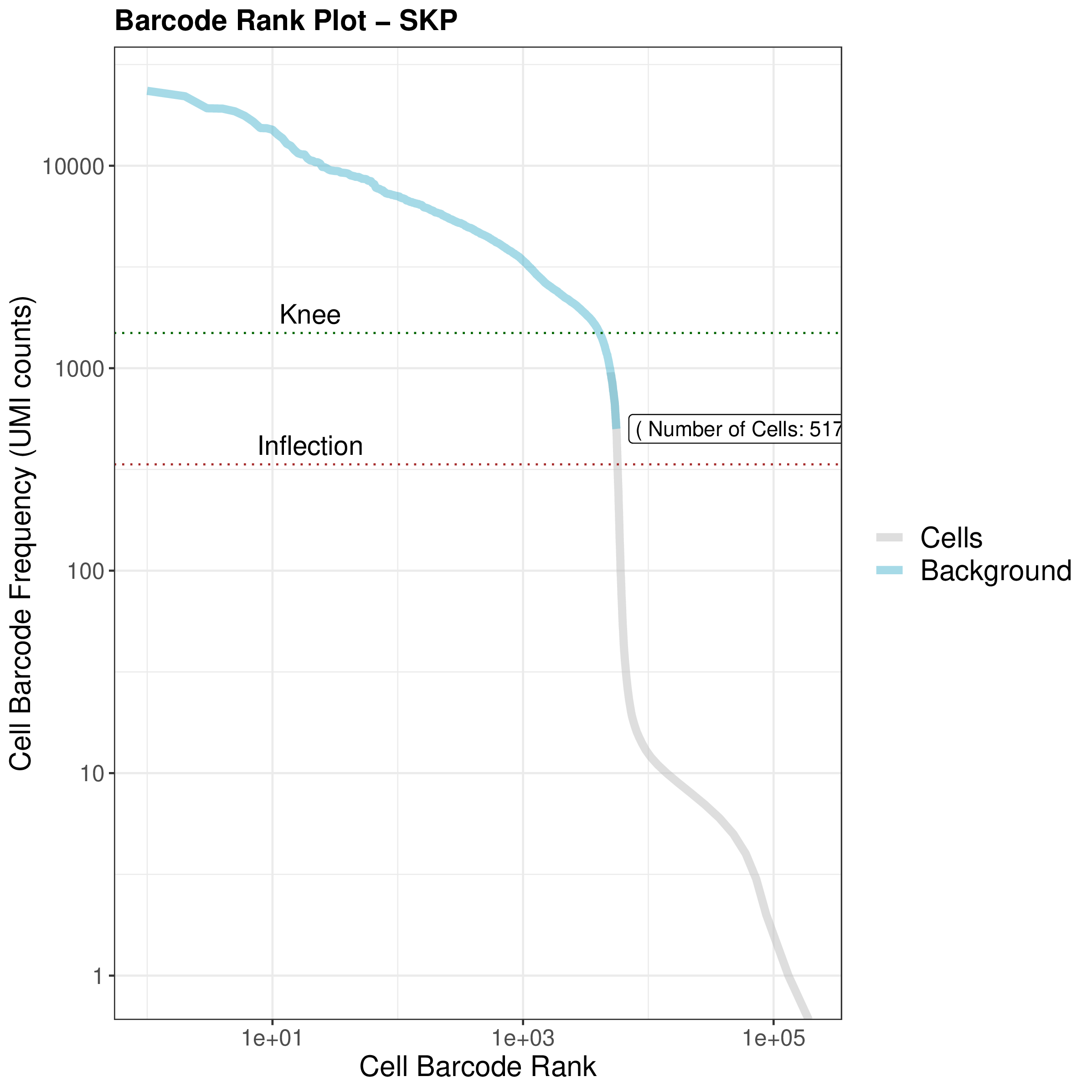

Supplement: Supplementary file 2 — Additional file 2: Supplementary file 2. To demonstrate the utility of scQCEA, we apply the workflow to the sixteen gene expression profiles of eight patients with metastatic melanoma, prepared from pre- and post-treatment experimental batches. You can find the QC interactive report at: https://github.com/isarnassiri/scQCEA/tree/Example-of-Application. Download and unzip the OGC_Interactive_QC_Report_P180121.zip file. You can open CLICK_ME.html file without using rStudio/R. [file 12864_2023_9447_MOESM2_ESM.zip › Inputs/10X-gex/481207_76/P180121-keep_481207_76_BarcodeRankPlot_10X.png]

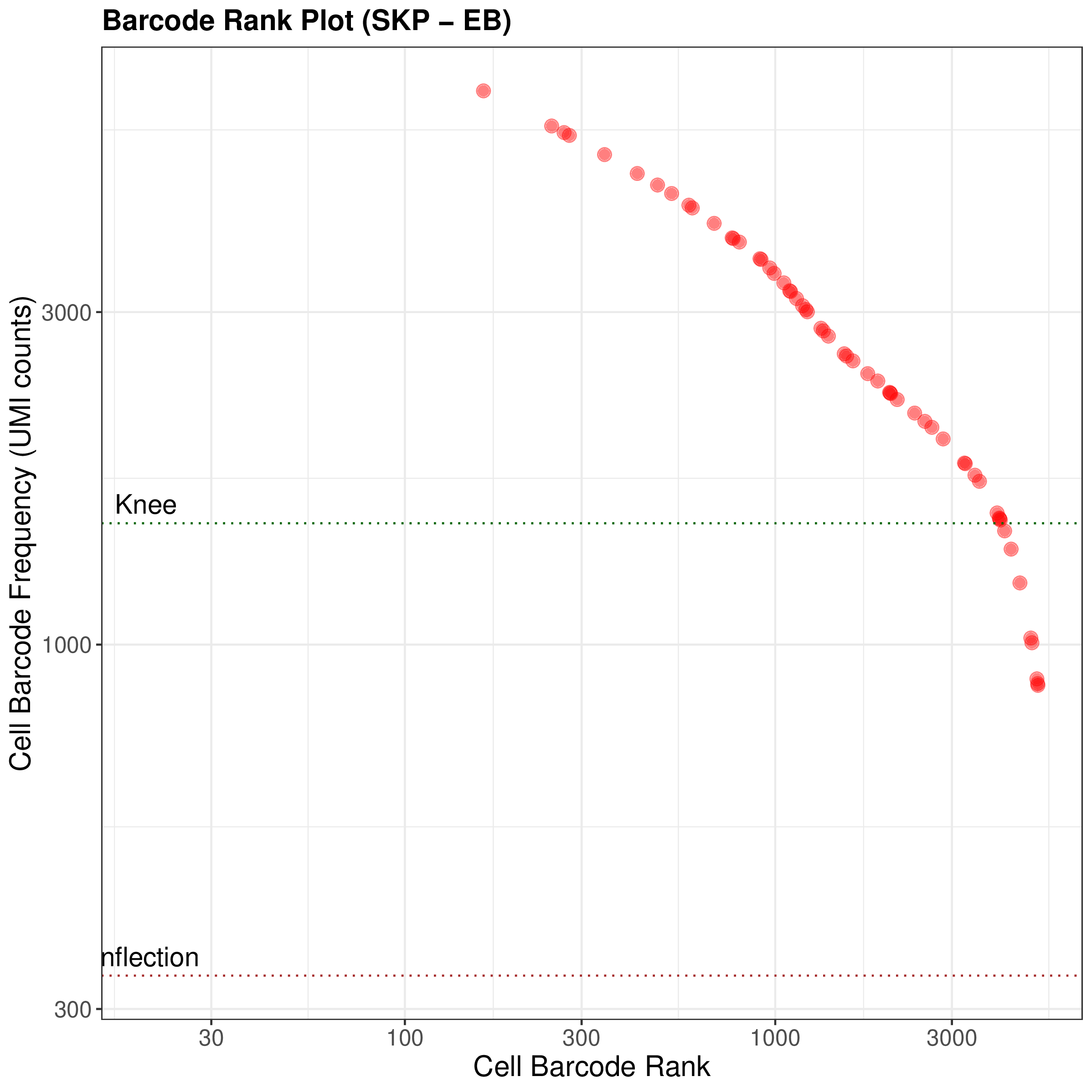

Supplement: Supplementary file 2 — Additional file 2: Supplementary file 2. To demonstrate the utility of scQCEA, we apply the workflow to the sixteen gene expression profiles of eight patients with metastatic melanoma, prepared from pre- and post-treatment experimental batches. You can find the QC interactive report at: https://github.com/isarnassiri/scQCEA/tree/Example-of-Application. Download and unzip the OGC_Interactive_QC_Report_P180121.zip file. You can open CLICK_ME.html file without using rStudio/R. [file 12864_2023_9447_MOESM2_ESM.zip › Inputs/10X-gex/481207_76/P180121-keep_481207_76_BarcodeRankPlot_EB_FilterOut.png]
